# Pan-Cancer Study on Variants of Canonical miRNA Biogenesis Pathway Components: A Pooled Analysis

**DOI:** 10.3390/cancers15020338

**Published:** 2023-01-04

**Authors:** Rami M. Elshazli, Eman A. Toraih, Mohammad H. Hussein, Emmanuelle M. Ruiz, Emad Kandil, Manal S. Fawzy

**Affiliations:** 1 Department of Biochemistry and Molecular Genetics, Faculty of Physical Therapy, Horus University–Egypt, New Damietta 34517, Egypt; 2 Endocrine and Oncology Division, Department of Surgery, Tulane University School of Medicine, New Orleans, LA 70112, USA; 3 Medical Genetics Unit, Department of Histology and Cell Biology, Suez Canal University, Ismailia 41522, Egypt; 4 Department of Pathobiological Sciences, School of Veterinary Medicine, Louisiana State University, Baton Rouge, LA 70803, USA; 5 Department of Medical Biochemistry and Molecular Biology, Faculty of Medicine, Suez Canal University, Ismailia 41522, Egypt; 6 Department of Biochemistry, Faculty of Medicine, Northern Border University, Arar 1321, Saudi Arabia

**Keywords:** machinery genes, non-coding RNA, malignancy, polymorphisms

## Abstract

**Simple Summary:**

Previous relations between microRNA machinery gene variants and human cancer risk have not reach the required statistical power. For the first time, the authors pooled data from 33 studies in “The Cancer Genome Atlas” database that cover 8176 pan-cancer samples which yielded 22 eligible variants within 11 genes subjected to further testing under different genetic association models, Cox regression, and trail sequential analyses. The potential roles of miRNA biogenesis genes in cancer development and prognosis have been concluded.

**Abstract:**

Single nucleotide polymorphisms in genes involved in microRNA processing/maturation and release may deregulate the microRNAome expression levels. We aimed to assess the relationship between miRNA machinery genetic variants and human cancer risk using integrative bioinformatics analyses to identify the role of these genes in cancer aggressiveness. Mutations of 8176 pan-cancer samples were retrieved from 33 studies in “TCGA” database, and a Cox regression model for survival was performed. Next, 22 computationally identified variants within 11 genes were selected based on their high citation rate and MAF. Relevant articles through March 2020 were included. Pooled estimates under the five genetic association models were calculated. Publication bias and heterogeneity between articles were evaluated. Trial Sequential Analysis (TSA) was applied to assess the power and reliability of the draw conclusions. TCGA patients with different cancer types revealed significant alterations in miRNA machinery genes, with mutation frequency ranging from 0.6–13% of samples. RAN was associated with LN metastasis, while TARBP2 and PIWIL1 gene mutations exhibited better overall survival. In the meta-analysis, 45 articles (74,593 cases and 89,198 controls) met the eligibility criteria. Pooled analysis revealed an increased cancer risk with DROSHArs10719*G, RANrs3803012*G, DGCR8rs417309*A, and GEMIN3rs197414*A. In contrast, both DICER1rs1057035*T and GEMIN4rs2743048*G conferred protection against developing cancer. TSA showed the cumulative evidence is inadequate, and the addition of further primary studies is necessary. This study suggests a potential role of miRNA biogenesis genes in cancer development/prognosis. Further functional studies may reveal biological explanations for the differential risks of the machinery variants in different cancer types.

## 1. Introduction

microRNAs (miRNAs) are endogenous small non-coding RNAs that are critical regulators of gene expression at the post-transcriptional level [1]. Typically, miRNAs interact with specific target genes through complementary base-pairing to influence the translation or stability of the target mRNA molecules [2]. Individual miRNAs can control the expression of several hundred genes, and each mRNA can be regulated by multiple miRNAs, forming a complex dynamic crosstalk regulatory network [3,4]. miRNAs are involved in almost all biological processes, including cellular development, metabolism, differentiation, proliferation, and angiogenesis. They may function as either tumor suppressors or promoters, giving miRNAs potential diagnostic and prognostic utility [5,6]. Despite compelling evidence demonstrating that overall global miRNA expression is deregulated in human cancers, the exact underlying mechanisms of miRNA deregulation have not been fully elucidated [7]. In addition, multiple emerging studies delineating the role of selected miRNA panels in tumors have produced inconsistent results.

**Figure 1:**
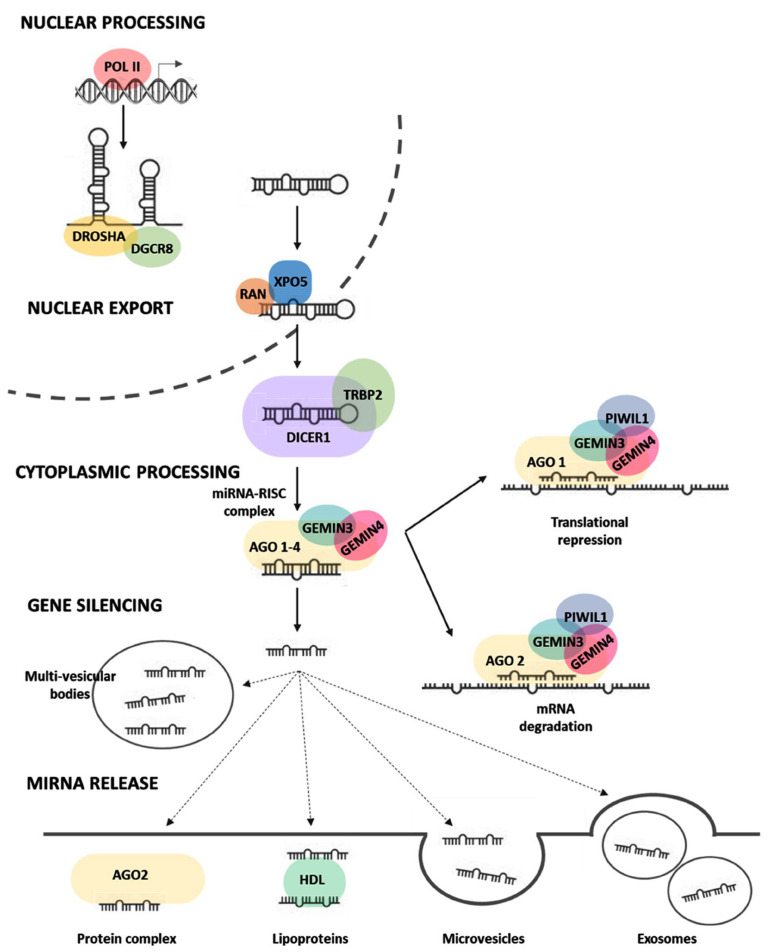
Regulation of microRNA biogenesis (processing, maturation, and release). After transcription of miRNA genes by polymerase II in the nucleus, primary microRNAs (pri-miRNAs) are cleaved by microprocessor DROSHA/DGCR8 complex to liberate 60–70 nucleotide hairpin-shaped precursor microRNAs (pre-miRNAs), which are then exported in the cytoplasm by XPO5/RAN/GTP complex and further cleaved by DICER1 to produce 21–24 nucleotide duplexes. One strand of the miRNA duplex can either associate with the miRNA-induced silencing complex (miRISC) and guide the binding to target mRNAs or be released by the cells. In the former case, sequence complementarity between the seed region of miRNAs and mRNAs mediates gene silencing by targeting mRNA degradation or translational repression in processing bodies (P-bodies). In the latter case, the mature miRNAs can either bind to RNA-binding proteins such as argonaute-2 or to lipoproteins, or miRNAs can be loaded in microvesicles formed by plasma membrane blebbing or in exosomes that are released in the extracellular space upon exocytic fusion of multivesicular bodies with the plasma membrane. Abbreviations: DROSHA: Drosha Ribonuclease III, DGCR8: double-stranded RNA (dsRNA)-binding protein DiGeorge syndrome critical region 8, XPO5: Exportin-5, RAN: RAN, Member RAS Oncogene Family, DICER1: Dicer 1, Ribonuclease Type III, TRABP2: Trans-Activation Responsive RNA-Binding Protein 2, AGO1/2: Protein argonaute-1 and -2, GEMIN3 or DDX20: Gem-associated protein 4 (also known as DEAD-Box Helicase 20), GEMIN4: Gem-associated protein 4; PIWIL1: Piwi-like protein 1, RISC: RNA-induced silencing complex. Figure created by Biorender.com under the license of Eman Toraih.

Canonical miRNA biogenesis begins with generating the primary miRNA (pri-miRNA) transcript in the nucleus, where the microprocessor complex, comprising Drosha and DGCR8, do further cleavage to produce the hairpin-shaped precursor miRNA (pre-miRNA). This pre-miRNA is exported to the cytoplasm in an XPO5/RAN GTP-dependent manner and processed to produce the mature miRNA duplex [8]. Finally, either the 5p or 3p strands of the mature miRNA duplex are loaded into the Argonaute (AGO) family of proteins to form a functional miRNA-induced silencing complex (miRISC) to guide the binding to target mRNAs or be released by the cells (Figure 1).

The incorporation of miRNAs into multivesicular bodies is not a random event. The sorting process can precisely guide intracellular miRNAs into exosomes through five mechanisms [9]. These mechanisms are (1) the miRNA-induced silencing complex (miRISC)-related pathway [10], (2) the neural sphingomyelinase 2 (nSMase2)-related pathway [11], (3) the pathway associated with the 3′ end adenylation and uridylation of miRNAs [12], (4) the pathway associated with miRNA motifs and sumoylated heterogeneous nuclear ribonucleoproteins (hnRNPs) [13], and (5) the ceramide-related pathway [14]. Selected exosomal miRNA cargo modulates cancer intercellular communication, impacting tumor growth, angiogenesis, metastasis, and multiple biological features [9].

Kinetic modeling studies reveal that miRNAs rank among the fastest produced and longest-lived cellular transcripts [15]. There is a strong correlation between the rate of primary miRNA transcription and that of mature miRNAs, reflecting the rapid turnover of miRNA duplexes even before being loaded into miRISC [9]. Notably, an increase in AGO proteins was directly correlated with the increase in miRNA abundance, indicating that miRISC formation can act as a kinetic bottleneck in miRNA homeostasis [16,17]. Another factor that may contribute to the dynamics of miRNA-mediated gene regulation is the functionalized compartmentalization and shuttling of miRISC within the cells [8]. Emerging evidence showing dysregulation of the miRNA biogenesis pathway components in cancer highlights its pathophysiological relevance in tumor development and progression [18,19]. The expression levels of miRNA machinery components such as *DROSHA* and *DICER1* are suppressed in cancer and associated with advanced tumor stage and poor clinical outcomes [20,21]; therefore, studying the genes involved in miRNA biogenesis could provide a comprehensive insight into the spatial perturbation along the signaling pathway and help identify the putative vital players in the miRNA machinery system that may function as promising therapeutic targets for cancer.

Here, we conducted a meta-analysis to assess the associations between these genetic variants and human cancer risk and applied integrative bioinformatics methods to identify their role in cancer aggressiveness. Our results reveal another level of fundamental principles that control miRNA transcriptomic profile and regulation and open new avenues toward understanding the intracellular dynamics of miRNA biogenesis in cancer.

## 2. Materials and Methods

### 2.1. Variant Selection 

Single nucleotide polymorphisms of 11 microRNA machinery genes were retrieved from the Ensembl genome browser 108 (www.ensembl.org) and Varsome (varsome.com) databases [22,23]. These included *DROSHA*, *DICER1*, *XPO5*, *RAN*, *DGCR8*, *GEMIN3*, *GEMIN4*, *AGO1*, *AGO2*, *TARBP2*, and *PIWIL1*. The variant tables were filtered by the presence of citations (Supplementary Table S1). We then identified the number of cited papers investigating the association between any of the abovementioned genes and cancer risk. The 22 SNPs with citations in five or more cancer papers were utilized in the current meta-analysis. These include *DROSHA* (rs10719, rs6877842), *DICER1* (rs13078, rs1057035, rs3742330), *XPO5* (rs11077), *RAN* (rs14035, rs3803012), *DGCR8* (rs3757, rs417309, rs1640299), *GEMIN3* (rs197412, rs197414), *GEMIN4* (rs7813, rs2743048, rs3744741), *AGO1* (rs595961, rs636832), *AGO2* (rs4961280), *TARBP2* (rs784567, rs1106042), and *PIWIL1* (rs10773771). 

**Figure 2:**
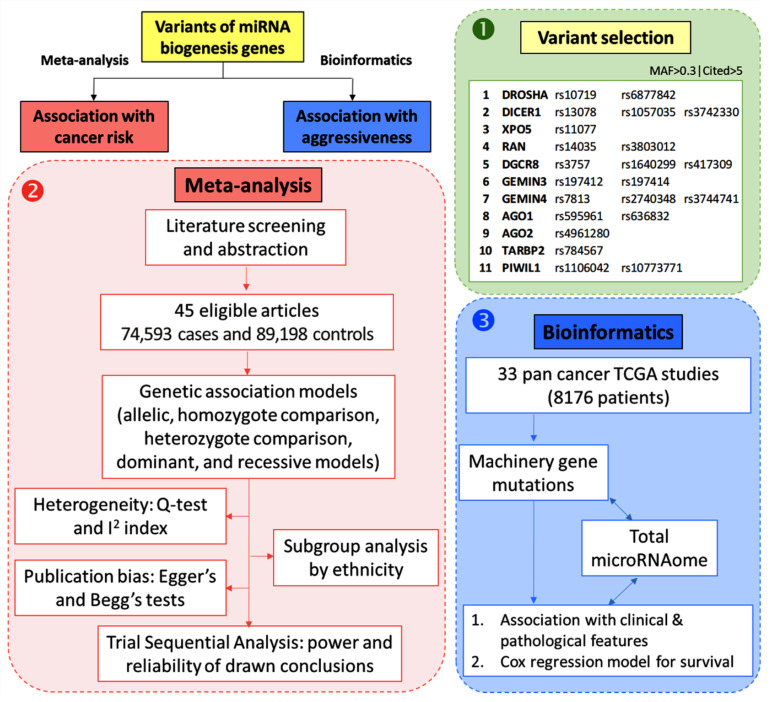
Workflow followed for in silico data analysis and meta-analysis. We followed three steps: (1) Selection of genes enrolled in microRNA machinery canonical pathway followed by a selection of genetic variants with five or more published articles and minor allele frequency (MAF) of more than 0.3 after identification of 11 genes, including 22 single nucleotide polymorphisms. (2) Meta-analysis was performed to pool the results of 45 published articles. Pairwise comparisons were carried out for each genetic association model to identify the association between gene polymorphism and cancer risk. Next, heterogeneity analysis, subgroup analysis, trials sequential analysis, and publication bias were assessed. (3) To explore the association between genetic variants and cancer prognosis, transcriptomic and genomic data of 33 different types of cancer were retrieved from the TCGA database. Genetic mutations of the 11 studied genes were screened in association with the expression of corresponding genes and the total microRNAs expression in each patient. The Mann–Whitney U and Kruskal–Wallis tests were employed to test the association between gene mutation and clinicopathological features, including lymph node metastasis, distant metastasis, and pathological grade. Survival analysis was performed using Cox regression analysis and Kaplan–Meier curve plots were generated. Both univariate and multivariate analyses using total miRNAome level were conducted.

As depicted in Figure 2, a meta-analysis was performed to identify the association between the 22 SNPs and susceptibility to cancer development. In addition, due to the low number of published papers on the association between these genes and cancer progression and aggressiveness features, we performed bioinformatics analysis using TCGA data of 33 cancer types in 8176 cancer patients.

### 2.2. Meta-Analysis on miRNA Biogenesis Gene Variants and Cancer Risk

We performed a systematic review and meta-analysis according to the “meta-analysis of observational studies in epidemiology (MOOSE)” guidelines [24]. The study results were reported following “preferred reporting items for systematic reviews and meta-analyses protocols (PRISMA-P)” regulations [25].

#### 2.2.1. Search Strategy

Two independent investigators (RE, MH) searched PubMed, Web of Science, Science Direct, and Scopus search engines up to March 2020 for the following terms: “genotype”, “allele”, “genetics”, “inheritance”, “polymorphism”, “variant”, “SNP”, AND “microRNA”, “miRNA”, “machinery”, “processing”, “maturation”, “canonical pathway”, AND “cancer”, “tumor”, “neoplasm”, “malignancy”, “carcinoma”. An additional search was performed using the gene names or their aliases and their selected variants: “DROSHA”, “rs10719”, “rs6877842”, “DICER1”, “rs13078”, “rs1057035”, “rs3742330”, “XPO5”, “rs11077”, “RAN”, ‘rs14035”, “rs3803012”, “DGCR8”, “rs3757”, “rs417309”, “rs1640299”, “GEMIN3”, “rs197412”, “rs197414”, “GEMIN4”, “rs7813”, “rs2743048”, “rs3744741”, “AGO1”, “rs595961”, “rs636832”, “AGO2”, “rs4961280”, “TARBP2”, “rs784567”, “PIWIL1”, “rs1106042”, “rs10773771”. Retrieved articles were manually checked for additional references. No constraints for publication date, country, language, sample size, or ethnic origin were applied to limit the publication bias raised throughout the construction of this survey.

#### 2.2.2. Identification of Eligible Studies

Articles were included in the study if (1) they reported an association between at least one of the selected gene variants and cancer risk, (2) genotype frequencies were available, and (3) the paper was presented as a case–control study. Articles were excluded if they (1) were review articles, abstracts, conference proceedings, or case reports, (2) were non-human studies, (3) lacked data necessary for a systematic review, or (4) retrieved repeated published data from previous articles. The discrepancy in study selection was resolved by a discussion with a third author (MSF).

#### 2.2.3. Data Extraction

Two-step screening of articles was performed by two independent authors (RE and ET), inspecting titles/abstracts and examining full text published articles for eligibility. Appropriate data were extracted from the previously published articles in consistent tabulated forms. Study characteristics were collected including first author surname, publication year, country of origin, ethnicity of subject, sample size for cancer and non-cancer cohorts, genotyping method, source of controls (“hospital-based” or “population-based”), the allelic and genotypic distributions for gene variants among cancer and non-cancer subjects, and *p*-value related to Hardy–Weinberg Equilibrium (HWE) for non-cancer controls. Published articles including different sets of comparison groups were extracted individually, and disagreements were discussed to reach an optimum conclusion with the aid of a third investigator (MSF).

#### 2.2.4. Quality Scoring

Quality assessment of articles was conducted based on defined criteria described in a prior publication [26] (Supplementary Tables S2 and S3). These included the patients’ representativeness, control source, ascertainment, case-control match, genotyping method, quality control, genotyping analysis, specimens used for ascertaining genotypes, HWE in controls, total sample size, and association evaluation. Quality scores ranged from 0 (worst) to 21 (best), and articles with scores ≥12 were designated as “high quality”.

#### 2.2.5. Statistical Analysis for Pooling Results

The genotypic and allelic distributions of the gene variants were analyzed among different ethnic subjects as previously described [27]. The HWE within non-cancer subjects was evaluated using the chi-square test [28]. Pooled odds ratio (OR) along with their equivalent 95% confidence interval (CI) were estimated to explore the potential impact of variants on the susceptibility of developing cancer under various inheritance models (allelic model, recessive model, dominant model, homozygote comparison, and heterozygote comparison) [29]. The comprehensive meta-analysis software version 3.0 (Biostat, Englewood, NJ, USA) was used for statistical calculation, and STATA 16.0 was used to generate the summarized forest plots.

#### 2.2.6. Heterogeneity Assessment

The chi-square-based *Q*-statistic test [30] along with the *I*^2^ index [31] were used to estimate heterogeneity among numerous published articles. A fixed-effects model was designated to compute the pooled OR for each individual study in cases where the *I*^2^ index < 50% (*p*-value ≥ 0.10) [32], while a random-effects model was applied to ascertain within-study inaccuracies if the heterogeneity was achieved with the *I*^2^ index > 50% (*p*-value < 0.10) [33]. 

#### 2.2.7. Subgroup Analysis

Stratification analysis based on ethnic origin (Caucasian, Asian, Latin American, African, Turkish), cancer type (breast, lung, hepatocellular, gastric, colorectal, bladder, renal cell carcinoma, papillary thyroid, larynx, Chronic lymphocytic leukemia (CLL), cervical, esophageal, Hodgkin lymphoma, Non-Hodgkin lymphoma, or other cancerous diseases), genotyping method (TaqMan, Sequenome mass spectrometry, restriction fragment length polymorphism polymerase chain reaction (RFLP PCR), SNPlex technology, or others), source of controls (population-based or hospital-based), HWE within controls (equilibrium or disequilibrium), and quality (high or low score) were constructed.

#### 2.2.8. Publication Bias

The impact of publication bias was quantified by the utilization of Begg’s funnel plot along with Egger’s linear regression approach [34]. A significant publication bias was detected using the asymmetric funnel model (*p*-value < 0.1), and the adjustment of bias was achieved by applying the trim and fill procedure [35]. 

#### 2.2.9. Trial Sequential Analysis (TSA) ta Extraction

The statistical reliability of this meta-analysis was tested with the aid of TSA by combining the cumulative sample trials of all available studies using the statistical threshold to decrease inadvertent errors and improve the strength of expectations [36,37]. Firstly, two side trials were used with type I error (α) and a power set of 5% [38]; then, when the cumulative Z-curve crossed the monitoring boundaries, a reasonable level of influence was achieved, and no further complementary trials were necessary. However, if the Z-curve failed to reach the threshold boundaries, the expected sample sizes were shown to have not completed the requirements to achieve a suitable significance, and additional trials were required. TSA software version 0.9.5.10 beta was applied in this trial.

### 2.3. In Silico Analysis on miRNA Biogenesis Gene Mutations and Cancer Prognosis

#### 2.3.1. Data Source

Mutation data for the pan-cancer study was collected through the cBioPortal web platform. A total of 33 studies were downloaded and analyzed. These included ACC (Adrenocortocal carcinoma), BLCA (bladder urothelial carcinoma), BRCA (Breast ductal and lobular carcinoma), CESC (cervical carcinoma), CHOL (cholangiocarcinoma), COAD (Colorectal adenocarcinoma), DLBC (diffuse large B-cell lymphoma), ESCA (esophageal carcinoma), GBM (gliobastoma multiforme), HNSC (head and neck squamous cell carcinoma), KICH (kidney chromophobe carcinoma), KIRC (kidney clear cell carcinoma), KIRP (kidney papillary cell carcinoma), AML (acute myeloid leukemia), LGG (lower grade glioma), LIHC (liver hepatocellular carcinoma), LUAD (lung adenocarcinoma), LUSC (lung squamous cell carcinoma), MESO (mesothelioma), OV (ovarian serous adenocarcinoma), PAAD (pancreatic ductal adenocarcinoma), PCPG (paraganglioma and pheochomocytoma), PRAD (prostate adenocarcinoma), READ (rectum adenocarcinoma), SARC (sarcoma), SKCM (skin cutaneous melanoma), STAD (stomach–gastric adenocarcinoma), TCGT (testicular germ cell cancer), THCA (thyroid papillary carcinoma), THYM (thymoma), UCEC (uterine corpus endometroid carcinoma), UCS (uterine carcinosarcoma), and UVM (uveal melanoma). Clinicopathological data were downloaded from previous pan-cancer studies [39,40].

#### 2.3.2. The Total microRNAome Expression Level

Across all cancers, the expression of all mature microRNAs of 717 paired tumors and normal samples were compared using the Mann–Whitney U test. Next, the z scores of mutants versus wild-type samples were compared for all tumors using the Mann–Whitney U test, and boxplots were generated. The total miRNome has been estimated as the sum of all the miRNAs expressed in each sample, hence reflecting the total quantity of miRNA present per sample. 

#### 2.3.3. Association between Gene Mutations and Total miroRNome and Clinicopathological Characteristics

To identify the impact of miRNA machinery gene mutation on clinicopathological features of tumors, we performed linear regression (for continuous variables) and logistic regression (for categorical variables) using *lm* and *glm* R functions. Heatmap and dot plots were generated with *pheatmap* and *ggplot2* R packages. Data are presented as OR and 95% CI. Multivariate analysis was performed using the z scores for total microRNome levels. Overall analysis for all cancers and stratification by the type of cancer were performed.

#### 2.3.4. Survival Analysis

Univariate and multivariate Cox regression and Kaplan–Meier survival analyses were performed using *survival* and *survminer* R packages. Briefly, Cox analysis was run with *the coxph* function, and the Kaplan–Meier curve was fit with *survfit* (conf.type = “log-log”) and plotted with *ggsurvplot*. Statistical differences were analyzed with *survdiff* (rho = 0). Definitions of survival times are described in our prior publication [41].

#### 2.3.5. Principal Component Analysis 

A principal component analysis (PCA) was performed with the 11 gene mutants across the pan-cancer study using the *FactoMineR* and *factoextra* R packages. Next, a PCA index score was calculated according to the equation:

*W* = ∑(|*Lij*| ∗ *Ei*)

*PCAIndex score* = ∑ *Xi* ∗ *Wi* ∑ *Wi*

with *Lij*, loading value of the *i*th variable of grouping on *j*th *PC*, *Ei*, the eigenvalue of the *j*th *PC*, *Wi*, weight of the *i*th variable, *Xi*, the normalized value of *i*th variable.

From the PCA, hierarchical clustering was performed with the HCPC R function (FactoMineR R packages). Ten clusters were identified. Regression and distribution analyses were performed between the ten PCA clusters and clinical/survival parameters. For each parameter analyzed, clusters were compared with each other, and *p*-values were clustered to extract similar and dissimilar clusters, reducing the number of clusters. Further analyses with all the clinical and survival parameters were performed. The cluster classification presenting the highest significant *p*-value was chosen as the most optimized cluster stratification. Cluster A gathered the PCA clusters 1, 2, 3, and 5; cluster B, the PCA clusters 7 and 6; cluster C, the PCA clusters 9 and 10; and cluster D, the PCA clusters 4 and 8.

## 3. Results

### 3.1. Meta-Analysis on miRNA Biogenesis Gene Variants and Cancer Risk

#### 3.1.1. Characteristics of Studies Included in the Meta-Analysis

**Figure 3:**
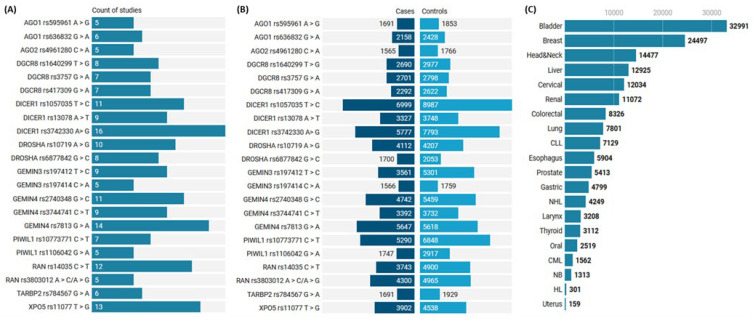
Characteristics of enrolled studies and SNP. (**A**) The number of studies included in the analysis of each gene variant. (**B**) The number of subjects demonstrating cases and controls. Y axis represents gene ID and SNP ID. (**C**) Count of study subjects included in the meta-analysis based on the cancer site. CLL: chronic lymphocytic leukemia, CML: chronic myeloid leukemia, HL: Hodgkin lymphoma, NB: neuroblastoma, NHL: non-Hodgkin lymphoma.

In the current study, 45 articles with 188 allelic discrimination comparisons of 163,791 cohorts (74,593 cancer cases and 89,198 controls) were included [42,43,44,45,46,47,48,49,50,51,52,53,54,55,56,57,58,59,60,61,62,63,64,65,66,67,68,69,70,71,72,73,74,75,76,77,78,79,80,81,82,83,84,85,86] (Supplementary Table S4). The sample size for each study ranged from 159 to 3003. The number of studies and the study population for each SNP are shown in Figure 3A,B. Based on the literature of eligible articles, a total of 20 different cancers were analyzed (Figure 3C and Supplementary Table S5). Studies covered different geographical distributions, with the majority in China (58 comparisons) and the USA (48 comparisons), followed by Korea (30 comparisons) and Spain (12 comparisons). The assessment score of included studies is listed in Supplementary Table S3. A high score was reported in 138 comparisons compared to 50, with a low-quality score of <12. In addition, 22 genotyping data deviated from HWE and were considered during sensitivity analysis.

#### 3.1.2. Pooled Analysis of Pairwise Comparisons

**Figure 4:**
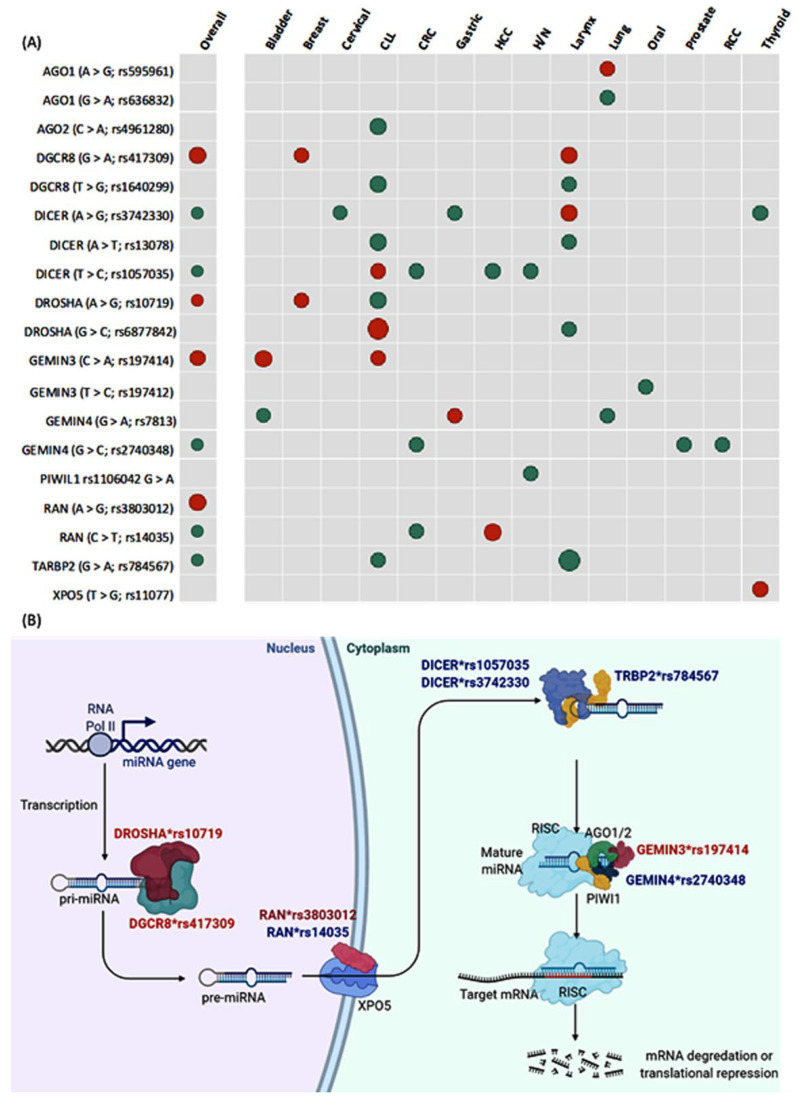
Pooled cancer risk of microRNA machinery gene variants. (**A**) Dot plot showing genes with significant high risk (red) and low risk (green) for cancer development. The size of the dot is proportional to the odds ratio. The matrix shows the association of combined studies of different types of cancer in addition to the data split for each type. Cancer types with insignificant results across all variants were not included in the plot. (**B**) Biogenesis of microRNA machinery pathway showing cellular localization of action for the significant genes and polymorphisms. Out of the 22 studied genetic variants, 9 SNPs were associated with cancer risk. The red gene with SNP ID is associated with higher cancer risk, while the blue gene and SNP ID conferred protection against cancer.

As summarized in Figure 4A, the overall analysis revealed higher susceptibility of cancer with the following polymorphisms; (1) *DROSHA* (A > G; rs10719) under both recessive model (OR = 1.19, 95% CI = 1.0–1.41, *p* = 0.043) and homozygote comparison model (OR = 1.19, 95% CI = 1.0–1.42, *p* = 0.046); (2) *DGCR8* (G > A; rs417309) under allelic model (OR = 1.26, 95% CI = 1.07–1.47, *p* = 0.003), recessive model (OR = 2.58, 95% CI = 1.47–4.51, *p* = 0.001), dominant model (OR = 1.19, 95% CI = 1.0–1.41, *p* = 0.040), and homozygote comparison model (OR = 2.65, 95% CI = 1.51–4.66, *p* = 0.001); (3) *RAN* (A > G; rs3803012) under recessive model (OR = 1.93, 95% CI = 1.03–3.61, *p* = 0.040) and homozygote comparison model (OR = 1.93, 95% CI = 1.03–3.62, *p* = 0.039); and (4) *GEMIN3* (C > A; rs197414) under recessive model (OR = 1.72, 95% CI = 1.05–2.83, *p* = 0.030) and homozygote comparison model (OR = 1.72, 95% CI = 1.05–2.84, *p* = 0.031).

In contrast, another five variants conferred protection against cancer; (1) *RAN* (C > T; rs14035) under heterozygote model (OR = 0.84, 95% CI = 0.72–0.98, *p* = 0.036); (2) *DICER1* (T > C; rs1057035) under allelic model (OR = 0.88, 95% CI = 0.82–0.95, *p* = 0.001); (3) *DICER1* (A > G; rs3742330) under homozygote comparison model (OR = 0.87, 95% CI = 0.77–0.99, *p* = 0.046); (4) *TARBP2* (G > A; rs784567) under allelic model (OR = 0.88, 95% CI = 0.78–0.99, *p* = 0.046) and homozygote comparison model (OR = 0.77, 95% CI = 0.61–0.99, *p* = 0.043); and (5) *GEMIN4* (G > C; rs2740348) under heterozygote comparison model (OR = 0.87, 95% CI = 0.78–0.96, *p* = 0.011). There was homogeneity between studies except for *RAN* (C > T; rs14035) (I^2^ = 58.9%, *p* = 0.005), *TARBP2* (G > A; rs784567) (I^2^ = 86.7%, *p* < 0.001), and *GEMIN4* (G > C; rs2740348) (I^2^ = 47.6%, *p* = 0.039). Figure 4B shows the cellular location to highlight the biological function of miRNA machinery genes studied in the current meta-analysis and depicts the significant SNPs that were associated with higher and lower risk of cancer development.

#### 3.1.3. Subgroup Analysis and Publication Bias

Iteration of pairwise comparisons stratified by geographical region, cancer type, genotyping method, sources of control subjects, agreement with HWE, and quality score are depicted in Supplementary Tables S6–S27. Egger’s regression test revealed publication bias in the association between RAN*rs14035 and cancer susceptibility (*p* = 0.046).

For each type of cancer, only one or two studies were available; thus, heterogeneity and publication bias assessments were not performed. However, it was noted that some genetic variants were associated with an increased risk of specific cancer and decreased susceptibility to other types. DICER1*rs3742330 increased the risk of laryngeal cancer (OR = 2.21) and conferred protection against cervical (OR = 0.73), gastric (OR = 0.70), and thyroid cancers (OR = 0.55). DICER1*rs1057035 is associated with high risk in CLL (OR = 1.85) and decreased cancer risk in CRC (OR = 0.62), HCC (OR = 0.78), and head and neck cancers (OR = 0.81). Similarly, DROSHA*rs10719 increases the risk of breast cancer (OR = 1.39) while decreasing the susceptibility of developing CLL (OR = 0.58). In contrast, DROSHA*rs6877842 carriers were three times more likely to develop CLL (OR = 3.15) and were associated with a 46% decreased risk in laryngeal cancer (OR = 0.56). Similar contradictory effects were found in GEMIN4*rs781 [gastric (OR = 1.46) versus bladder (OR = 0.76) and lung cancer (OR = 0.79)] and RAN*rs14035 results [HCC (OR = 3.11) versus CRC (OR = 0.75)].

#### 3.1.4. Trial Sequential Analysis

**Figure 5:**
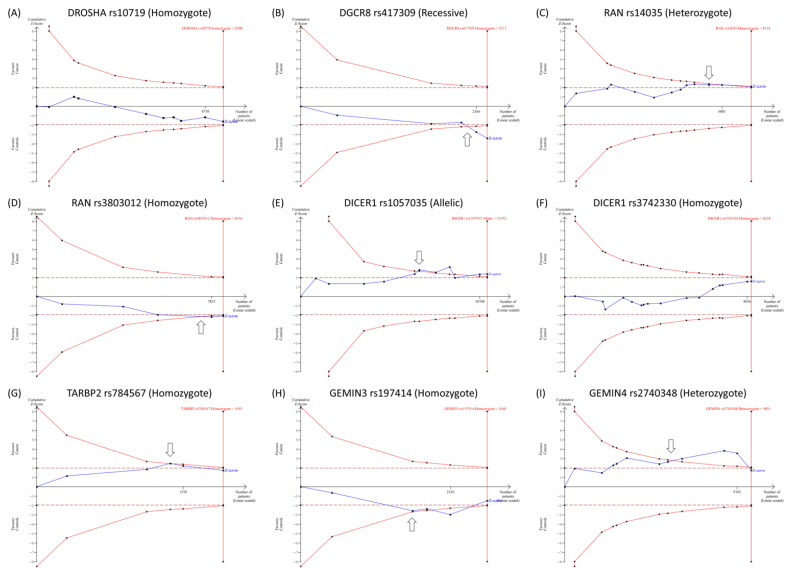
Trial sequential analysis (TSA) for gene polymorphisms. Variants with ten studies or more were tested for TSA. (**A**) TSA for *DROSHA* (A > G; rs10719) in 20 studies under homozygote model. (**B**) TSA for *DGCR8* (G > A; rs417309) in 24 studies under the recessive model. (**C**) TSA for *RAN* (C > T; rs14035) in 12 studies under heterozygote model. (**D**) TSA for *RAN* (A > G; rs3803012) in 10 studies under homozygote model. (**E**) TSA for *DICER1* (T > C; rs1057035) in 11 studies under the allelic model. (**F**) TSA for *DICER1* (A > G; rs3742330) in 16 studies under homozygote model. (**G**) TSA for *TARBP2* (G > A; rs784567) in 10 studies under homozygote model. (**H**) TSA for *GEMIN3* (C > A; rs197414) in 10 articles under homozygote model. (**I**) TSA for *GEMIN4* (G > C; rs2740348) in 11 studies under heterozygote model.

TSA was applied to assess the power and reliability of drawn conclusions. Our results revealed that the cumulative Z-curve for DGCR8*rs417309, RAN*rs14035, RAN*rs3803012, DICER1*rs1057035, TARBP2*rs784567, GEMIN3*rs197414, and GEMIN4*rs2740348 transverses the monitoring boundaries of the trial sequential before it approximates the requested sample sizes, indicating that accumulative evidence was convenient, and no further trials are required. However, cumulative evidence for the other genetic variants was inadequate, and additional primary studies are necessary to validate the outcomes (Figure 5).

### 3.2. Bioinformatics on miRNA Biogenesis Gene Mutation and Cancer Prognosis

#### 3.2.1. Mutation Rates in Cancer

**Figure 6:**
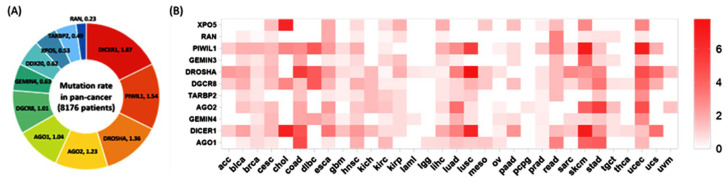
The mutation rate of the 11 genes across the pan-cancer study. (**A**) The left panel shows the proportion of mutations of 11 genes in all cancers included in the study. The percentage of mutant samples is shown for each gene. (**B**) Heatmap depicting the mutation rate of the 11 genes in the 33 cancer types included in the pan-cancer study. Higher color intensity indicates a higher mutation rate. ACC (adrenocortocal carcinoma), BLCA (bladder urothelial carcinoma), BRCA (breast ductal and lobular carcinoma), CESC (cervical carcinoma), CHOL (cholangiocarcinoma), COAD (colorectal adenocarcinoma), DLBC (diffuse large B-cell lymphoma), ESCA (esophageal carcinoma), GBM (gliobastoma multiforme), HNSC (head and neck squamous cell carcinoma), KICH (kidney chromophobe carcinoma), KIRC (kidney clear cell carcinoma), KIRP (kidney papillary cell carcinoma), LGG (lower grade glioma), LIHC (liver hepatocellular carcinoma), LUAD (lung adenocarcinoma), LUSC (lung squamous cell carcinoma), MESO (mesothelioma), OV (ovarian serous adenocarcinoma), PAAD (pancreatic ductal adenocarcinoma), PCPG (paraganglioma and pheochromocytoma), PRAD (prostate adenocarcinoma), READ (rectum adenocarcinoma), SARC (sarcoma), SKCM (skin cutaneous melanoma), STAD (stomach–gastric adenocarcinoma), TCGT (testicular germ cell cancer), THCA (thyroid papillary carcinoma), UCEC (uterine corpus endometroid carcinoma), UCS (uterine carcinosarcoma), and UVM (uveal melanoma). THYM (thymoma) in the TCGA pan-cancer data is not shown as there was not any mutation of the studied genes.

Thirty-three different cancer studies were collected through The Cancer Genome Atlas, corresponding to 8176 samples with mutation information for 11 genes. In the pan-cancer study, average mutation rates ranged from 0.23% for the RAN gene to 1.87% for the DICER1 gene. As for cancer type, there was a wide variation across studies (Figure 6). Higher mutation rates were evident in gastrointestinal tumors (esophagus, gastric, gall bladder, and colorectal), uterine cancer, melanoma, and lung carcinoma.

#### 3.2.2. Association with Clinical and Pathological Characteristics

**Figure 7:**
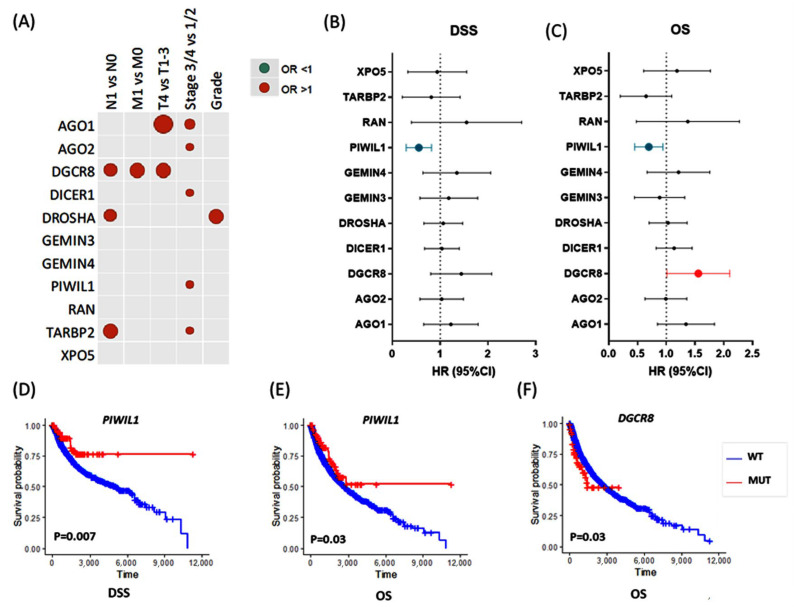
The 11 miRNA machinery gene mutations are potential prognosis markers in a pan-cancer study. Comparisons for mutant versus wild type were performed. (**A**) Dot plots show the association between the gene mutants and advanced pathological characteristics. The higher odds ratio of univariate regression analysis imposed by each gene mutation on various pathological parameters of overall pan-cancer samples. The size of the dots is equivalent to the *p*-values. Only significant associations are shown. N: lymph node stage, M: metastasis stage, T: tumor size stage. (**B**,**C**) Association of gene mutations with disease-specific survival (DSS) and overall survival (OS). Data of multivariate Cox regression models are presented as hazard ratio (HR) and 95% confidence interval (CI). (**D**–**F**) Kaplan–Meier curves for survival analysis to compare mutant (MUT) and wild-type (WT) genes. The X-axis shows the survival times in days. Log-rank test was used. Only significant associations and survival curves are shown; these include the Kaplan–Meier curve for PIWIL1 and DGCR8 according to OS, and PIWIL1 according to DSS. The results of other genes are summarized in supplementary materials. DSS, Disease-specific survival; OS, Overall survival.

To assess the association between gene mutation and aggressive features or cancer progression, we performed univariate and multivariate logistic regression analyses for clinical and pathological parameters. Patients with TARBP2 gene mutation were more likely to have lymph node metastasis (OR = 2.49, 95% CI = 1.14–5.46, *p* = 0.022) and advanced disease stage (OR = 2.20, 95% CI = 1.08–4.46, *p* = 0.029). DROSHA mutation was significantly associated with lymph node metastasis (OR = 1.74, 95% CI = 1.04–2.92, *p* = 0.035) and poor pathological grade (OR = 2.16, 95% CI = 1.09–4.28, *p* = 0.027). Mutation of DGCR8 gene showed a significant association with lymph node metastasis (OR = 1.80, 95% CI = 1.02–3.17, *p* = 0.041), high tumor size (OR = 2.73, 95% CI = 1.45–5.13, *p* = 0.002), and distant metastasis (OR = 2.45, 95% CI = 1.03–5.85, *p* = 0.043). AGO1 gene mutation was more enriched in T4 compared to T1/2/3 tumor stage (OR = 2.95, 95% CI = 1.52–5.72, *p* = 0.001) and in stages III/IV compared to stages I/II (OR = 2.34, 95% CI = 1.36–4.0, *p* = 0.002). AGO2 (OR = 1.11, 1.81–2.95, *p* = 0.017), DICER1 (OR = 1.09, 95% CI = 1.60–2.34, *p* = 0.016), and PIWIL1 (OR = 1.07, 95%CI = 1.61–2.43, *p* = 0.023) genetic alterations were associated with advanced disease stage (Figure 7A).

After adjustment by total microRNAome level, lymphatic infiltration was more likely in patients with DGCR8 (OR = 1.83, 95%CI = 1.04–3.22, *p* = 0.037) and TARBP2 (OR = 2.65, 95%CI = 1.17–6.01, *p* = 0.020) mutations. Cohorts carrying DGCR8 mutations had 2.5 times more risk for distant metastasis (OR = 2.5, 95%CI = 1.05–5.96, *p* = 0.039). The risk of poorly differentiated tumors was twofold in DROSHA mutation carriers (OR = 2.0, 95%CI = 1.0–3.98, *p* = 0.049). Larger tumor size was associated with both AGO1 (OR = 2.54, 1.25–5.18, *p* = 0.010) and DGCR8 (OR = 2.75, 1.47–5.17, *p* = 0.002) mutations. A higher risk of advanced clinical stage was observed in AGO1 (OR = 1.34, 2.33–4.05, *p* = 0.003), AGO2 (OR = 1.16, 1.91–3.15, *p* = 0.011), DICER1 (OR = 1.09, 1.61–2.37, *p* = 0.017), PIWIL1 (OR = 1.10, 1.68–2.54, *p* = 0.015), and TARBP2 (OR = 1.10, 2.29–4.77, *p* = 0.026) mutant patients.

#### 3.2.3. Survival Analysis 

As depicted in Figure 7B,C, multivariate Cox regression analysis revealed that PIWIL1 mutant was associated with better disease-specific survival (DSS) (HR = 0.51, 95% CI = 0.31–0.84, *p* = 0.008) and overall survival (OS) (HR = 0.68, 95% CI = 0.47–0.97, *p* = 0.034). In contrast, DGCR8 mutation carriers presented a worse OS (HR = 1.51, 95% CI = 1.05–2.18, *p* = 0.027). Similarly, Kaplan–Meier curves (Figure 7D–F) revealed higher five-year DSS and OS survival for PIWIL1 mutant patients (DSS: 79.3% vs. 68.2%, *p* = 0.007; OS: 68.8% vs. 59.1%, *p* = 0.033), and lower five-year OS survival for DGCR8 mutant cohorts (48.2% vs. 59.8%, *p* = 0.026) compared to wild-type patients. Supplementary Tables S28 and S29 summarize the results of each gene in the overall TCGA dataset.

Next, we checked if the panel of miRNA machinery gene mutations, estimated as mutation count, could be a prognosis marker. Interestingly, multivariate Cox showed significance for disease-free survival (DFS) and progression-free interval (PFI) outcome markers, with a high number of mutations related to a worse prognosis (DFS, HR = 1.19, 95% CI = 1.03–1.38, *p* = 0.018; PFI, HR = 1.20, 95% CI = 1.06–1.37, *p* = 0.005) and a decrease in the survival rate (DFS: 51% [45–56%] vs. 57% [55–59%], *p* = 0.028; PFI: 47% [42–42%] vs. 54% [52–55%], *p* = 0.003) (Data not shown).

#### 3.2.4. Total microRNome Analysis

**Figure 8:**
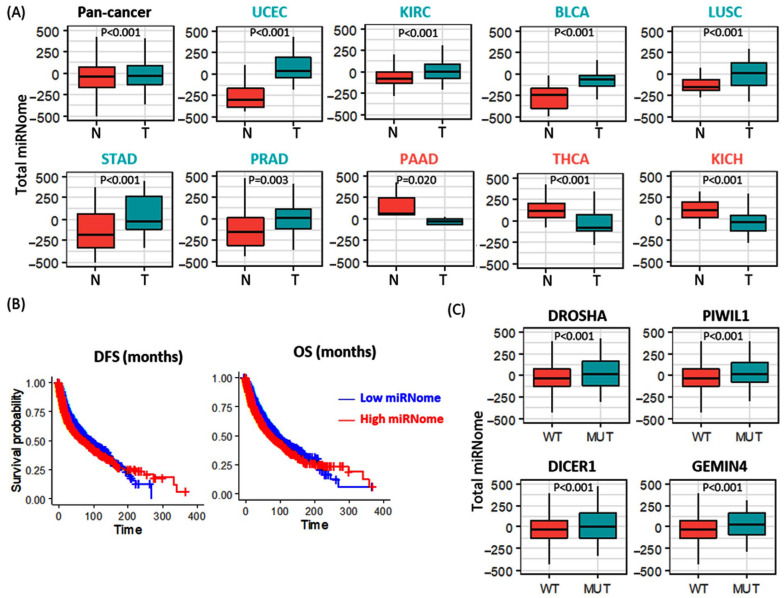
Differential expression of total microRNome level in cancer. Genes with significant results are only shown. (**A**) Total microRNome levels in paired tumor (T) and normal (N) samples. Overall and stratified analyses were performed. Y axis represents the average z score for all mature miRNAs per the study. Across the pan-cancer subtypes, 9 out of 33 cancers showed significant results. BLCA (bladder urothelial carcinoma), KICH (kidney chromophobe carcinoma), KIRC (kidney clear cell carcinoma), LGG (lower grade glioma), LUSC (lung squamous cell carcinoma), PAAD (pancreatic ductal adenocarcinoma), PRAD (prostate adenocarcinoma), STAD (stomach–gastric adenocarcinoma), THCA (thyroid papillary carcinoma), UCEC (uterine corpus endometroid carcinoma). (**B**) Kaplan–Meier curves for survival analysis show the association of the expression level of total micrRNome level with disease-free survival (DFS) and overall survival (OS) in pan-cancer. (**C**) Impact of gene mutation on the expression level of mature microRNAs. Y axis represents the average z score for all mature miRNAs. Comparisons between mutant (MUT) and wild-type samples (WT) are shown. The Mann–Whitney U test was used to test the significance.

A total of 717 paired tumor and normal samples were compared, and the average total microRNome levels were significantly higher in pan-cancer samples. Across different types of tumors, elevated levels were found in uterine, bladder, lung squamous, stomach, prostate, and kidney clear cell cancers. In contrast, lower expressions of mature miRNAs were observed in thyroid, pancreatic, and kidney chromophobe carcinoma samples compared to paired normal counterparts (Figure 8A). Higher levels of total microRNome were associated with poor survival times as demonstrated by Cox regression models (DFS: HR = 1.29, 95% CI = 1.19–1.39, *p* = 1.04 × 10^−10^, and OS: HR = 1.33, 95% CI = 1.24–1.43, *p* = 2.65 × 10^−15^) and Kaplan-Meier curves (Figure 8B). Genetic alterations in DICER1, DROSHA, PIWIL1, and GEMIN4 exhibited higher levels of expressed mature miRNAs (Figure 8C).

#### 3.2.5. Stratification Analysis for Genetic Alteration by Cancer Type

**Figure 9:**
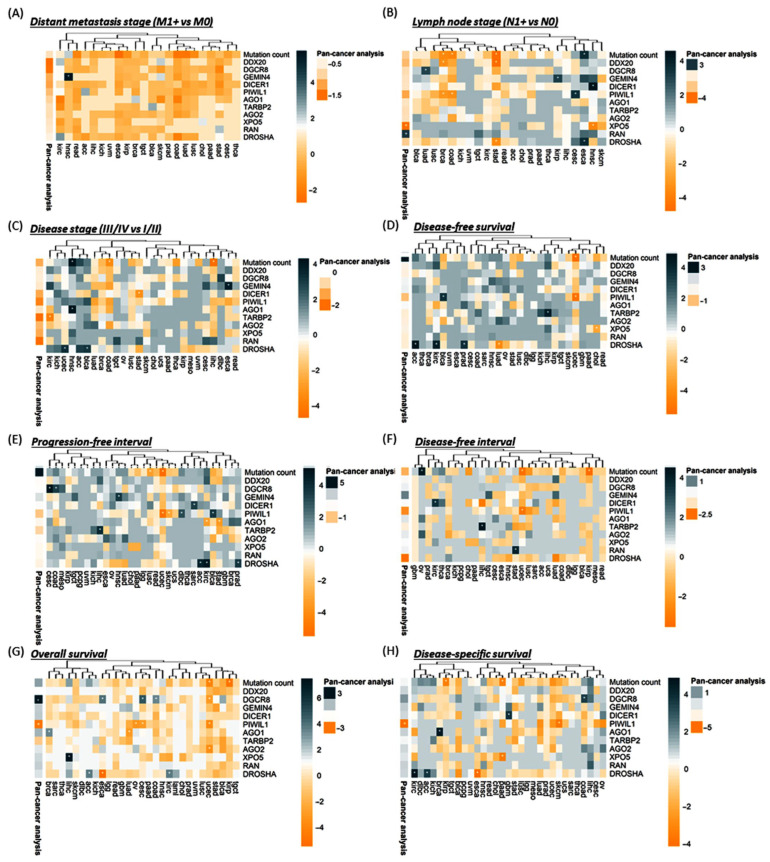
miRNA machinery gene mutations are potential prognosis markers in cancers. Heatmap representing the hierarchical clustering of the TCGA cancer types with the likelihood ratio *p*-values [Log(*p*-value)] obtained after logistic (for clinical parameters) and Cox (for outcome parameters) regression analyses between gene mutants/mutation count and clinical or survival parameters. The sign of Log(*p*-value) follows the sign of the regression beta-coefficient exponential. Annotation represents similar logistic regression in the pan-cancer study. * representing significant (*p* < 0.05) logistic/Cox regressions.

Data analysis per cancer type is depicted in Supplementary Tables S30 and S31. The per-cancer study showed that DROSHA mutation was impoverished in (a) lymph node metastatic in STAD tumors (*p* = 0.026) and COAD (*p* = 0.024), and (b) late (III/IV) stages in BLCA (*p* = 0.047) and UCEC (*p* = 0.036) cancers. DROSHA mutant was associated with worse survival outcomes in (a) ACC (DFS, *p* = 0.007; PFI, *p* = 0.012; OS, *p* = 0.024; DSS, *p* = 0.023), (b) KIRC (DFS, *p* = 0.011; PFI, *p* = 0.009; OS, *p* = 0.023; DSS, *p* = 0.008), (c) PRAD (DFS, *p* = 0.021; PFI, *p* = 0.026), and (d) LUAD (DFS, *p* = 0.016) tumors. However, it was associated with a good prognosis in ESCA (OS, *p* = 0.004; DSS, *p* = 0.016) (Figure 9). 

Next, PIWIL1 mutation was less frequent in (a) lymphatic disseminated tumors of BRCA (*p* = 0.043) and COAD (*p* = 0.037) and (b) late stages in ESCA (*p* = 0.021) and READ (*p* = 0.019) carcinoma. However, it has a positive association with the lymph node stage in CESC (*p* = 0.031). Its association with survival outcomes was more heterogeneous. Indeed, the mutant variant of PIWIL1 was more frequent in patients with a better survival prognosis than their counterparts with the wild-type version in (a) UCEC (DFS, *p* = 0.005; PFI, *p* = 0.005; DFI, *p* = 0.044; OS, *p* = 0.024), (b) SKCM (PFI, *p* = 0.048; DSS, *p* = 0.025), (c) CESC (OS, *p* = 0.046), and (d) OV (OS, *p* = 0.038) tumors, and associated with a worse outcome in (a) BLCA (DFS, *p* = 0.026; PFI, *p* = 0.024) and (b) DLBC (PFI, *p* = 0.042) tumors (Figure 9). 

DGCR8 mutant was associated with (a) the presence of lymphatic invasion in LUAD tumors (*p* = 0.048) and (b) late-stage III/IV in DLBC (*p* = 0.022) tumors. The mutation was impoverished in late stages (II/III/IV) in STAD tumors (*p* = 0.039). Except for UCEC tumors where the mutant variant was associated with a better prognosis (DFS, *p* = 0.044), DGCR8 mutation was globally found in patients with a worse outcome presenting COAD (DFS, *p* = 0.031, PFI, *p* = 0.031; OS, *p* = 0.031), CESC (DFS, *p* = 0.009; OS, *p* = 0.006) and ESCA (DFS, *p* = 0.021) cancer types (Figure 9). 

AGO1 mutants were positively associated with late-stage (III/IV) in HNSC cancers (*p* = 0.015) and a worse outcome in BRCA (DFS, *p* = 0.030, PFI, *p* = 0.011). However, the mutant variant was more frequent in LUAD (DFS, *p* = 0.044), PRAD (DFI, *p* = 0.002), KIRC (OS, *p* = 0.033), and STAD (*p* = 0.028) tumors showing good prognosis. TARBP2 mutant was more abundant in early-stage KIRC (I/II, *p* = 0.021) tumors and was positively associated with recurrence in LIHC (DFS, *p* = 0.012; PFI, *p* = 0.015; DFI, *p* = 0.011) patients (Figure 9). 

DICER1 mutant was significantly found in HNSC tumors with lymph node metastasis (*p* = 0.010) but was impoverished in late-stage (III/IV) STAD tumors (*p* = 0.033). DICER1 mutation was associated with a worse prognosis in SARC (PFI, *p* = 0.020), KIRC (DFI, *p* = 0.027), and GBM (DSS, *p* = 0.022) tumors. GEMIN4 mutation was associated with an aggressive phenotype described by (a) distant metastasis in HNSC (*p* = 0.003), (b) lymph node dissemination in KIRP (*p* = 0.044), (c) late stages in ESCA (III/IV, *p* = 0.020) and CESC (II/II/III, *p* = 0.016), and (d) PFI in HNSC (*p* = 0.024) tumors (Figure 9).

XPO5 mutant was absent in lymphatic invasive HNSC tumors (*p* = 0.022). It was associated with decreased survival in LIHC (OS, *p* = 0.001) and a better prognosis in CHOL (DFS, *p* = 0.044) and PAAD tumors (DSS, *p* = 0.029). DDX20 mutant was significantly enriched in lymphatic invasive BRCA (*p* = 0.035) and STAD (*p* = 0.008) tumors but was more frequent in late-stage IV HNSC tumors (*p* = 0.020). Regarding AGO2, gene mutation was associated with a longer survival in UCEC (OS, *p* = 0.032), whereas RAN mutant was associated with a shorter survival in STAD tumors (PFI, *p* = 0.020) (Figure 9). 

Finally, we assessed the prognosis ability of the mutation count in different cancer types. A higher number of mutations was associated with (a) lymph node dissemination in ESCA (*p* = 0.024) and (b) late stages in HNSC (*p* = 0.013) and KICH (*p* = 0.045) tumors. Inversely, a higher mutation count was more frequent in (a) non-disseminated BRCA (*p* = 0.027), COAD (*p* = 0.022), and STAD (*p* = 0.009) tumors, and (b) early-stage COAD (I/II, *p* = 0030), LIHC (I/II, *p* = 0.009), READ (I, *p* = 0.017), and ESCA (I/II/III, *p* = 0.023) tumors. Except in OV tumors where the mutation count was associated with shorter survival times (DFI, *p* = 0.014), a high number of miRNA machinery gene alterations was correlated with longer survival in (a) UCEC (DFS, *p* = 0.004; PFI, *p* = 0.004; DFI, *p* = 0.036; OS, *p* = 0.009), (b) KIRP (DFI, *p* = 0.028; OS, *p* = 0.019; DSS, *p* = 0.039), (c) LUSC (PFI, *p* = 0.039), and (d) PAAD (DFI, *p* = 0.033) tumors (Figure 9). 

Taken together, these results suggested that miRNA machinery gene alterations present a diverse prognosis prediction. However, the accumulation of mutation tends to be more associated with poor prognosis.

**Figure 10:**
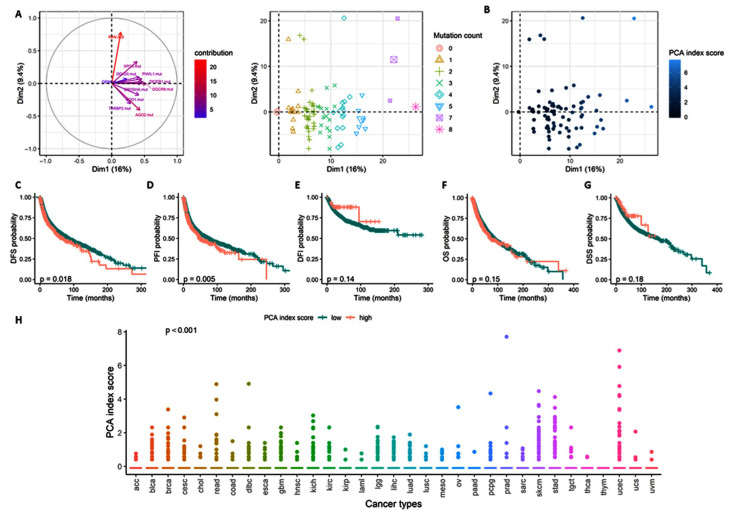
Principal Component Analysis using miRNA machinery gene mutations differentiated cancer samples. (**A**) Principal Component Analysis of the pan-cancer study according to the 11 gene mutants with the variable (left) and individual (right) plots, samples stratified according to the mutation count. (**B**) PCA individual plots are colored according to the PCA index score. (**C**–**G**) Kaplan–Meier curve estimated for the PCA index score according to DFS (**E**), PFI (**D**), DFI (**E**), OS (**F**), and DSS (**G**). Log test *p*-value. (**H**) PCA index score distribution across the cancer type. Kruskal test *p*-value. Significant *p*-value < 0.05. DFI, Disease-free interval; DFS, Disease-free survival; DSS, Disease-specific survival; OS, Overall survival; PFI, Progression-free interval.

As the accumulation of mutation presented a specific association with clinical and survival parameters, we performed PCA to determine how the panel of gene mutants differentiated cancer samples (Figure 10). Interestingly, the eleven mutants’ distribution could explain more than 25% of the heterogeneity observed across the tumor samples (Figure 10A), with a clear stratification following mutation count (Figure 10B). 

A PCA index score (see Method section for formula) was estimated and used as a risk marker score to study the association of the mutant panel with an aggressive phenotype and worse outcome (Figure 10C). The PCA index score did not present any significant differentiation with an aggressive phenotype, described with distant metastasis, lymph node dissemination, or disease stage (Supplementary Table S32). However, similarly to the accumulation of gene mutants, the PCA index score was associated with recurrence (Figure 10C–E) but not with survival (Figure 10F,G). Indeed, a high PCA index score presented a HR of 1.19 (95% CI = 1.03–1.38, *p* = 0.021) for DFS and of 1.20 (95% CI = 1.07–1.37, *p* = 0.006) for PFI, with a 5-year survival rate of 51% (45–56% vs. 57% [CI 95% = 55–59%], *p* = 0.018) and 47% (95% CI = 42–52% vs. 54% [95% CI = 52–55%], *p* = 0.005) for DFS and PFI, respectively (Supplementary Table S33). Finally, the PCA index scores show a different distribution across the cancer types (Figure 10H).

**Figure 11:**
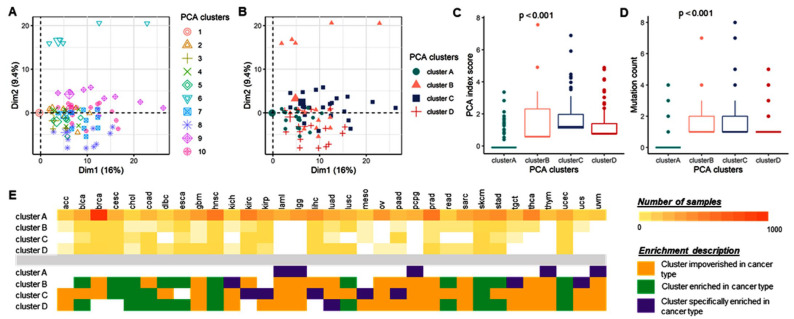
Four pan-cancer clusters of samples extract tumors with poor outcomes. (**A**,**B**) Principal Component Analysis of the pan-cancer study with the samples clustered in 10 distinct clusters (**A**), from the original hierarchical HCPC R function, and four clusters (**B**), following an optimized reduction. (**C**,**D**) PCA index score (**C**) and mutation count (**D**) distribution across the four clusters. Kruskal test *p*-value. (**E**) Contingency table of the four clusters across the cancer types (top table) with corresponding description of cancer type impoverishment (orange) or enrichment (green) in each cluster (table bottom). Purple highlights cancer types presenting a specific enrichment in one cluster.

Hence, we used hierarchical clustering on the PCA analysis to further stratify the pan-cancer samples based on the eleven-gene mutant panel. Ten clusters were first identified (Figure 11A). After a robust analysis of the clinical–survival parameter association, the ten clusters were classified into four optimized subgroups (Figure 11B), with a significant distribution of the PCA index score (*p* = 0, Figure 11C) and accumulation of mutations (*p* = 0, Figure 11D). Cluster C presented the highest number of mutations, whereas cluster A bore the smallest. The four clusters showed a significant distribution across the cancer type (*p* = 5.16 × 10^−26^), with cluster A specifically enriched in LAML, LGG, PCPG, THYM, and UVM cancer types, cluster B specifically characterized by KICH, TGCT, and UCS cancer types, cluster C defined by KIRC, KIRP, LIHC, MESO, and PAAD, and finally cluster D by LUAD cancer type. 

Compared to cluster A, cluster B was enriched in lymph node disseminated samples (OR = 1.55, 95%CI = 0.94–2.55, *p* = 0.084), whereas cluster C was impoverished of lymphatic invasive samples (OR = 0.43, 95%CI = 0.23–0.81, *p* = 0.008). Indeed, with a Chi^2^ test assessing a significant distribution of the different lymph node stages across the clusters, cluster C was differentially enriched in lymph disseminated samples compared to cluster A (*p* = 0.010) and cluster B (*p* = 0.002), as well as cluster D compared to cluster B (*p* = 0.033). Cluster D was characterized by early-stage (stage I) samples (OR = 0.65, 95% CI = 0.45–0.94) (Figure 11E).

**Figure 12:**
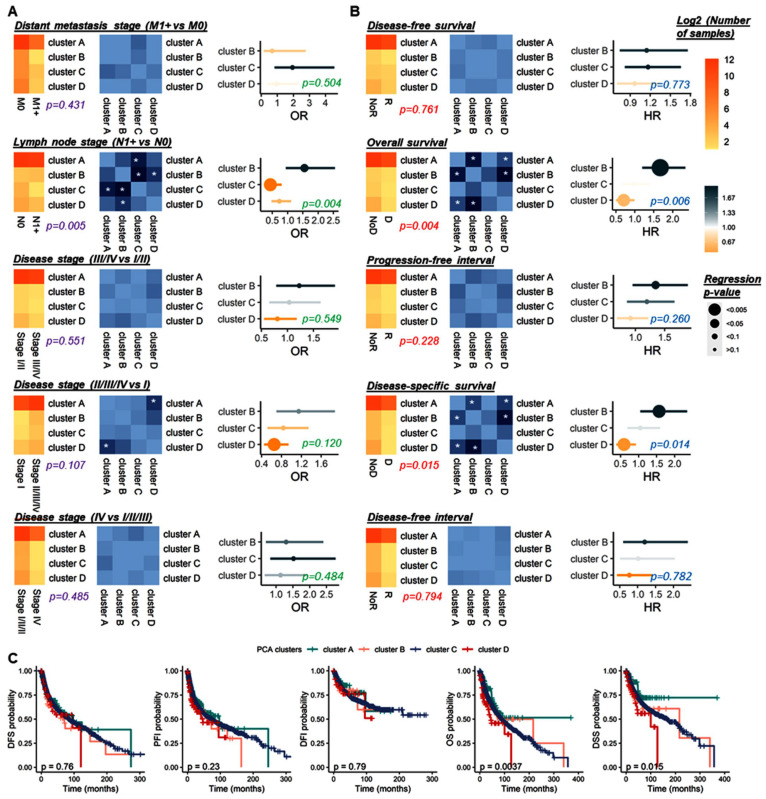
Clinical and survival analyses of the four pan-cancer clusters. (**A**) Association with distant metastasis stage, lymph node stage, disease stage (III/IV vs. I/II–II/III/IV vs. I–IV vs. I/II/III). (**B**) Association with disease-free survival, progression-free interval, disease-free interval, overall survival, and disease-specific survival. Details on the distribution of samples according to the marker variates (left), with the estimation of Chi2 test *p*-value (**A**) and log-rank test *p*-value (**B**), the corresponding *p*-values are comparing cluster differentiation (middle), and the logistic (**A**) or Cox (**B**) regression analyses. White * within the heatmap box for significant *p*-values. (**C**) Kaplan–Meier curve estimated for the four PCA pan-cancer according to DFS, PF I, DFI, OS, and DSS. Log-rank test *p*-value. Significant *p*-value < 0.05. DFI, Disease-free interval; DFS, Disease-free survival; DSS, Disease-specific survival; OS, Overall survival; PFI, Progression-free interval; D, Death; NoD, No death; R, Recurrence; NoR, Recurrence.

The regression was confirmed with a Chi^2^ test (*p* = 0.028). Furthermore, cluster B presented the worst outcome (PFI, HR = 1.34, 95% CI = 0.95–1.89, *p* = 0.092; OS, HR = 1.67, 95% CI = 1.20–2.33, *p* = 0.003; DSS, HR = 1.57, 95% CI = 1.05–2.35, *p* = 0.029) and cluster D was associated with a good prognosis (OS, HR = 0.71, 95% CI = 0.50–0.99, *p* = 0.045; DSS, HR = 0.59, 95% CI = 0.38–0.92, *p* = 0.021), compared to cluster A (Figure 12A). Indeed, from the less to the more aggressive, clusters D, C, A, and B presented a DSS 5-year survival rate of 75% (95% CI = 62–84%), 69% (95% CI = 55–80%), 68% (95% CI = 67–70%), and 56% (95% CI = 39–70%), respectively (*p* = 0.015). Similar results were observed for overall survival (Figure 12B). Cluster B presents a significant outcome than cluster A (OS, *p* = 0.002); DSS, *p* = 0.027), and cluster D (OS, *p* = 5.04 × 10^−4^; DSS, *p* = 0.001), as well as cluster A compared to cluster D (OS, *p* = 0.048; DSS, *p* = 0.022) (Figure 12C).

#### 3.2.6. Stratification Analysis for Total miRNome by Cancer Type

**Figure 13:**
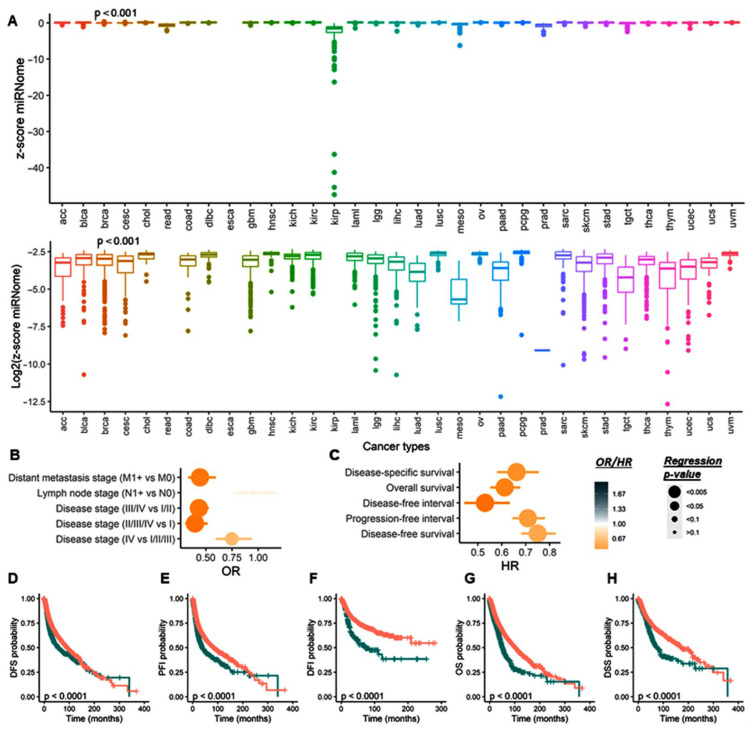
The total miRNome score is an independent marker for late stages and worse outcomes in cancer. (**A**) Box plots representing the distribution of the z-score miRNome across the samples without further transformation (top) and log2 transformation (bottom). (**B**,**C**) Logistic and Cox regression analysis to predict the impact of the z-score total miRNome on clinical (**B**) and survival (**C**) parameters. Significant *p*-value < 0.05. (**D**–**H**) Kaplan–Meier curve estimated for the z-score total miRNome according to DFS (**D**), PFI (**E**), DFI (**F**), OS (**G**), and DSS (**H**). Log-rank test *p*-value. Significant *p*-value < 0.05. DFI, Disease-free interval; DFS, Disease-free survival; DSS, Disease-specific survival; OS, Overall survival; PFI, Progression-free interval.

The total miRNome presented a heterogenous distribution across the cancer types, with a lower expression in KIRP, MESO, and TCGT cancer types (*p* = 0) (Figure 13A). We investigated the prognosis propensity of the total miRNome marker in a pan-cancer study. We observed that a high score was associated with (a) an absence of distant metastasis (OR = 0.44, 95% CI = 0.31–0.61, *p* = 1.99 × 10^−4^) and (b) early stages (I/II, OR = 0.46, 95% CI = 0.37–0.58, *p* = 1.16 × 10^−12^; I/II/III, OR = 0.74, 95% CI = 0.58–0.95, *p* = 0.026) (Figure 13B). Furthermore, a high total miRNome score was associated with a good prognosis, characterized by a longer DFS (HR = 0.75, 95% CI = 0.67–0.84, *p* = 1.93 × 10^−6^) with a 5-year survival rate of 61% (95% CI = 59–53% vs. 50% [95% CI = 46–54%], *p* = 0), PFI (HR = 0.61, 95% CI = 0.63–0.70, *p* = 1.32 × 10^−11^) with the 5-year survival rate of 57% (95% CI = 55–59% vs. 36% [95% CI = 30–42%], *p* = 0), by DFI (HR = 0.45, 95% CI = 0.35–0.57, *p* = 1.74 × 10^−9^) with the 5-year survival rate of 74% (95% CI = 72–76% vs. 48% [95% CI = 38–58%], *p* = 6.67 × 10^−12^), by OS (HR = 0.53, 95% CI = 0.47–0.60, *p* = 5.96 × 10^−21^) with the 5-year survival rate of 65% (95% CI = 63–66% vs. 41% [95% CI = 36–46%], *p* = 0), and DSS (HR = 0.60, 95% CI = 0.50–0.71, *p* = 2.51 × 10^−8^) with the 5-year survival rate of 73% (95% CI = 71–75% vs. 53% [95% CI = 46–59%], *p* = 1.78 × 10^−9^) (Figure 13C–H), Supplementary Table S34.

After adjusting for age, gender, and race, the total miRNome score was defined as an independent marker for the prediction of disease stage (III/IV vs. I/II, OR = 0.28, OR-CI 95% = 0.18–0.32, *p* = 2.20 × 10^−16^; II/III/IV vs. I, OR = 0.26, 95% CI = 0.17–0.38, *p* = 5.86 × 10^−12^; IV vs. I/II/III, OR = 0.73, 95% CI = 0.54–0.98, *p* = 0.038), recurrence (DFS, HR = 0.70, 95% CI = 0.63-0.77, *p* = 3.58 × 10^−12^; PFI, HR = 0.66, 95% CI = 0.60–0.74, *p* = 5.48 × 10^−15^; DFI, HR = 0.43, HR-CI 95% = 0.36–0.52, *p* = 6.20 × 10^−19^), and survival (OS, HR = 0.57, 95% CI = 0.51–0.63, *p* = 6.76 × 10^−25^; DSS, HR = 0.65, 95% CI = 0.0.53–0.75, *p* = 2.92 × 10^−9^).

Interestingly, in the pan-cancer study, the association between the total miRNome score and the cancer aggressive phenotype and outcome partially depended on the accumulation of miRNA machinery gene epigenomic alteration or specific mutations. Indeed, after a multivariate analysis with the mutation count or a single mutation status, the total miRNome score continued to be significantly associated with clinical and survival parameters but with a weaker *p*-value. Furthermore, we observed a different behavior of the total miRNome score across the cancer types, Supplementary Tables S34–S36.

**Figure 14:**
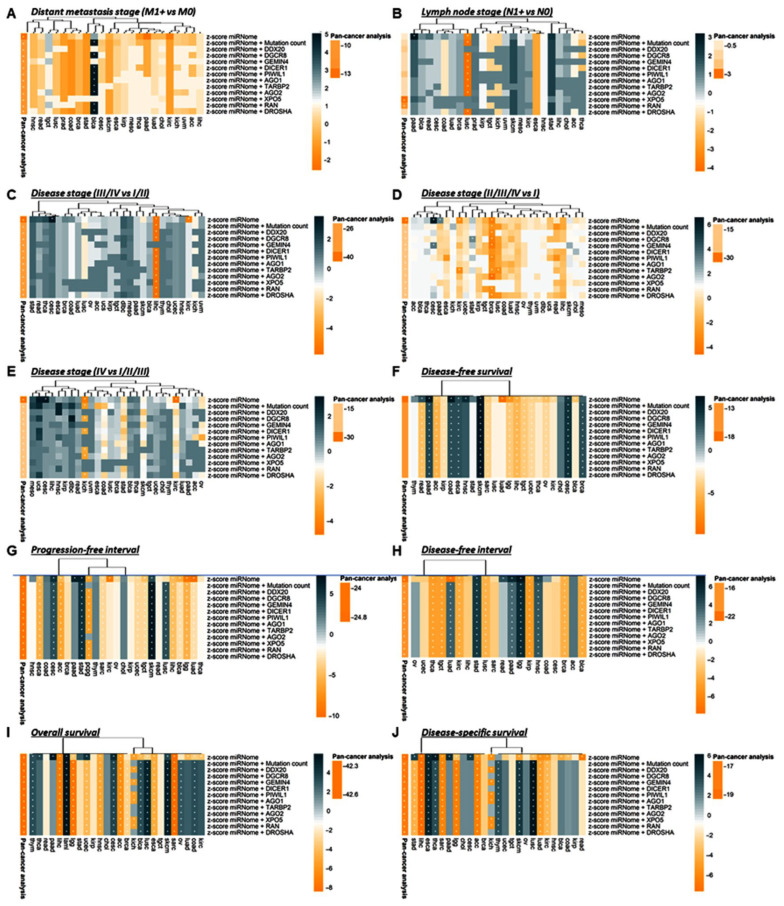
The total miRNome marker is dependent on the miRNA biogenesis epigenomic alterations to be a potential prognosis marker in cancer. Heatmap representing the hierarchical clustering of the TCGA cancer types with the likelihood ratio *p*-values [Log(*p*-value)] obtained after univariate and multivariate logistic (for clinical parameters) and Cox (for outcome parameters) regression analyses between z-score miRNome count and clinical or survival parameters, adjusted to gene mutants. The sign of Log(*p*-value) follows the sign of the regression beta-coefficient exponential. Annotation represents similar logistic regression in the pan-cancer study.

A low total miRNome was still associated with late disease stages in KIRC (III/IV, *p* = 0.005), dependently on any single gene mutant or the accumulation of mutations, in BRCA (II/III/IV, *p* = 0.016), dependent on DICER1 and AGO1 mutation status, and in LUSC (II/III/IV, *p* = 0.046), independently of TARBP2, LIHC (III/IV, *p* = 0.003) and KICH (IV, *p* = 0.014), both independently of the miRNA machinery gene alteration. Furthermore, a low total miRNome was also significantly found in lymph node disseminated LUSC cancer type (*p* = 0.028), independently of the miRNA machinery genes (Figure 14).

A high total miRNome score was more frequent in lymph node metastatic PAAD (*p* = 0.041) and late stages CESC (III/IV, *p* = 0.030) tumors, controlled by the miRNA machinery gene alterations. Interestingly, after adjusting the miRNA machinery gene alteration, a high score is also independently associated with distant metastasis in BLCA tumors (Figure 14).

A low total miRNome score was associated with a recurrence and or survival in ACC (DFS, *p* = 0.007; PFI, *p* = 0.006; OS, *p* = 0.003; DSS, *p* = 0.003), BLCA (DFS, *p* = 0.013; PFI, *p* = 0.003; DFI, *p* = 0.023; OS, *p* = 0.013; DSS, *p* = 0.015), BRCA (DFI, *p* = 0.021), KICH (DFS, *p* = 0.019; PFI, *p* = 0.050; OS, *p* = 0.004; DSS, *p* = 0.004), KIRC (DFS, *p* = 0.031; PFI = 1.13 × 10^−4^; DFI, *p* = 0.011; OS, *p* = 0.026; DSS, *p* = 0.005), KIRP (DFS, *p* = 0.046; PFI, *p* = 0.008), LIHC (DFS, *p* = 0.003; PFI, *p* = 0.007; OS, *p* = 7.29 × 10^−4^; DSS, *p* = 7.29 × 10^−4^), LUAD (DFS, *p* = 5.34 × 10^−5^; DFI, *p* = 3.65 × 10^−5^; OS, *p* = 0.047; DSS, *p* = 0.003), LUSC (OS, *p* = 0.006; DSS, *p* = 0.030), OV (DFS, *p* = 0.008; PFI, *p* = 3.74 × 10^−5^), PCPG (PFI, *p* = 0.001; DSS, *p* = 0.006), SARC (DFS, *p* = 0.016; PFI, *p* = 0.021; OS, *p* = 0.2.17 × 10^−4^; DSS, *p* = 0.002), TGCT (DFS, *p* = 0.015; PFI, *p* = 0.010; DFI, *p* = 0.009), and UVM (OS, *p* = 0.047) (Figure 14).

Inversely, a high total miRNome score was associated with a worse prognosis in CESC (DFS, *p* = 0.015; PFI, *p* = 0.005; OS, *p* = 0.008; DSS, *p* = 0.015), LAML (OS, *p* = 0.002), PAAD (DFS, *p* = 0.002; PFI, *p* = 0.002; DFI, *p* = 0.003; OS, *p* = 0.004; DSS, *p* = 0.008), PRAD (DFS, *p* = 0.018; PFI, *p* = 0.041; DFI, *p* = 0.036; DSS, *p* = 0.041), and UCS (DFS, *p* = 0.007; PFI, *p* = 0.001; DFI, *p* = 0.025). Their dependence on the miRNA machinery gene alteration was diverse with the specific signature of mutant controlling the total miRNome score to predict patient outcome (Figure 14).

## 4. Discussion

Authors should discuss the results and how they can be interpreted from the perspective of previous studies and of the working hypotheses. The findings and their implications should be discussed in the broadest context possible. Future research directions may also be highlighted. Insights into core miRNA biogenesis mutations reveal a link between cancer development and progression [18,19,20,21,87,88]. Knockouts or mutations of core miRNA factors illustrate their requirement for normal development, differentiation, and cell maturation in most tissues [89]. Phenotypes in cancer may stem from concomitant depletion of multiple functional miRNAs or failure to maintain subclasses of active miRNAs. Here, we addressed a diverse array of mutations in different core miRNA machinery genes. A meta-analysis of 22 gene variants was conducted. Pooling the data from 45 articles, our results reveal the association of *DROSHA* (A > G; rs10719), *DGCR8* (G > A; rs417309), *RAN* (A > G; rs3803012), and *GEMIN3* (C > A; rs197414) with a 19–93% higher susceptibility to overall cancer. 

The DROSHA–DGCR8 microprocessor complex could mediate microRNA maturation by precise cleavage of the stem-loops that are embedded in primary transcripts in a complementary way, and neither is sufficient to process alone [48]. Based on the HaploReg v4.1 online tool for variant annotations (https://pubs.broadinstitute.org/mammals/haploreg/haploreg.php), *DROSHA* (A > G; rs10719) variant can alter HMG box protein 1 (hbp1) motif sequence, a transcription factor, and a potent cell cycle inhibitor in normal and cancer cells [90]. DROSHA* rs10719 is located in the 3′UTR region. Based on bioinformatics website prediction (https://snpinfo.niehs.nih.gov/), this point mutation was found to disrupt the binding site of miR-27b and lead to overexpression of *DROSHA* gene, which in turn was found to facilitate proliferation and inhibit apoptosis of cancer cells [91,92]. Our findings indicate that the rs417309 SNP of *DGCR8* might facilitate oncogenesis. The *DGCR8* (G > A; rs417309) variant was the most extensively investigated polymorphism in this gene, and the rs417309-A allele was strongly associated with increased cancer susceptibility. This SNP also exists in the 3′UTR region at the binding sites of the *miR-106b* and *miR-579* genes. Gene overexpression was associated with the risk allele rs417309*A, thus facilitating cancer development [48]. The HaploReg website showed that the SNP could modify the binding sites for Doublesex and Mab-3 Related Transcription Factor 1 (DMRT1) and 2 (DMRT2) and zinc finger-like DNA-binding domain, which are associated with testicular germ cell cancer [93]. *RAN* is essential for the translocation of pre-miRNAs from the nucleus to the cytoplasm through the nuclear pore complex in a GTP-dependent manner [94]. Nucleotide sequence change in *RAN* (A > G; rs3803012) leads to modification of the binding site of the transcription factor NANOG, which is associated with the differentiation of pluripotent stem cells and various types of cancer [95]. Studies also proposed that the RAN rs3803012*G allele might affect miRNA-199a-3p targeting and decrease RAN mRNA expression in cancer cells, which may affect miRNA biosynthesis [68]. The *GEMIN3* (C > A; rs197414) variant is located in the CTCF transcription factor binding site, a transcription factor linked to chromatin remodeling and genome topology [96]. Taken together, these findings are in line with our meta-analysis results and support the role of miRNA machinery gene mutation in human tumors. Apart from *TARBP2* and *RAN*, our trial sequential analysis results showed that the cumulative evidence for miRNA gene machinery variants was inadequate, and additional primary allelic discrimination studies for the other nine genes are warranted to validate the outcomes in various types of cancers.

To identify the role of miRNA machinery gene mutation in association with clinical and pathological parameters, 33 pan-cancer studies in the TCGA were analyzed. Average mutation rates varied, with the most common being *DICER1* (accounting for 1.87% of gene mutations in the sample population), followed by *PIWIL1* and *DROSHA* gene mutations (1.54% and 1.36%, respectively). Higher mutation rates of machinery genes were evident in gastrointestinal tumors (esophagus, gastric, gall bladder, and colorectal), uterine cancer, melanoma, and lung carcinoma. Consistently, genetic variants of *DICER1* have been found to modulate the risk of gastric cancer [97], endocrine tumors [98], ovarian cancer [99], testicular germ-cell tumors [98], and neurofibromatosis [100], and have been found to be associated with head and neck cancer recurrence [98] and ovarian cancer prognosis [98]. Gene mutations in *Piwi*-interacting RNA pathway genes confer susceptibility to esophageal cancer [85], renal cell carcinoma [51], hepatocellular cancer [68], and glioblastoma progression [101]. Moreover, multiple SNPs in *DROSHA* mutations are common in diverse human malignancies, including Wilms tumor [102], esophageal cancer [85], and breast cancer [48]. 

Another finding in the TCGA was that seven miRNA-related genes were associated with poor prognostic parameters: the nuclear *DROSHA* and *DGCR8* complex, the cytoplasmic *DICER1* and *TARBP2* enzymes, and miRISC-related components such as *AGO1*, *AGO2*, and *PIWIL1*. Patients with *DGCR8*, *DROSHA*, and *TARBP2* mutations were twice as likely to present with lymph node metastasis. *DGCR8* was associated with higher susceptibility to distant metastasis. While *AGO1* and *DGCR8* mutations conferred around 2.5 times higher risk of advanced tumor size, *DROSHA* mutations were 2.5 times more likely to present with poorly differentiated tumors at the time of diagnosis. Multivariate Cox regression analysis revealed that the *PIWIL1* mutant was associated with better survival. In contrast, *DGCR8* mutation carriers presented with shorter five-year survival. It remains to be defined whether these SNPs affect the expression and function of miRNA machinery genes directly or are merely tagging SNPs. 

While the canonical pathway generates the large majority of miRNA species, it is worth noting that emerging studies reveal a growing number of Drosha-independent and Dicer-independent alternative mechanisms that generate functional microRNAs and contribute to distinct phenotypes among core microRNA biogenesis mutants. Interpretation of the consequences of these mechanisms is challenging, given the paucity of currently available publications and public databases. A better understanding of these mechanisms will lead to better prediction of the health effects of each perturbation. Overall, we provide the most comprehensive bioinformatics analysis and meta-analysis on genetic variations in 11 miRNA machinery pathway genes supporting their association with the risk and prognosis of 33 different types of cancer. Nonetheless, it should be noted that although our study has one of the largest collections of pan-cancer patients, it differs from the recent interesting bioinformatic study carried out by Galka-Marciniak et al. [103] in running a systematic review and metanalysis for all published related studies in the specified period of the analysis. A limited number of studies per SNP constrains the ability of polygenic assessment and decreases the confidence of having sufficient power for each cancer subtype analysis. Future independent replication studies and functional characterizations are needed to explore the potential gene-gene and gene-environment interactions.

## 5. Conclusions

Our results suggest that genetic polymorphisms of the miRNA-machinery genes may affect cancer susceptibility and progression. Genetic mapping and functional characterizations are warranted to identify causal SNPs and their underlying molecular mechanisms. Defining the landscape of perturbation with a functional consequence may aid in risk stratification for high-risk populations and facilitate the development of future treatments.

## Data Availability

All data used were included in the manuscript, and additional results were available in the Supplementary Materials.

## Supplementary Materials

The following supporting information can be downloaded at: https://www.mdpi.com/article/10.3390/cancers15020338/s1, Table S1: Characteristics and selection criteria of single nucleotide Polymorphisms in microRNA machinery genes; Table S2: Scale for methodological quality assessment; Table S3: Quality score assessment for included studies; Table S4: Characteristics of the included studies., Table S5: Distribution of samples according to the type of cancer, Table S6: Meta-analysis of the association between *DROSHA* (A > G; rs10719) variant and cancer risk.; Table S7: Meta-analysis of the association between *DROSHA* (G > C; rs6877842) variant and cancer risk.; Table S8: Meta-analysis of the association between *DGCR8* (G > A; rs3757) variant and cancer risk; Table S9: Meta-analysis of the association between *DGCR8* (G > A; rs417309) variant and cancer risk; Table S10: Meta-analysis of the association between *DGCR8* (T > G; rs1640299) variant and cancer risk; Table S11: Meta-analysis of the association between *XPO5* (T > G; rs11077) variant and cancer risk; Table S12: Meta-analysis of the association between *RAN* (C > T; rs14035) variant and cancer risk; Table S13: Meta-analysis of the association between *RAN* (A > G; rs3803012) variant and cancer risk; Table S14: Meta-analysis of the association between *DICER* (A > T; rs13078) variant and cancer risk; Table S15: Meta-analysis of the association between *DICER* (T > C; rs1057035) variant and cancer risk; Table S16: Meta-analysis of the association between *DICER* (A > G; rs3742330) variant and cancer risk; Table S17: Meta-analysis of the association between *TARBP2* (G > A; rs784567) variant and cancer risk; Table S18: Meta-analysis of the association between *AGO1* (A > G; rs595961) variant and cancer risk; Table S19: Meta-analysis of the association between *AGO1* (G > A; rs636832) variant and cancer risk; Table S20: Meta-analysis of the association between *AGO2* (C > A; rs4961280) variant and cancer risk; Table S21: Meta-analysis of the association between *GEMIN3* (T > C; rs197412) variant and cancer risk; Table S22: Meta-analysis of the association between *GEMIN3* (C > A; rs197414) variant and cancer risk; Table S23: Meta-analysis of the association between *GEMIN4* (G > A; rs7813) variant and cancer risk; Table S24: Meta-analysis of the association between *GEMIN4* (G > C; rs2740348) variant and cancer risk; Table S25: Meta-analysis of the association between *GEMIN4* (C > T; rs3744741) variant and cancer risk; Table S26: Meta-analysis of the association between *PIWIL1* rs1106042 G > A variant and cancer risk; Table S27: Meta-analysis of the association between *PIWIL1* rs10773771 C > T variant and cancer risk; Table S28: Overall survival analysis for TCGA pan-cancer dataset, Table S29: Disease-specific survival analysis for TCGA pan-cancer dataset; Table S30: Analysis of mutation count and gene mutants association with clinical parameters—PER-cancer study; Table S31: Analysis of mutation count and gene mutants association with survival parameters—PER-cancer study; Table S32: Logistic and cox regression analysis with PCA Index score; Table S33: Analysis of PCA clusters association with clinical and survival parameters; Table S34: Logistic and cox regression analysis with z-score miRNome; Table S35: Logistic and cox regression analysis with z-score miRNome in univariate and multivariate analyses (adjusted to a single gene mutant or the mutation count)—PAN cancer analysis; Table S36: Logistic and cox regression analysis with z-score miRNome in univariate and multivariate analyses (adjusted to a single gene mutant or the mutation count)—PER cancer analysis.
