# Supplementary material for: Pan-Cancer Study on Variants of Canonical miRNA Biogenesis Pathway Components: A Pooled Analysis
Source: Cancers (Basel). 2023 Jan 4;15(2):338. doi: 10.3390/cancers15020338 (PMC9856462; doi:10.3390/cancers15020338)
Supplement: Supplementary file 1 [file cancers-15-00338-s001.zip › Supplementary Tables_data set.pdf]

Table of Contents

Supplementary Table	Title	Pages
**Variant selection**		
S1	Characteristics and selection criteria of single nucleotide Polymorphisms in microRNA machinery genes.	2
**Quality assessment**		
S2	Scale for methodological quality assessment.	3
S3	Quality score assessment for included studies.	4
**Study characteristics**		
S4	Characteristics of the included studies.	7
S5	Distribution of samples according to the type of cancer.	11
**Pooled analysis**		
S6	Meta-analysis of the association between DROSHA (A > G; rs10719) variant and cancer risk.	12
S7	Meta-analysis of the association between DROSHA (G > C; rs6877842) variant and cancer risk.	14
S8	Meta-analysis of the association between DGCR8 (G > A; rs3757) variant and cancer risk.	16
S9	Meta-analysis of the association between DGCR8 (G > A; rs417309) variant and cancer risk.	18
S10	Meta-analysis of the association between DGCR8 (T > G; rs1640299) variant and cancer risk.	20
S11	Meta-analysis of the association between XPO5 (T > G; rs11077) variant and cancer risk.	22
S12	Meta-analysis of the association between RAN (C > T; rs14035) variant and cancer risk.	25
S13	Meta-analysis of the association between RAN (A > G; rs3803012) variant and cancer risk.	28
S14	Meta-analysis of the association between DICER (A > T; rs13078) variant and cancer risk.	30
S15	Meta-analysis of the association between DICER (T > C; rs1057035) variant and cancer risk.	32
S16	Meta-analysis of the association between DICER (A > G; rs3742330) variant and cancer risk.	34
S17	Meta-analysis of the association between TARBP2 (G > A; rs784567) variant and cancer risk.	37
S18	Meta-analysis of the association between AGO1 (A > G; rs595961) variant and cancer risk.	39
S19	Meta-analysis of the association between AGO1 (G > A; rs636832) variant and cancer risk.	41
S20	Meta-analysis of the association between AGO2 (C > A; rs4961280) variant and cancer risk.	43
S21	Meta-analysis of the association between GEMIN3 (T > C; rs197412) variant and cancer risk.	45
S22	Meta-analysis of the association between GEMIN3 (C > A; rs197414) variant and cancer risk.	47
S23	Meta-analysis of the association between GEMIN4 (G > A; rs7813) variant and cancer risk.	49
S24	Meta-analysis of the association between GEMIN4 (G > C; rs2740348) variant and cancer risk.	51
S25	Meta-analysis of the association between GEMIN4 (C > T; rs3744741) variant and cancer risk.	54
S26	Meta-analysis of the association between PIWIL1 rs1106042 G > A variant and cancer risk.	57
S27	Meta-analysis of the association between PIWIL1 rs10773771 C > T variant and cancer risk.	59
**TCGA results**		
S28	Overall survival analysis for TCGA pan-cancer dataset .	61
S29	Disease-specific survival analysis for TCGA pan-cancer dataset.	61

Supplementary Tables S30 to S36 is in a separate excel file.

Supplementary Table S1. Characteristics and selection criteria of single nucleotide Polymorphisms in microRNA machinery genes.

Gene	SNP ID	Alleles	MAF	Ancestral	Location	dbSNP	Chr	Position	Function	Reason for Selection	Total Citations	Citations for cancer
DROSHA	rs10719	A/G/T	0.48 (A)	A	31401340	A>G / A>T	5	3' UTR	3' UTR variant	Bibliographic	50	10
	rs6877842	G/C	0.14 (C)	G	31532531	G>C	5	Intron	Intron variant	Bibliographic	25	8
	rs642321	T/C	0.32 (T)	T	31400896	T>C	5	3' UTR	3' UTR variant	Bibliographic	17	3
	rs644236	T/C	0.47 (C)	C	31409008	T>C	5	Intron	Non-coding transcript exon variant	Bibliographic	7	1
	rs2287584	T/C	0.45 (C)	C	31422900	T>C	5	Synonymous	Synonymous variant	Bibliographic	4	1
	rs2291109	A/G/T	0.06 (A)	T	31532322	A>G / A>T	5	5' UTR	5' UTR variant	Bibliographic	2	1
	rs3805500	G/A	0.50 (G)	G	31462870	G>A	5	Intron	Intron variant	Bibliographic	6	1
	rs4867329	A/C	0.37 (C)	A	31435520	A>C	5	Intron	Intron variant	Bibliographic	6	1
DICER1	rs13078	A/T	0.09 (A)	T	95090410	A>T	14	3' UTR	3' UTR variant	Bibliographic	39	9
	rs1057035	T/C	0.17 (C)	T	95087805	T>C	14	3' UTR	3' UTR variant	Bibliographic	35	11
	rs3742330	A/G	0.14 (G)	A	95087025	A>G	14	3' UTR	3' UTR variant	Bibliographic	58	16
	rs12323635	C/T	0.49 (T)	T	95159374	C>T	14	Intron	Intron variant	Bibliographic	8	1
XPO5	rs11077	T/G	0.40 (G)	G	43523209	T>G	6	3' UTR	3' UTR variant	Bibliographic	57	13
	rs1106841	A/C	0.39 (C)	C	43528924	A>C	6	p.Arg893=	Splice region variant	Bibliographic	3	1
	rs2257082	G/A	0.32 (A)	G	43524840	G>A	6	p.Tyr1101=	Synonymous variant	Bibliographic	10	3
	rs11544382	A/G	NA	A	43524604	A>G	6	p.Met1115Thr	Missense variant	Bibliographic	6	1
	rs34324334	C/T	0.04 (T)	C	43567281	C>T	6	p.Ser241Asn	Missense variant	Bibliographic	4	1
RAN	rs14035	C/T	0.27 (T)	T	130876696	C>T	12	3'UTR	3' UTR variant	Bibliographic	45	12
	rs3803012	A/C/G	0.01 (G)	A	130876170	A>C / A>G	12	3'UTR	3' UTR variant	Bibliographic	11	5
	rs3809142	C/G/T	0.12 (T)	C	130871001	C>G / C>T	12	NA	Upstream variant	Bibliographic	1	1
	rs7301722	C/A	0.42	A	130871510	C>A	12	NA	Upstream variant	Bibliographic	1	1
	rs7132224	A/G	0.30 (G)	G	130871501	A>G	12	NA	Upstream variant	Bibliographic	1	1
	rs56109543	C/T	0.17 (T)	C	130870520	C>T	12	NA	Upstream variant	Bibliographic	1	1
DGCR8	rs3757	G/A	0.18 (A)	G	20111808	G>A	22	3' UTR	3' UTR variant	Bibliographic	31	7
	rs417309	G/A	0.04 (A)	G	20111021	G>A	22	3' UTR	3' UTR variant	Bibliographic	21	7
	rs1640299	T/G	0.38 (G)	G	20110836	T>G	22	3' UTR	3' UTR variant	Bibliographic	23	8
	rs720012	G/A	0.22 (A)	G	20111059	G>A	22	3' UTR	3' UTR variant	Bibliographic	11	1
	rs720014	T/C	0.18 (C)	T	20111359	T>C	22	3' UTR	3' UTR variant	Bibliographic	8	3
	rs1558496	T/C	0.14 (C)	C	20084214	T>C	22	Intron	Intron variant	Bibliographic	2	1
	rs11089328	A/G	0.48 (G)	G	20092080	A>G	22	Intron	Intron variant	Bibliographic	2	1
	rs9605062	T/C	0.25 (C)	C	20102357	T>C	22	Intron	Intron variant	Bibliographic	2	1
	rs9606250	A/T	0.14 (T)	T	20102669	A>T	22	Intron	Intron variant	Bibliographic	3	1
	rs9606248	A/G	0.09 (G)	G	20100016	A>G	22	Intron	Intron variant	Bibliographic	4	1
GEMIN3 (DDX20)	rs197388	A/T	0.24 (T)	T	111754860	A>T	1	5' UTR	Upstream variant	Bibliographic	11	3
	rs197412	T/C	0.47 (C)	C	111766331	T>C	1	p.Ile636Thr	Missense variant	Bibliographic	43	9
	rs197414	C/A/T	0.17 (A)	C	111766501	C>A / C>T	1	p.Arg693Ser	Missense variant	Bibliographic	32	5
GEMIN4	rs7813	G/A/C	0.29 (G)	G	744946	G>A / G>C	17	p.Arg1033Cys	Missense variant	Bibliographic	55	14
	rs2740348	G/C/T	0.11 (G)	C	746695	G>C / G>T	17	p.Gln450Glu	Missense variant	Bibliographic	43	11
	rs2740349	C/G/T	0.11 (C)	T	745258	C>G / C>T	17	p.Asp929Asn	Missense variant	Bibliographic	11	1
	rs910924	G/A	0.16 (A)	G	752680	G>A	17	5' UTR	5' UTR variant	Bibliographic	16	3
	rs910925	G/C	0.29 (G)	G	746307	G>C	17	p.Ala579Val	Missense variant	Bibliographic	19	4
	rs1045481	G/A/C	0.16 (A)	G	744917	G>A / G>C	17	p.Ile1042=	Missense variant	Bibliographic	5	0
	rs1045491	C/T	0.16 (T)	C	744748	C>T	17	3' UTR	3' UTR variant	Bibliographic	6	1
	rs1062923	A/G	0.07 (G)	A	745827	A>G	17	p.Ile739Thr	Missense variant	Bibliographic	23	3
	rs3087833	G/A	0.16 (A)	G	754490	G>A	17	Promoter	Non-coding transcript exon variant	Bibliographic	2	0
	rs3744741	C/T	0.28 (T)	C	745992	C>T	17	p.Arg684Gln	Missense variant	Bibliographic	28	9
	rs4968104	T/A	0.16 (A)	T	746265	T>A	17	p.Glu593Val	Missense variant	Bibliographic	30	4
AGO1	rs636832	G/A	0.37 (A)	A	35897874	G>A	1	Intron	Intron variant	Bibliographic	35	6
	rs595055	T/C	0.49 (T)	C	35914532	T>C	1	Intron	Intron variant	Bibliographic	7	1
	rs595961	A/G	0.45 (A)	G	35902179	A>G	1	Intron	Intron variant	Bibliographic	26	5
	rs11584005	A/G	0.01 (G)	A	35881973	A>G	1	Intron	Intron variant	Bibliographic	9	0
AGO2	rs4961280	C/A/T	0.15 (A)	A	140637315	C>A / C>T	8	NA	Non-coding transcript exon variant	Bibliographic	13	5
	rs77216619	G/A/T	0.01 (T)	G	140586834	G>A / G>T	8	Intron	Intron variant	Bibliographic	16	0
	rs78796470	C/T	0.04 (T)	C	140586871	C>T	8	Intron	Intron variant	Bibliographic	16	0
	rs2176397	C/T	0.30 (T)	C	140612438	C>T	8	Intron	Intron variant	Bibliographic	16	1
	rs2292779	G/C/T	0.33 (G)	C	140551294	G>C / G>T	8	Intron	Intron variant	Bibliographic	9	1
	rs3864659	A/C	0.14 (C)	A	140545763	A>C	8	Intron	Intron variant	Bibliographic	11	2
	rs11786030	G/A	0.21 (G)	A	140620073	G>A	8	Intron	Intron variant	Bibliographic	16	1
	rs7005286	T/C	0.17 (T)	C	140584361	T>C	8	Intron	Intron variant	Bibliographic	9	0
TARBP2	rs784567	G/A/T	0.22 (A)	G	53500681	G>A / G>T	12	Promoter	Non-coding transcript exon variant	Bibliographic	22	5
	rs34649330	C/T	0.08 (T)	C	53499247	C>T	12	Intron	Intron variant	Bibliographic	1	1
PIWIL1	rs10773771	C/T	0.50 (T)	C	130371771	C>T	12	3' UTR	3' UTR variant	Bibliographic	8	7
	rs11060845	G/T	0.13 (T)	G	130367629	G>T	12	Intron	Intron variant	Bibliographic	7	5
	rs1106042	G/A	0.09 (A)	A	130357093	G>A	12	p.Arg527Lys	Missense variant	Bibliographic	8	4

Supplementary Table S2. Scale for methodological quality assessment.

Criterion	Score
**1. Representativeness of cases**	
Selected from population or cancer registry or multiple cancer center sites	2
Selected from hospital (Oncology Department or Cancer Institute)	1.5
Selected from pathology archives, but without description	1
Not described	0
**2. Source of controls**	
Population or community based	3
Blood donors or volunteers	2
Hospital-based (cancer-free controls)	1.5
Healthy volunteers, but without total description	1
Cancer-free controls with related diseases	0.5
Not described	0
**3. Ascertainment of relevant cancer**	
Histological or pathological confirmation	2
Diagnosis of cancer by patient medical record	1
Not described	0
**4. Case-control match**	
Matched by age and gender	1
Not matched by age and gender	0
**5. Quality control of genotyping method**	
Repetition of partial/total tested samples with a different method	2
Repetition of partial/total tested samples with the same method	1
Not described	0
**6. Genotyping examination**	
Genotyping done under ‘‘blinded’’ condition	1
Unblinded or not mentioned	0
**7. Specimens used for determining genotypes**	
White blood cells or normal tissues	1
Tumor tissues or exfoliated cells of tissue	0
**8. Hardy-Weinberg equilibrium in controls**	
Hardy-Weinberg equilibrium	1
Hardy-Weinberg disequilibrium	0
**9. Total sample size**	
>1000	3
>500 and <1000	2
>200 and <500	1
<200	0
**10. Association assessment**	
Assess association between genotypes and relevant cancer with appropriate statistics and adjustment for confounders	2
Assess association between genotypes and relevant cancer with appropriate statistics without adjustment for confounders	1
Inappropriate statistics used	0

Supplementary Table S3. Quality score assessment for included studies.

N	Gene	SNP	First author	Year	I	II	III	IV	V	VI	VII	VIII	IX	X	Total
1	DROSHA	rs10719	Bermisheva	2018	2	3	0	0	1	0	1	1	2	1	11
			Song	2017	1.5	1.5	2	1	1	0	1	1	3	2	14
			Kim	2016	1.5	3	0	1	2	1	1	1	1	2	13.5
			Martin-Guerrero	2015	2	3	2	1	1	0	1	1	1	2	14
			Cho	2015	1.5	3	2	1	2	1	1	1	2	2	16.5
			Yuan	2013	2	1.5	2	1	1	0	1	1	3	2	14.5
			Jiang	2013	2	3	2	1	1	0	1	1	3	2	16
			Kim	2010	1.5	1.5	2	1	1	0	1	1	0	2	11
			Yang	2008	2	3	2	1	1	1	1	0	2	2	15
			Horikawa	2008	1.5	3	2	1	1	1	1	1	1	2	14.5
2	DROSHA	rs6877842	Mohammadpour-Gharehbagh	2019	1.5	3	2	1	1	0	1	1	1	1	12.5
			Kim	2016	1.5	3	0	1	2	1	1	1	1	2	13.5
			Bruzgielewicz	2016	1.5	3	2	1	1	0	1	1	0	1	11.5
			Osuch-Wojcikiewicz	2015	1.5	1.5	2	1	1	0	0	1	1	1	10
			Martin-Guerrero	2015	2	3	2	1	1	0	1	1	1	2	14
			Kim	2010	1.5	1.5	2	1	1	0	1	1	0	2	11
			Yang	2008	2	3	2	1	1	1	1	1	3	2	17
			Horikawa	2008	1.5	3	2	1	1	1	1	1	2	2	15.5
3	DICER1	rs13078	Huang	2018	1.5	3	2	1	1	0	1	1	2	2	14.5
			Kim	2016	1.5	3	0	1	2	1	1	1	1	2	13.5
			Osuch-Wojcikiewicz	2015	1.5	1.5	2	1	1	0	0	0	1	1	9
			Martin-Guerrero	2015	2	3	2	1	1	0	1	1	1	2	14
			Yuan	2013	2	1.5	2	1	1	0	1	1	3	2	14.5
			Jiang	2013	2	3	2	1	1	0	1	1	3	2	16
			Kim	2010	1.5	1.5	2	1	1	0	1	1	0	2	11
			Yang	2008	2	3	2	1	1	1	1	1	3	2	17
			Horikawa	2008	1.5	3	2	1	1	1	1	1	2	2	15.5
4	DICER1	rs1057035	Wang	2017	1.5	1.5	2	1	1	0	1	1	2	2	13
			Yuan	2016	1.5	3	2	1	1	0	1	1	3	2	15.5
			Martin-Guerrero	2015	2	3	2	1	1	0	1	1	1	2	14
			Yuan	2013	2	1.5	2	1	1	0	1	1	3	2	14.5
			Slaby	2013	1.5	2	2	1	1	0	1	1	1	1	11.5
			Liu	2013	1.5	3	2	1	1	0	1	1	3	2	15.5
			Jiang	2013	2	3	2	1	1	0	1	1	3	2	16
			Zu	2013	1.5	1.5	0	1	1	0	0	1	3	2	11
			Chen	2013	2	3	2	1	1	0	1	1	3	2	16
			Wang	2012	1.5	3	2	1	1	0	0	0	3	2	13.5
			Ma	2012	2	3	0	1	1	0	1	1	3	2	14
5	DICER1	rs3742330	Mohammadpour-Gharehbagh	2019	1.5	3	2	1	1	0	1	1	1	1	12.5
			Kim	2019	1.5	1.5	2	1	1	0	1	1	3	2	14
			Oz	2018	0	1.5	2	1	1	0	1	0	0	1	7.5
			Huang	2018	1.5	3	2	1	1	0	1	0	2	2	13.5
			Song	2017	1.5	1.5	2	1	1	0	1	1	3	2	14
			Nikolic	2017	1.5	1	0	1	1	0	1	1	2	2	10.5
			Yuan	2016	1.5	3	2	1	1	0	1	1	3	2	15.5
			Peckham-Gregory	2016	2	1.5	1	1	1	0	1	1	2	2	12.5
			Kim	2016	1.5	3	0	1	2	1	1	1	1	2	13.5
			Osuch-Wojcikiewicz	2015	1.5	1.5	2	1	1	0	0	0	1	1	9
			Cho	2015	1.5	3	2	1	2	1	1	1	2	2	16.5
			Zheng	2013	NA	3	NA	NA	1	NA	NA	1	2	NA	NA
			Yuan	2013	2	1.5	2	1	1	0	1	1	3	2	14.5
			Kim	2010	1.5	1.5	2	1	1	0	1	1	0	2	11
			Yang	2008	2	3	2	1	1	1	1	1	3	2	17
			Horikawa	2008	1.5	3	2	1	1	1	1	1	2	2	15.5
6	XPO5	rs11077	Mohammadpour-Gharehbagh	2019	1.5	3	2	1	1	0	1	1	1	1	12.5
			Thakkar	2018	1.5	3	0	1	1	0	1	1	1	1	10.5
			Huang	2018	1.5	3	2	1	1	0	1	1	1	2	13.5
			Wen	2017	2	1.5	2	1	1	0	1	1	3	2	14.5
			Peckham-Gregory	2016	2	1.5	1	1	1	0	1	1	2	2	12.5
			Kim	2016	1.5	3	0	1	2	1	1	1	1	2	13.5
			Zhao	2015	1.5	3	0	1	1	0	1	1	1	1	10.5
			Xie	2015	1.5	3	0	1	1	0	1	1	1	1	10.5
			Osuch-Wojcikiewicz	2015	1.5	1.5	2	1	1	0	0	0	1	1	9
			Cho	2015	1.5	3	2	1	2	1	1	1	2	2	16.5
			Kim	2010	1.5	1.5	2	1	1	0	1	0	0	2	10
			Yang	2008	2	3	2	1	1	1	1	1	3	2	17
			Horikawa	2008	1.5	3	2	1	1	1	1	0	2	2	14.5
7	RAN	rs14035	Wang	2018	2	1.5	0	1	1	0	1	1	3	2	12.5
			Huang	2018	1.5	3	2	1	1	0	1	1	2	2	14.5
			Peckham-Gregory	2016	2	1.5	1	1	1	0	1	1	2	2	12.5
			Kim	2016	1.5	3	0	1	2	1	1	1	1	2	13.5
			Osuch-Wojcikiewicz	2015	1.5	1.5	2	1	1	0	0	0	1	1	9
			Martin-Guerrero	2015	2	3	2	1	1	0	1	1	1	2	14
			Cho	2015	1.5	3	2	1	2	1	1	1	2	2	16.5
			Roy	2014	2	1.5	2	0	1	0	1	1	2	2	12.5
			Li	2012	2	3	2	1	2	0	1	1	3	1	16

			Kim	2010	1.5	1.5	2	1	1	0	1	1	0	2	11
			Yang	2008	2	3	2	1	1	1	1	0	3	2	16
			Horikawa	2008	1.5	3	2	1	1	1	1	1	2	2	15.5
8	RAN	rs3803012	Wang	2017	1.5	1.5	2	1	1	0	1	1	2	2	13
			Liu	2013	1.5	3	2	1	1	0	1	1	3	2	15.5
			Jiang	2013	2	3	2	1	1	0	1	1	3	2	16
			Chen	2013	2	3	2	1	1	0	1	1	3	2	16
			Ma	2012	2	3	0	1	1	0	1	1	3	2	14
9	DGCR8	rs3757	Osuch-Wojcikiewicz	2015	1.5	1.5	2	1	1	0	0	0	1	1	9
			Mi	2014	2	1.5	2	1	1	0	1	1	2	2	13.5
			Zu	2013	1.5	1.5	0	1	1	0	0	1	3	2	11
			Wang	2012	1.5	3	2	1	1	0	0	0	3	2	13.5
			Kim	2010	1.5	1.5	2	1	1	0	1	1	0	2	11
			Yang	2008	2	3	2	1	1	1	1	1	3	2	17
			Horikawa	2008	1.5	3	2	1	1	1	1	1	2	2	15.5
10	DGCR8	rs1640299	Bermisheva	2018	2	3	0	0	1	0	1	1	2	1	11
			Bruzgielewicz	2016	1.5	3	2	1	1	0	1	1	0	1	11.5
			Osuch-Wojcikiewicz	2015	1.5	1.5	2	1	1	0	0	0	1	1	9
			Martin-Guerrero	2015	2	3	2	1	1	0	1	1	1	2	14
			Jiang	2013	2	3	2	1	1	0	1	1	3	2	16
			Kim	2010	1.5	1.5	2	1	1	0	1	1	0	2	11
			Yang	2008	2	3	2	1	1	1	1	1	3	2	17
			Horikawa	2008	1.5	3	2	1	1	1	1	1	2	2	15.5
11	DGCR8	rs417309	Bruzgielewicz	2016	1.5	3	2	1	1	0	1	1	0	1	11.5
			Osuch-Wojcikiewicz	2015	1.5	1.5	2	1	1	0	0	1	1	1	10
			Martin-Guerrero	2015	2	3	2	1	1	0	1	1	1	2	14
			Jiang	2013	2	3	2	1	1	0	1	1	3	2	16
			Kim	2010	1.5	1.5	2	1	1	0	1	1	0	2	11
			Yang	2008	2	3	2	1	1	1	1	1	3	2	17
			Horikawa	2008	1.5	3	2	1	1	1	1	1	2	2	15.5
12	DDX20	rs197412	Peckham-Gregory	2016	2	1.5	1	1	1	0	1	1	2	2	12.5
			Yuan	2016	1.5	3	2	1	1	0	1	1	3	2	15.5
			Martin-Guerrero	2015	2	3	2	1	1	0	1	1	1	2	14
			Roy	2014	2	1.5	2	0	1	0	1	1	2	2	12.5
			Roy	2014	2	1.5	0	1	1	0	1	1	2	2	11.5
			Jiang	2013	2	3	2	1	1	0	1	1	3	2	16
			Kim	2010	1.5	1.5	2	1	1	0	1	1	0	2	11
			Yang	2008	2	3	2	1	1	1	1	0	3	2	16
			Horikawa	2008	1.5	3	2	1	1	1	1	1	2	2	15.5
13	DDX20	rs197414	Nikolic	2017	1.5	1	0	1	1	0	1	1	2	2	10.5
			Martin-Guerrero	2015	2	3	2	1	1	0	1	1	1	2	14
			Kim	2010	1.5	1.5	2	1	1	0	1	0	0	2	10
			Yang	2008	2	3	2	1	1	1	1	1	3	2	17
			Horikawa	2008	1.5	3	2	1	1	1	1	1	2	2	15.5
14	GEMIN4	rs7813	Verma	2019	1.5	3	2	1	1	0	1	1	1	1	12.5
			Song	2017	1.5	1.5	2	1	1	0	1	1	3	2	14
			Nikolic	2017	1.5	1	0	1	1	0	1	1	2	2	10.5
			Peckham-Gregory	2016	2	1.5	1	1	1	0	1	1	2	2	12.5
			Fang	2016	2	1.5	2	1	1	0	1	1	2	2	13.5
			Zhang	2014	1	3	2	1	2	0	1	0	1	1	12
			Qu	2014	2	3	2	1	1	0	1	1	2	2	15
			Jiang	2013	2	3	2	1	1	0	1	1	3	2	16
			Liu	2012	2	1.5	2	1	1	0	1	1	2	1	12.5
			Sung	2011	2	1.5	2	1	1	0	1	1	3	2	14.5
			Kim	2010	1.5	1.5	2	1	1	0	1	1	0	2	11
			Yang	2008	2	3	2	1	1	1	1	1	3	2	17
			Ye	2008	1.5	1.5	2	1	1	0	1	1	2	2	13
			Horikawa	2008	1.5	3	2	1	1	1	1	1	2	2	15.5
15	GEMIN4	rs2740348	Bermisheva	2018	2	3	0	0	1	0	1	1	2	1	11
			Peckham-Gregory	2016	2	1.5	1	1	1	0	1	1	2	2	12.5
			Mullany	2016	2	3	0	1	1	0	1	1	3	1	13
			Gutierrez-Malacatt	2016	2	1.5	2	1	1	0	1	1	2	1	12.5
			Martin-Guerrero	2015	2	3	2	1	1	0	1	1	1	2	14
			Jiang	2013	2	3	2	1	1	0	1	1	3	2	16
			Liu	2012	2	1.5	2	1	1	0	1	0	2	1	11.5
			Kim	2010	1.5	1.5	2	1	1	0	1	1	0	2	11
			Yang	2008	2	3	2	1	1	1	1	1	3	2	17
			Ye	2008	1.5	1.5	2	1	1	0	1	1	2	2	13
			Horikawa	2008	1.5	3	2	1	1	1	1	1	2	2	15.5
16	GEMIN4	rs3744741	Verma	2019	1.5	3	2	1	1	0	1	1	1	1	12.5
			Martin-Guerrero	2015	2	3	2	1	1	0	1	1	1	2	14
			Jiang	2013	2	3	2	1	1	0	1	1	3	2	16
			Liu	2012	2	1.5	2	1	1	0	1	1	2	1	12.5
			Sung	2011	2	1.5	2	1	1	0	1	1	3	2	14.5
			Kim	2010	1.5	1.5	2	1	1	0	1	1	0	2	11
			Yang	2008	2	3	2	1	1	1	1	1	3	2	17
			Ye	2008	1.5	1.5	2	1	1	0	1	1	2	2	13
			Horikawa	2008	1.5	3	2	1	1	1	1	1	2	2	15.5
17	AGO1	rs595961	Fang	2016	2	1.5	2	1	1	0	1	1	2	2	13.5
			Martin-Guerrero	2015	2	3	2	1	1	0	1	1	1	2	14
			Kim	2010	1.5	1.5	2	1	1	0	1	1	0	2	11

			**Yang**	**2008**	**2**	**3**	**2**	**1**	**1**	**1**	**1**	**1**	**3**	**2**	**17**
			**Horikawa**	**2008**	**1.5**	**3**	**2**	**1**	**1**	**1**	**1**	**1**	**2**	**2**	**15.5**
**18**	**AGO1**	**rs636832**	**Song**	**2017**	**1.5**	**1.5**	**2**	**1**	**1**	**0**	**1**	**1**	**3**	**2**	**14**
			**Gutierrez-Malacatt**	**2016**	**2**	**1.5**	**2**	**1**	**1**	**0**	**1**	**1**	**2**	**1**	**12.5**
			**Martin-Guerrero**	**2015**	**2**	**3**	**2**	**1**	**1**	**0**	**1**	**1**	**1**	**2**	**14**
			**Kim**	**2010**	**1.5**	**1.5**	**2**	**1**	**1**	**0**	**1**	**1**	**0**	**2**	**11**
			**Yang**	**2008**	**2**	**3**	**2**	**1**	**1**	**1**	**1**	**1**	**3**	**2**	**17**
			**Horikawa**	**2008**	**1.5**	**3**	**2**	**1**	**1**	**1**	**1**	**1**	**2**	**2**	**15.5**
**19**	**AGO2**	**rs4961280**	**Nikolic**	**2017**	**1.5**	**1**	**0**	**1**	**1**	**0**	**1**	**1**	**2**	**2**	**10.5**
			**Martin-Guerrero**	**2015**	**2**	**3**	**2**	**1**	**1**	**0**	**1**	**1**	**1**	**2**	**14**
			**Kim**	**2010**	**1.5**	**1.5**	**2**	**1**	**1**	**0**	**1**	**1**	**0**	**2**	**11**
			**Yang**	**2008**	**2**	**3**	**2**	**1**	**1**	**1**	**1**	**1**	**3**	**2**	**17**
			**Horikawa**	**2008**	**1.5**	**3**	**2**	**1**	**1**	**1**	**1**	**1**	**2**	**2**	**15.5**
**20**	**TARBP2**	**rs784567**	**Nikolic**	**2017**	**1.5**	**1**	**0**	**1**	**1**	**0**	**1**	**0**	**2**	**2**	**9.5**
			**Osuch-Wojcikiewicz**	**2015**	**1.5**	**1.5**	**2**	**1**	**1**	**0**	**0**	**0**	**1**	**1**	**9**
			**Martin-Guerrero**	**2015**	**2**	**3**	**2**	**1**	**1**	**0**	**1**	**1**	**1**	**2**	**14**
			**Kim**	**2010**	**1.5**	**1.5**	**2**	**1**	**1**	**0**	**1**	**0**	**0**	**2**	**10**
			**Yang**	**2008**	**2**	**3**	**2**	**1**	**1**	**1**	**1**	**1**	**3**	**2**	**17**
			**Horikawa**	**2008**	**1.5**	**3**	**2**	**1**	**1**	**1**	**1**	**1**	**2**	**2**	**15.5**
**21**	**PIWIL1**	**rs1106042**	**Yuan**	**2016**	**1.5**	**3**	**2**	**1**	**1**	**0**	**1**	**1**	**3**	**2**	**15.5**
			**Martin-Guerrero**	**2015**	**2**	**3**	**2**	**1**	**1**	**0**	**1**	**1**	**1**	**2**	**14**
			**Kim**	**2010**	**1.5**	**1.5**	**2**	**1**	**1**	**0**	**1**	**1**	**0**	**2**	**11**
			**Yang**	**2008**	**2**	**3**	**2**	**1**	**1**	**1**	**1**	**1**	**3**	**2**	**17**
			**Horikawa**	**2008**	**1.5**	**3**	**2**	**1**	**1**	**1**	**1**	**1**	**1**	**2**	**14.5**
**22**	**PIWIL1**	**rs10773771**	**Bermisheva**	**2018**	**2**	**3**	**0**	**0**	**1**	**0**	**1**	**1**	**2**	**1**	**11**
			**Wang**	**2017**	**1.5**	**1.5**	**2**	**1**	**1**	**0**	**1**	**1**	**2**	**2**	**13**
			**Yuan**	**2016**	**1.5**	**3**	**2**	**1**	**1**	**0**	**1**	**1**	**3**	**2**	**15.5**
			**Liu**	**2013**	**1.5**	**3**	**2**	**1**	**1**	**0**	**1**	**1**	**3**	**2**	**15.5**
			**Jiang**	**2013**	**2**	**3**	**2**	**1**	**1**	**0**	**1**	**1**	**3**	**2**	**16**
			**Chen**	**2013**	**2**	**3**	**2**	**1**	**1**	**0**	**1**	**1**	**3**	**2**	**16**
			**Ma**	**2012**	**2**	**3**	**0**	**1**	**1**	**0**	**1**	**0**	**3**	**2**	**13**

Columns I to X represents the 10 assessment questions in Supplementary Table S2.

Supplementary Table S4. Characteristics of the included studies.

First author, year [Ref]	Cancer type	Country	Cases	Controls	Genotyping Method	Control Source	HWE (P)	Quality score
DROSHA rs10719 A > G								
**Bermisheva et al. 2018 [42]**	Breast cancer	Russia	413	361	TaqMan OpenArray Genotyping	PB	Eqi	L
**Song et al. 2017 [43]**	Gastric cancer	China	628	502	HRMA	HB	Eqi	H
**Kim et al. 2016 [44]**	Hepatocellular carcinoma	South Korea	147	209	PCR-RFLP	PB	Eqi	H
**Martin-Guerrero et al. 2015 [45]**	CLL	Spain	102	345	TaqMan OpenArray Genotyping	PB	Eqi	H
**Cho et al. 2015 [46]**	Colorectal cancer	South Korea	408	400	PCR-RFLP	HB	Eqi	H
**Yuan et al. 2013 [47]**	Bladder cancer	China	684	727	TaqMan genotyping assay	HB	Eqi	H
**Jiang et al. 2013 [48]**	Breast cancer	China	847	878	TaqMan OpenArray Genotyping	PB	Eqi	H
**Kim et al. 2010 [49]**	Lung cancer	Republic of Korea	97	97	SMSG	HB	Eqi	L
**Yang et al. 2008 [50]**	Bladder cancer	USA	534	442	SNPlex technology	PB	Diseq	H
**Horikawa et al. 2008 [51]**	Renal cell carcinoma	USA	252	246	SNPlex technology	PB	Eqi	H
DROSHA rs6877842 G > C								
**Mohammadpour-Gharehbagh et al. 2020 [52]**	Papillary Thyroid Carcinoma	Iran	120	130	PCR-RFLP	PB	Eqi	H
**Kim et al. 2016 [44]**	hepatocellular carcinoma	South Korea	147	209	PCR-RFLP	PB	Eqi	H
**Bruzgielewicz et al. 2016 [53]**	Larynx Cancer	Poland	100	100	TaqMan genotyping assay	PB	Eqi	L
**Osuch-Wojcikiewicz et al. 2015 [54]**	Larynx Cancer	Poland	128	170	TaqMan genotyping assay	HB	Eqi	L
**Martin-Guerrero et al. 2015 [45]**	CLL	Spain	102	341	TaqMan OpenArray Genotyping	PB	Eqi	H
**Kim et al. 2010 [49]**	Lung cancer	Republic of Korea	93	93	SMSG	HB	Eqi	L
**Yang et al. 2008 [50]**	Bladder cancer	USA	735	732	SNPlex technology	PB	Eqi	H
**Horikawa et al. 2008 [51]**	Renal cell carcinoma	USA	275	278	SNPlex technology	PB	Eqi	H
DGCR8 rs3757 G > A								
**Osuch-Wojcikiewicz et al. 2015 [54]**	Larynx Cancer	Poland	122	170	TaqMan genotyping assay	PB	Diseq	L
**Mi et al. 2014 [55]**	Cervical cancer	China	338	332	MALDI-TOF within the MassARRAY system	HB	Eqi	H
**Zu et al. 2013 [56]**	Lung cancer	China	600	600	TaqMan genotyping assay	HB	Eqi	L
**Wang et al. 2012 [57]**	Esophageal cancer	China	534	593	SNaPshot assay	PB	Diseq	H
**Kim et al. 2010 [49]**	Lung cancer	Republic of Korea	94	90	SMSG	HB	Eqi	L
**Yang et al. 2008 [50]**	Bladder cancer	USA	737	735	SNPlex technology	HB	Eqi	H
**Horikawa et al. 2008 [51]**	Renal cell carcinoma	USA	276	278	SNPlex technology	PB	Eqi	H
DGCR8 rs417309 G > A								
**Bruzgielewicz et al. 2016 [53]**	Larynx Cancer	Poland	100	100	TaqMan genotyping assay	PB	Eqi	L
**Osuch-Wojcikiewicz et al. 2015 [54]**	Larynx Cancer	Poland	112	170	TaqMan genotyping assay	PB	Eqi	L
**Martin-Guerrero et al. 2015 [45]**	CLL	Spain	105	346	TaqMan OpenArray Genotyping	PB	Eqi	H
**Jiang et al. 2013 [48]**	Breast cancer	China	860	893	TaqMan OpenArray Genotyping	PB	Eqi	H
**Kim et al. 2010 [49]**	Lung cancer	Republic of Korea	98	97	SMSG	HB	Eqi	L
**Yang et al. 2008 [50]**	Bladder cancer	USA	740	738	SNPlex technology	HB	Eqi	H
**Horikawa et al. 2008 [51]**	Renal cell carcinoma	USA	277	278	SNPlex technology	PB	Eqi	H
DGCR8 rs1640299 T > G								
**Bermisheva et al. 2018 [42]**	Breast cancer	Russia	413	355	TaqMan OpenArray Genotyping	PB	Eqi	L
**Bruzgielewicz et al. 2016 [53]**	Larynx Cancer	Poland	100	100	TaqMan genotyping assay	PB	Eqi	L
**Osuch-Wojcikiewicz et al. 2015 [54]**	Larynx Cancer	Poland	113	170	TaqMan genotyping assay	PB	Diseq	L
**Martin-Guerrero et al. 2015 [45]**	CLL	Spain	103	349	TaqMan OpenArray Genotyping	PB	Eqi	H
**Jiang et al. 2013 [48]**	Breast cancer	China	849	891	TaqMan OpenArray Genotyping	PB	Eqi	H
**Kim et al. 2010 [49]**	Lung cancer	Republic of Korea	98	97	SMSG	HB	Eqi	L
**Yang et al. 2008 [50]**	Bladder cancer	USA	737	737	SNPlex technology	HB	Eqi	H
**Horikawa et al. 2008 [51]**	Renal cell carcinoma	USA	277	278	SNPlex technology	PB	Eqi	H
XPO5 rs11077 T > G								
**Mohammadpour-Gharehbagh et al. 2020 [52]**	Papillary Thyroid Cancer	Iran	120	130	PCR-RFLP	PB	Eqi	H
**Thakkar et al. 2018 [58]**	Hodgkin lymphoma	India	101	200	TaqMan genotyping assay	PB	Eqi	L
**Huang et al. 2018 [59]**	Cervical cancer	China	296	296	MALDI-TOF MS	PB	Eqi	H
**Wen et al. 2017 [60]**	Papillary Thyroid Cancer	China	1134	1228	TaqMan genotyping assay	HB	Eqi	H
**Peckham-Gregory et al. 2016 [61]**	Non-Hodgkin lymphoma	USA	178	529	Customized Fluidigm Dynamic 96.96 ArrayTM assay	HB	Eqi	H
**Kim et al. 2016 [44]**	Hepatocellular Carcinoma	South Korea	147	209	PCR-RFLP	PB	Eqi	H
**Zhao et al. 2015 [62]**	Colorectal cancer	China	163	142	PCR-ligase detection reaction	PB	Eqi	L
**Xie et al. 2015 [63]**	Gastric cancer	China	137	142	PCR-ligase detection reaction	PB	Eqi	L
**Osuch-Wojcikiewicz et al. 2015 [54]**	Larynx Cancer	Poland	124	160	TaqMan genotyping assay	PB	Diseq	L
**Cho et al. 2015 [46]**	Colorectal cancer	South Korea	408	400	PCR-RFLP	HB	Eqi	H
**Kim et al. 2010 [49]**	Lung cancer	Republic of Korea	100	99	SMSG	HB	Diseq	L
**Yang et al. 2008 [50]**	Bladder cancer	USA	718	726	SNPlex technology	HB	Eqi	H

**Horikawa et al. 2008 [51]**	Renal cell carcinoma	USA	276	277	SNPlex technology	PB	Diseq	H
**RAN rs14035 C > T**								
**Wang et al. 2018 [64]**	Neuroblastoma	China	429	884	TaqMan genotyping assay	HB	Eqi	H
**Huang et al. 2018 [59]**	Cervical cancer	China	295	296	MALDI-TOF MS method	PB	Eqi	H
**Peckham-Gregory et al. 2016 [61]**	Non-Hodgkin lymphoma	USA	180	529	Customized Fluidigm Dynamic 96.96 ArrayTM assay	HB	Eqi	H
**Kim et al. 2016 [44]**	Hepatocellular Carcinoma	South Korea	147	209	PCR-RFLP	PB	Eqi	H
**Osuch-Wojcikiewicz et al. 2015 [54]**	Larynx Cancer	Poland	110	170	TaqMan genotyping assay	PB	Diseq	L
**Martin-Guerrero et al. 2015 [45]**	CLL	Spain	99	342	TaqMan OpenArray Genotyping	PB	Eqi	H
**Cho et al. 2015 [46]**	Colorectal cancer	South Korea	408	400	PCR-RFLP	HB	Eqi	H
**Roy et al. 2014 [65]**	Oral cancer	India	439	438	TaqMan genotyping assay	HB	Eqi	H
**Li et al. 2012 [66]**	Hepatocellular Carcinoma	China	532	532	PCR-RFLP	PB	Eqi	H
**Kim et al. 2010 [49]**	Lung cancer	Republic of Korea	93	90	SMSG	HB	Eqi	L
**Yang et al. 2008 [50]**	Bladder cancer	USA	735	732	SNPlex technology	HB	Diseq	H
**Horikawa et al. 2008 [51]**	Renal cell carcinoma	USA	276	278	SNPlex technology	PB	Eqi	H
**RAN rs3803012 A > C/A > G**								
**Wang et al. 2017 [67]**	Hepatocellular carcinoma	China	312	320	TaqMan genotyping assay	HB	Eqi	H
**Liu et al. 2013 [68]**	Hepatocellular carcinoma	China	1256	1340	TaqMan genotyping assay	PB	Eqi	H
**Jiang et al. 2013 [48]**	Breast cancer	China	870	884	TaqMan OpenArray Genotyping	PB	Eqi	H
**Chen et al. 2013 [69]**	Cervical cancer	China	1471	1529	TaqMan genotyping assay	PB	Eqi	H
**Ma et al. 2012 [70]**	Head and Neck Cancer	China	391	892	TaqMan genotyping assay	PB	Eqi	H
**DICER1 rs13078 A > T**								
**Huang et al. 2018 [59]**	Cervical cancer	China	295	296	MALDI-TOF MS method	PB	Eqi	H
**Kim et al. 2016 [44]**	Hepatocellular Carcinoma	South Korea	147	209	PCR-RFLP	PB	Eqi	H
**Osuch-Wojcikiewicz et al. 2015 [54]**	Larynx Cancer	Poland	128	170	TaqMan genotyping assay	PB	Diseq	L
**Martin-Guerrero et al. 2015 [45]**	CLL	Spain	105	345	TaqMan OpenArray Genotyping	PB	Eqi	H
**Yuan et al. 2013 [71]**	Bladder cancer	China	679	723	TaqMan genotyping assay	HB	Eqi	H
**Jiang et al. 2013 [48]**	Breast cancer	China	864	892	TaqMan OpenArray Genotyping	PB	Eqi	H
**Kim et al. 2010 [49]**	Lung cancer	Republic of Korea	98	97	SMSG	HB	Eqi	L
**Yang et al. 2008 [50]**	Bladder cancer	USA	734	738	SNPlex technology	PB	Eqi	H
**Horikawa et al. 2008 [51]**	Renal cell carcinoma	USA	277	278	SNPlex technology	PB	Eqi	H
**DICER1 rs1057035 T > C**								
**Wang et al. 2017 [67]**	Hepatocellular carcinoma	China	312	320	TaqMan genotyping assay	HB	Eqi	H
**Yuan et al. 2016 [71]**	Head and Neck Cancer	China	575	1548	Illumina Infmium BeadChip platform	PB	Eqi	H
**Martin-Guerrero et al. 2015 [45]**	CLL	Spain	104	346	TaqMan OpenArray Genotyping	PB	Eqi	H
**Yuan et al. 2013 [47]**	Bladder cancer	China	685	730	TaqMan genotyping assay	HB	Eqi	H
**Slaby et al. 2013 [72]**	Colorectal cancer	Czech Republic	198	203	TaqMan genotyping assay	PB	Eqi	L
**Liu et al. 2013 [68]**	Hepatocellular carcinoma	China	1275	1339	TaqMan genotyping assay	PB	Eqi	H
**Jiang et al. 2013 [48]**	Breast cancer	China	848	881	TaqMan OpenArray Genotyping	PB	Eqi	H
**Zu et al. 2013 [56]**	Lung cancer	China	600	600	TaqMan genotyping assay	HB	Eqi	L
**Chen et al. 2013 [69]**	Cervical cancer	China	1475	1528	TaqMan genotyping assay	PB	Eqi	H
**Wang et al. 2012 [57]**	Esophageal cancer	China	533	601	SNaPshot assay	PB	Diseq	H
**Ma et al. 2012 [70]**	Head and Neck Cancer	China	394	891	TaqMan genotyping assay	PB	Eqi	H
**DICER1 rs3742330 A> G**								
**Mohammadpour-Gharehbagh et al. 2020 [52]**	Papillary Thyroid Cancer	Iran	120	130	PCR-RFLP	PB	Eqi	H
**Kim et al. 2019 [73]**	Colorectal cancer	South Korea	700	1400	MassARRAY iPLEX Gold Assay	HB	Eqi	H
**Oz et al. 2018 [74]**	Endometrial cancer	Turkey	80	79	TaqMan genotyping assay	HB	Diseq	L
**Huang et al. 2018 [59]**	Cervical cancer	China	296	296	MALDI-TOF MS method	PB	Diseq	H
**Song et al. 2017 [43]**	Gastric cancer	China	628	502	HRMA	HB	Eqi	H
**Nikolic et al. 2017 [75]**	Prostate cancer	Serbia	353	318	HRMA	PB	Eqi	L
**Yuan et al. 2016 [71]**	Head and Neck Cancer	China	575	1551	Illumina Infmium BeadChip platform	PB	Eqi	H
**Peckham-Gregory et al. 2016 [61]**	Non-Hodgkin lymphoma	USA	180	529	Customized Fluidigm Dynamic 96.96 ArrayTM assay	HB	Eqi	H
**Kim et al. 2016 [44]**	Hepatocellular Carcinoma	South Korea	147	209	PCR-RFLP	PB	Eqi	H
**Osuch-Wojcikiewicz et al. 2015 [54]**	Larynx Cancer	Poland	123	170	TaqMan genotyping assay	PB	Diseq	L
**Cho et al. 2015 [46]**	Colorectal cancer	South Korea	408	400	PCR-RFLP	HB	Eqi	H
**Zheng et al. 2013 [76]**	Esophageal cancer	China	380	380	MALDI-TOF MS method	PB	Eqi	H
**Yuan et al. 2013 [47]**	Bladder cancer	China	683	727	TaqMan genotyping assay	HB	Eqi	H
**Kim et al. 2010 [49]**	Lung cancer	Republic of Korea	100	100	SMSG	HB	Eqi	L
**Yang et al. 2008 [50]**	Bladder cancer	USA	727	724	SNPlex technology	HB	Eqi	H
**Horikawa et al. 2008 [51]**	Renal cell carcinoma	USA	277	278	SNPlex technology	PB	Eqi	H
**TARBP2 rs784567 G > A**								
**Nikolic et al. 2017 [75]**	Prostate cancer	Serbia	355	318	HRMA	PB	Diseq	L

**Osuch-Wojcikiewicz et al. 2015 [54]**	Larynx Cancer	Poland	128	170	TaqMan genotyping assay	PB	Diseq	L
**Martin-Guerrero et al. 2015 [45]**	CLL	Spain	97	338	TaqMan OpenArray Genotyping	PB	Eqi	H
**Kim et al. 2010 [49]**	Lung cancer	Republic of Korea	98	97	SMSG	HB	Diseq	L
**Yang et al. 2008 [50]**	Bladder cancer	USA	737	729	SNPlex technology	HB	Eqi	H
**Horikawa et al. 2008 [51]**	Renal cell carcinoma	USA	276	277	SNPlex technology	PB	Eqi	H
**AGO1 rs595961 A > G**								
**Fang et al. 2016 [77]**	Lung cancer	China	473	395	TaqMan genotyping assay	HB	Eqi	H
**Martin-Guerrero et al. 2015 [78]**	CLL	Spain	103	339	TaqMan OpenArray Genotyping	PB	Eqi	H
**Kim et al. 2010 [49]**	Lung cancer	Republic of Korea	98	97	SMSG	HB	Eqi	L
**Yang et al. 2008 [50]**	Bladder cancer	USA	740	741	SNPlex technology	HB	Eqi	H
**Horikawa et al. 2008 [51]**	Renal cell carcinoma	USA	277	281	SNPlex technology	PB	Eqi	H
**AGO1 rs636832 G > A**								
**Song et al. 2017 [43]**	Gastric cancer	China	628	502	HRMA	HB	Eqi	H
**Gutiérrez-Malacatt et al. 2016 [78]**	CML	Mexico	312	469	TaqMan genotyping assay	HB	Eqi	H
**Martin-Guerrero et al. 2015 [45]**	CLL	Spain	105	347	TaqMan OpenArray Genotyping	PB	Eqi	H
**Kim et al. 2010 [49]**	Lung cancer	Republic of Korea	98	97	Melting-curve analysis using fluorescence-labeled hybridization probes	HB	Eqi	L
**Yang et al. 2008 [50]**	Bladder cancer	USA	738	736	SNPlex technology	HB	Eqi	H
**Horikawa et al. 2008 [51]**	Renal cell carcinoma	USA	277	277	SNPlex technology	PB	Eqi	H
**AGO2 rs4961280 C > A**								
**Nikolic et al. 2017 [75]**	Prostate cancer	Serbia	355	318	TaqMan genotyping assay	PB	Eqi	L
**Martin-Guerrero et al. 2015 [45]**	CLL	Spain	106	349	TaqMan OpenArray Genotyping	PB	Eqi	H
**Kim et al. 2010 [49]**	Lung cancer	Republic of Korea	99	97	SMSG	HB	Eqi	L
**Yang et al. 2008 [50]**	Bladder cancer	USA	729	725	SNPlex technology	HB	Eqi	H
**Horikawa et al. 2008 [51]**	Renal cell carcinoma	USA	276	277	SNPlex technology	PB	Eqi	H
**GEMIN3 (DDX20) rs197412 T > C**								
**Peckham-Gregory et al. 2016 [61]**	Non-Hodgkin lymphoma	USA	180	529	Customized Fluidigm Dynamic 96.96 ArrayTM assay	HB	Eqi	H
**Yuan et al. 2016 [71]**	Head and Neck Cancer	China	575	1550	Illumina Infmium BeadChip platform	PB	Eqi	H
**Martin-Guerrero et al. 2015 [45]**	CLL	Spain	101	344	TaqMan OpenArray Genotyping	PB	Eqi	H
**Roy et al. 2014 [79]**	Oral cancer	India	450	447	TaqMan genotyping assay	HB	Eqi	H
**Roy et al. 2014 [79]**	Oral cancer	India	298	447	TaqMan genotyping assay	HB	Eqi	L
**Jiang et al. 2013 [48]**	Breast cancer	China	853	886	TaqMan OpenArray Genotyping	PB	Eqi	H
**Kim et al. 2010 [49]**	Lung cancer	Republic of Korea	92	90	SMSG	HB	Eqi	L
**Yang et al. 2008 [50]**	Bladder cancer	USA	735	730	SNPlex technology	HB	Diseq	H
**Horikawa et al. 2008 [51]**	Renal cell carcinoma	USA	277	278	SNPlex technology	PB	Eqi	H
**GEMIN3 (DDX20) rs197414 C > A**								
**Nikolic et al. 2017 [75]**	Prostate cancer	Serbia	353	319	PCR-RFLP	HB	Eqi	L
**Martin-Guerrero et al. 2015 [45]**	CLL	Spain	95	321	TaqMan OpenArray Genotyping	PB	Eqi	H
**Kim et al. 2010 [49]**	Lung cancer	Republic of Korea	100	99	SMSG	HB	Diseq	L
**Yang et al. 2008 [50]**	Bladder cancer	USA	740	742	SNPlex technology	HB	Eqi	H
**Horikawa et al. 2008 [51]**	Renal cell carcinoma	USA	278	278	SNPlex technology	PB	Eqi	H
**GEMIN4 rs7813 G > A**								
**Verma et al. 2019 [80]**	Renal cell carcinoma	India	100	225	TaqMan genotyping assay	PB	Eqi	H
**Song et al. 2017 [43]**	Gastric cancer	China	628	502	HRMA	HB	Eqi	H
**Nikolic et al. 2017 [75]**	Prostate cancer	Serbia	355	319	TaqMan genotyping assay	PB	Eqi	L
**Peckham-Gregory et al. 2016 [61]**	Non-Hodgkin lymphoma	USA	180	527	Customized Fluidigm Dynamic 96.96 ArrayTM assay	HB	Eqi	H
**Fang et al. 2016 [77]**	Lung cancer	China	473	395	TaqMan genotyping assay	HB	Eqi	H
**Zhang et al. 2014 [81]**	Prostate cancer	China	348	70	HRMA	PB	Diseq	L
**Qu et al. 2014 [82]**	Esophageal cancer	China	381	426	PCR-RFLP	PB	Eqi	H
**Jiang et al. 2013 [48]**	Breast cancer	China	866	885	TaqMan OpenArray Genotyping	PB	Eqi	H
**Liu et al. 2012 [83]**	Prostate cancer	China	300	244	HRMA	HB	Eqi	H
**Sung et al. 2011 [84]**	Breast cancer	Korea	559	566	TaqMan genotyping assay	HB	Eqi	H
**Kim et al. 2010 [49]**	Lung cancer	Republic of Korea	98	99	SMSG	HB	Eqi	L
**Yang et al. 2008 [50]**	Bladder cancer	USA	736	736	SNPlex technology	HB	Eqi	H
**Ye et al. 2008 [85]**	Esophageal cancer	USA	346	346	SNPlex technology	HB	Eqi	H
**Horikawa et al. 2008 [51]**	Renal cell carcinoma	USA	277	278	SNPlex technology	PB	Eqi	H
**GEMIN4 rs2740348 G > C**								
**Bermisheva et al. 2018 [42]**	Breast cancer	Russia	415	359	TaqMan OpenArray Genotyping	PB	Eqi	L
**Peckham-Gregory et al. 2016 [61]**	Non-Hodgkin lymphoma	USA	180	528	Customized Fluidigm Dynamic 96.96 ArrayTM assay	HB	Eqi	H
**Mullany et al. 2016 [86]**	Colorectal cancer	USA	1115	1173	Illumina HumanHap 550, 610 K arrays	PB	Eqi	H
**Gutiérrez-Malacatt et al. 2016 [78]**	CML	Mexico	312	469	TaqMan genotyping assay	HB	Eqi	H
**Martin-Guerrero et al. 2015 [45]**	CLL	Spain	105	345	TaqMan OpenArray Genotyping	PB	Eqi	H
**Jiang et al. 2013 [48]**	Breast cancer	China	863	888	TaqMan OpenArray Genotyping	PB	Eqi	H
**Liu et al. 2012 [83]**	Prostate cancer	China	300	244	HRMA	HB	Diseq	L

**Kim et al. 2010 [49]**	Lung cancer	Republic of Korea	92	90	SMSG	HB	Eqi	L
**Yang et al. 2008 [50]**	Bladder cancer	USA	738	739	SNPlex technology	HB	Eqi	H
**Ye et al. 2008 [85]**	Esophageal cancer	USA	346	346	SNPlex technology	HB	Eqi	H
**Horikawa et al. 2008 [51]**	Renal cell carcinoma	USA	276	278	SNPlex technology	PB	Eqi	H
**GEMIN4 rs3744741 C > T**								
**Verma et al. 2019 [80]**	Renal cell carcinoma	India	100	225	TaqMan genotyping assay	PB	Eqi	H
**Martin-Guerrero et al. 2015 [45]**	CLL	Spain	106	347	TaqMan OpenArray Genotyping	PB	Eqi	H
**Jiang et al. 2013 [48]**	Breast cancer	China	862	887	TaqMan OpenArray Genotyping	PB	Eqi	H
**Liu et al. 2012 [83]**	Prostate cancer	China	300	244	HRMA	HB	Eqi	H
**Sung et al. 2011 [84]**	Breast cancer	Korea	559	567	TaqMan genotyping assay	HB	Eqi	H
**Kim et al. 2010 [49]**	Lung cancer	Republic of Korea	100	99	SMSG	HB	Eqi	L
**Yang et al. 2008 [50]**	Bladder cancer	USA	742	740	SNPlex technology	HB	Eqi	H
**Ye et al. 2008 [85]**	Esophageal cancer	USA	346	346	SNPlex technology	HB	Eqi	H
**Horikawa et al. 2008 [51]**	Renal cell carcinoma	USA	277	277	SNPlex technology	PB	Eqi	H
**PIWIL1 rs1106042 G > A**								
**Yuan et al. 2016 [71]**	Head and Neck Cancer	China	575	1551	Illumina Infmium BeadChip	PB	Eqi	H
**Martin-Guerrero et al. 2015 [45]**	CLL	Spain	104	343	TaqMan OpenArray Genotyping	PB	Eqi	H
**Kim et al. 2010 [49]**	Lung cancer	Republic of Korea	97	97	SMSG	HB	Eqi	L
**Yang et al. 2008 [50]**	Bladder cancer	USA	708	691	SNPlex technology	HB	Eqi	H
**Horikawa et al. 2008 [51]**	Renal cell carcinoma	USA	263	235	SNPlex technology	PB	Eqi	H
**PIWIL1 rs10773771 C > T**								
**Bermisheva et al. 2018 [42]**	Breast cancer	Russia	403	350	TaqMan OpenArray Genotyping	PB	Eqi	L
**Wang et al. 2017 [67]**	Hepatocellular carcinoma	China	312	320	TaqMan genotyping assay	HB	Eqi	H
**Yuan et al. 2016 [71]**	Head and Neck Cancer	China	575	1550	Illumina Infmium BeadChip	PB	Eqi	H
**Liu et al. 2013 [68]**	Hepatocellular carcinoma	China	1285	1334	TaqMan genotyping assay	PB	Eqi	H
**Jiang et al. 2013 [48]**	Breast cancer	China	854	876	TaqMan OpenArray Genotyping	PB	Eqi	H
**Chen et al. 2013 [69]**	Cervical cancer	China	1469	1526	TaqMan genotyping assay	PB	Eqi	H
**Ma et al. 2012 [70]**	Head and Neck Cancer	China	392	892	TaqMan genotyping assay	PB	Diseq	H

HRMA : High resolution melting analysis, SMSG: Sequenome mass spectrometry-based genotyping assay, PCR-RFLP : polymerase chain reaction-restriction fragment length polymorphism, PB : population-based, HB : hospital-based, Eqi : Hardy-Weinberg equilibrium, Diseq : Hardy-Weinberg disequilibrium, H : high quality score, L : low quality score; CLL : Chronic lymphocytic leukemia; CML : Chronic Myeloid Leukemia

Supplementary Table S5. Distribution of samples according to the type of cancer.

Gene/SNP	UB	Breast	Cervical	CLL	CML	CRC	Uterus	Esophagus	Gastric	H&N	HCC	HL	Larynx	Lung	NB	NHL	Oral	PTC	Prostate	RCC
AGO1 rs595961	1481			442										1063						558
AGO1 rs636832	1474			452	781				1130					195						554
AGO2 rs4961280	1454			455										196					673	553
DGCR8 rs1640299	1474	2508		452									483	195						555
DGCR8 rs3757	1472		670					1127					292	1384						554
DGCR8 rs417309	1478	1753		451									482	195						555
DICER1 rs1057035	1415	1729	3003	450		401		1134		3408	3246			1200						
DICER1 rs13078	2874	1756	591	450							356		298	195						555
DICER1 rs3742330	2861		592			2908	159	760	1130	2126	356		293	200		709		250	671	555
DROSHA rs10719	2387	2499		447		808			1130		356			194						498
DROSHA rs6877842	1467			443							356		498	186				250		553
GEMIN3 rs197412	1465	1739		445						2125				182		709	1642			555
GEMIN3 rs197414	1482			416										199					672	556
GEMIN4 rs2740348	1477	2525		450	781	2288		692						182		708			544	554
GEMIN4 rs3744741	1482	2875		453				692						199					544	879
GEMIN4 rs7813	1472	2876						1499	1130					1065		707			1636	880
PIWIL1 rs10773771		2483	2995							3409	3251									
PIWIL1 rs1106042	1399			447						2126				194						498
RAN rs14035	1467		591	441		808					1420		280	183	1313	709	877			554
RAN rs3803012		1754	3000							1283	3228									
TARBP2 rs784567	1466			435									298	195					673	553
XPO5 rs11077	1444		592			1113			279		356	301	284	199		707		2612		553

CLL: chronic lymphocytic leukemia, CRC: colorectal cancer, CML: chronic myeloid leukemia, H&N: head and neck, HCC: hepatocellular carcinoma, HL: Hodgkin lymphoma, NB: neuroblastoma, NHL: non-Hodgkin lymphoma, PTC: papillary thyroid cancer, RCC: renal cell cancer.

Supplementary Table S6. Meta-analysis of the association between DROSHA (A > G; rs10719) variant and cancer risk.

Comparison	Subgroups	No. of studies	Sample size		Test of association				Test of heterogeneity			Publication bias
			Cancer	Control	OR	95% CI	*P*-value	Model	Q test	*P*-value	I^2 (%)	*P*-value (Egger's)
**Allelic model (G allele *versus* A allele)**	**Overall**	10	8224	8414	1.032	0.958-1.113	0.406	R	18.93	0.026	52.45	0.179
	**Geographical region**											
	Asian	6	5622	5626	1.062	0.978-1.154	0.154	F	4.806	0.440	0.0	0.342
	European	2	1030	1412	0.972	0.466-2.025	0.939	R	10.20	0.001	90.19	NA
	American	2	1572	1376	0.906	0.756-1.085	0.281	F	0.420	0.517	0.0	NA
	**Cancer type**											
	Bladder cancer	2	2436	2338	1.039	0.740-1.460	0.824	R	5.889	0.015	83.02	NA
	Breast cancer	2	2520	2478	1.198	0.927-1.549	0.167	R	3.573	0.059	72.01	NA
	CLL	1	204	690	**0.655**	**0.440-0.977**	**0.038**	F	0.0	1.0	0.0	NA
	Colorectal cancer	1	816	800	0.999	0.800-1.247	0.991	F	0.0	1.0	0.0	NA
	Gastric cancer	1	1256	1004	0.970	0.809-1.163	0.741	F	0.0	1.0	0.0	NA
	HCC	1	294	418	1.029	0.734-1.442	0.869	F	0.0	1.0	0.0	NA
	Lung Cancer	1	194	194	0.873	0.553-1.379	0.561	F	0.0	1.0	0.0	NA
	Renal cell carcinoma	1	504	492	0.981	0.725-1.329	0.904	F	0.0	1.0	0.0	NA
	**Genotyping method**											
	TaqMan PCR	4	4092	4622	1.100	0.892-1.356	0.373	R	11.70	0.008	74.36	0.531
	RFLP-PCR	2	1110	1218	1.008	0.837-1.213	0.935	F	0.021	0.885	0.0	NA
	SNPlex technology	2	1572	1376	0.906	0.756-1.085	0.281	F	0.420	0.517	0.0	NA
	Other methods	2	1450	1198	0.956	0.808-1.132	0.601	F	0.176	0.675	0.0	NA
	**Source of controls**											
	Population-based	6	4590	4962	1.002	0.843-1.192	0.978	R	14.01	0.016	64.30	0.413
	Hospital-based	4	3634	3452	1.064	0.958-1.181	0.249	F	4.769	0.190	37.09	0.443
	**HWE in controls**											
	Equilibrium	9	7156	7530	1.048	0.936-1.173	0.414	R	15.88	0.044	49.63	0.192
	Disequilibrium	1	1068	884	0.866	0.692-1.084	0.210	F	0.0	1.0	0.0	NA
	**Quality score**											
	High quality	8	7204	7498	1.001	0.902-1.111	0.985	R	12.08	0.098	42.05	0.089
	Low quality	2	1020	916	1.150	0.736-1.797	0.539	R	3.159	0.076	68.34	NA
**Recessive model (GG versus AA+AG)**	**Overall**	10	4112	4207	**1.192**	**1.005-1.414**	**0.043**	F	10.40	0.319	13.49	0.835
	**Geographical region**											
	Asian	6	2811	2813	**1.223**	**1.001-1.494**	**0.049**	F	3.975	0.553	0.0	0.331
	European	2	515	706	1.589	0.991-2.547	0.054	F	1.325	0.250	24.55	NA
	American	2	786	688	0.809	0.517-1.268	0.356	F	0.761	0.383	0.0	NA
	**Cancer type**											
	Bladder cancer	2	1218	1169	1.048	0.488-2.251	0.905	R	4.629	0.031	78.40	NA
	Breast cancer	2	1260	1239	1.330	0.988-1.789	0.060	F	2.057	0.151	51.39	NA
	CLL	1	102	345	0.936	0.339-2.588	0.899	F	0.0	1.0	0.0	NA
	Colorectal cancer	1	408	400	1.432	0.806-2.547	0.221	F	0.0	1.0	0.0	NA
	Gastric cancer	1	628	502	0.923	0.618-1.378	0.695	F	0.0	1.0	0.0	NA
	HCC	1	147	209	1.746	0.760-4.014	0.189	F	0.0	1.0	0.0	NA
	Lung Cancer	1	97	97	1.315	0.469-3.685	0.603	F	0.0	1.0	0.0	NA
	Renal cell carcinoma	1	252	246	1.044	0.504-2.161	0.907	F	0.0	1.0	0.0	NA
	**Genotyping method**											
	TaqMan PCR	4	2046	2311	**1.357**	**1.071-1.720**	**0.011**	F	2.835	0.418	0.0	0.982
	RFLP-PCR	2	555	609	1.527	0.951-2.452	0.080	F	0.147	0.701	0.0	NA
	SNPlex technology	2	786	688	0.809	0.517-1.268	0.356	F	0.761	0.383	0.0	NA
	Other methods	2	725	599	0.967	0.665-1.405	0.860	F	0.394	0.530	0.0	NA
	**Source of controls**											
	Population-based	6	2295	2481	1.169	0.928-1.473	0.186	F	7.189	0.207	30.45	0.987
	Hospital-based	4	1817	1726	1.220	0.948-1.571	0.122	F	3.154	0.369	4.877	0.713
	**HWE in controls**											
	Equilibrium	9	3578	3765	**1.257**	**1.052-1.503**	**0.012**	F	6.572	0.583	0.0	0.644
	Disequilibrium	1	534	442	0.692	0.391-1.225	0.206	F	0.0	1.0	0.0	NA
	**Quality score**											
	High quality	8	3602	3749	1.129	0.941-1.356	0.192	F	7.499	0.379	6.656	0.978
	Low quality	2	510	458	**1.712**	**1.067-2.748**	**0.026**	F	0.320	0.572	0.0	NA
**Dominant model (AG+GG versus AA)**	**Overall**	10	4112	4207	1.006	0.911-1.112	0.901	R	17.76	0.038	49.32	0.064
	**Geographical region**											
	Asian	6	2811	2813	1.041	0.938-1.157	0.449	F	5.588	0.348	10.52	0.105
	European	2	515	706	0.895	0.364-2.198	0.809	R	10.61	0.001	90.57	NA
	American	2	786	688	0.912	0.736-1.131	0.401	F	0.130	0.719	0.0	NA
	**Cancer type**											
	Bladder cancer	2	1218	1169	1.059	0.762-1.472	0.731	R	3.788	0.052	73.60	NA
	Breast cancer	2	1260	1239	1.156	0.987-1.353	0.072	F	2.267	0.132	55.88	NA
	CLL	1	102	345	**0.554**	**0.346-0.889**	**0.014**	F	0.0	1.0	0.0	NA
	Colorectal cancer	1	408	400	0.917	0.695-1.209	0.540	F	0.0	1.0	0.0	NA
	Gastric cancer	1	628	502	0.976	0.772-1.235	0.842	F	0.0	1.0	0.0	NA
	HCC	1	147	209	0.905	0.593-1.383	0.645	F	0.0	1.0	0.0	NA

	Lung Cancer	1	97	97	0.744	0.421-1.317	0.310	F	0.0	1.0	0.0	NA
	Renal cell carcinoma	1	252	246	0.963	0.668-1.387	0.838	F	0.0	1.0	0.0	NA
	Genotyping method											
	TaqMan PCR	4	2046	2311	1.068	0.822-1.388	0.621	R	11.83	0.008	74.64	0.419
	RFLP-PCR	2	555	609	0.914	0.725-1.152	0.444	F	0.002	0.960	0.0	NA
	SNPlex technology	2	786	688	0.912	0.736-1.131	0.401	F	0.130	0.719	0.0	NA
	Other methods	2	725	599	0.939	0.756-1.166	0.568	F	0.744	0.388	0.0	NA
	Source of controls											
	Population-based	6	2295	2481	0.968	0.792-1.184	0.752	R	12.52	0.028	60.05	0.275
	Hospital-based	4	1817	1726	1.043	0.914-1.191	0.529	F	5.105	0.164	41.23	0.258
	HWE in controls											
	Equilibrium	9	3578	3765	1.005	0.871-1.160	0.945	R	16.48	0.036	51.47	0.071
	Disequilibrium	1	534	442	0.886	0.679-1.156	0.373	F	0.0	1.0	0.0	NA
	Quality score											
	High quality	8	3602	3749	1.000	0.910-1.098	0.993	F	11.95	0.102	41.45	0.029
	Low quality	2	510	458	1.069	0.585-1.953	0.827	R	3.664	0.056	72.70	NA
Homozygote model (GG *versus* AA)	Overall	10	2589	2619	**1.195**	**1.004-1.424**	**0.046**	F	12.28	0.198	26.69	0.809
	Geographical region											
	Asian	6	1694	1686	**1.229**	**1.000-1.510**	**0.050**	F	4.002	0.549	0.0	0.636
	European	2	335	450	1.365	0.531-3.506	0.518	R	2.745	0.100	63.57	NA
	American	2	560	483	0.793	0.503-1.249	0.317	F	0.761	0.383	0.0	NA
	Cancer type											
	Bladder cancer	2	789	765	1.071	0.455-2.524	0.875	R	5.630	0.018	82.24	NA
	Breast cancer	2	760	764	**1.390**	**1.024-1.886**	**0.034**	F	2.616	0.106	61.77	NA
	CLL	1	76	211	0.755	0.270-2.110	0.592	F	0.0	1.0	0.0	NA
	Colorectal cancer	1	254	232	1.346	0.747-2.424	0.323	F	0.0	1.0	0.0	NA
	Gastric cancer	1	371	297	0.919	0.606-1.393	0.690	F	0.0	1.0	0.0	NA
	HCC	1	94	121	1.605	0.684-3.765	0.277	F	0.0	1.0	0.0	NA
	Lung Cancer	1	68	59	1.133	0.394-3.257	0.816	F	0.0	1.0	0.0	NA
	Renal cell carcinoma	1	177	170	1.027	0.491-2.148	0.944	F	0.0	1.0	0.0	NA
	Genotyping method											
	TaqMan PCR	4	1242	1427	**1.410**	**1.106-1.798**	**0.006**	F	4.449	0.217	32.57	0.813
	RFLP-PCR	2	348	353	1.424	0.878-2.312	0.152	F	0.111	0.739	0.0	NA
	SNPlex technology	2	560	483	0.793	0.503-1.249	0.317	F	0.761	0.383	0.0	NA
	Other methods	2	439	356	0.945	0.641-1.392	0.775	F	0.131	0.717	0.0	NA
	Source of controls											
	Population-based	6	1490	1579	1.169	0.923-1.481	0.195	F	8.640	0.124	42.13	0.793
	Hospital-based	4	1099	1040	1.228	0.947-1.592	0.122	F	3.563	0.313	15.81	0.951
	HWE in controls											
	Equilibrium	9	2206	2306	**1.266**	**1.054-1.521**	**0.012**	F	8.172	0.417	2.104	0.919
	Disequilibrium	1	383	313	0.677	0.380-1.206	0.185	F	0.0	1.0	0.0	NA
	Quality score											
	High quality	8	2262	2321	1.125	0.933-1.358	0.217	F	8.325	0.305	15.92	0.705
	Low quality	2	327	298	**1.787**	**1.101-2.902**	**0.019**	F	0.907	0.341	0.0	NA
Heterozygote model (AG *versus* AA)	Overall	10	3793	3936	0.983	0.882-1.095	0.753	R	14.99	0.091	39.98	0.021
	Geographical region											
	Asian	6	2580	2624	1.009	0.904-1.127	0.871	F	5.926	0.313	15.63	0.043
	European	2	466	666	0.839	0.354-1.989	0.690	R	8.726	0.003	88.54	NA
	American	2	747	646	0.937	0.746-1.177	0.575	F	0.008	0.930	0.0	NA
	Cancer type											
	Bladder cancer	2	1141	1103	1.082	0.910-1.286	0.371	F	1.786	0.181	44.01	NA
	Breast cancer	2	1148	1155	1.111	0.941-1.311	0.213	F	1.116	0.291	10.42	NA
	CLL	1	97	327	**0.527**	**0.320-0.870**	**0.012**	F	0.0	1.0	0.0	NA
	Colorectal cancer	1	378	379	0.863	0.647-1.152	0.318	F	0.0	1.0	0.0	NA
	Gastric cancer	1	571	453	0.990	0.773-1.269	0.938	F	0.0	1.0	0.0	NA
	HCC	1	134	198	0.818	0.524-1.277	0.377	F	0.0	1.0	0.0	NA
	Lung Cancer	1	88	90	0.673	0.365-1.238	0.203	F	0.0	1.0	0.0	NA
	Renal cell carcinoma	1	236	231	0.950	0.645-1.400	0.796	F	0.0	1.0	0.0	NA
	Genotyping method											
	TaqMan PCR	4	1875	2170	1.028	0.800-1.321	0.831	R	9.809	0.020	69.42	0.339
	RFLP-PCR	2	512	577	0.850	0.667-1.083	0.187	F	0.040	0.841	0.0	NA
	SNPlex technology	2	747	646	0.937	0.746-1.177	0.575	F	0.008	0.930	0.0	NA
	Other methods	2	659	543	0.937	0.745-1.179	0.581	F	1.324	0.250	24.45	NA
	Source of controls											
	Population-based	6	2126	2326	0.947	0.784-1.145	0.576	R	9.990	0.076	49.95	0.164
	Hospital-based	4	1667	1610	1.011	0.880-1.162	0.878	F	4.931	0.177	39.16	0.180
	HWE in controls											
	Equilibrium	9	3282	3521	0.969	0.840-1.117	0.660	R	14.74	0.064	45.72	0.031
	Disequilibrium	1	511	415	0.930	0.702-1.232	0.613	F	0.0	1.0	0.0	NA
	Quality score											

	High quality	8	3336	3507	0.981	0.889-1.082	0.700	F	10.78	0.148	35.08	0.012
	Low quality	2	457	429	0.980	0.529-1.815	0.949	R	3.374	0.066	70.36	NA

OR: odds ratio, CI: confidence interval, F: fixed model, R: random model, NA: not appreciable

Supplementary Table S7. Meta-analysis of the association between DROSHA (G > C; rs6877842) variant and cancer risk.

**Comparison**	**Subgroups**	**No. of studies**	**Sample size**		**Test of association**				**Test of heterogeneity**			**Publication bias**
			**Cancer**	**Control**	**OR**	**95% CI**	***P*-value**	**Model**	**Q test**	***P*-value**	**I^2 (%)**	***P*-value (Egger's)**
**Allelic model (C allele *versus* G allele)**	**Overall**	8	3400	4106	1.035	0.888-1.207	0.659	R	18.32	0.011	61.79	0.710
	**Geographical region**											
	Asian	3	720	864	0.897	0.582-1.384	0.624	F	3.336	0.189	40.04	**0.082***
	European	3	660	1222	0.894	0.476-1.515	0.580	R	12.95	0.002	84.55	0.715
	American	2	2020	2020	1.058	0.897-1.249	0.502	F	0.012	0.913	0.0	NA
	**Cancer type**											
	Larynx cancer	2	456	540	**0.638**	**0.481-0.847**	**0.002**	F	0.104	0.747	0.0	NA
	Bladder cancer	1	1470	1464	1.064	0.878-1.289	0.526	F	0.0	1.0	0.0	NA
	CLL	1	204	682	**1.505**	**1.035-2.189**	**0.032**	F	0.0	1.0	0.0	NA
	HCC	1	294	418	1.435	0.563-3.660	0.450	F	0.0	1.0	0.0	NA
	Lung cancer	1	186	186	1.323	0.565-3.096	0.520	F	0.0	1.0	0.0	NA
	PTC	1	240	260	0.613	0.337-1.113	0.108	F	0.0	1.0	0.0	NA
	Renal cell carcinoma	1	550	556	1.042	0.750-1.447	0.807	F	0.0	1.0	0.0	NA
	**Genotyping method**											
	TaqMan PCR	3	660	1222	0.849	0.476-1.515	0.580	R	12.95	0.002	84.55	0.715
	RFLP-PCR	2	534	678	0.783	0.474-1.296	0.342	F	2.258	0.133	55.71	NA
	SNPlex technology	2	2020	2020	1.058	0.897-1.249	0.502	F	0.012	0.913	0.0	NA
	Other methods	1	186	186	1.323	0.565-3.096	0.520	F	0.0	1.0	0.0	NA
	**Source of controls**											
	Population-based	6	2958	3580	0.984	0.756-1.280	0.904	R	13.02	0.023	61.59	0.650
	Hospital-based	2	442	526	0.739	0.527-1.035	0.078	F	2.135	0.144	53.16	NA
	**HWE in controls**											
	Equilibrium	8	3400	4106	0.943	0.741-1.199	0.629	R	18.32	0.011	61.79	0.710
	**Quality score**											
	High quality	5	2758	3380	1.088	0.941-1.258	0.253	F	6.896	0.142	41.99	0.953
	Low quality	3	642	726	**0.686**	**0.524-0.897**	**0.006**	F	2.644	0.267	24.36	0.293
**Recessive model (CC versus GG+GC)**	**Overall**	7	1553	1844	1.326	0.835-2.106	0.232	R	13.11	0.041	54.23	0.516
	**Geographical region**											
	Asian	2	213	223	1.056	0.211-5.291	0.948	F	0.002	0.963	0.0	NA
	European	3	330	611	1.057	0.255-4.386	0.939	R	12.64	0.002	84.18	0.359
	American	2	1010	1010	1.398	0.836-2.336	0.201	F	0.307	0.579	0.0	NA
	**Cancer type**											
	Larynx cancer	2	228	270	0.518	0.248-1.085	0.081	F	0.004	0.952	0.0	NA
	Bladder cancer	1	735	732	1.542	0.830-2.867	0.171	F	0.0	1.0	0.0	NA
	CLL	1	102	341	**4.000**	**1.708-9.365**	**0.001**	F	0.0	1.0	0.0	NA
	Lung cancer	1	93	93	1.000	0.062-16.23	1.000	F	0.0	1.0	0.0	NA
	PTC	1	120	130	1.085	0.150-7.824	0.936	F	0.0	1.0	0.0	NA
	Renal cell carcinoma	1	275	278	1.128	0.451-2.820	0.797	F	0.0	1.0	0.0	NA
	**Genotyping method**											
	TaqMan PCR	3	330	611	1.057	0.255-4.386	0.939	R	12.64	0.002	84.18	0.395
	SNPlex technology	2	1010	1010	1.398	0.836-2.336	0.201	F	0.307	0.579	0.0	NA
	Other methods	2	213	223	1.056	0.211-5.291	0.948	F	0.002	0.963	0.0	NA
	**Source of controls**											
	Population-based	5	1332	1581	1.445	0.757-2.757	0.264	R	8.765	0.066	54.52	0.592
	Hospital-based	2	221	263	0.547	0.218-1.375	0.200	F	0.202	0.653	0.0	NA
	**HWE in controls**											
	Equilibrium	7	1553	1844	1.193	0.655-2.174	0.564	R	13.11	0.041	54.23	0.516
	**Quality score**											
	High quality	4	1232	1481	**1.805**	**1.175-2.773**	**0.007**	F	4.875	0.181	38.46	0.883
	Low quality	3	321	363	0.541	0.265-1.105	0.092	F	0.203	0.903	0.0	0.022
**Dominant model (GC+CC versus GG)**	**Overall**	8	1700	2053	0.989	0.832-1.176	0.902	R	13.86	0.054	49.51	0.610
	**Geographical region**											
	Asian	3	360	432	0.880	0.553-1.400	0.589	F	3.968	0.138	49.59	**0.030***
	European	3	330	611	0.745	0.438-1.269	0.279	R	6.987	0.030	71.37	0.596
	American	2	1010	1010	1.028	0.850-1.243	0.776	F	0.001	0.973	0.0	NA
	**Cancer type**											
	Larynx cancer	2	228	270	**0.573**	**0.401-0.819**	**0.002**	F	0.165	0.684	0.0	NA
	Bladder cancer	1	735	732	1.026	0.823-1.279	0.819	F	0.0	1.0	0.0	NA
	CLL	1	102	341	1.247	0.786-1.977	0.348	F	0.0	1.0	0.0	NA
	HCC	1	147	209	1.449	0.561-3.744	0.443	F	0.0	1.0	0.0	NA
	Lung cancer	1	93	93	1.383	0.553-3.458	0.488	F	0.0	1.0	0.0	NA
	PTC	1	120	130	0.550	0.286-1.060	0.074	F	0.0	1.0	0.0	NA

	Renal cell carcinoma	1	275	278	1.034	0.710-1.505	0.862	F	0.0	1.0	0.0	NA
	**Genotyping method**											
	TaqMan PCR	3	330	611	0.745	0.438-1.269	0.279	R	6.987	0.030	71.37	0.596
	RFLP-PCR	2	267	339	0.752	0.439-1.290	0.301	F	2.709	0.100	63.08	NA
	SNPlex technology	2	1010	1010	1.028	0.850-1.243	0.776	F	0.001	0.973	0.0	NA
	Other methods	1	93	93	1.383	0.553-3.458	0.488	F	0.0	1.0	0.0	NA
	**Source of controls**											
	Population-based	6	1479	1790	0.923	0.711-1.200	0.551	R	9.676	0.085	48.33	0.530
	Hospital-based	2	221	263	0.719	0.476-1.087	0.118	F	2.449	0.118	59.17	NA
	**HWE in controls**											
	Equilibrium	8	1700	2053	0.889	0.697-1.134	0.344	R	13.86	0.054	49.51	0.610
	**Quality score**											
	High quality	5	1379	1690	1.023	0.866-1.209	0.788	F	4.668	0.323	14.31	0.872
	Low quality	3	321	363	0.644	0.461-0.898	0.009	F	3.244	0.197	38.35	0.345
**Homozygote model (CC *versus* GG)**	**Overall**	7	1145	1307	1.304	0.815-2.086	0.268	R	15.55	0.016	61.41	0.486
	**Geographical region**											
	Asian	2	187	187	0.993	0.198-4.985	0.993	F	0.001	0.970	0.0	NA
	European	3	220	386	0.900	0.186-4.346	0.896	R	14.81	0.001	86.50	0.334
	American	2	738	734	1.396	0.833-2.339	0.206	F	0.286	0.593	0.0	NA
	**Cancer type**											
	Larynx cancer	2	144	144	**0.413**	**0.193-0.880**	**0.022**	F	0.001	0.978	0.0	NA
	Bladder cancer	1	528	521	1.536	0.823-2.865	0.178	F	0.0	1.0	0.0	NA
	CLL	1	76	242	**3.938**	**1.660-9.340**	**0.002**	F	0.0	1.0	0.0	NA
	Lung cancer	1	82	85	1.037	0.064-16.86	0.980	F	0.0	1.0	0.0	NA
	PTC	1	105	102	0.971	0.134-7.026	0.977	F	0.0	1.0	0.0	NA
	Renal cell carcinoma	1	210	213	1.133	0.451-2.848	0.790	F	0.0	1.0	0.0	NA
	**Genotyping method**											
	TaqMan PCR	3	220	386	0.900	0.186-4.346	0.896	R	14.81	0.001	86.50	0.334
	SNPlex technology	2	738	734	1.396	0.833-2.339	0.206	F	0.286	0.593	0.0	NA
	Other methods	2	187	187	0.993	0.198-4.985	0.993	F	0.001	0.970	0.0	NA
	**Source of controls**											
	Population-based	5	984	1131	1.346	0.665-2.725	0.409	R	10.19	0.037	60.74	0.535
	Hospital-based	2	161	176	0.462	0.180-1.184	0.108	F	0.364	0.546	0.0	NA
	**HWE in controls**											
	Equilibrium	7	1145	1307	1.090	0.561-2.117	0.800	R	15.55	0.016	61.41	0.486
	**Quality score**											
	High quality	4	919	1078	**1.779**	**1.154-2.742**	**0.009**	F	4.744	0.192	36.76	0.850
	Low quality	3	226	229	**0.440**	**0.212-0.913**	**0.028**	F	0.392	0.822	0.0	0.139
**Heterozygote model (GC *versus* GG)**	**Overall**	8	1638	1989	0.893	0.764-1.044	0.156	F	9.965	0.191	29.75	0.615
	**Geographical region**											
	Asian	3	357	429	0.871	0.539-1.409	0.574	F	4.295	0.117	53.43	0.026
	European	3	307	576	**0.705**	**0.522-0.952**	**0.023**	F	2.139	0.343	6.494	0.742
	American	2	974	984	0.994	0.815-1.211	0.949	F	0.023	0.881	0.0	NA
	**Cancer type**											
	Larynx cancer	2	217	246	**0.604**	**0.417-0.875**	**0.008**	F	0.188	0.664	0.0	NA
	Bladder cancer	1	709	715	0.985	0.784-1.237	0.896	F	0.0	1.0	0.0	NA
	CLL	1	90	330	0.948	0.567-1.583	0.838	F	0.0	1.0	0.0	NA
	HCC	1	147	209	1.449	0.561-3.744	0.443	F	0.0	1.0	0.0	NA
	Lung cancer	1	92	92	1.426	0.546-3.726	0.469	F	0.0	1.0	0.0	NA
	PTC	1	118	128	0.520	0.262-1.032	0.061	F	0.0	1.0	0.0	NA
	Renal cell carcinoma	1	265	269	1.020	0.687-1.514	0.922	F	0.0	1.0	0.0	NA
	**Genotyping method**											
	TaqMan PCR	3	307	576	**0.705**	**0.522-0.952**	**0.023**	F	2.139	0.343	6.494	0.742
	RFLP-PCR	2	265	337	0.822	0.303-2.231	0.700	R	2.945	0.086	66.05	NA
	SNPlex technology	2	974	984	0.994	0.815-1.211	0.949	F	0.023	0.881	0.0	NA
	Other methods	1	92	92	1.426	0.546-3.726	0.469	F	0.0	1.0	0.0	NA
	**Source of controls**											
	Population-based	6	1424	1742	0.916	0.775-1.083	0.306	F	7.209	0.206	30.65	0.453
	Hospital-based	2	214	247	0.756	0.492-1.162	0.202	F	2.091	0.148	52.18	NA
	**HWE in controls**											
	Equilibrium	8	1638	1989	0.893	0.764-1.044	0.156	F	9.965	0.191	29.75	0.615
	**Quality score**											
	High quality	5	1329	1651	0.960	0.805-1.143	0.643	F	3.942	0.414	0.0	0.738
	Low quality	3	309	338	**0.675**	**0.477-0.954**	**0.026**	F	2.865	0.239	30.19	0.367

OR: odds ratio, CI: confidence interval, F: fixed model, R: random model, NA: not appreciable

Supplementary Table S8. Meta-analysis of the association between DGCR8 (G > A; rs3757) variant and cancer risk.

Comparison	Subgroups	No. of studies	Sample size		Test of association				Test of heterogeneity			Publication bias
			Cancer	Control	OR	95% CI	*P*-value	Model	Q test	*P*-value	I^2 (%)	*P*-value (Egger's)
**Allelic model (A allele *versus* G allele)**	**Overall**	7	5402	5596	0.977	0.896-1.066	0.606	F	3.068	0.800	0.0	0.868
	**Geographical region**											
	Asian	4	3132	3230	1.036	0.923-1.162	0.550	F	0.510	0.917	0.0	0.972
	European	1	224	340	0.846	0.606-1.181	0.326	F	0.0	1.0	0.0	NA
	American	2	2026	2026	0.915	0.791-1.059	0.234	F	0.089	0.766	0.0	NA
	**Cancer type**											
	Lung cancer	2	1388	1380	1.067	0.880-1.293	0.513	F	0.036	0.849	0.0	NA
	Bladder cancer	1	1474	1470	0.903	0.762-1.071	0.242	F	0.0	1.0	0.0	NA
	Cervical cancer	1	676	664	0.949	0.717-1.256	0.714	F	0.0	1.0	0.0	NA
	ESCC	1	1068	1186	1.045	0.884-1.235	0.605	F	0.0	1.0	0.0	NA
	Larynx cancer	1	244	340	0.846	0.606-1.181	0.326	F	0.0	1.0	0.0	NA
	Renal cell carcinoma	1	552	556	0.949	0.717-1.256	0.716	F	0.0	1.0	0.0	NA
	**Genotyping method**											
	TaqMan PCR	2	1444	1540	0.994	0.833-1.186	0.945	F	1.243	0.265	19.54	NA
	SNPlex technology	2	2026	2026	0.915	0.791-1.059	0.234	F	0.089	0.766	0.0	NA
	Other methods	3	1932	2030	1.026	0.894-1.178	0.716	F	0.451	0.798	0.0	0.996
	**Source of controls**											
	Population-based	3	1864	2082	0.990	0.868-1.129	0.881	F	1.341	0.511	0.0	0.141
	Hospital-based	4	3538	3514	0.968	0.861-1.087	0.579	F	1.662	0.645	0.0	0.460
	**HWE in controls**											
	Equilibrium	5	4090	4070	0.965	0.867-1.074	0.514	F	1.677	0.795	0.0	0.405
	Disequilibrium	2	1312	1526	1.002	0.863-1.163	0.981	F	1.231	0.267	18.77	NA
	**Quality score**											
	High quality	4	3770	3876	0.967	0.873-1.071	0.515	F	1.480	0.687	0.0	0.796
	Low quality	3	1632	1720	1.007	0.852-1.189	0.939	F	1.423	0.491	0.0	0.863
**Recessive model (AA versus GG+GA)**	**Overall**	7	2701	2798	0.812	0.618-1.067	0.135	F	3.085	0.798	0.0	0.831
	**Geographical region**											
	Asian	4	1566	1615	0.972	0644-1.467	0.891	F	0.131	0.988	0.0	0.880
	European	1	122	170	0.350	0.113-1.083	0.068	F	0.0	1.0	0.0	NA
	American	2	1013	1013	0.766	0.522-1.125	0.174	F	0.004	0.952	0.0	NA
	**Cancer type**											
	Lung cancer	2	694	690	1.029	0.593-1.784	0.920	F	0.032	0.857	0.0	NA
	Bladder cancer	1	737	735	0.761	0.492-1.178	0.220	F	0.0	1.0	0.0	NA
	Cervical cancer	1	338	332	0.881	0.353-2.196	0.785	F	0.0	1.0	0.0	NA
	ESCC	1	534	593	0.924	0.396-2.156	0.855	F	0.0	1.0	0.0	NA
	Larynx cancer	1	122	170	0.350	0.113-1.083	0.068	F	0.0	1.0	0.0	NA
	Renal cell carcinoma	1	276	278	0.783	0.349-1.756	0.552	F	0.0	1.0	0.0	NA
	**Genotyping method**											
	TaqMan PCR	2	722	770	0.779	0.449-1.351	0.374	F	2.529	0.112	60.47	NA
	SNPlex technology	2	1013	1013	0.766	0.522-1.125	0.174	F	0.004	0.952	0.0	NA
	Other methods	3	966	1015	0.951	0.552-1.640	0.857	F	0.118	0.943	0.0	0.309
	**Source of controls**											
	Population-based	3	932	1041	0.703	0.418-1.181	0.183	F	1.931	0.381	0.0	0.218
	Hospital-based	4	1769	1757	0.858	0.623-1.182	0.349	F	0.742	0.863	0.0	0.221
	**HWE in controls**											
	Equilibrium	5	2045	2035	0.847	0.629-1.141	0.276	F	0.785	0.940	0.0	0.244
	Disequilibrium	2	656	763	0.651	0.331-1.283	0.215	F	1.814	0.178	44.88	NA
	**Quality score**											
	High quality	4	1885	1938	0.802	0.578-1.112	0.185	F	0.206	0.977	0.0	0.179
	Low quality	3	816	860	0.836	0.510-1.372	0.479	F	2.859	0.239	30.05	0.645
**Dominant model (GA+AA versus GG)**	**Overall**	7	2701	2798	1.000	0.890-1.123	0.997	F	2.823	0.831	0.0	0.733
	**Geographical region**											
	Asian	4	1566	1615	1.075	0.916-1.262	0.377	F	1.028	0.794	0.0	0.866
	European	1	122	170	0.862	0.494-1.502	0.599	F	0.0	1.0	0.0	NA
	American	2	1013	1013	0.929	0.779-1.109	0.417	F	0.078	0.780	0.0	NA
	**Cancer type**											
	Lung cancer	2	694	690	1.086	0.868-1.360	0.470	F	0.022	0.883	0.0	NA
	Bladder cancer	1	737	735	0.915	0.743-1.126	0.402	F	0.0	1.0	0.0	NA
	Cervical cancer	1	338	332	0.948	0.686-1.309	0.745	F	0.0	1.0	0.0	NA
	ESCC	1	534	593	1.197	0.863-1.660	0.281	F	0.0	1.0	0.0	NA
	Larynx cancer	1	122	170	0.862	0.494-1.502	0.599	F	0.0	1.0	0.0	NA
	Renal cell carcinoma	1	276	278	0.968	0.690-1.358	0.851	F	0.0	1.0	0.0	NA
	**Genotyping method**											
	TaqMan PCR	2	722	770	1.041	0.834-1.299	0.722	F	0.529	0.467	0.0	NA
	SNPlex technology	2	1013	1013	0.929	0.779-1.109	0.417	F	0.078	0.780	0.0	NA
	Other methods	3	966	1015	1.072	0.865-1.329	0.526	F	1.027	0.598	0.0	0.867

	Source of controls											
	Population-based	3	932	1041	1.044	0.841-1.296	0.698	F	1.320	0.517	0.0	0.517
	Hospital-based	4	1769	1757	0.982	0.856-1.128	0.801	F	1.288	0.732	0.0	0.556
	HWE in controls											
	Equilibrium	5	2045	2035	0.980	0.863-1.114	0.761	F	1.294	0.862	0.0	0.504
	Disequilibrium	2	656	763	1.100	0.830-1.458	0.508	F	0.998	0.318	0.0	NA
	Quality score											
	High quality	4	1885	1938	0.977	0.849-1.124	0.747	F	1.899	0.594	0.0	0.402
	Low quality	3	816	860	1.051	0.854-1.295	0.636	F	0.595	0.743	0.0	0.697
Homozygote model (AA *versus* GG)	Overall	7	1498	1538	0.824	0.624-1.088	0.171	F	3.573	0.734	0.0	0.929
	Geographical region											
	Asian	4	811	837	1.019	0.670-1.550	0.931	F	0.185	0.980	0.0	0.816
	European	1	33	51	0.331	0.099-1.106	0.073	F	0.0	1.0	0.0	NA
	American	2	654	650	0.754	0.510-1.113	0.155	F	0.009	0.923	0.0	NA
	Cancer type											
	Lung cancer	2	487	496	1.057	0.606-1.842	0.846	F	0.037	0.847	0.0	NA
	Bladder cancer	1	480	474	0.746	0.478-1.163	0.195	F	0.0	1.0	0.0	NA
	Cervical cancer	1	239	232	0.869	0.346-2.178	0.764	F	0.0	1.0	0.0	NA
	ESCC	1	85	109	1.078	0.442-2.629	0.869	F	0.0	1.0	0.0	NA
	Larynx cancer	1	33	51	0.331	0.099-1.106	0.073	F	0.0	1.0	0.0	NA
	Renal cell carcinoma	1	174	176	0.781	0.344-1.771	0.554	F	0.0	1.0	0.0	NA
	Genotyping method											
	TaqMan PCR	2	453	481	0.802	0.457-1.407	0.442	F	2.640	0.104	62.12	NA
	SNPlex technology	2	654	650	0.754	0.510-1.113	0.155	F	0.009	0.923	0.0	NA
	Other methods	3	391	407	1.014	0.580-1.773	0.961	F	0.184	0.912	0.0	0.632
	Source of controls											
	Population-based	3	292	336	0.740	0.431-1.269	0.274	F	2.407	0.300	16.92	0.361
	Hospital-based	4	1206	1202	0.856	0.619-1.184	0.348	F	0.961	0.811	0.0	0.248
	HWE in controls											
	Equilibrium	5	1380	1378	0.845	0.625-1.143	0.275	F	1.002	0.909	0.0	0.255
	Disequilibrium	2	118	160	0.710	0.347-1.455	0.350	F	2.378	0.123	57.95	NA
	Quality score											
	High quality	4	978	991	0.807	0.578-1.126	0.208	F	0.557	0.906	0.0	0.242
	Low quality	3	520	547	0.862	0.521-1.429	0.565	F	2.970	0.226	32.66	0.616
Heterozygote model (GA *versus* GG)	Overall	7	2602	2672	1.021	0.905-1.152	0.737	F	1.990	0.921	0.0	0.740
	Geographical region											
	Asian	4	1520	1567	1.082	0.917-1.276	0.353	F	0.930	0.818	0.0	0.933
	European	1	118	155	0.928	0.530-1.627	0.795	F	0.0	1.0	0.0	NA
	American	2	964	950	0.960	0.798-1.155	0.663	F	0.053	0.818	0.0	NA
	Cancer type											
	Lung cancer	2	667	664	1.090	0.863-1.378	0.470	F	0.010	0.920	0.0	NA
	Bladder cancer	1	699	686	0.947	0.762-1.177	0.623	F	0.0	1.0	0.0	NA
	Cervical cancer	1	329	322	0.956	0.685-1.334	0.789	F	0.0	1.0	0.0	NA
	ESCC	1	524	581	1.200	0.865-1.665	0.276	F	0.0	1.0	0.0	NA
	Larynx cancer	1	118	155	0.928	0.530-1.627	0.795	F	0.0	1.0	0.0	NA
	Renal cell carcinoma	1	265	264	0.994	0.700-1.411	0.973	F	0.0	1.0	0.0	NA
	Genotyping method											
	TaqMan PCR	2	698	735	1.057	0.841-1.330	0.633	F	0.248	0.618	0.0	NA
	SNPlex technology	2	964	950	0.960	0.798-1.155	0.663	F	0.053	0.818	0.0	NA
	Other methods	3	940	987	1.079	0.866-1.344	0.500	F	0.928	0.629	0.0	0.929
	Source of controls											
	Population-based	3	907	1000	1.071	0.859-1.334	0.543	F	0.886	0.642	0.0	0.528
	Hospital-based	4	1695	1672	1.000	0.866-1.155	0.997	F	0.846	0.838	0.0	0.633
	HWE in controls											
	Equilibrium	5	1960	1936	0.999	0.875-1.142	0.992	F	0.848	0.932	0.0	0.576
	Disequilibrium	2	642	736	1.124	0.847-1.492	0.418	F	0.599	0.439	0.0	NA
	Quality score											
	High quality	4	1817	1853	1.002	0.867-1.158	0.981	F	1.502	0.682	0.0	0.505
	Low quality	3	785	819	1.065	0.858-1.321	0.571	F	0.279	0.870	0.0	0.717

OR: odds ratio, CI: confidence interval, F: fixed model, R: random model, NA: not appreciable

Supplementary Table S9. Meta-analysis of the association between DGCR8 (G > A; rs417309) variant and cancer risk.

Comparison	Subgroups	No. of studies	Sample size		Test of association				Test of heterogeneity			Publication bias
			Cancer	Control	OR	95% CI	*P*-value	Model	Q test	*P*-value	I^2 (%)	*P*-value (Egger's)
**Allelic model (A allele *versus* G allele)**	**Overall**	7	4584	5244	**1.260**	**1.079-1.472**	**0.003**	F	8.628	0.196	30.46	0.318
	**Geographical region**											
	Asian	2	1916	1980	1.336	0.982-1.817	0.065	F	0.807	0.369	0.0	NA
	European	3	634	1232	**1.376**	**1.032-1.835**	**0.030**	F	6.607	0.037	69.73	0.231
	American	2	2034	2032	1.153	0.916-1.451	0.224	F	0.148	0.701	0.0	NA
	**Cancer type**											
	Larynx cancer	2	424	540	**1.603**	**1.175-2.188**	**0.003**	F	0.030	0.863	0.0	NA
	Bladder Cancer	1	1480	1476	1.183	0.909-1.539	0.212	F	0.0	1.0	0.0	NA
	Breast cancer	1	1720	1786	**1.400**	**1.012-1.936**	**0.042**	F	0.0	1.0	0.0	NA
	CLL	1	210	692	0.544	0.253-1.169	0.119	F	0.0	1.0	0.0	NA
	Lung cancer	1	196	194	0.875	0.330-2.316	0.788	F	0.0	1.0	0.0	NA
	Renal cell carcinoma	1	554	556	1.064	0.664-1.705	0.797	F	0.0	1.0	0.0	NA
	**Genotyping method**											
	TaqMan PCR	4	2354	3018	1.319	0.937-1.856	0.112	R	6.613	0.085	54.63	0.282
	SNPlex technology	2	2034	2032	1.153	0.916-1.451	0.224	F	0.148	0.701	0.0	NA
	Other methods	1	196	194	0.875	0.330-2.316	0.788	F	0.0	1.0	0.0	NA
	**Source of controls**											
	Population-based	5	2908	3574	1.325	1.089-1.611	0.005	F	7.616	0.107	47.48	0.163
	Hospital-based	2	1676	1670	1.159	0.899-1.494	0.256	F	0.343	0.558	0.0	NA
	**HWE in controls**											
	Equilibrium	7	4584	5244	**1.260**	**1.079-1.472**	**0.003**	F	8.628	0.196	30.46	0.318
	**Quality score**											
	High quality	4	3964	4510	1.175	0.979-1.410	0.083	F	5.186	0.159	42.15	0.185
	Low quality	3	620	734	**1.516**	**1.127-2.038**	**0.006**	F	1.380	0.502	0.0	0.238
**Recessive model (AA versus GG+GA)**	**Overall**	5	1334	1632	**2.582**	**1.478-4.511**	**0.001**	F	0.948	0.918	0.0	0.722
	**Geographical region**											
	European	3	317	616	**2.636**	**1.326-5.239**	**0.006**	F	0.700	0.705	0.0	0.410
	American	2	1017	1016	2.481	0.954-6.457	0.062	F	0.237	0.626	0.0	NA
	**Cancer type**											
	Larynx cancer	2	212	270	**2.878**	**1.400-5.915**	**0.004**	F	0.075	0.784	0.0	NA
	Bladder Cancer	1	740	738	2.212	0.765-6.398	0.143	F	0.0	1.0	0.0	NA
	CLL	1	105	346	1.099	0.113-10.68	0.935	F	0.0	1.0	0.0	NA
	Renal cell carcinoma	1	277	278	4.059	0.451-36.54	0.212	F	0.0	1.0	0.0	NA
	**Genotyping method**											
	TaqMan PCR	3	317	616	**2.636**	**1.326-5.239**	**0.006**	F	0.700	0.705	0.0	0.410
	SNPlex technology	2	1017	1016	2.481	0.954-6.457	0.062	F	0.237	0.626	0.0	NA
	**Source of controls**											
	Population-based	4	594	894	**2.739**	**1.422-5.276**	**0.003**	F	0.835	0.841	0.0	0.697
	Hospital-based	1	740	738	2.212	0.765-6.398	0.143	F	0.0	1.0	0.0	NA
	**HWE in controls**											
	Equilibrium	5	1334	1632	**2.582**	**1.478-4.511**	**0.001**	F	0.948	0.918	0.0	0.722
	**Quality score**											
	High quality	3	1122	1362	2.196	0.909-5.301	0.080	F	0.656	0.720	0.0	0.956
	Low quality	2	212	270	**2.878**	**1.400-5.915**	**0.004**	F	0.075	0.784	0.0	NA
**Dominant model (GA+AA versus GG)**	**Overall**	7	2292	2622	**1.194**	**1.009-1.415**	**0.040**	F	7.720	0.259	22.28	0.277
	**Geographical region**											
	Asian	2	958	990	1.355	0.989-1.856	0.059	F	0.846	0.358	0.0	NA
	European	3	317	616	1.210	0.858-1.707	0.278	F	5.510	0.064	63.70	0.231
	American	2	1017	1016	1.098	0.858-1.406	0.458	F	0.299	0.585	0.0	NA
	**Cancer type**											
	Larynx cancer	2	212	270	**1.460**	**1.000-2.131**	**0.050**	F	0.005	0.943	0.0	NA
	Bladder Cancer	1	740	738	1.141	0.860-1.515	0.360	F	0.0	1.0	0.0	NA
	Breast cancer	1	860	893	**1.423**	**1.021-1.983**	**0.037**	F	0.0	1.0	0.0	NA
	CLL	1	105	346	0.490	0.214-1.124	0.092	F	0.0	1.0	0.0	NA
	Lung cancer	1	98	97	0.869	0.321-2.355	0.783	F	0.0	1.0	0.0	NA
	Renal cell carcinoma	1	277	278				F	0.0	1.0	0.0	NA
	**Genotyping method**											
	TaqMan PCR	4	1177	1509	**1.316**	**1.036-1.671**	**0.024**	F	5.952	0.114	49.60	0.280
	SNPlex technology	2	1017	1016	1.098	0.858-1.406	0.458	F	0.299	0.585	0.0	NA
	Other methods	1	98	97	0.869	0.321-2.355	0.783	F	0.0	1.0	0.0	NA
	**Source of controls**											
	Population-based	5	1454	1787	**1.245**	**1.003-1.545**	**0.047**	F	7.090	0.131	43.58	0.179
	Hospital-based	2	838	835	1.118	0.852-1.469	0.421	F	0.266	0.606	0.0	NA
	**HWE in controls**											
	Equilibrium	7	2292	2622	**1.194**	**1.009-1.415**	**0.040**	F	7.720	0.259	22.28	0.277
	**Quality score**											

	High quality	4	1982	2255	1.147	0.946-1.391	0.162	F	6.075	0.108	50.61	0.171
	Low quality	3	310	367	1.368	0.960-1.948	0.083	F	0.915	0.633	0.0	0.208
**Homozygote model (AA *versus* GG)**	**Overall**	5	1129	1382	**2.659**	**1.514-4.669**	**0.001**	F	1.105	0.894	0.0	0.637
	**Geographical region**											
	European	3	251	502	2.747	1.369-5.512	0.004	F	0.863	0.649	0.0	0.382
	American	2	878	880	2.499	0.960-6.507	0.061	F	0.217	0.641	0.0	NA
	**Cancer type**											
	Larynx cancer	2	152	197	**3.042**	**1.464-6.321**	**0.003**	F	0.071	0.791	0.0	NA
	Bladder Cancer	1	631	636	2.239	0.773-6.482	0.137	F	0.0	1.0	0.0	NA
	CLL	1	99	305	1.027	0.106-9.989	0.982	F	0.0	1.0	0.0	NA
	Renal cell carcinoma	1	247	244	4.000	0.444-36.05	0.217	F	0.0	1.0	0.0	NA
	**Genotyping method**											
	TaqMan PCR	3	251	502	**2.747**	**1.369-5.512**	**0.004**	F	0.863	0.649	0.0	0.382
	SNPlex technology	2	878	880	2.499	0.960-6.507	**0.061**	F	0.217	0.641	0.0	NA
	**Source of controls**											
	Population-based	4	498	746	**2.843**	**1.464-5.522**	**0.002**	F	0.965	0.810	0.0	0.609
	Hospital-based	1	631	636	2.239	0.773-6.482	0.137	F	0.0	1.0	0.0	NA
	**HWE in controls**											
	Equilibrium	5	1129	1382	**2.659**	**1.514-4.669**	**0.001**	F	1.105	0.894	0.0	0.637
	**Quality score**											
	High quality	3	977	1185	2.186	0.905-5.282	0.082	F	0.716	0.699	0.0	0.916
	Low quality	2	152	197	**3.042**	**1.464-6.321**	**0.003**	F	0.071	0.791	0.0	NA
**Heterozygote model (GA *versus* GG)**	**Overall**	7	2251	2601	1.118	0.939-1.332	0.211	F	7.242	0.299	17.15	0.151
	**Geographical region**											
	Asian	2	958	990	1.355	0.989-1.856	0.059	F	0.846	0.358	0.0	NA
	European	3	291	601	1.008	0.694-1.465	0.965	F	3.849	0.146	48.03	0.203
	American	2	1002	1010	1.035	0.802-1.335	0.793	F	0.469	0.493	0.0	NA
	**Cancer type**											
	Larynx cancer	2	187	258	1.199	0.795-1.810	0.386	F	0.001	0.981	0.0	NA
	Bladder Cancer	1	729	733	1.088	0.812-1.456	0.573	F	0.0	1.0	0.0	NA
	Breast cancer	1	860	893	**1.423**	**1.021-1.983**	**0.037**	F	0.0	1.0	0.0	NA
	CLL	1	104	343	0.451	0.186-1.094	0.078	F	0.0	1.0	0.0	NA
	Lung cancer	1	98	97	0.869	0.321-2.355	0.783	F	0.0	1.0	0.0	NA
	Renal cell carcinoma	1	273	277	0.882	0.523-1.487	0.638	F	0.0	1.0	0.0	NA
	**Genotyping method**											
	TaqMan PCR	4	1151	1494	1.222	0.954-1.566	0.113	F	5.676	0.129	47.14	0.127
	SNPlex technology	2	1002	1010	1.035	0.802-1.335	0.793	F	0.469	0.493	0.0	NA
	Other methods	1	98	97	0.869	0.321-2.355	0.783	F	0.0	1.0	0.0	NA
	**Source of controls**											
	Population-based	5	1424	1771	1.151	0.920-1.440	0.218	F	6.897	0.141	42.01	0.073
	Hospital-based	2	827	830	1.068	0.807-1.414	0.643	F	0.179	0.672	0.0	NA
	**HWE in controls**											
	Equilibrium	7	2251	2601	1.118	0.939-1.332	0.211	F	7.242	0.299	17.15	0.151
	**Quality score**											
	High quality	4	1966	2246	1.030	0.738-1.437	0.864	R	6.880	0.076	56.40	0.175
	Low quality	3	285	355	1.144	0.782-1.674	0.487	F	0.343	0.842	0.0	0.148

OR: odds ratio, CI: confidence interval, F: fixed model, R: random model, NA: not appreciable

Supplementary Table S10. Meta-analysis of the association between DGCR8 (T > G; rs1640299) variant and cancer risk.

Comparison	Subgroups	No. of studies	Sample size		Test of association				Test of heterogeneity			Publication bias
			Cancer	Control	OR	95% CI	*P*-value	Model	Q test	*P*-value	I^2 (%)	*P*-value (Egger's)
**Allelic model (G allele *versus* T allele)**	**Overall**	8	5380	5954	0.974	0.874-1.085	0.627	R	25.69	0.001	72.75	0.087
	**Geographical region**											
	Asian	2	1894	1976	0.957	0.829-1.104	0.545	F	0.054	0.816	0.0	NA
	European	4	1458	1948	0.808	0.524-1.246	0.335	R	22.60	< 0.001	86.73	0.038
	American	2	2028	2030	1.051	0.929-1.189	0.432	F	1.766	0.184	43.37	NA
	**Cancer type**											
	Breast cancer	2	2524	2492	1.133	0.810-1.585	0.465	R	6.910	0.009	85.53	NA
	Larynx cancer	2	426	540	**0.623**	**0.471-0.823**	**0.001**	F	0.040	0.842	0.0	NA
	Bladder cancer	1	1474	1474	1.106	0.957-1.278	0.172	F	0.0	1.0	0.0	NA
	CLL	1	206	698	0.744	0.543-1.018	0.064	F	0.0	1.0	0.0	NA
	Lung cancer	1	196	194	0.908	0.574-1.438	0.682	F	0.0	1.0	0.0	NA
	Renal cell carcinoma	1	554	556	0.917	0.725-1.161	0.472	F	0.0	1.0	0.0	NA
	**Genotyping method**											
	TaqMan PCR	4	2330	3020	**0.761**	**0.596-0.971**	**0.028**	R	8.085	0.044	62.89	0.020
	SNPlex technology	2	2028	2030	1.051	0.929-1.189	0.432	F	1.766	0.184	43.37	NA
	Other methods	2	1022	904	**1.267**	**1.050-1.529**	**0.013**	F	2.424	0.120	58.74	NA
	**Source of controls**											
	Population-based	6	3710	4286	0.872	0.697-1.090	0.229	R	22.72	< 0.001	77.99	0.159
	Hospital-based	2	1670	1668	1.087	0.947-1.248	0.238	F	0.642	0.423	0.0	NA
	**HWE in controls**											
	Equilibrium	7	5154	5614	0.969	0.828-1.134	0.692	R	18.54	0.005	67.63	0.205
	Disequilibrium	1	226	340	**0.608**	**0.420-0.880**	**0.008**	F	0.0	1.0	0.0	NA
	**Quality score**											
	High quality	4	3932	4510	0.988	0.902-1.083	0.796	F	5.988	0.112	49.90	0.140
	Low quality	4	1448	1444	0.848	0.542-1.326	0.469	R	19.62	< 0.001	84.71	0.140
**Recessive model (GG versus TT+TG)**	**Overall**	8	2690	2977	1.029	0.888-1.192	0.706	F	7.459	0.383	6.150	0.189
	**Geographical region**											
	Asian	2	947	988	1.011	0.702-1.455	0.955	F	0.344	0.558	0.0	NA
	European	4	729	974	1.002	0.751-1.335	0.992	F	3.678	0.298	18.44	0.063
	American	2	1014	1015	0.973	0.647-1.462	0.894	R	3.363	0.067	70.26	NA
	**Cancer type**											
	Breast cancer	2	1262	1246	1.103	0.840-1.449	0.481	F	0.800	0.371	0.0	NA
	Larynx cancer	2	213	270	0.585	0.285-1.201	0.144	F	0.059	0.809	0.0	NA
	Bladder cancer	1	737	737	1.163	0.929-1.457	0.187	F	0.0	1.0	0.0	NA
	CLL	1	103	349	0.884	0.519-1.506	0.651	F	0.0	1.0	0.0	NA
	Lung cancer	1	98	97	1.415	0.433-4.623	0.565	F	0.0	1.0	0.0	NA
	Renal cell carcinoma	1	277	278	0.764	0.518-1.127	0.175	F	0.0	1.0	0.0	NA
	**Genotyping method**											
	TaqMan PCR	4	1165	1510	0.875	0.658-1.164	0.359	F	1.573	0.665	0.0	0.042
	SNPlex technology	2	1014	1015	0.973	0.647-1.462	0.894	R	3.363	0.067	70.26	NA
	Other methods	2	511	452	1.266	0.876-1.831	0.210	F	0.038	0.846	0.0	NA
	**Source of controls**											
	Population-based	6	1855	2143	0.927	0.761-1.130	0.454	F	4.972	0.419	0.0	0.214
	Hospital-based	2	835	834	1.171	0.939-1.461	0.161	F	0.102	0.750	0.0	NA
	**HWE in controls**											
	Equilibrium	7	2577	2807	1.045	0.900-1.213	0.566	F	5.720	0.455	0.0	0.405
	Disequilibrium	1	113	170	0.540	0.205-1.424	0.213	F	0.0	1.0	0.0	NA
	**Quality score**											
	High quality	4	1966	2255	1.017	0.862-1.199	0.845	F	3.758	0.289	20.17	0.187
	Low quality	4	724	722	1.078	0.777-1.497	0.652	F	3.602	0.308	16.71	0.362
**Dominant model (TG+GG versus TT)**	**Overall**	8	2690	2977	0.984	0.861-1.124	0.814	R	31.85	< 0.001	78.02	0.167
	**Geographical region**											
	Asian	2	947	988	0.931	0.778-1.114	0.434	F	0.321	0.571	0.0	NA
	European	4	729	974	0.709	0.355-1.416	0.330	R	29.65	< 0.001	89.88	0.035
	American	2	1014	1015	1.093	0.888-1.345	0.403	F	0.101	0.751	0.0	NA
	**Cancer type**											
	Breast cancer	2	1262	1246	1.234	0.718-2.122	0.447	R	9.527	0.002	89.50	NA
	Larynx cancer	2	213	270	**0.505**	**0.349-0.729**	**< 0.001**	F	0.017	0.897	0.0	NA
	Bladder cancer	1	737	737	1.116	0.873-1.427	0.381	F	0.0	1.0	0.0	NA
	CLL	1	103	349	**0.551**	**0.345-0.880**	**0.013**	F	0.0	1.0	0.0	NA
	Lung cancer	1	98	97	0.797	0.452-1.405	0.433	F	0.0	1.0	0.0	NA
	Renal cell carcinoma	1	277	278	1.036	0.701-1.531	0.861	F	0.0	1.0	0.0	NA
	**Genotyping method**											
	TaqMan PCR	4	1165	1510	**0.634**	**0.426-0.942**	**0.024**	R	11.53	0.009	73.99	0.013
	SNPlex technology	2	1014	1015	1.093	0.888-1.345	0.403	F	0.101	0.751	0.0	NA
	Other methods	2	511	452	1.195	0.589-2.423	0.622	R	4.950	0.026	79.80	NA

	**Source of controls**											
	Population-based	6	1855	2143	0.815	0.569-1.169	0.267	R	29.96	< 0.001	83.31	0.282
	Hospital-based	2	835	834	1.058	0.845-1.326	0.622	F	1.141	0.285	12.39	NA
	**HWE in controls**											
	Equilibrium	7	2577	2807	0.932	0.720-1.208	0.596	R	23.95	0.001	74.95	0.321
	Disequilibrium	1	113	170	**0.494**	**0.305-0.802**	**0.004**	F	0.0	1.0	0.0	NA
	**Quality score**											
	High quality	4	1966	2255	0.930	0.741-1.168	0.534	R	7.002	0.072	57.15	0.482
	Low quality	4	724	722	0.778	0.401-1.513	0.460	R	24.77	< 0.001	87.89	0.100
**Homozygote model (GG *versus* TT)**	**Overall**	8	1477	1598	0.986	0.775-1.254	0.907	R	15.76	0.027	55.59	0.114
	**Geographical region**											
	Asian	2	584	591	0.978	0.674-1.421	0.909	F	0.181	0.671	0.0	NA
	European	4	384	491	0.707	0.329-1.519	0.374	R	13.48	0.004	77.75	0.129
	American	2	509	516	1.100	0.859-1.408	0.450	F	1.669	0.196	40.08	NA
	**Cancer type**											
	Breast cancer	2	719	736	1.249	0.726-2.148	0.422	R	3.505	0.061	71.47	NA
	Larynx cancer	2	124	122	**0.415**	**0.197-0.876**	**0.021**	F	0.062	0.803	0.0	NA
	Bladder cancer	1	383	374	1.213	0.909-1.617	0.189	F	0.0	1.0	0.0	NA
	CLL	1	60	167	0.600	0.327-1.101	0.099	F	0.0	1.0	0.0	NA
	Lung cancer	1	65	57	1.255	0.375-4.197	0.712	F	0.0	1.0	0.0	NA
	Renal cell carcinoma	1	126	142	0.838	0.519-1.355	0.472	F	0.0	1.0	0.0	NA
	**Genotyping method**											
	TaqMan PCR	4	703	823	0.743	0.550-1.004	0.053	F	4.422	0.219	32.15	0.056
	SNPlex technology	2	509	516	1.100	0.859-1.408	0.450	F	1.669	0.196	40.08	NA
	Other methods	2	265	259	**1.608**	**1.075-2.404**	**0.021**	F	0.182	0.670	0.0	NA
	**Source of controls**											
	Population-based	6	1029	1167	0.827	0.557-1.229	0.347	R	13.77	0.017	63.70	0.096
	Hospital-based	2	448	431	1.215	0.918-1.607	0.173	F	0.003	0.957	0.0	NA
	**HWE in controls**											
	Equilibrium	7	1411	1521	1.007	0.765-1.325	0.963	R	11.83	0.066	49.28	0.274
	Disequilibrium	1	66	77	0.381	0.140-1.040	0.060	F	0.0	1.0	0.0	NA
	**Quality score**											
	High quality	4	1088	1217	0.995	0.816-1.212	0.957	F	5.019	0.170	40.22	0.004
	Low quality	4	389	381	0.834	0.369-1.886	0.662	R	10.03	0.018	70.08	0.203
**Heterozygote model (TG *versus* TT)**	**Overall**	8	2235	2477	0.974	0.847-1.120	0.716	R	28.60	< 0.001	75.52	0.191
	**Geographical region**											
	Asian	2	886	925	0.923	0.766-1.113	0.403	F	0.596	0.440	0.0	NA
	European	4	622	815	0.712	0.359-1.415	0.333	R	26.33	< 0.001	88.61	0.032
	American	2	727	737	1.085	0.870-1.353	0.468	F	0.090	0.765	0.0	NA
	**Cancer type**											
	Breast cancer	2	1135	1136	1.230	0.716-2.112	0.454	R	8.516	0.004	88.26	NA
	Larynx cancer	2	201	245	**0.520**	**0.356-0.760**	**0.001**	F	0.005	0.942	0.0	NA
	Bladder cancer	1	511	534	1.062	0.818-1.380	0.651	F	0.0	1.0	0.0	NA
	CLL	1	81	267	**0.528**	**0.318-0.877**	**0.014**	F	0.0	1.0	0.0	NA
	Lung cancer	1	91	92	0.740	0.408-1.339	0.320	F	0.0	1.0	0.0	NA
	Renal cell carcinoma	1	216	203	1.144	0.758-1.729	0.521	F	0.0	1.0	0.0	NA
	**Genotyping method**											
	TaqMan PCR	4	1077	1345	**0.639**	**0.431-0.947**	**0.026**	R	10.30	0.016	70.87	0.011
	SNPlex technology	2	727	737	1.085	0.870-1.353	0.468	F	0.090	0.765	0.0	NA
	Other methods	2	431	395	1.149	0.528-2.504	0.726	R	5.429	0.020	81.58	NA
	**Source of controls**											
	Population-based	6	1633	1851	0.833	0.580-1.197	0.323	R	27.27	< 0.001	81.67	0.327
	Hospital-based	2	602	626	1.002	0.788-1.272	0.990	F	1.195	0.274	16.33	NA
	**HWE in controls**											
	Equilibrium	7	2128	2323	0.930	0.715-1.209	0.588	R	22.16	0.001	72.92	0.348
	Disequilibrium	1	107	154	0.514	0.312-0.847	0.009	F	0.0	1.0	0.0	NA
	**Quality score**											
	High quality	4	1603	1837	0.932	0.738-1.179	0.558	R	6.622	0.085	54.69	0.570
	Low quality	4	632	640	0.777	0.404-1.491	0.447	R	21.94	< 0.001	86.33	0.088

OR: odds ratio, CI: confidence interval, F: fixed model, R: random model, NA: not appreciable

Supplementary Table S11. Meta-analysis of the association between XPO5 (T > G; rs11077) variant and cancer risk.

Comparison	Subgroups	No. of studies	Sample size		Test of association				Test of heterogeneity			Publication bias
			Cancer	Control	OR	95% CI	*P*-value	Model	Q test	*P*-value	I^2 (%)	*P*-value (Egger's)
**Allelic model (G allele *versus* T allele)**	**Overall**	13	7804	9076	1.018	0.939-1.105	0.660	R	35.72	< 0.001	66.40	0.646
	**Geographical region**											
	Asian	9	5212	5692	**1.168**	**1.032-1.322**	**0.014**	F	8.230	0.411	2.794	0.083
	European	1	248	320	**0.458**	**0.326-0.643**	**< 0.001**	F	0.0	1.0	0.0	NA
	American	3	2344	3064	1.003	0.897-1.122	0.958	F	1.440	0.487	0.0	0.389
	**Cancer type**											
	PTC	2	2508	2716	**1.282**	**1.081-1.519**	**0.004**	F	0.285	0.593	0.0	NA
	Colorectal cancer	2	1142	1084	1.098	0.810-1.488	0.548	F	0.442	0.506	0.0	NA
	Bladder cancer	1	1436	1452	0.954	0.823-1.107	0.535	F	0.0	1.0	0.0	NA
	Cervical precancerous lesions	1	592	592	1.445	0.885-2.358	0.141	F	0.0	1.0	0.0	NA
	Gastric cancer	1	274	284	0.984	0.513-1.886	0.960	F	0.0	1.0	0.0	NA
	HCC	1	294	418	0.653	0.370-1.152	0.141	F	0.0	1.0	0.0	NA
	Hodgkin lymphoma	1	202	400	1.091	0.773-1.540	0.621	F	0.0	1.0	0.0	NA
	Larynx cancer	1	248	320	**0.458**	**0.326-0.643**	**< 0.001**	F	0.0	1.0	0.0	NA
	Lung cancer	1	200	198	0.779	0.355-1.709	0.533	F	0.0	1.0	0.0	NA
	NHL	1	356	1058	1.011	0.793-1.289	0.931	F	0.0	1.0	0.0	NA
	Renal cell carcinoma	1	552	554	1.133	0.892-1.438	0.305	F	0.0	1.0	0.0	NA
	**Genotyping method**											
	TaqMan PCR	3	2718	3176	0.863	0.475-1.567	0.628	R	25.74	< 0.001	92.23	0.502
	RFLP-PCR	3	1350	1478	1.080	0.743-1.570	0.687	R	4.944	0.084	59.55	0.258
	PCR-ligase detection	2	600	568	0.944	0.600-1.488	0.805	F	0.029	0.865	0.0	NA
	SNPlex technology	2	1988	2006	1.001	0.882-1.135	0.989	F	1.435	0.231	30.32	NA
	Other methods	3	1148	1848	1.059	0.859-1.307	0.590	F	2.272	0.321	11.96	0.947
	**Source of controls**											
	Population-based	8	2728	3112	0.956	0.710-1.286	0.764	R	29.26	< 0.001	76.07	0.790
	Hospital-based	5	5076	5964	1.051	0.950-1.161	0.335	F	5.724	0.221	30.12	0.997
	**HWE in controls**											
	Equilibrium	10	6804	8004	1.070	0.979-1.170	0.135	F	12.02	0.212	25.15	0.846
	Disequilibrium	3	1000	1072	0.741	0.375-1.464	0.388	R	18.33	< 0.001	89.09	0.740
	**Quality score**											
	High quality	8	6554	7590	1.082	0.991-1.181	0.077	F	11.85	0.106	40.92	0.604
	Low quality	5	1250	1486	0.794	0.523-1.205	0.278	R	13.67	0.008	70.74	0.660
**Recessive model (GG versus TT+TG)**	**Overall**	13	3902	4538	0.980	0.797-1.204	0.846	R	38.19	< 0.001	68.58	0.849
	**Geographical region**											
	Asian	9	2606	2846	**1.439**	**1.007-2.056**	**0.045**	F	4.373	0.822	0.0	0.035
	European	1	124	160	**0.252**	**0.148-0.430**	**< 0.001**	F	0.0	1.0	0.0	NA
	American	3	1172	1532	1.091	0.886-1.345	0.412	F	3.411	0.182	41.36	NA
	**Cancer type**											
	PTC	2	1254	1358	**1.700**	**1.053-2.742**	**0.030**	F	0.001	0.975	0.0	NA
	Colorectal cancer	2	571	542	0.626	0.102-3.858	0.613	F	0.095	0.759	0.0	NA
	Bladder cancer	1	718	726	0.934	0.707-1.235	0.634	F	0.0	1.0	0.0	NA
	Cervical precancerous lesions	1	296	296	2.007	0.181-22.25	0.570	F	0.0	1.0	0.0	NA
	Gastric cancer	1	137	142	1.037	0.064-16.74	0.980	F	0.0	1.0	0.0	NA
	HCC	1	147	209	0.471	0.019-11.65	0.646	F	0.0	1.0	0.0	NA
	Hodgkin lymphoma	1	101	200	1.378	0.748-2.540	0.304	F	0.0	1.0	0.0	NA
	Larynx cancer	1	124	160	**0.252**	**0.148-0.430**	**< 0.001**	F	0.0	1.0	0.0	NA
	Lung cancer	1	100	99	0.137	0.007-2.691	0.191	F	0.0	1.0	0.0	NA
	NHL	1	178	529	1.167	0.754-1.808	0.488	F	0.0	1.0	0.0	NA
	Renal cell carcinoma	1	276	277	1.530	0.972-2.408	0.066	F	0.0	1.0	0.0	NA
	**Genotyping method**											
	TaqMan PCR	3	1359	1588	0.822	0.234-2.881	0.759	R	23.89	< 0.001	91.63	0.376
	RFLP-PCR	3	675	739	1.521	0.847-2.731	0.160	F	1.508	0.470	0.0	0.113
	PCR-ligase detection	2	300	284	0.950	0.133-6.789	0.959	F	0.008	0.931	0.0	NA
	SNPlex technology	2	994	1003	1.156	0.716-1.866	0.553	R	3.293	0.070	69.63	NA
	Other methods	3	574	924	1.136	0.742-1.740	0.557	F	2.168	0.338	7.738	0.683
	**Source of controls**											
	Population-based	8	1364	1556	0.987	0.471-2.067	0.972	R	33.57	< 0.001	79.15	0.975
	Hospital-based	5	2538	2982	1.024	0.819-1.281	0.832	F	4.506	0.342	11.23	0.691
	**HWE in controls**											
	Equilibrium	10	3402	4002	1.124	0.923-1.369	0.244	F	6.010	0.739	0.0	0.609
	Disequilibrium	3	500	536	0.482	0.099-2.347	0.366	R	26.70	< 0.001	92.51	0.780
	**Quality score**											
	High quality	8	3277	3795	1.165	0.964-1.409	0.114	F	7.184	0.410	2.557	0.594
	Low quality	5	625	743	0.561	0.173-1.817	0.335	R	17.99	0.001	77.77	0.921
**Dominant model (TG+GG versus TT)**	**Overall**	13	3902	4538	1.047	0.943-1.163	0.386	F	12.81	0.383	6.307	0.343
	**Geographical region**											
	Asian	9	2606	2846	**1.158**	**1.006-1.332**	**0.041**	F	6.648	0.575	0.0	0.136

	European	1	124	160	0.660	0.384-1.134	0.132	F	0.0	1.0	0.0	NA
	American	3	1172	1532	0.953	0.808-1.123	0.563	F	0.145	0.930	0.0	0.730
	Cancer type											
	PTC	2	1254	1358	**1.262**	**1.040-1.531**	**0.018**	F	0.067	0.796	0.0	NA
	Colorectal cancer	2	571	542	1.128	0.817-1.558	0.464	F	0.534	0.465	0.0	NA
	Bladder cancer	1	718	726	0.942	0.757-1.171	0.589	F	0.0	1.0	0.0	NA
	Cervical precancerous lesions	1	296	296	1.452	0.868-2.430	0.155	F	0.0	1.0	0.0	NA
	Gastric cancer	1	137	142	0.979	0.490-1.957	0.953	F	0.0	1.0	0.0	NA
	HCC	1	147	209	0.647	0.357-1.172	0.151	F	0.0	1.0	0.0	NA
	Hodgkin lymphoma	1	101	200	0.974	0.596-1.594	0.918	F	0.0	1.0	0.0	NA
	Larynx cancer	1	124	160	0.660	0.384-1.134	0.132	F	0.0	1.0	0.0	NA
	Lung cancer	1	100	99	0.989	0.421-2.321	0.979	F	0.0	1.0	0.0	NA
	NHL	1	178	529	0.925	0.647-1.323	0.669	F	0.0	1.0	0.0	NA
	Renal cell carcinoma	1	276	277	1.011	0.707-1.446	0.951	F	0.0	1.0	0.0	NA
	Genotyping method											
	TaqMan PCR	3	1359	1588	0.992	0.686-1.435	0.967	R	5.016	0.081	60.12	0.249
	RFLP-PCR	3	675	739	1.096	0.840-1.430	0.499	F	3.892	0.143	48.61	0.548
	PCR-ligase detection	2	300	284	0.941	0.581-1.524	0.803	F	0.025	0.874	0.0	NA
	SNPlex technology	2	994	1003	0.960	0.797-1.157	0.668	F	0.112	0.738	0.0	NA
	Other methods	3	574	924	1.062	0.805-1.402	0.670	F	2.022	0.364	1.097	0.778
	Source of controls											
	Population-based	8	1364	1556	0.988	0.823-1.185	0.894	F	7.730	0.357	9.446	0.551
	Hospital-based	5	2538	2982	1.079	0.949-1.227	0.249	F	4.479	0.345	10.69	0.793
	HWE in controls											
	Equilibrium	10	3402	4002	1.074	0.959-1.202	0.218	F	9.774	0.369	7.916	0.605
	Disequilibrium	3	500	536	0.899	0.678-1.191	0.458	F	1.718	0.424	0.0	0.776
	Quality score											
	High quality	8	3277	3795	1.081	0.965-1.211	0.181	F	9.404	0.225	25.57	0.853
	Low quality	5	625	743	0.872	0.663-1.149	0.331	F	1.417	0.841	0.0	0.620
Homozygote model (GG *versus* TT)	Overall	13	2795	3209	1.015	0.830-1.242	0.882	R	24.05	0.020	50.11	0.789
	Geographical region											
	Asian	9	2133	2339	1.435	0.983-2.097	0.062	F	4.678	0.791	0.0	0.043
	European	1	62	116	**0.299**	**0.157-0.570**	**< 0.001**	F	0.0	1.0	0.0	NA
	American	3	600	754	1.040	0.823-1.313	0.743	F	2.305	0.316	13.21	0.309
	Cancer type											
	PTC	2	1000	1114	**1.784**	**1.078-2.953**	**0.024**	F	0.006	0.937	0.0	NA
	Colorectal cancer	2	478	463	0.635	0.103-3.917	0.625	F	0.080	0.777	0.0	NA
	Bladder cancer	1	362	363	0.908	0.665-1.239	0.543	F	0.0	1.0	0.0	NA
	Cervical precancerous lesions	1	259	269	2.086	0.188-23.14	0.549	F	0.0	1.0	0.0	NA
	Gastric cancer	1	120	124	1.034	0.064-16.71	0.981	F	0.0	1.0	0.0	NA
	HCC	1	128	171	0.442	0.018-10.95	0.618	F	0.0	1.0	0.0	NA
	Hodgkin lymphoma	1	60	108	1.279	0.653-2.505	0.473	F	0.0	1.0	0.0	NA
	Larynx cancer	1	62	116	**0.299**	**0.157-0.570**	**< 0.001**	F	0.0	1.0	0.0	NA
	Lung cancer	1	88	90	0.141	0.007-2.775	0.198	F	0.0	1.0	0.0	NA
	NHL	1	96	264	1.078	0.661-1.760	0.763	F	0.0	1.0	0.0	NA
	Renal cell carcinoma	1	142	127	1.437	0.864-2.391	0.163	F	0.0	1.0	0.0	NA
	Genotyping method											
	TaqMan PCR	3	1046	1258	0.863	0.291-2.560	0.791	R	14.82	0.001	86.50	0.423
	RFLP-PCR	3	538	590	1.573	0.835-2.964	0.161	F	1.631	0.442	0.0	0.094
	PCR-ligase detection	2	264	248	0.943	0.132-6.745	0.953	F	0.008	0.927	0.0	NA
	SNPlex technology	2	504	490	1.029	0.789-1.341	0.835	F	2.277	0.131	56.08	NA
	Other methods	3	443	623	1.051	0.654-1.688	0.838	F	2.067	0.356	3.241	0.731
	Source of controls											
	Population-based	8	991	1119	1.011	0.543-1.883	0.973	R	19.55	0.007	64.20	0.907
	Hospital-based	5	1804	2090	0.996	0.779-1.274	0.973	F	4.461	0.347	10.34	0.701
	HWE in controls											
	Equilibrium	10	2503	2876	1.101	0.887-1.367	0.384	F	6.217	0.718	0.0	0.616
	Disequilibrium	3	292	333	0.528	0.131-2.131	0.370	R	15.29	< 0.001	86.92	0.687
	Quality score											
	High quality	8	2321	2647	1.137	0.922-1.402	0.230	F	6.956	0.434	0.0	0.572
	Low quality	5	474	562	0.588	0.219-1.577	0.291	R	10.46	0.033	61.76	0.955
Heterozygote model (TG *versus* TT)	Overall	13	3600	4133	1.051	0.942-1.173	0.371	F	9.956	0.620	0.0	0.692
	Geographical region											
	Asian	9	2532	2772	1.134	0.980-1.311	0.090	F	6.105	0.635	0.0	0.213
	European	1	98	78	1.331	0.725-2.443	0.356	F	0.0	1.0	0.0	NA
	American	3	970	1283	0.925	0.777-1.101	0.381	F	0.167	0.920	0.0	0.181
	Cancer type											
	PTC	2	1206	1325	1.210	0.990-1.478	0.063	F	0.058	0.810	0.0	NA
	Colorectal cancer	2	569	539	1.147	0.827-1.590	0.411	F	0.572	0.450	0.0	NA
	Bladder cancer	1	604	604	0.953	0.757-1.199	0.682	F	0.0	1.0	0.0	NA

	Cervical precancerous lesions	1	294	295	1.429	0.846-2.415	0.182	F	0.0	1.0	0.0	NA
	Gastric cancer	1	136	141	0.976	0.480-1.984	0.947	F	0.0	1.0	0.0	NA
	HCC	1	147	208	0.664	0.366-1.206	0.179	F	0.0	1.0	0.0	NA
	Hodgkin lymphoma	1	80	168	0.868	0.509-1.480	0.604	F	0.0	1.0	0.0	NA
	Larynx cancer	1	98	78	1.331	0.725-2.443	0.356	F	0.0	1.0	0.0	NA
	Lung cancer	1	100	96	1.318	0.529-3.287	0.553	F	0.0	1.0	0.0	NA
	NHL	1	144	440	0.873	0.597-1.279	0.486	F	0.0	1.0	0.0	NA
	Renal cell carcinoma	1	222	239	0.903	0.621-1.315	0.596	F	0.0	1.0	0.0	NA
	**Genotyping method**											
	TaqMan PCR	3	1295	1463	1.179	0.976-1.425	0.087	F	1.516	0.469	0.0	0.710
	RFLP-PCR	3	643	714	1.057	0.803-1.392	0.691	F	3.017	0.221	33.71	0.456
	PCR-ligase detection	2	298	282	0.940	0.574-1.541	0.807	F	0.021	0.886	0.0	NA
	SNPlex technology	2	826	843	0.939	0.772-1.143	0.531	F	0.056	0.812	0.0	NA
	Other methods	3	538	831	1.061	0.792-1.421	0.690	F	2.455	0.293	18.53	0.532
	**Source of controls**											
	Population-based	8	1228	1378	0.995	0.821-1.206	0.956	F	5.256	0.629	0.0	0.835
	Hospital-based	5	2372	2755	1.080	0.945-1.234	0.259	F	4.225	0.376	5.315	0.953
	**HWE in controls**											
	Equilibrium	10	3180	3720	1.054	0.937-1.186	0.383	F	8.515	0.483	0.0	0.401
	Disequilibrium	3	420	413	1.036	0.766-1.400	0.819	F	1.430	0.489	0.0	0.324
	**Quality score**											
	High quality	8	3024	3509	1.055	0.937-1.187	0.378	F	8.430	0.296	16.96	0.605
	Low quality	5	576	624	1.032	0.768-1.385	0.836	F	1.508	0.825	0.0	0.475

OR: odds ratio, CI: confidence interval, F: fixed model, R: random model, NA: not appreciable

Supplementary Table S12. Meta-analysis of the association between RAN (C > T; rs14035) variant and cancer risk.

Comparison	Subgroups	No. of studies	Sample size		Test of association				Test of heterogeneity			Publication bias
			Cancer	Control	OR	95% CI	*P*-value	Model	Q test	*P*-value	I^2 (%)	*P*-value (Egger's)
**Allelic model (T allele *versus* C allele)**	**Overall**	12	7486	9800	0.940	0.854-1.034	0.203	R	29.96	0.002	63.29	0.273
	**Geographical region**											
	Asian	7	4686	5698	0.991	0.843-1.164	0.909	R	14.28	0.027	57.98	0.456
	European	2	418	1024	0.624	0.373-1.046	0.074	R	3.819	0.051	73.81	NA
	American	3	2382	3078	0.932	0.828-1.049	0.245	F	2.081	0.353	3.907	0.676
	**Cancer type**											
	Bladder cancer	1	1470	1464	0.898	0.769-1.050	0.178	F	0.0	1.0	0.0	NA
	Cervical precancerous lesions	1	590	592	0.885	0.666-1.176	0.400	F	0.0	1.0	0.0	NA
	CLL	1	198	684	0.803	0.572-1.128	0.206	F	0.0	1.0	0.0	NA
	Colorectal cancer	1	816	800	**0.779**	**0.612-0.991**	**0.042**	F	0.0	1.0	0.0	NA
	HCC	1	294	418	1.076	0.733-1.579	0.708	F	0.0	1.0	0.0	NA
	Larynx cancer	1	220	340	**0.474**	**0.316-0.711**	**< 0.001**	F	0.0	1.0	0.0	NA
	Lung cancer	1	186	180	0.687	0.413-1.143	0.148	F	0.0	1.0	0.0	NA
	Neuroblastoma	1	858	1768	0.964	0.779-1.191	0.732	F	0.0	1.0	0.0	NA
	NHL	1	360	1058	1.101	0.852-1.422	0.464	F	0.0	1.0	0.0	NA
	Oral cancer	1	878	876	1.266	0.996-1.609	0.054	F	0.0	1.0	0.0	NA
	Primary liver cancer	1	1064	1064	1.237	0.986-1.551	0.066	F	0.0	1.0	0.0	NA
	Renal cell carcinoma	1	552	556	0.872	0.674-1.129	0.299	F	0.0	1.0	0.0	NA
	**Genotyping method**											
	TaqMan PCR	4	2154	3668	0.851	0.601-1.205	0.363	R	17.77	< 0.001	83.11	0.199
	RFLP-PCR	3	2174	2282	1.008	0.739-1.376	0.958	R	7.667	0.022	73.91	0.991
	SNPlex technology	2	2022	2020	0.891	0.780-1.019	0.091	F	0.037	0.847	0.0	NA
	Other methods	3	1136	1830	0.953	0.798-1.140	0.600	F	3.056	0.217	34.55	0.335
	**Source of controls**											
	Population-based	6	2918	3654	0.874	0.689-1.110	0.270	R	18.35	0.003	72.76	0.130
	Hospital-based	6	4568	6146	0.957	0.824-1.110	0.561	R	11.44	0.043	56.28	0.875
	**HWE in controls**											
	Equilibrium	10	5796	7996	0.974	0.862-1.100	0.672	R	17.42	0.043	48.33	0.264
	Disequilibrium	2	1690	1804	0.671	0.360-1.253	0.211	R	8.339	0.004	88.01	NA
	**Quality score**											
	High quality	10	7080	9280	0.977	0.878-1.086	0.663	R	16.73	0.053	46.21	0.865
	Low quality	2	406	520	**0.547**	**0.399-0.751**	**< 0.001**	F	1.253	0.263	20.20	NA
**Recessive model (TT versus CC+CT)**	**Overall**	12	3743	4900	1.018	0.813-1.276	0.874	R	19.01	0.061	42.14	0.552
	**Geographical region**											
	Asian	7	2343	2849	1.217	0.706-2.098	0.479	R	16.46	0.011	63.55	0.633
	European	2	209	512	0.821	0.446-1.510	0.526	F	0.025	0.874	0.0	NA
	American	3	1191	1539	1.017	0.775-1.334	0.905	F	1.373	0.503	0.0	0.625
	**Cancer type**											
	Bladder cancer	1	735	732	0.910	0.627-1.320	0.619	F	0.0	1.0	0.0	NA
	Cervical precancerous lesions	1	295	296	0.657	0.290-1.488	0.314	F	0.0	1.0	0.0	NA
	CLL	1	99	342	0.848	0.408-1.764	0.660	F	0.0	1.0	0.0	NA
	Colorectal cancer	1	408	400	0.741	0.355-1.547	0.426	F	0.0	1.0	0.0	NA
	HCC	1	147	209	3.433	0.873-13.50	0.077	F	0.0	1.0	0.0	NA
	Larynx cancer	1	110	170	0.762	0.253-2.292	0.628	F	0.0	1.0	0.0	NA
	Lung cancer	1	93	90	0.966	0.270-3.456	0.957	F	0.0	1.0	0.0	NA
	Neuroblastoma	1	429	884	0.590	0.278-1.250	0.168	F	0.0	1.0	0.0	NA
	NHL	1	180	529	1.334	0.783-2.272	0.289	F	0.0	1.0	0.0	NA
	Oral cancer	1	439	438	1.891	0.950-3.763	0.070	F	0.0	1.0	0.0	NA
	Primary liver cancer	1	532	532	**3.094**	**1.377-6.952**	**0.006**	F	0.0	1.0	0.0	NA
	Renal cell carcinoma	1	276	278	0.962	0.529-1.749	0.899	F	0.0	1.0	0.0	NA
	**Genotyping method**											
	TaqMan PCR	4	1077	1834	0.981	0.664-1.450	0.925	F	5.607	0.132	46.50	0.610
	RFLP-PCR	3	1087	1141	1.858	0.636-5.427	0.257	R	7.944	0.019	74.82	0.621
	SNPlex technology	2	1011	1010	0.924	0.674-1.268	0.624	F	0.024	0.876	0.0	NA
	Other methods	3	568	915	1.067	0.700-1.626	0.763	F	2.049	0.359	2.397	0.584
	**Source of controls**											
	Population-based	6	1459	1827	1.191	0.702-2.019	0.517	R	11.53	0.042	56.63	0.498
	Hospital-based	6	2284	3073	1.010	0.793-1.286	0.937	F	7.191	0.207	30.47	0.977
	**HWE in controls**											
	Equilibrium	10	2898	3998	1.139	0.809-1.604	0.456	R	17.76	0.038	49.31	0.686
	Disequilibrium	2	845	902	0.893	0.628-1.271	0.531	F	0.089	0.765	0.0	NA
	**Quality score**											
	High quality	10	3540	4640	1.106	0.812-1.505	0.524	R	18.65	0.028	54.75	0.383
	Low quality	2	203	260	0.843	0.366-1.940	0.688	F	0.076	0.783	0.0	NA
**Dominant model**	**Overall**	12	3743	4900	0.908	0.812-1.015	0.089	R	30.97	0.001	64.48	0.085

**(CT+TT versus CC)**	**Geographical region**											
	Asian	7	2343	2849	0.964	0.818-1.136	0.660	R	10.90	0.091	44.96	0.225
	European	2	209	512	0.491	0.229-1.053	0.068	R	5.149	0.023	80.58	NA
	American	3	1191	1539	0.882	0.755-1.030	0.113	F	1.465	0.481	0.0	0.733
	**Cancer type**											
	Bladder cancer	1	735	732	0.854	0.695-1.049	0.133	F	0.0	1.0	0.0	NA
	Cervical precancerous lesions	1	295	296	0.907	0.648-1.269	0.570	F	0.0	1.0	0.0	NA
	CLL	1	99	342	0.719	0.459-1.127	0.150	F	0.0	1.0	0.0	NA
	Colorectal cancer	1	408	400	**0.737**	**0.554-0.980**	**0.036**	F	0.0	1.0	0.0	NA
	HCC	1	147	209	0.951	0.609-1.486	0.827	F	0.0	1.0	0.0	NA
	Larynx cancer	1	110	170	**0.330**	**0.200-0.544**	**< 0.001**	F	0.0	1.0	0.0	NA
	Lung cancer	1	93	90	0.589	0.321-1.084	0.089	F	0.0	1.0	0.0	NA
	Neuroblastoma	1	429	884	1.014	0.794-1.295	0.911	F	0.0	1.0	0.0	NA
	NHL	1	180	529	1.056	0.753-1.482	0.752	F	0.0	1.0	0.0	NA
	Oral cancer	1	439	438	1.235	0.933-1.636	0.140	F	0.0	1.0	0.0	NA
	Primary liver cancer	1	532	532	1.150	0.887-1.492	0.290	F	0.0	1.0	0.0	NA
	Renal cell carcinoma	1	276	278	0.805	0.577-1.124	0.203	F	0.0	1.0	0.0	NA
	**Genotyping method**											
	TaqMan PCR	4	1077	1834	0.769	0.481-1.229	0.272	R	22.11	< 0.001	86.43	0.146
	RFLP-PCR	3	1087	1141	0.933	0.694-1.254	0.645	R	5.139	0.077	61.08	0.899
	SNPlex technology	2	1011	1010	0.840	0.705-1.001	0.052	F	0.087	0.768	0.0	NA
	Other methods	3	568	915	0.914	0.732-1.142	0.430	F	2.691	0.260	25.68	0.257
	**Source of controls**											
	Population-based	6	1459	1827	0.784	0.579-1.062	0.117	R	19.95	0.001	74.93	0.062
	Hospital-based	6	2284	3073	0.927	0.780-1.102	0.390	R	10.33	0.066	51.58	0.666
	**HWE in controls**											
	Equilibrium	10	2898	3998	0.955	0.862-1.058	0.377	F	14.01	0.122	35.73	0.083
	Disequilibrium	2	845	902	0.546	0.215-1.386	0.203	R	11.87	0.001	91.57	NA
	**Quality score**											
	High quality	10	3540	4640	0.944	0.861-1.036	0.223	F	12.66	0.178	28.93	0.672
	Low quality	2	203	260	**0.417**	**0.283-0.614**	**< 0.001**	F	2.084	0.149	52.02	NA
**Homozygote model (TT *versus* CC)**	**Overall**	12	2453	3061	0.932	0.737-1.179	0.559	R	22.70	0.019	51.55	0.732
	**Geographical region**											
	Asian	7	1638	1965	1.182	0.672-2.081	0.561	R	17.46	0.008	65.63	0.738
	European	2	136	255	0.623	0.331-1.174	0.143	F	0.418	0.518	0.0	NA
	American	3	679	841	0.952	0.717-1.264	0.734	F	1.864	0.394	0.0	0.664
	**Cancer type**											
	Bladder cancer	1	406	381	0.841	0.571-1.240	0.382	F	0.0	1.0	0.0	NA
	Cervical precancerous lesions	1	202	201	0.646	0.283-1.474	0.299	F	0.0	1.0	0.0	NA
	CLL	1	58	178	0.719	0.334-1.547	0.399	F	0.0	1.0	0.0	NA
	Colorectal cancer	1	280	250	0.667	0.317-1.403	0.286	F	0.0	1.0	0.0	NA
	HCC	1	105	140	3.262	0.823-12.93	0.092	F	0.0	1.0	0.0	NA
	Larynx cancer	1	78	77	0.459	0.149-1.411	0.174	F	0.0	1.0	0.0	NA
	Lung cancer	1	70	57	0.800	0.220-2.912	0.735	F	0.0	1.0	0.0	NA
	Neuroblastoma	1	294	621	0.601	0.282-1.279	0.187	F	0.0	1.0	0.0	NA
	NHL	1	107	307	1.330	0.761-2.325	0.316	F	0.0	1.0	0.0	NA
	Oral cancer	1	305	314	1.978	0.988-3.959	0.054	F	0.0	1.0	0.0	NA
	Primary liver cancer	1	382	382	**3.134**	**1.390-7.067**	**0.006**	F	0.0	1.0	0.0	NA
	Renal cell carcinoma	1	166	153	0.865	0.465-1.606	0.645	F	0.0	1.0	0.0	NA
	**Genotyping method**											
	TaqMan PCR	4	735	1190	0.839	0.436-1.614	0.599	R	7.747	0.052	61.28	0.361
	RFLP-PCR	3	767	772	1.784	0.568-5.601	0.321	R	8.936	0.011	77.62	0.646
	SNPlex technology	2	572	534	0.848	0.610-1.178	0.325	F	0.005	0.942	0.0	NA
	Other methods	3	379	565	1.027	0.665-1.587	0.905	F	2.183	0.336	8.392	0.497
	**Source of controls**											
	Population-based	6	991	1131	1.061	0.582-1.934	0.847	R	14.25	0.014	64.92	0.671
	Hospital-based	6	1462	1930	0.965	0.752-1.238	0.778	F	8.391	0.136	40.41	0.986
	**HWE in controls**											
	Equilibrium	10	1969	2603	1.089	0.753-1.575	0.651	R	19.72	0.020	54.37	0.772
	Disequilibrium	2	484	458	0.789	0.547-1.138	0.204	F	0.999	0.318	0.0	NA
	**Quality score**											
	High quality	10	2305	2927	1.062	0.761-1.483	0.724	R	20.75	0.014	56.63	0.375
	Low quality	2	148	134	0.583	0.250-1.361	0.212	F	0.405	0.525	0.0	NA
**Heterozygote model (CT *versus* CC)**	**Overall**	12	3533	4621	**0.848**	**0.727-0.989**	**0.036**	R	26.82	0.005	58.99	0.046
	**Geographical region**											
	Asian	7	2251	2757	0.961	0.851-1.084	0.517	F	8.425	0.209	28.79	0.117
	European	2	194	462	0.480	0.214-1.074	0.074	R	5.235	0.022	80.90	NA
	American	3	1088	1402	0.869	0.738-1.022	0.090	F	0.810	0.667	0.0	0.827
	**Cancer type**											
	Bladder cancer	1	677	669	0.857	0.692-1.061	0.156	F	0.0	1.0	0.0	NA

	Cervical precancerous lesions	1	285	281	0.948	0.668-1.346	0.766	F	0.0	1.0	0.0	NA
	CLL	1	89	302	0.719	0.447-1.155	0.172	F	0.0	1.0	0.0	NA
	Colorectal cancer	1	395	383	**0.745**	**0.555-0.999**	**0.049**	F	0.0	1.0	0.0	NA
	HCC	1	140	206	0.851	0.536-1.352	0.494	F	0.0	1.0	0.0	NA
	Larynx cancer	1	105	160	**0.316**	**0.188-0.532**	**< 0.001**	F	0.0	1.0	0.0	NA
	Lung cancer	1	88	85	0.558	0.292-1.063	0.076	F	0.0	1.0	0.0	NA
	Neuroblastoma	1	420	853	1.063	0.827-1.366	0.635	F	0.0	1.0	0.0	NA
	NHL	1	158	479	0.994	0.693-1.426	0.975	F	0.0	1.0	0.0	NA
	Oral cancer	1	415	425	1.158	0.863-1.552	0.328	F	0.0	1.0	0.0	NA
	Primary liver cancer	1	508	524	1.045	0.799-1.367	0.750	F	0.0	1.0	0.0	NA
	Renal cell carcinoma	1	253	254	0.794	0.560-1.126	0.196	F	0.0	1.0	0.0	NA
	**Genotyping method**											
	TaqMan PCR	4	1029	1740	0.760	0.473-1.222	0.257	R	20.85	< 0.001	85.61	0.122
	RFLP-PCR	3	1043	1113	0.888	0.740-1.066	0.204	F	2.813	0.245	28.91	0.814
	SNPlex technology	2	930	923	0.839	0.699-1.007	0.059	F	0.132	0.716	0.0	NA
	Other methods	3	531	845	0.902	0.714-1.140	0.388	F	2.493	0.287	19.78	0.107
	**Source of controls**											
	Population-based	6	1380	1727	0.760	0.567-1.019	0.066	R	17.02	0.004	70.62	0.065
	Hospital-based	6	2153	2894	0.924	0.821-1.041	0.194	F	8.520	0.130	41.31	0.509
	**HWE in controls**											
	Equilibrium	10	2751	3792	0.933	0.839-1.038	0.201	F	10.75	0.293	16.28	0.032
	Disequilibrium	2	782	829	0.536	0.202-1.422	0.210	R	12.06	0.001	91.71	NA
	**Quality score**											
	High quality	10	3340	4376	0.928	0.842-1.021	0.126	F	8.906	0.446	0.0	0.464
	Low quality	2	193	245	**0.395**	**0.264-0.593**	**< 0.001**	F	1.805	0.179	44.60	NA

OR: odds ratio, CI: confidence interval, F: fixed model, R: random model, NA: not appreciable

Supplementary Table S13. Meta-analysis of the association between RAN (A > G; rs3803012) variant and cancer risk.

Comparison	Subgroups	No. of studies	Sample size		Test of association				Test of heterogeneity			Publication bias
			Cancer	Control	OR	95% CI	*P*-value	Model	Q test	*P*-value	I^2 (%)	*P*-value (Egger's)
**Allelic model (G allele *versus* A allele)**	**Overall**	5	8600	9930	1.103	0.969-1.254	0.138	F	0.888	0.926	0.0	0.958
	**Geographical region**											
	Asian	5	8600	9930	1.103	0.969-1.254	0.138	F	0.888	0.926	0.0	0.958
	**Cancer type**											
	HCC	2	3136	3320	1.081	0.866-1.350	0.490	F	0.0	0.996	0.0	NA
	Breast cancer	1	1740	1768	1.008	0.773-1.315	0.954	F	0.0	1.0	0.0	NA
	Cervical cancer	1	2942	3058	1.171	0.923-1.486	0.192	F	0.0	1.0	0.0	NA
	Head/Neck cancer	1	782	1784	1.188	0.833-1.696	0.341	F	0.0	1.0	0.0	NA
	**Genotyping method**											
	TaqMan PCR	5	8600	9930	1.103	0.969-1.254	0.138	F	0.888	0.926	0.0	0.958
	**Source of controls**											
	Population-based	4	7976	9290	1.106	0.963-1.270	0.155	F	0.876	0.831	0.0	0.863
	Hospital-based	1	624	640	1.082	0.755-1.550	0.668	F	0.0	1.0	0.0	NA
	**HWE in controls**											
	Equilibrium	5	8600	9930	1.103	0.969-1.254	0.138	F	0.888	0.926	0.0	0.958
	**Quality score**											
	High quality	5	8600	9930	1.103	0.969-1.254	0.138	F	0.888	0.926	0.0	0.958
**Recessive model (GG versus AA+AG)**	**Overall**	5	4300	4965	**1.930**	**1.032-3.611**	**0.040**	F	0.982	0.913	0.0	0.613
	**Geographical region**											
	Asian	5	4300	4965	**1.930**	**1.032-3.611**	**0.040**	F	0.982	0.913	0.0	0.613
	**Cancer type**											
	HCC	2	1568	1660	1.521	0.528-4.384	0.437	F	0.533	0.466	0.0	NA
	Breast cancer	1	870	884	2.459	0.863-7.009	0.092	F	0.0	1.0	0.0	NA
	Cervical cancer	1	1471	1529	1.735	0.414-7.272	0.451	F	0.0	1.0	0.0	NA
	Head/Neck cancer	1	391	892	2.288	0.321-16.30	0.409	F	0.0	1.0	0.0	NA
	**Genotyping method**											
	TaqMan PCR	5	4300	4965	**1.930**	**1.032-3.611**	**0.040**	F	0.982	0.913	0.0	0.613
	**Source of controls**											
	Population-based	4	3988	4645	**2.284**	**1.095-4.761**	**0.028**	F	0.247	0.970	0.0	0.802
	Hospital-based	1	312	320	1.235	0.373-4.090	0.729	F	0.0	1.0	0.0	NA
	**HWE in controls**											
	Equilibrium	5	4300	4965	**1.930**	**1.032-3.611**	**0.040**	F	0.982	0.913	0.0	0.613
	**Quality score**											
	High quality	5	4300	4965	**1.930**	**1.032-3.611**	**0.040**	F	0.982	0.913	0.0	0.613
**Dominant model (AG+GG versus AA)**	**Overall**	5	4300	4965	1.077	0.940-1.234	0.285	F	1.547	0.818	0.0	0.987
	**Geographical region**											
	Asian	5	4300	4965	1.077	0.940-1.234	0.285	F	1.547	0.818	0.0	0.987
	**Cancer type**											
	HCC	2	1568	1660	1.066	0.843-1.347	0.594	F	0.003	0.959	0.0	NA
	Breast cancer	1	870	884	0.936	0.704-1.244	0.648	F	0.0	1.0	0.0	NA
	Cervical cancer	1	1471	1529	1.166	0.911-1.493	0.223	F	0.0	1.0	0.0	NA
	Head/Neck cancer	1	391	892	1.174	0.808-1.704	0.400	F	0.0	1.0	0.0	NA
	**Genotyping method**											
	TaqMan PCR	5	4300	4965	1.077	0.940-1.234	0.285	F	1.547	0.818	0.0	0.987
	**Source of controls**											
	Population-based	4	3988	4645	1.077	0.932-1.245	0.313	F	1.547	0.672	0.0	0.977
	Hospital-based	1	312	320	1.075	0.724-1.595	0.721	F	0.0	1.0	0.0	NA
	**HWE in controls**											
	Equilibrium	5	4300	4965	1.077	0.940-1.234	0.285	F	1.547	0.818	0.0	0.987
	**Quality score**											
	High quality	5	4300	4965	1.077	0.940-1.234	0.285	F	1.547	0.818	0.0	0.987
**Homozygote model (GG *versus* AA)**	**Overall**	5	3871	4485	**1.933**	**1.033-3.618**	**0.039**	F	0.931	0.920	0.0	0.578
	**Geographical region**											
	Asian	5	3871	4485	**1.933**	**1.033-3.618**	**0.039**	F	0.931	0.920	0.0	0.578
	**Cancer type**											
	HCC	2	1417	1507	1.535	0.532-4.432	0.428	F	0.524	0.469	0.0	NA
	Breast cancer	1	778	777	2.419	0.848-6.899	0.099	F	0.0	1.0	0.0	NA
	Cervical cancer	1	1330	1400	1.757	0.419-7.367	0.441	F	0.0	1.0	0.0	NA
	Head/Neck cancer	1	346	801	2.323	0.326-16.56	0.400	F	0.0	1.0	0.0	NA
	**Genotyping method**											
	TaqMan PCR	5	3871	4485	**1.933**	**1.033-3.618**	**0.039**	F	0.931	0.920	0.0	0.578
	**Source of controls**											
	Population-based	4	3615	4220	**2.279**	**1.093-4.752**	**0.028**	F	0.228	0.973	0.0	0.736
	Hospital-based	1	256	265	1.248	0.376-4.141	0.717	F	0.0	1.0	0.0	NA
	**HWE in controls**											

	Equilibrium	5	3871	4485	**1.933**	**1.033-3.618**	**0.039**	F	0.931	0.920	0.0	0.578
	**Quality score**											
	High quality	5	3871	4485	**1.933**	**1.033-3.618**	**0.039**	F	0.931	0.920	0.0	0.578
**Heterozygote model (AG *versus* AA)**	**Overall**	5	4272	4949	1.047	0.911-1.203	0.518	F	2.368	0.669	0.0	0.965
	**Geographical region**											
	Asian	5	4272	4949	1.047	0.911-1.203	0.518	F	2.368	0.669	0.0	0.965
	**Cancer type**											
	HCC	2	1559	1654	1.046	0.824-1.327	0.714	F	0.006	0.941	0.0	NA
	Breast cancer	1	858	879	0.867	0.644-1.165	0.343	F	0.0	1.0	0.0	NA
	Cervical cancer	1	1466	1526	1.152	0.897-1.480	0.267	F	0.0	1.0	0.0	NA
	Head/Neck cancer	1	389	890	1.149	0.786-1.678	0.474	F	0.0	1.0	0.0	NA
	**Genotyping method**											
	TaqMan PCR	5	4272	4949	1.047	0.911-1.203	0.518	F	2.368	0.669	0.0	0.965
	**Source of controls**											
	Population-based	4	3966	4634	1.045	0.902-1.211	0.556	F	2.364	0.500	0.0	0.925
	Hospital-based	1	306	315	1.059	0.702-1.597	0.785	F	0.0	1.0	0.0	NA
	**HWE in controls**											
	Equilibrium	5	4272	4949	1.047	0.911-1.203	0.518	F	2.368	0.669	0.0	0.965
	**Quality score**											
	High quality	5	4272	4949	1.047	0.911-1.203	0.518	F	2.368	0.669	0.0	0.965

OR: odds ratio, CI: confidence interval, F: fixed model, R: random model, NA: not appreciable

Supplementary Table S14. Meta-analysis of the association between DICER (A > T; rs13078) variant and cancer risk.

Comparison	Subgroups	No. of studies	Sample size		Test of association				Test of heterogeneity			Publication bias
			Cancer	Control	OR	95% CI	*P*-value	Model	Q test	*P*-value	I^2 (%)	*P*-value (Egger's)
**Allelic model (T allele *versus* A allele)**	**Overall**	9	6654	7496	0.970	0.826-1.138	0.709	R	23.48	0.003	65.93	0.795
	**Geographical region**											
	Asian	5	4166	4434	1.007	0.826-1.228	0.943	F	0.873	0.928	0.0	0.077
	European	2	466	1030	0.571	0.304-1.071	0.081	R	5.367	0.021	81.37	NA
	American	2	2022	2032	1.003	0.742-1.356	0.982	R	2.946	0.086	66.05	NA
	**Cancer type**											
	Bladder cancer	2	2826	2922	0.892	0.760-1.047	0.161	F	0.081	0.776	0.0	NA
	Breast cancer	1	1728	1784	1.009	0.740-1.375	0.957	F	0.0	1.0	0.0	NA
	Cervical precancerous lesions	1	590	592	1.077	0.515-2.252	0.844	F	0.0	1.0	0.0	NA
	CLL	1	210	690	0.793	0.527-1.192	0.264	F	0.0	1.0	0.0	NA
	HCC	1	294	418	1.268	0.623-2.582	0.512	F	0.0	1.0	0.0	NA
	Larynx cancer	1	256	340	**0.417**	**0.291-0.597**	**< 0.001**	F	0.0	1.0	0.0	NA
	Lung cancer	1	196	194	1.222	0.495-3.019	0.664	F	0.0	1.0	0.0	NA
	Renal cell carcinoma	1	554	556	1.201	0.887-1.625	0.237	F	0.0	1.0	0.0	NA
	**Genotyping method**											
	TaqMan PCR	3	2194	2814	0.695	0.406-1.192	0.186	R	13.60	0.001	85.29	0.729
	SNPlex technology	2	2022	2032	1.003	0.742-1.356	0.982	R	2.946	0.086	66.05	NA
	Other methods	4	2438	2650	1.006	0.778-1.302	0.962	F	0.873	0.832	0.0	0.075
	**Source of controls**											
	Population-based	7	5100	5856	0.873	0.668-1.142	0.322	R	22.86	0.001	73.75	0.957
	Hospital-based	2	1554	1640	0.956	0.710-1.287	0.766	F	0.319	0.572	0.0	NA
	**HWE in controls**											
	Equilibrium	8	6398	7156	0.960	0.853-1.081	0.498	F	4.892	0.673	0.0	0.239
	Disequilibrium	1	256	340	**0.417**	**0.291-0.597**	**< 0.001**	F	0.0	1.0	0.0	NA
	**Quality score**											
	High quality	7	6202	6962	0.956	0.848-1.077	0.458	F	4.613	0.594	0.0	0.351
	Low quality	2	452	534	0.657	0.232-1.861	0.429	R	4.695	0.030	78.70	NA
**Recessive model (TT versus AA+AT)**	**Overall**	6	2787	3146	1.094	0.763-1.570	0.626	F	4.912	0.427	0.0	0.925
	**Geographical region**											
	Asian	2	1543	1615	0.861	0.227-3.267	0.826	F	2.658	0.103	62.37	NA
	European	2	233	515	1.395	0.717-2.717	0.327	F	1.188	0.276	15.85	NA
	American	2	1011	1016	1.005	0.638-1.581	0.983	F	0.294	0.587	0.0	NA
	**Cancer type**											
	Bladder cancer	2	1413	1461	0.865	0.523-1.430	0.571	F	1.741	0.187	42.55	NA
	Breast cancer	1	864	892	2.070	0.378-11.33	0.402	F	0.0	1.0	0.0	NA
	CLL	1	105	345	1.950	0.795-4.785	0.145	F	0.0	1.0	0.0	NA
	Larynx cancer	1	128	170	0.926	0.342-2.502	0.879	F	0.0	1.0	0.0	NA
	Renal cell carcinoma	1	277	278	1.264	0.491-3.252	0.627	F	0.0	1.0	0.0	NA
	**Genotyping method**											
	TaqMan PCR	3	1097	1407	1.471	0.791-2.735	0.223	F	1.367	0.505	0.0	0.874
	SNPlex technology	2	1011	1016	1.005	0.638-1.581	0.983	F	0.294	0.587	0.0	NA
	Other methods	1	679	723	0.212	0.025-1.818	0.157	F	0.0	1.0	0.0	NA
	**Source of controls**											
	Population-based	5	2108	2423	1.147	0.796-1.655	0.462	F	2.606	0.626	0.0	0.227
	Hospital-based	1	679	723	0.212	0.025-1.818	0.157	F	0.0	1.0	0.0	NA
	**HWE in controls**											
	Equilibrium	5	2659	2976	1.122	0.762-1.653	0.560	F	4.787	0.310	16.44	0.977
	Disequilibrium	1	128	170	0.926	0.342-2.502	0.879	F	0.0	1.0	0.0	NA
	**Quality score**											
	High quality	5	2659	2976	1.122	0.762-1.653	0.560	F	4.787	0.310	16.44	0.977
	Low quality	1	128	170	0.926	0.342-2.502	0.879	F	0.0	1.0	0.0	NA
**Dominant model (AT+TT versus AA)**	**Overall**	9	3327	3748	0.987	0.826-1.178	0.882	R	43.13	< 0.001	81.45	0.779
	**Geographical region**											
	Asian	5	2083	2217	1.017	0.828-1.250	0.870	F	0.706	0.951	0.0	0.033
	European	2	233	515	0.341	0.109-1.068	0.065	R	10.47	0.001	90.45	NA
	American	2	1011	1016	0.999	0.692-1.442	0.995	R	3.215	0.073	68.90	NA
	**Cancer type**											
	Bladder cancer	2	1413	1461	0.884	0.737-1.060	0.184	F	0.451	0.502	0.0	NA
	Breast cancer	1	864	892	0.981	0.710-1.355	0.907	F	0.0	1.0	0.0	NA
	Cervical precancerous lesions	1	295	296	1.079	0.511-2.277	0.842	F	0.0	1.0	0.0	NA
	CLL	1	105	345	**0.609**	**0.373-0.994**	**0.047**	F	0.0	1.0	0.0	NA
	HCC	1	147	209	1.283	0.619-2.660	0.502	F	0.0	1.0	0.0	NA

	Larynx cancer	1	128	170	**0.190**	**0.114-0.316**	**< 0.001**	F	0.0	1.0	0.0	NA
	Lung cancer	1	98	97	1.236	0.488-3.132	0.655	F	0.0	1.0	0.0	NA
	Renal cell carcinoma	1	277	278	1.239	0.872-1.761	0.232	F	0.0	1.0	0.0	NA
	**Genotyping method**											
	TaqMan PCR	3	1097	1407	0.491	0.190-1.270	0.142	R	28.49	< 0.001	92.98	0.367
	SNPlex technology	2	1011	1016	0.999	0.692-1.442	0.995	R	3.215	0.073	68.90	NA
	Other methods	4	1219	1325	1.043	0.798-1.363	0.757	F	0.623	0.891	0.0	0.102
	**Source of controls**											
	Population-based	7	2550	2928	0.768	0.509-1.159	0.208	R	41.69	< 0.001	85.61	0.668
	Hospital-based	2	777	820	0.998	0.731-1.363	0.992	F	0.229	0.633	0.0	NA
	**HWE in controls**											
	Equilibrium	8	3199	3578	0.942	0.825-1.076	0.378	F	7.501	0.379	6.676	0.442
	Disequilibrium	1	128	170	**0.190**	**0.114-0.316**	**< 0.001**	F	0.0	1.0	0.0	NA
	**Quality score**											
	High quality	7	3101	3481	0.937	0.819-1.071	0.339	F	7.166	0.306	16.27	0.592
	Low quality	2	226	267	0.465	0.074-2.908	0.413	R	12.01	0.001	91.67	NA
**Homozygote model (TT *versus* AA)**	**Overall**	6	2281	2446	0.923	0.640-1.331	0.669	F	8.331	0.139	39.99	0.770
	**Geographical region**											
	Asian	2	1393	1456	0.860	0.227-3.262	0.824	F	2.642	0.104	62.15	NA
	European	2	168	280	0.753	0.162-3.496	0.717	R	4.939	0.026	79.75	NA
	American	2	720	710	0.981	0.621-1.549	0.933	F	0.562	0.454	0.0	NA
	**Cancer type**											
	Bladder cancer	2	1138	1157	0.823	0.496-1.366	0.451	F	1.615	0.204	38.09	NA
	Breast cancer	1	789	811	2.061	0.376-11.28	0.404	F	0.0	1.0	0.0	NA
	CLL	1	86	234	1.612	0.651-3.989	0.302	F	0.0	1.0	0.0	NA
	Larynx cancer	1	82	46	**0.336**	**0.118-0.955**	**0.041**	F	0.0	1.0	0.0	NA
	Renal cell carcinoma	1	186	198	1.349	0.521-3.496	0.537	F	0.0	1.0	0.0	NA
	**Genotyping method**											
	TaqMan PCR	3	957	1091	0.968	0.303-3.088	0.956	R	5.906	0.052	66.13	0.890
	SNPlex technology	2	720	710	0.981	0.621-1.549	0.933	F	0.562	0.454	0.0	NA
	Other methods	1	604	645	0.212	0.025-1.822	0.158	F	0.0	1.0	0.0	NA
	**Source of controls**											
	Population-based	5	1677	1801	0.965	0.665-1.398	0.849	F	6.482	0.166	38.29	0.754
	Hospital-based	1	604	645	0.212	0.025-1.822	0.158	F	0.0	1.0	0.0	NA
	**HWE in controls**											
	Equilibrium	5	2199	2400	1.063	0.720-1.571	0.758	F	4.231	0.376	5.460	0.987
	Disequilibrium	1	82	46	**0.336**	**0.118-0.955**	**0.041**	F	0.0	1.0	0.0	NA
	**Quality score**											
	High quality	5	2199	2400	1.063	0.720-1.571	0.758	F	4.231	0.376	5.460	0.987
	Low quality	1	82	46	**0.336**	**0.118-0.955**	**0.041**	F	0.0	1.0	0.0	NA
**Heterozygote model (AT *versus* AA)**	**Overall**	9	3268	3678	0.976	0.817-1.166	0.788	R	46.27	< 0.001	82.71	0.703
	**Geographical region**											
	Asian	5	2078	2210	1.026	0.833-1.265	0.806	F	0.722	0.949	0.0	0.063
	European	2	218	491	**0.292**	**0.110-0.776**	**0.014**	R	6.636	0.010	84.93	NA
	American	2	972	977	0.989	0.687-1.425	0.955	R	2.955	0.086	66.16	NA
	**Cancer type**											
	Bladder cancer	2	1383	1425	0.895	0.741-1.081	0.249	F	0.853	0.356	0.0	NA
	Breast cancer	1	860	890	0.954	0.687-1.326	0.780	F	0.0	1.0	0.0	NA
	Cervical precancerous lesions	1	295	296	1.079	0.511-2.277	0.842	F	0.0	1.0	0.0	NA
	CLL	1	97	331	**0.483**	**0.278-0.838**	**0.010**	F	0.0	1.0	0.0	NA
	HCC	1	147	209	1.283	0.619-2.660	0.502	F	0.0	1.0	0.0	NA
	Larynx cancer	1	121	160	**0.178**	**0.106-0.300**	**< 0.001**	F	0.0	1.0	0.0	NA
	Lung cancer	1	98	97	1.236	0.488-3.132	0.655	F	0.0	1.0	0.0	NA
	Renal cell carcinoma	1	267	270	1.228	0.854-1.767	0.268	F	0.0	1.0	0.0	NA
	**Genotyping method**											
	TaqMan PCR	3	1078	1381	0.441	0.160-1.212	0.112	R	28.86	< 0.001	93.07	0.377
	SNPlex technology	2	972	977	0.989	0.687-1.425	0.955	R	2.955	0.086	66.16	NA
	Other methods	4	1218	1320	1.078	0.823-1.413	0.585	F	0.406	0.939	0.0	0.153
	**Source of controls**											
	Population-based	7	2492	2863	0.734	0.474-1.136	0.165	R	43.93	< 0.001	86.34	0.604
	Hospital-based	2	776	815	1.043	0.761-1.431	0.792	F	0.145	0.704	0.0	NA
	**HWE in controls**											
	Equilibrium	8	3147	3518	0.935	0.815-1.073	0.340	F	9.974	0.190	29.82	0.673
	Disequilibrium	1	121	160	**0.178**	**0.106-0.300**	**< 0.001**	F	0.0	1.0	0.0	NA
	**Quality score**											
	High quality	7	3049	3421	0.930	0.809-1.068	0.302	F	9.621	0.142	37.63	0.842
	Low quality	2	219	257	0.451	0.068-3.007	0.411	R	12.69	< 0.001	92.12	NA

OR: odds ratio, CI: confidence interval, F: fixed model, R: random model, NA: not appreciable

Supplementary Table S15. Meta-analysis of the association between DICER (T > C; rs1057035) variant and cancer risk.

Comparison	Subgroups	No. of studies	Sample size		Test of association				Test of heterogeneity			Publication bias
			Cancer	Control	OR	95% CI	*P*-value	Model	Q test	*P*-value	I^2 (%)	*P*-value (Egger's)
**Allelic model (C allele *versus* T allele)**	**Overall**	11	13998	17974	**0.887**	**0.825-0.953**	**0.001**	R	17.95	0.056	44.29	0.618
	**Geographical region**											
	Asian	9	13394	16876	**0.886**	**0.827-0.949**	**0.001**	R	8.730	0.366	8.366	0.983
	European	2	604	1098	0.994	0.527-1.876	0.985	R	8.610	0.003	88.39	NA
	**Cancer type**											
	Head/Neck carcinoma	2	1938	4878	**0.816**	**0.685-0.972**	**0.023**	F	0.152	0.697	0.0	NA
	HCC	2	3174	3318	**0.802**	**0.687-0.935**	**0.005**	F	2.350	0.125	57.44	NA
	Bladder cancer	1	1370	1460	1.022	0.808-1.292	0.857	F	0.0	1.0	0.0	NA
	Breast cancer	1	1696	1762	0.915	0.731-1.145	0.438	F	0.0	1.0	0.0	NA
	Cervical carcinoma	1	2950	3056	0.968	0.825-1.135	0.687	F	0.0	1.0	0.0	NA
	CLL	1	208	692	**1.379**	**1.002-1.897**	**0.049**	F	0.0	1.0	0.0	NA
	Colorectal cancer	1	396	406	**0.721**	**0.538-0.966**	**0.028**	F	0.0	1.0	0.0	NA
	ESCC	1	1066	1202	0.947	0.793-1.131	0.550	F	0.0	1.0	0.0	NA
	Lung cancer	1	1200	1200	0.826	0.681-1.001	0.051	F	0.0	1.0	0.0	NA
	**Genotyping method**											
	TaqMan PCR	9	11782	13676	0.897	0.802-1.004	0.060	R	17.24	0.028	53.60	0.534
	Other methods	2	2216	4298	0.905	0.787-1.041	0.164	F	0.663	0.416	0.0	NA
	**Source of controls**											
	Population-based	8	10804	14674	**0.887**	**0.789-0.997**	**0.044**	R	15.66	0.028	55.31	0.888
	Hospital-based	3	3194	3300	0.914	0.801-1.044	0.186	F	2.128	0.345	6.005	0.470
	**HWE in controls**											
	Equilibrium	10	12932	16772	**0.891**	**0.805-0.985**	**0.025**	R	17.46	0.042	48.46	0.523
	Disequilibrium	1	1066	1202	0.947	0.793-1.131	0.550	F	0.0	1.0	0.0	NA
	**Quality score**											
	High quality	9	12402	16368	0.922	0.834-1.020	0.114	R	14.82	0.063	46.02	0.379
	Low quality	2	1596	1606	**0.793**	**0.675-0.931**	**0.005**	F	0.577	0.447	0.0	NA
**Recessive model (CC versus TT+TC)**	**Overall**	11	6999	8987	0.902	0.700-1.163	0.428	R	22.35	0.013	55.25	0.619
	**Geographical region**											
	Asian	9	6697	8438	0.893	0.690-1.156	0.389	F	6.579	0.583	0.0	0.908
	European	2	302	549	1.279	0.293-5.583	0.744	R	13.03	< 0.001	92.33	NA
	**Cancer type**											
	Head/Neck carcinoma	2	969	2439	0.689	0.298-1.595	0.385	F	0.239	0.625	0.0	NA
	HCC	2	1587	1659	0.971	0.581-1.623	0.911	F	0.006	0.941	0.0	NA
	Bladder cancer	1	685	730	2.297	0.985-5.357	0.054	F	0.0	1.0	0.0	NA
	Breast cancer	1	848	881	0.806	0.299-2.175	0.671	F	0.0	1.0	0.0	NA
	Cervical carcinoma	1	1475	1528	0.854	0.463-1.574	0.613	F	0.0	1.0	0.0	NA
	CLL	1	104	346	**2.702**	**1.547-4.717**	**< 0.001**	F	0.0	1.0	0.0	NA
	Colorectal cancer	1	198	203	0.600	0.331-1.091	0.094	F	0.0	1.0	0.0	NA
	ESCC	1	533	601	0.561	0.140-2.256	0.416	F	0.0	1.0	0.0	NA
	Lung cancer	1	600	600	0.737	0.453-1.200	0.220	F	0.0	1.0	0.0	NA
	**Genotyping method**											
	TaqMan PCR	9	5891	6838	1.029	0.698-1.517	0.886	R	21.40	0.006	62.62	0.880
	Other methods	2	1108	2149	0.703	0.312-1.585	0.395	F	0.153	0.696	0.0	NA
	**Source of controls**											
	Population-based	8	5402	7337	0.927	0.590-1.456	0.742	R	17.10	0.017	59.06	0.264
	Hospital-based	3	1597	1650	1.095	0.575-2.086	0.782	R	5.204	0.074	61.57	0.362
	**HWE in controls**											
	Equilibrium	10	6466	8386	1.009	0.704-1.447	0.960	R	21.66	0.010	58.45	0.792
	Disequilibrium	1	533	601	0.561	0.140-2.256	0.416	F	0.0	1.0	0.0	NA
	**Quality score**											
	High quality	9	6201	8184	1.117	0.750-1.662	0.587	R	15.91	0.044	49.73	0.112
	Low quality	2	798	803	**0.679**	**0.465-0.990**	**0.044**	F	0.271	0.603	0.0	NA
**Dominant model (TC+CC versus TT)**	**Overall**	11	6999	8987	**0.861**	**0.798-0.929**	**< 0.001**	F	10.13	0.429	1.284	0.983
	**Geographical region**											
	Asian	9	6697	8438	**0.864**	**0.799-0.934**	**< 0.001**	F	8.271	0.407	3.275	0.893
	European	2	302	549	0.828	0.616-1.111	0.209	F	1.785	0.182	43.98	NA
	**Cancer type**											
	Head/Neck carcinoma	2	969	2439	**0.805**	**0.667-0.971**	**0.024**	F	0.122	0.727	0.0	NA
	HCC	2	1587	1659	**0.765**	**0.645-0.907**	**0.002**	F	2.603	0.107	61.58	NA
	Bladder cancer	1	685	730	0.943	0.728-1.221	0.655	F	0.0	1.0	0.0	NA
	Breast cancer	1	848	881	0.913	0.717-1.164	0.464	F	0.0	1.0	0.0	NA
	Cervical carcinoma	1	1475	1528	0.974	0.818-1.160	0.767	F	0.0	1.0	0.0	NA
	CLL	1	104	346	1.035	0.666-1.609	0.878	F	0.0	1.0	0.0	NA

	Colorectal cancer	1	198	203	0.691	0.465-1.027	0.068	F	0.0	1.0	0.0	NA
	ESCC	1	533	601	0.922	0.725-1.173	0.511	F	0.0	1.0	0.0	NA
	Lung cancer	1	600	600	0.811	0.643-1.022	0.076	F	0.0	1.0	0.0	NA
	Genotyping method											
	TaqMan PCR	9	5891	6838	**0.858**	**0.789-0.933**	**< 0.001**	F	9.707	0.286	17.59	0.977
	Other methods	2	1108	2149	0.875	0.737-1.039	0.127	F	0.383	0.536	0.0	NA
	Source of controls											
	Population-based	8	5402	7337	**0.853**	**0.782-0.930**	**< 0.001**	F	8.831	0.265	20.74	0.779
	Hospital-based	3	1597	1650	0.888	0.761-1.036	0.132	F	1.093	0.579	0.0	0.423
	HWE in controls											
	Equilibrium	10	6466	8386	**0.855**	**0.789-0.925**	**< 0.001**	F	9.780	0.369	7.978	0.979
	Disequilibrium	1	533	601	0.922	0.725-1.173	0.511	F	0.0	1.0	0.0	NA
	Quality score											
	High quality	9	6201	8184	**0.867**	**0.807-0.950**	**0.001**	F	8.521	0.384	6.114	0.589
	Low quality	2	798	803	**0.778**	**0.637-0.951**	**0.014**	F	0.463	0.496	0.0	NA
**Homozygote model (CC *versus* TT)**	Overall	11	5366	6678	0.868	0.672-1.121	0.278	R	19.38	0.036	48.39	0.741
	Geographical region											
	Asian	9	5178	6372	0.861	0.664-1.117	0.260	F	6.819	0.556	0.0	0.927
	European	2	188	306	1.103	0.255-4.771	0.895	R	11.33	0.001	91.17	NA
	Cancer type											
	Head/Neck carcinoma	2	796	1929	0.663	0.286-1.535	0.337	F	0.256	0.613	0.0	NA
	HCC	2	1312	1299	0.934	0.558-1.563	0.794	F	0.002	0.966	0.0	NA
	Bladder cancer	1	565	585	2.237	0.958-5.227	0.063	F	0.0	1.0	0.0	NA
	Breast cancer	1	703	720	0.795	0.294-2.145	0.650	F	0.0	1.0	0.0	NA
	Cervical carcinoma	1	1182	1221	0.851	0.461-1.571	0.606	F	0.0	1.0	0.0	NA
	CLL	1	72	194	**2.320**	**1.277-4.217**	**0.006**	F	0.0	1.0	0.0	NA
	Colorectal cancer	1	116	112	**0.521**	**0.277-0.980**	**0.043**	F	0.0	1.0	0.0	NA
	ESCC	1	210	228	0.536	0.132-2.172	0.383	F	0.0	1.0	0.0	NA
	Lung cancer	1	410	390	0.691	0.421-1.133	0.143	F	0.0	1.0	0.0	NA
	Genotyping method											
	TaqMan PCR	9	4679	5208	0.964	0.666-1.395	0.846	R	18.59	0.017	56.96	0.981
	Other methods	2	687	1470	0.677	0.300-1.530	0.348	F	0.162	0.688	0.0	NA
	Source of controls											
	Population-based	8	4158	5465	0.870	0.575-1.317	0.512	R	13.87	0.053	49.55	0.310
	Hospital-based	3	1208	1213	1.063	0.545-2.072	0.858	R	5.500	0.064	63.63	0.336
	HWE in controls											
	Equilibrium	10	5156	6450	0.948	0.674-1.333	0.758	R	18.77	0.027	52.05	0.939
	Disequilibrium	1	210	228	0.536	0.132-2.172	0.383	F	0.0	1.0	0.0	NA
	Quality score											
	High quality	9	4840	6176	1.130	0.861-1.482	0.379	F	12.79	0.119	37.47	0.148
	Low quality	2	526	502	**0.621**	**0.420-0.916**	**0.016**	F	0.475	0.491	0.0	NA
**Heterozygote model (TC *versus* TT)**	Overall	11	6841	8774	**0.854**	**0.790-0.923**	**< 0.001**	F	9.148	0.518	0.0	0.510
	Geographical region											
	Asian	9	6585	8295	**0.862**	**0.795-0.934**	**< 0.001**	F	8.251	0.409	3.040	0.943
	European	2	256	479	0.735	0.533-1.016	0.062	F	0.023	0.880	0.0	NA
	Cancer type											
	Head/Neck carcinoma	2	962	2413	**0.812**	**0.671-0.984**	**0.033**	F	0.078	0.779	0.0	NA
	HCC	2	1558	1628	0.797	0.570-1.114	0.185	R	2.728	0.099	63.35	NA
	Bladder cancer	1	668	722	0.871	0.666-1.140	0.315	F	0.0	1.0	0.0	NA
	Breast cancer	1	841	872	0.920	0.718-1.178	0.509	F	0.0	1.0	0.0	NA
	Cervical carcinoma	1	1456	1505	0.983	0.822-1.176	0.852	F	0.0	1.0	0.0	NA
	CLL	1	78	308	0.714	0.432-1.181	0.190	F	0.0	1.0	0.0	NA
	Colorectal cancer	1	178	171	0.751	0.493-1.144	0.182	F	0.0	1.0	0.0	NA
	ESCC	1	530	595	0.929	0.730-1.182	0.547	F	0.0	1.0	0.0	NA
	Lung cancer	1	570	560	0.833	0.653-1.064	0.143	F	0.0	1.0	0.0	NA
	Genotyping method											
	TaqMan PCR	9	5741	6648	**0.848**	**0.777-0.925**	**< 0.001**	F	8.610	0.376	7.080	0.571
	Other methods	2	1100	2126	0.881	0.740-1.048	0.152	F	0.390	0.532	0.0	NA
	Source of controls											
	Population-based	8	5304	7186	**0.848**	**0.776-0.927**	**< 0.001**	F	8.496	0.291	17.60	0.379
	Hospital-based	3	1537	1588	0.875	0.745-1.028	0.105	F	0.540	0.763	0.0	0.052
	HWE in controls											
	Equilibrium	10	6311	8179	**0.846**	**0.779-0.918**	**< 0.001**	F	0.630	0.472	0.0	0.543
	Disequilibrium	1	530	595	0.929	0.730-1.182	0.547	F	0.0	1.0	0.0	NA
	Quality score											
	High quality	9	6093	8043	**0.861**	**0.792-0.936**	**< 0.001**	F	8.714	0.367	8.199	0.705
	Low quality	2	748	731	0.812	0.657-1.003	0.053	F	0.176	0.675	0.0	NA

OR: odds ratio, CI: confidence interval, F: fixed model, R: random model, NA: not appreciable

Supplementary Table S16. Meta-analysis of the association between DICER (A > G; rs3742330) variant and cancer risk.

Comparison	Subgroups	No. of studies	Sample size		Test of association				Test of heterogeneity			Publication bias
			Cancer	Control	OR	95% CI	*P*-value	Model	Q test	*P*-value	I^2 (%)	*P*-value (Egger's)
**Allelic model (G allele *versus* A allele)**	**Overall**	16	11554	15586	0.974	0.889-1.066	0.562	R	34.92	0.003	57.04	0.942
	**Geographical region**											
	Asian	10	8074	11390	0.945	0.846-1.054	0.309	R	25.55	0.002	64.78	0.861
	European	2	952	976	1.212	0.949-1.548	0.124	F	1.615	0.204	38.07	NA
	American	3	2368	3062	1.026	0.844-1.247	0.796	F	1.829	0.401	0.0	0.012
	Turkish	1	160	158	0.626	0.333-1.174	0.144	F	0.0	1.0	0.0	NA
	**Cancer type**											
	Colorectal cancer	2	2216	3600	1.002	0.822-1.220	0.987	R	2.850	0.091	64.92	NA
	Bladder cancer	2	2820	2902	1.034	0.905-1.182	0.619	F	0.889	0.346	0.0	NA
	PTC	1	240	260	**0.553**	**0.342-0.894**	**0.016**	F	0.0	1.0	0.0	NA
	Endometrial cancer	1	160	158	0.626	0.333-1.174	0.144	F	0.0	1.0	0.0	NA
	Cervical precancerous lesions	1	592	592	**0.730**	**0.578-0.924**	**0.009**	F	0.0	1.0	0.0	NA
	Gastric cancer	1	1256	1004	**0.762**	**0.642-0.905**	**0.002**	F	0.0	1.0	0.0	NA
	Prostate cancer	1	706	636	1.029	0.723-1.462	0.875	F	0.0	1.0	0.0	NA
	Head/neck carcinoma	1	1150	3102	0.998	0.867-1.149	0.979	F	0.0	1.0	0.0	NA
	NHL	1	360	1058	0.883	0.580-1.345	0.563	F	0.0	1.0	0.0	NA
	HCC	1	294	418	1.020	0.755-1.378	0.900	F	0.0	1.0	0.0	NA
	Larynx cancer	1	246	340	**1.413**	**1.005-1.987**	**0.047**	F	0.0	1.0	0.0	NA
	Esophageal cancer	1	760	760	1.117	0.909-1.373	0.293	F	0.0	1.0	0.0	NA
	Lung cancer	1	200	200	1.359	0.914-2.023	0.130	F	0.0	1.0	0.0	NA
	Renal cell carcinoma	1	554	556	0.863	0.555-1.341	0.512	F	0.0	1.0	0.0	NA
	**Genotyping method**											
	TaqMan PCR	3	1772	1952	1.029	0.736-1.440	0.867	R	5.913	0.052	66.18	0.942
	RFLP-PCR	3	1350	1478	0.915	0.652-1.283	0.605	R	7.180	0.028	72.14	0.207
	Mass spectrometry	3	1552	1552	1.013	0.716-1.434	0.942	R	10.17	0.006	80.34	0.766
	HRMA	2	1962	1640	**0.808**	**0.692-0.942**	**0.007**	F	2.249	0.134	55.53	NA
	SNPlex technology	2	2008	2004	1.069	0.858-1.332	0.552	F	1.208	0.272	17.19	NA
	Other methods	3	2910	6960	0.950	0.866-1.043	0.281	F	0.860	0.651	0.0	0.812
	**Source of controls**											
	Population-based	8	4542	6664	0.959	0.820-1.123	0.606	R	17.97	0.012	61.04	0.606
	Hospital-based	8	7012	8922	0.969	0.854-1.100	0.628	R	16.76	0.019	58.24	0.752
	**HWE in controls**											
	Equilibrium	13	10556	14496	0.978	0.894-1.069	0.624	R	22.85	0.029	47.48	0.933
	Disequilibrium	3	998	1090	0.888	0.539-1.464	0.642	R	10.96	0.004	81.76	0.936
	**Quality score**											
	High quality	11	9482	13492	0.922	0.835-1.018	0.110	R	22.13	0.014	54.81	0.467
	Low quality	4	1312	1334	1.168	0.958-1.423	0.124	F	6.041	0.110	50.34	0.220
**Recessive model (GG versus AA+AG)**	**Overall**	16	5777	7793	0.892	0.794-1.003	0.055	F	12.88	0.611	0.0	0.308
	**Geographical region**											
	Asian	10	4037	5695	0.894	0.795-1.007	0.064	F	8.874	0.449	0.0	0.590
	European	2	476	488	0.710	0.245-2.058	0.528	F	1.247	0.264	19.80	NA
	American	3	1184	1531	1.260	0.481-3.302	0.638	F	0.104	0.949	0.0	0.167
	Turkish	1	80	79	0.187	0.021-1.641	0.130	F	0.0	1.0	0.0	NA
	**Cancer type**											
	Colorectal cancer	2	1108	1800	0.983	0.810-1.194	0.863	F	0.028	0.866	0.0	NA
	Bladder cancer	2	1410	1451	0.886	0.644-1.220	0.459	F	0.651	0.420	0.0	NA
	PTC	1	120	130	0.298	0.061-1.463	0.136	F	0.0	1.0	0.0	NA
	Endometrial cancer	1	80	79	0.187	0.021-1.641	0.130	F	0.0	1.0	0.0	NA
	Cervical precancerous lesions	1	296	296	1.000	0.638-1.566	1.000	F	0.0	1.0	0.0	NA
	Gastric cancer	1	628	502	**0.683**	**0.484-0.963**	**0.030**	F	0.0	1.0	0.0	NA
	Prostate cancer	1	353	318	0.899	0.287-2.817	0.855	F	0.0	1.0	0.0	NA
	Head/neck carcinoma	1	575	1551	0.789	0.584-1.067	0.124	F	0.0	1.0	0.0	NA
	NHL	1	180	529	0.980	0.101-9.477	0.986	F	0.0	1.0	0.0	NA
	HCC	1	147	209	0.738	0.422-1.290	0.286	F	0.0	1.0	0.0	NA
	Larynx cancer	1	123	170	0.150	0.008-2.808	0.204	F	0.0	1.0	0.0	NA
	Esophageal cancer	1	380	380	1.092	0.724-1.646	0.675	F	0.0	1.0	0.0	NA
	Lung cancer	1	100	100	1.346	0.683-2.653	0.390	F	0.0	1.0	0.0	NA
	Renal cell carcinoma	1	277	278	1.004	0.062-16.13	0.998	F	0.0	1.0	0.0	NA
	**Genotyping method**											
	TaqMan PCR	3	886	976	0.807	0.582-1.119	0.198	F	3.116	0.211	35.82	0.067
	RFLP-PCR	3	675	739	0.887	0.661-1.190	0.424	F	2.728	0.256	26.68	0.124
	Mass spectrometry	3	776	776	1.093	0.829-1.442	0.527	F	0.513	0.774	0.0	0.375
	HRMA	2	981	820	**0.698**	**0.502-0.971**	**0.033**	F	0.205	0.651	0.0	NA
	SNPlex technology	2	1004	1002	1.332	0.459-3.860	0.598	F	0.047	0.829	0.0	NA

	Other methods	3	1455	3480	0.900	0.750-1.081	0.261	F	1.164	0.559	0.0	0.913
	**Source of controls**											
	Population-based	8	2271	3332	0.865	0.712-1.051	0.144	F	5.418	0.609	0.0	0.249
	Hospital-based	8	3506	4461	0.908	0.785-1.050	0.193	F	7.312	0.397	4.265	0.857
	**HWE in controls**											
	Equilibrium	13	5278	7248	0.892	0.791-1.007	0.064	F	9.225	0.684	0.0	0.956
	Disequilibrium	3	499	545	0.897	0.581-1.385	0.623	F	3.658	0.161	45.33	0.079
	**Quality score**											
	High quality	11	4741	6746	**0.871**	**0.769-0.987**	**0.030**	F	6.992	0.726	0.0	0.650
	Low quality	4	656	667	0.996	0.573-1.732	0.990	F	4.670	0.198	35.76	0.017
**Dominant model (AG+GG versus AA)**	**Overall**	16	5777	7793	0.979	0.856-1.120	0.761	R	51.99	< 0.001	71.15	0.758
	**Geographical region**											
	Asian	10	4037	5695	0.949	0.788-1.143	0.581	R	36.92	< 0.001	75.62	0.980
	European	2	476	488	1.602	0.668-3.841	0.291	R	6.784	0.009	85.26	NA
	American	3	1184	1531	1.019	0.827-1.254	0.863	F	1.837	0.399	0.0	0.004
	Turkish	1	80	79	0.707	0.346-1.445	0.342	F	0.0	1.0	0.0	NA
	**Cancer type**											
	Colorectal cancer	2	1108	1800	1.026	0.678-1.553	0.904	R	5.623	0.018	82.22	NA
	Bladder cancer	2	1410	1451	1.089	0.922-1.286	0.315	F	0.243	0.622	0.0	NA
	PTC	1	120	130	**0.544**	**0.314-0.943**	**0.030**	F	0.0	1.0	0.0	NA
	Endometrial cancer	1	80	79	0.707	0.346-1.445	0.342	F	0.0	1.0	0.0	NA
	Cervical precancerous lesions	1	296	296	**0.528**	**0.377-0.740**	**< 0.001**	F	0.0	1.0	0.0	NA
	Gastric cancer	1	628	502	**0.708**	**0.556-0.902**	**0.005**	F	0.0	1.0	0.0	NA
	Prostate cancer	1	353	318	1.047	0.711-1.543	0.815	F	0.0	1.0	0.0	NA
	Head/neck carcinoma	1	575	1551	1.105	0.909-1.344	0.316	F	0.0	1.0	0.0	NA
	NHL	1	180	529	0.869	0.555-1.361	0.539	F	0.0	1.0	0.0	NA
	HCC	1	147	209	1.286	0.813-2.034	0.282	F	0.0	1.0	0.0	NA
	Larynx cancer	1	123	170	**2.560**	**1.477-4.436**	**0.001**	F	0.0	1.0	0.0	NA
	Esophageal cancer	1	380	380	1.201	0.892-1.615	0.227	F	0.0	1.0	0.0	NA
	Lung cancer	1	100	100	1.547	0.865-2.767	0.142	F	0.0	1.0	0.0	NA
	Renal cell carcinoma	1	277	278	0.848	0.533-1.351	0.489	F	0.0	1.0	0.0	NA
	**Genotyping method**											
	TaqMan PCR	3	886	976	1.256	0.666-2.367	0.482	R	10.56	0.005	81.06	0.798
	RFLP-PCR	3	675	739	1.005	0.617-1.636	0.984	R	7.845	0.020	74.50	0.458
	Mass spectrometry	3	776	776	0.971	0.512-1.838	0.927	R	16.55	< 0.001	87.91	0.819
	HRMA	2	981	820	0.835	0.572-1.220	0.352	R	2.824	0.093	64.59	NA
	SNPlex technology	2	1004	1002	1.064	0.841-1.346	0.605	F	1.221	0.269	18.10	NA
	Other methods	3	1455	3480	0.954	0.837-1.088	0.485	F	3.952	0.139	49.39	0.875
	**Source of controls**											
	Population-based	8	2271	3332	1.005	0.755-1.339	0.970	R	33.81	< 0.001	79.30	0.910
	Hospital-based	8	3506	4461	0.978	0.823-1.162	0.801	R	17.63	0.014	60.30	0.626
	**HWE in controls**											
	Equilibrium	13	5278	7248	1.005	0.884-1.141	0.943	R	26.39	0.009	54.53	0.831
	Disequilibrium	3	499	545	0.979	0.353-2.711	0.967	R	23.13	< 0.001	91.35	0.620
	**Quality score**											
	High quality	11	4741	6746	0.910	0.773-1.072	0.261	R	33.94	< 0.001	70.54	0.495
	Low quality	4	656	667	1.329	0.805-2.192	0.266	R	10.16	0.017	70.47	0.919
**Homozygote model (GG *versus* AA)**	**Overall**	16	3525	4713	**0.878**	**0.774-0.998**	**0.046**	F	18.31	0.247	18.08	0.644
	**Geographical region**											
	Asian	10	2157	3042	0.886	0.740-1.060	0.187	R	15.08	0.089	40.32	0.940
	European	2	314	332	0.787	0.271-2.288	0.660	F	0.468	0.494	0.0	NA
	American	3	991	1278	1.269	0.484-3.329	0.628	F	0.134	0.935	0.0	0.164
	Turkish	1	63	61	0.181	0.020-1.594	0.123	F	0.0	1.0	0.0	NA
	**Cancer type**											
	Colorectal cancer	2	576	922	0.957	0.769-1.191	0.693	F	1.648	0.199	39.32	NA
	Bladder cancer	2	974	1029	0.930	0.666-1.301	0.673	F	0.579	0.447	0.0	NA
	PTC	1	93	89	0.257	0.052-1.275	0.096	F	0.0	1.0	0.0	NA
	Endometrial cancer	1	63	61	0.181	0.020-1.594	0.123	F	0.0	1.0	0.0	NA
	Cervical precancerous lesions	1	179	135	0.672	0.411-1.099	0.113	F	0.0	1.0	0.0	NA
	Gastric cancer	1	344	256	**0.582**	**0.402-0.845**	**0.004**	F	0.0	1.0	0.0	NA
	Prostate cancer	1	291	265	0.909	0.289-2.853	0.870	F	0.0	1.0	0.0	NA
	Head/neck carcinoma	1	288	853	0.862	0.625-1.189	0.364	F	0.0	1.0	0.0	NA
	NHL	1	151	433	0.956	0.099-9.257	0.969	F	0.0	1.0	0.0	NA
	HCC	1	65	113	0.926	0.490-1.748	0.812	F	0.0	1.0	0.0	NA
	Larynx cancer	1	23	67	0.300	0.016-5.793	0.426	F	0.0	1.0	0.0	NA
	Esophageal cancer	1	184	196	1.212	0.774-1.899	0.401	F	0.0	1.0	0.0	NA
	Lung cancer	1	55	60	1.671	0.780-3.578	0.187	F	0.0	1.0	0.0	NA
	Renal cell carcinoma	1	239	234	0.979	0.061-15.74	0.988	F	0.0	1.0	0.0	NA
	**Genotyping method**											
	TaqMan PCR	3	459	546	0.847	0.601-1.194	0.343	F	2.500	0.287	19.99	0.224

	RFLP-PCR	3	359	421	1.042	0.748-1.451	0.808	F	3.499	0.174	42.84	0.042
	Mass spectrometry	3	418	391	1.056	0.641-1.742	0.830	R	4.951	0.084	59.61	0.686
	HRMA	2	635	521	**0.608**	**0.427-0.866**	**0.006**	F	0.524	0.469	0.0	NA
	SNPlex technology	2	840	845	1.351	0.466-3.920	0.580	F	0.061	0.806	0.0	NA
	Other methods	3	814	1989	0.868	0.709-1.063	0.170	F	0.010	0.995	0.0	0.470
	**Source of controls**											
	Population-based	8	1362	1952	0.873	0.708-1.078	0.207	F	5.934	0.547	0.0	0.316
	Hospital-based	8	2163	2761	0.907	0.705-1.167	0.447	R	12.37	0.089	43.42	0.858
	**HWE in controls**											
	Equilibrium	13	3260	4450	0.903	0.791-1.030	0.129	F	14.47	0.272	17.07	0.730
	Disequilibrium	3	265	263	0.618	0.385-0.993	0.047	F	1.564	0.457	0.0	0.263
	**Quality score**											
	High quality	11	2909	4064	**0.842**	**0.735-0.965**	**0.013**	F	10.69	0.382	6.464	0.811
	Low quality	4	432	453	1.118	0.616-2.029	0.714	F	4.645	0.200	35.42	0.069
**Heterozygote model (AG *versus* AA)**	**Overall**	16	5201	6891	0.997	0.866-1.147	0.964	R	52.15	< 0.001	71.23	0.754
	**Geographical region**											
	Asian	10	3477	4817	0.972	0.797-1.186	0.780	R	37.83	< 0.001	76.21	0.951
	European	2	470	478	1.648	0.672-4.044	0.275	R	6.924	0.009	85.56	NA
	American	3	1175	1522	1.010	0.817-1.248	0.929	F	1.692	0.429	0.0	0.001
	Turkish	1	79	74	0.853	0.401-1.815	0.680	F	0.0	1.0	0.0	NA
	**Cancer type**											
	Colorectal cancer	2	901	1458	1.034	0.655-1.633	0.885	R	6.042	0.014	83.45	NA
	Bladder cancer	2	1332	1359	1.114	0.937-1.325	0.221	F	0.035	0.851	0.0	NA
	PTC	1	118	123	0.593	0.336-1.049	0.073	F	0.0	1.0	0.0	NA
	Endometrial cancer	1	79	74	0.853	0.401-1.815	0.680	F	0.0	1.0	0.0	NA
	Cervical precancerous lesions	1	251	251	**0.488**	**0.341-0.698**	**< 0.001**	F	0.0	1.0	0.0	NA
	Gastric cancer	1	557	423	**0.749**	**0.580-0.966**	**0.026**	F	0.0	1.0	0.0	NA
	Prostate cancer	1	347	312	1.063	0.710-1.591	0.766	F	0.0	1.0	0.0	NA
	Head/neck carcinoma	1	513	1345	1.177	0.959-1.444	0.118	F	0.0	1.0	0.0	NA
	NHL	1	179	526	0.866	0.549-1.365	0.535	F	0.0	1.0	0.0	NA
	HCC	1	124	167	1.444	0.892-2.338	0.135	F	0.0	1.0	0.0	NA
	Larynx cancer	1	123	166	**2.659**	**1.533-4.614**	**0.001**	F	0.0	1.0	0.0	NA
	Esophageal cancer	1	325	329	1.197	0.877-1.634	0.256	F	0.0	1.0	0.0	NA
	Lung cancer	1	76	81	1.488	0.791-2.798	0.218	F	0.0	1.0	0.0	NA
	Renal cell carcinoma	1	276	277	0.845	0.528-1.353	0.484	F	0.0	1.0	0.0	NA
	**Genotyping method**											
	TaqMan PCR	3	814	880	1.368	0.740-2.530	0.317	R	9.383	0.009	78.68	0.736
	RFLP-PCR	3	574	616	1.085	0.674-1.747	0.736	R	6.801	0.033	70.59	0.535
	Mass spectrometry	3	652	661	0.932	0.469-1.849	0.840	R	16.84	< 0.001	88.12	0.857
	HRMA	2	904	735	0.827	0.667-1.026	0.084	F	2.077	0.150	51.86	NA
	SNPlex technology	2	996	996	1.053	0.829-1.338	0.670	F	1.134	0.287	11.80	NA
	Other methods	3	1261	3003	0.963	0.743-1.248	0.776	R	5.851	0.054	65.82	0.854
	**Source of controls**											
	Population-based	8	2077	2970	1.036	0.759-1.413	0.824	R	36.76	< 0.001	80.96	0.874
	Hospital-based	8	3124	3921	0.996	0.845-1.173	0.958	R	14.64	0.041	52.17	0.552
	**HWE in controls**											
	Equilibrium	13	4748	6400	1.029	0.906-1.169	0.661	R	24.16	0.019	50.33	0.870
	Disequilibrium	3	453	491	1.026	0.339-3.106	0.964	R	25.59	< 0.001	92.18	0.593
	**Quality score**											
	High quality	11	4251	5929	0.931	0.780-1.110	0.426	R	36.09	< 0.001	72.29	0.496
	Low quality	4	625	633	1.396	0.861-2.263	0.176	R	8.661	0.034	65.36	0.889

OR: odds ratio, CI: confidence interval, F: fixed model, R: random model, NA: not appreciable

Supplementary Table S17. Meta-analysis of the association between TARBP2 (G > A; rs784567) variant and cancer risk.

Comparison	Subgroups	No. of studies	Sample size		Test of association				Test of heterogeneity			Publication bias
			Cancer	Control	OR	95% CI	*P*-value	Model	Q test	*P*-value	I^2 (%)	*P*-value (Egger's)
**Allelic model (A allele *versus* G allele)**	**Overall**	5	3186	3664	**0.884**	**0.783-0.998**	**0.046**	R	30.08	< 0.001	86.70	0.301
	**Geographical region**											
	European	3	1160	1652	0.708	0.372-1.346	0.292	R	29.67	< 0.001	93.26	0.205
	American	2	2026	2012	0.891	0.788-1.009	0.068	F	0.007	0.935	0.0	NA
	**Cancer type**											
	Prostate cancer	1	710	636	1.213	0.978-1.504	0.078	F	0.0	1.0	0.0	NA
	Larynx Cancer	1	256	340	**0.414**	**0.296-0.580**	**< 0.001**	F	0.0	1.0	0.0	NA
	CLL	1	194	676	**0.684**	**0.495-0.947**	**0.022**	F	0.0	1.0	0.0	NA
	Bladder cancer	1	1474	1458	0.894	0.774-1.034	0.130	F	0.0	1.0	0.0	NA
	Renal cell carcinoma	1	552	554	0.884	0.698-1.119	0.306	F	0.0	1.0	0.0	NA
	**Genotyping method**											
	TaqMan PCR	2	450	1016	**0.534**	**0.326-0.873**	**0.012**	R	4.438	0.035	77.47	NA
	SNPlex technology	2	2026	2012	0.891	0.788-1.009	0.068	F	0.007	0.935	0.0	NA
	Other methods	1	710	636	1.213	0.978-1.504	0.078	F	0.0	1.0	0.0	NA
	**Source of controls**											
	Population-based	4	1712	2206	0.753	0.492-1.154	0.193	R	29.82	< 0.001	89.94	0.075
	Hospital-based	1	1474	1458	0.894	0.774-1.034	0.130	F	0.0	1.0	0.0	NA
	**HWE in controls**											
	Equilibrium	3	2220	2688	**0.862**	**0.768-0.967**	**0.012**	F	2.234	0.327	10.49	0.370
	Disequilibrium	2	966	976	0.715	0.249-2.049	0.532	R	27.77	< 0.001	96.40	NA
	**Quality score**											
	High quality	3	2220	2688	**0.862**	**0.768-0.967**	**0.012**	F	2.234	0.327	10.49	0.370
	Low quality	2	966	976	0.715	0.249-2.049	0.532	R	27.77	< 0.001	96.40	NA
**Recessive model (AA versus GG+GA)**	**Overall**	5	1593	1832	0.860	0.706-1.048	0.135	R	13.29	0.010	69.91	0.189
	**Geographical region**											
	European	3	580	826	0.651	0.288-1.475	0.304	R	12.57	0.002	84.09	0.170
	American	2	1013	1006	0.875	0.714-1.073	0.200	F	0.719	0.397	0.0	NA
	**Cancer type**											
	Prostate cancer	1	355	318	1.175	0.819-1.688	0.381	F	0.0	1.0	0.0	NA
	Larynx Cancer	1	128	170	**0.223**	**0.096-0.521**	**0.001**	F	0.0	1.0	0.0	NA
	CLL	1	97	338	0.826	0.471-1.449	0.504	F	0.0	1.0	0.0	NA
	Bladder cancer	1	737	729	0.923	0.727-1.170	0.506	F	0.0	1.0	0.0	NA
	Renal cell carcinoma	1	276	277	0.755	0.508-1.123	0.166	F	0.0	1.0	0.0	NA
	**Genotyping method**											
	TaqMan PCR	2	225	508	0.447	0.124-1.607	0.217	R	6.355	0.012	84.26	NA
	SNPlex technology	2	1013	1006	0.875	0.714-1.073	0.200	F	0.719	0.397	0.0	NA
	Other methods	1	355	318	1.175	0.819-1.688	0.381	F	0.0	1.0	0.0	NA
	**Source of controls**											
	Population-based	4	856	1103	0.706	0.420-1.188	0.190	R	12.99	0.005	76.90	0.127
	Hospital-based	1	737	729	0.923	0.727-1.170	0.506	F	0.0	1.0	0.0	NA
	**HWE in controls**											
	Equilibrium	3	1110	1344	0.869	0.718-1.053	0.152	F	0.755	0.686	0.0	0.458
	Disequilibrium	2	483	488	0.536	0.106-2.726	0.453	R	12.48	< 0.001	91.99	NA
	**Quality score**											
	High quality	3	1110	1344	0.869	0.718-1.053	0.152	F	0.755	0.686	0.0	0.458
	Low quality	2	483	488	0.536	0.106-2.726	0.453	R	12.48	< 0.001	91.99	NA
**Dominant model (GA+AA versus GG)**	**Overall**	5	1593	1832	0.828	0.679-1.008	0.060	R	35.55	< 0.001	88.75	0.257
	**Geographical region**											
	European	3	580	826	0.530	0.179-1.570	0.252	R	34.54	< 0.001	94.21	0.029
	American	2	1013	1006	0.840	0.687-1.027	0.090	F	0.475	0.491	0.0	NA
	**Cancer type**											
	Prostate cancer	1	355	318	1.367	0.986-1.895	0.060	F	0.0	1.0	0.0	NA
	Larynx Cancer	1	128	170	**0.213**	**0.119-0.379**	**< 0.001**	F	0.0	1.0	0.0	NA
	CLL	1	97	338	**0.481**	**0.298-0.777**	**0.003**	F	0.0	1.0	0.0	NA
	Bladder cancer	1	737	729	0.804	0.635-1.019	0.071	F	0.0	1.0	0.0	NA
	Renal cell carcinoma	1	276	277	0.941	0.644-1.374	0.752	F	0.0	1.0	0.0	NA
	**Genotyping method**											
	TaqMan PCR	2	225	508	**0.325**	**0.146-0.724**	**0.006**	R	4.548	0.033	78.01	NA
	SNPlex technology	2	1013	1006	0.840	0.687-1.027	0.090	F	0.475	0.491	0.0	NA
	Other methods	1	355	318	1.367	0.986-1.895	0.060	F	0.0	1.0	0.0	NA
	**Source of controls**											
	Population-based	4	856	1103	0.620	0.298-1.289	0.200	R	35.55	< 0.001	91.56	0.001
	Hospital-based	1	737	729	0.804	0.635-1.019	0.071	F	0.0	1.0	0.0	NA
	**HWE in controls**											

	Equilibrium	3	1110	1344	0.743	0.539-1.024	0.070	R	4.898	0.086	59.16	0.638
	Disequilibrium	2	483	488	0.548	0.089-3.392	0.518	R	30.18	< 0.001	96.69	NA
	**Quality score**											
	High quality	3	1110	1344	0.743	0.539-1.024	0.070	R	4.898	0.086	59.16	0.638
	Low quality	2	483	488	0.548	0.089-3.392	0.518	R	30.18	< 0.001	96.69	NA
**Homozygote model (AA *versus* GG)**	**Overall**	5	808	887	**0.776**	**0.608-0.992**	**0.043**	R	30.40	< 0.001	86.84	0.184
	**Geographical region**											
	European	3	301	392	0.415	0.099-1.739	0.229	R	30.37	< 0.001	93.42	0.041
	American	2	507	495	0.791	0.617-1.014	0.065	F	0.017	0.897	0.0	NA
	**Cancer type**											
	Prostate cancer	1	186	179	1.404	0.925-2.131	0.111	F	0.0	1.0	0.0	NA
	Larynx Cancer	1	58	56	**0.082**	**0.032-0.215**	**< 0.001**	F	0.0	1.0	0.0	NA
	CLL	1	57	157	**0.519**	**0.276-0.979**	**0.043**	F	0.0	1.0	0.0	NA
	Bladder cancer	1	376	353	0.799	0.597-1.069	0.131	F	0.0	1.0	0.0	NA
	Renal cell carcinoma	1	131	142	0.770	0.478-1.241	0.283	F	0.0	1.0	0.0	NA
	**Genotyping method**											
	TaqMan PCR	2	115	213	0.215	0.035-1.302	0.094	R	9.887	0.002	89.89	NA
	SNPlex technology	2	507	495	0.791	0.617-1.014	0.065	F	0.017	0.897	0.0	NA
	Other methods	1	186	179	1.404	0.925-2.131	0.111	F	0.0	1.0	0.0	NA
	**Source of controls**											
	Population-based	4	432	534	0.505	0.203-1.258	0.142	R	30.37	< 0.001	90.12	0.021
	Hospital-based	1	376	353	0.799	0.597-1.069	0.131	F	0.0	1.0	0.0	NA
	**HWE in controls**											
	Equilibrium	3	564	652	**0.748**	**0.594-0.943**	**0.014**	F	1.486	0.476	0.0	0.352
	Disequilibrium	2	244	235	0.352	0.022-5.661	0.461	R	28.32	< 0.001	96.47	NA
	**Quality score**											
	High quality	3	564	652	**0.748**	**0.594-0.943**	**0.014**	F	1.486	0.476	0.0	0.352
	Low quality	2	244	235	0.352	0.022-5.661	0.461	R	28.32	< 0.001	96.47	NA
**Heterozygote model (GA *versus* GG)**	**Overall**	5	1248	1396	0.849	0.686-1.050	0.130	R	28.94	< 0.001	86.18	0.304
	**Geographical region**											
	European	3	468	646	0.553	0.200-1.530	0.254	R	27.12	< 0.001	92.62	0.035
	American	2	780	750	0.864	0.698-1.069	0.178	F	1.031	0.310	3.010	NA
	**Cancer type**											
	Prostate cancer	1	269	250	1.350	0.950-1.918	0.094	F	0.0	1.0	0.0	NA
	Larynx Cancer	1	121	135	**0.253**	**0.140-0.456**	**< 0.001**	F	0.0	1.0	0.0	NA
	CLL	1	78	261	**0.465**	**0.278-0.780**	**0.004**	F	0.0	1.0	0.0	NA
	Bladder cancer	1	561	544	0.806	0.628-1.036	0.093	F	0.0	1.0	0.0	NA
	Renal cell carcinoma	1	219	206	1.031	0.690-1.539	0.883	F	0.0	1.0	0.0	NA
	**Genotyping method**											
	TaqMan PCR	2	199	396	0.357	0.242-0.526	< 0.001	F	2.330	0.127	57.08	NA
	SNPlex technology	2	780	750	0.864	0.698-1.069	0.178	F	1.031	0.310	3.010	NA
	Other methods	1	269	250	1.350	0.950-1.918	0.094	F	0.0	1.0	0.0	NA
	**Source of controls**											
	Population-based	4	687	852	0.655	0.325-1.319	0.236	R	28.94	< 0.001	89.63	0.002
	Hospital-based	1	561	544	0.806	0.628-1.036	0.093	F	0.0	1.0	0.0	NA
	**HWE in controls**											
	Equilibrium	3	858	1011	0.757	0.522-1.098	0.142	R	5.755	0.056	65.25	0.706
	Disequilibrium	2	390	385	0.594	0.115-3.067	0.534	R	22.91	< 0.001	95.64	NA
	**Quality score**											
	High quality	3	858	1011	0.757	0.522-1.098	0.142	R	5.755	0.056	65.25	0.706
	Low quality	2	390	385	0.594	0.115-3.067	0.534	R	22.91	< 0.001	95.64	NA

OR: odds ratio, CI: confidence interval, F: fixed model, R: random model, NA: not appreciable.

Supplementary Table S18. Meta-analysis of the association between AGO1 (A > G; rs595961) variant and cancer risk.

Comparison	Subgroups	No. of studies	Sample size		Test of association				Test of heterogeneity			Publication bias
			**Cancer**	**Control**	**OR**	**95% CI**	***P*-value**	**Model**	**Q test**	***P*-value**	**I^2 (%)**	***P*-value (Egger's)**
**Allelic model (G allele *versus* A allele)**	**Overall**	5	3382	3706	1.008	0.885-1.149	0.903	R	15.62	0.004	74.40	0.642
	**Geographical region**											
	Asian	2	1142	984	**1.445**	**1.151-1.815**	**0.002**	F	0.035	0.852	0.0	NA
	European	1	206	678	1.055	0.692-1.608	0.804	F	0.0	1.0	0.0	NA
	American	2	2034	2044	**0.816**	**0.687-0.968**	**0.020**	F	0.095	0.757	0.0	NA
	**Cancer type**											
	Lung cancer	2	1142	984	**1.445**	**1.151-1.815**	**0.002**	F	0.035	0.852	0.0	NA
	Bladder cancer	1	1480	1482	0.830	0.677-1.017	0.072	F	0.0	1.0	0.0	NA
	CLL	1	206	678	1.055	0.692-1.608	0.804	F	0.0	1.0	0.0	NA
	Renal cell carcinoma	1	554	562	0.782	0.568-1.076	0.130	F	0.0	1.0	0.0	NA
	**Genotyping method**											
	TaqMan PCR	2	1152	1468	**1.340**	**1.080-1.664**	**0.008**	F	1.680	0.195	40.47	NA
	SNPlex technology	2	2034	2044	**0.816**	**0.687-0.968**	**0.020**	F	0.095	0.757	0.0	NA
	Other methods	1	196	194	1.380	0.806-2.360	0.240	F	0.0	1.0	0.0	NA
	**Source of controls**											
	Population-based	2	760	1240	0.872	0.676-1.125	0.291	F	1.234	0.267	18.94	NA
	Hospital-based	3	2622	2466	1.160	0.753-1.786	0.500	R	12.69	0.002	84.25	0.655
	**HWE in controls**											
	Equilibrium	5	3382	3706	1.045	0.790-1.383	0.757	R	15.62	0.004	74.40	0.642
	**Quality score**											
	High quality	4	3186	3512	0.999	0.733-1.363	0.997	R	14.23	0.003	78.92	0.889
	Low quality	1	196	194	1.380	0.806-2.360	0.240	F	0.0	1.0	0.0	NA
**Recessive model (GG versus AA+AG)**	**Overall**	5	1691	1853	0.946	0.601-1.490	0.811	F	4.620	0.329	13.42	0.114
	**Geographical region**											
	Asian	2	571	492	1.607	0.685-3.770	0.276	F	1.498	0.221	33.25	NA
	European	1	103	339	1.329	0.408-4.331	0.637	F	0.0	1.0	0.0	NA
	American	2	1017	1022	0.664	0.364-1.214	0.184	F	0.0	0.987	0.0	NA
	**Cancer type**											
	Lung cancer	2	571	492	1.607	0.685-3.770	0.276	F	1.498	0.221	33.25	NA
	Bladder cancer	1	740	741	0.662	0.317-1.384	0.273	F	0.0	1.0	0.0	NA
	CLL	1	103	339	1.329	0.408-4.331	0.637	F	0.0	1.0	0.0	NA
	Renal cell carcinoma	1	277	281	0.669	0.235-1.906	0.452	F	0.0	1.0	0.0	NA
	**Genotyping method**											
	TaqMan PCR	2	576	734	1.353	0.664-2.756	0.405	F	0.001	0.970	0.0	NA
	SNPlex technology	2	1017	1022	0.664	0.364-1.214	0.184	F	0.0	0.987	0.0	NA
	Other methods	1	98	97	9.286	0.493-174.8	0.137	F	0.0	1.0	0.0	NA
	**Source of controls**											
	Population-based	2	380	620	0.905	0.413-1.981	0.803	F	0.727	0.394	0.0	NA
	Hospital-based	3	1311	1233	0.968	0.554-1.691	0.908	F	3.874	0.144	48.38	0.312
	**HWE in controls**											
	Equilibrium	5	1691	1853	0.946	0.601-1.490	0.811	F	4.620	0.329	14.42	0.114
	**Quality score**											
	High quality	4	1593	1756	0.895	0.565-1.417	0.635	F	2.237	0.525	0.0	0.532
	Low quality	1	98	97	9.286	0.493-174.8	0.137	F	0.0	1.0	0.0	NA
**Dominant model (AG+GG versus AA)**	**Overall**	5	1691	1853	1.012	0.873-1.173	0.874	R	15.45	0.004	74.10	0.767
	**Geographical region**											
	Asian	2	571	492	**1.523**	**1.174-1.975**	**0.002**	F	0.494	0.482	0.0	NA
	European	1	103	339	1.025	0.631-1.667	0.920	F	0.0	1.0	0.0	NA
	American	2	1017	1022	0.805	0.664-0.977	0.028	F	0.120	0.729	0.0	NA
	**Cancer type**											
	Lung cancer	2	571	492	**1.523**	**1.174-1.975**	**0.002**	F	0.494	0.482	0.0	NA
	Bladder cancer	1	740	741	0.823	0.655-1.034	0.095	F	0.0	1.0	0.0	NA
	CLL	1	103	339	1.025	0.631-1.667	0.920	F	0.0	1.0	0.0	NA
	Renal cell carcinoma	1	277	281	0.763	0.530-1.097	0.145	F	0.0	1.0	0.0	NA
	**Genotyping method**											
	TaqMan PCR	2	576	734	1.420	1.108-1.819	0.006	F	2.330	0.127	57.07	NA
	SNPlex technology	2	1017	1022	**0.805**	**0.664-0.977**	**0.028**	F	0.120	0.729	0.0	NA
	Other methods	1	98	97	1.251	0.682-2.296	0.469	F	0.0	1.0	0.0	NA
	**Source of controls**											
	Population-based	2	380	620	0.848	0.634-1.135	0.268	F	0.912	0.340	0.0	NA
	Hospital-based	3	1311	1233	1.164	0.714-1.896	0.543	R	12.63	0.002	84.16	0.731
	**HWE in controls**											
	Equilibrium	5	1691	1853	1.039	0.757-1.426	0.812	R	15.45	0.004	74.10	0.767
	**Quality score**											

	High quality	4	1593	1756	1.008	0.702-1.448	0.965	R	14.95	0.002	79.93	0.916
	Low quality	1	98	97	1.251	0.682-2.296	0.469	F	0.0	1.0	0.0	NA
**Homozygote model (GG *versus* AA)**	**Overall**	5	1223	1353	0.953	0.604-1.504	0.836	F	5.680	0.224	29.58	0.174
	**Geographical region**											
	Asian	2	375	362	1.841	0.782-4.338	0.163	F	1.315	0.252	23.94	NA
	European	1	77	252	1.326	0.404-4.353	0.642	F	0.0	1.0	0.0	NA
	American	2	771	739	0.630	0.344-1.154	0.134	F	0.0	0.983	0.0	NA
	**Cancer type**											
	Lung cancer	2	375	362	1.841	0.782-4.338	0.163	F	1.315	0.252	23.94	NA
	Bladder cancer	1	563	541	0.633	0.302-1.327	0.226	F	0.0	1.0	0.0	NA
	CLL	1	77	252	1.326	0.404-4.353	0.642	F	0.0	1.0	0.0	NA
	Renal cell carcinoma	1	208	198	0.624	0.218-1.786	0.379	F	0.0	1.0	0.0	NA
	**Genotyping method**											
	TaqMan PCR	2	383	545	1.483	0.725-3.033	0.280	F	0.053	0.817	0.0	NA
	SNPlex technology	2	771	739	0.630	0.344-1.154	0.134	F	0.0	0.983	0.0	NA
	Other methods	1	69	69	9.550	0.504-180.8	0.133	F	0.0	1.0	0.0	NA
	**Source of controls**											
	Population-based	2	285	450	0.869	0.395-1.910	0.726	F	0.867	0.352	0.0	NA
	Hospital-based	3	938	903	1.229	0.442-3.415	0.692	R	4.733	0.094	57.74	0.384
	**HWE in controls**											
	Equilibrium	5	1223	1353	0.953	0.604-1.504	0.836	F	5.680	0.224	29.58	0.174
	**Quality score**											
	High quality	4	1154	1284	0.900	0.567-1.429	0.656	F	3.264	0.353	8.082	0.607
	Low quality	1	69	69	9.550	0.504-180.8	0.133	F	0.0	1.0	0.0	NA
**Heterozygote model (AG *versus* AA)**	**Overall**	5	1652	1808	0.983	0.839-1.151	0.829	R	13.42	0.009	70.20	0.903
	**Geographical region**											
	Asian	2	554	484	**1.487**	**1.139-1.942**	**0.004**	F	1.118	0.290	10.56	NA
	European	1	99	329	0.991	0.595-1.650	0.971	F	0.0	1.0	0.0	NA
	American	2	999	995	0.822	0.674-1.003	0.054	F	0.116	0.734	0.0	NA
	**Cancer type**											
	Lung cancer	2	554	484	**1.487**	**1.139-1.942**	**0.004**	F	1.118	0.290	10.56	NA
	Bladder cancer	1	728	723	0.840	0.664-1.063	0.146	F	0.0	1.0	0.0	NA
	CLL	1	99	329	0.991	0.595-1.650	0.971	F	0.0	1.0	0.0	NA
	Renal cell carcinoma	1	271	272	0.778	0.534-1.133	0.190	F	0.0	1.0	0.0	NA
	**Genotyping method**											
	TaqMan PCR	2	559	716	**1.414**	**1.095-1.825**	**0.008**	F	2.490	0.115	59.83	NA
	SNPlex technology	2	999	995	0.822	0.674-1.003	0.054	F	0.116	0.734	0.0	NA
	Other methods	1	94	97	1.099	0.591-2.044	0.764	F	0.0	1.0	0.0	NA
	**Source of controls**											
	Population-based	2	370	601	0.847	0.626-1.146	0.282	F	0.560	0.454	0.0	NA
	Hospital-based	3	1282	1207	1.135	0.710-1.813	0.597	R	11.04	0.004	81.88	0.810
	**HWE in controls**											
	Equilibrium	5	1652	1808	1.024	0.755-1.390	0.877	R	13.42	0.009	70.20	0.903
	**Quality score**											
	High quality	4	1558	1711	1.013	0.711-1.443	0.945	R	13.35	0.004	77.53	0.963
	Low quality	1	94	97	1.099	0.591-2.044	0.764	F	0.0	1.0	0.0	NA

OR: odds ratio, CI: confidence interval, F: fixed model, R: random model, NA: not appreciable

Supplementary Table S19. Meta-analysis of the association between AGO1 (G > A; rs636832) variant and cancer risk.

Comparison	Subgroups	No. of studies	Sample size		Test of association				Test of heterogeneity			Publication bias
			Cancer	Control	OR	95% CI	*P*-value	Model	Q test	*P*-value	I^2 (%)	*P*-value (Egger's)
**Allelic model (A allele *versus* G allele)**	**Overall**	6	4316	4856	0.967	0.866-1.080	0.556	F	8.727	0.120	42.70	**0.035**
	**Geographical region**											
	Asian	2	1452	1198	0.847	0.499-1.437	0.538	R	4.331	0.037	76.91	NA
	European	1	210	694	0.720	0.402-1.289	0.269	F	0.0	1.0	0.0	NA
	American	2	2030	2026	0.839	0.677-1.040	0.109	F	0.037	0.847	0.0	NA
	Latin American	1	624	938	1.103	0.901-1.351	0.342	F	0.0	1.0	0.0	NA
	**Cancer type**											
	Bladder cancer	1	1476	1472	0.828	0.641-1.069	0.147	F	0.0	1.0	0.0	NA
	CLL	1	210	694	0.720	0.402-1.289	0.269	F	0.0	1.0	0.0	NA
	CML	1	624	938	1.103	0.901-1.351	0.342	F	0.0	1.0	0.0	NA
	Gastric cancer	1	1256	1004	1.063	0.882-1.280	0.522	F	0.0	1.0	0.0	NA
	Lung cancer	1	196	194	**0.614**	**0.380-0.994**	**0.047**	F	0.0	1.0	0.0	NA
	Renal cell carcinoma	1	554	554	0.867	0.583-1.289	0.480	F	0.0	1.0	0.0	NA
	**Genotyping method**											
	TaqMan PCR	2	834	1632	1.054	0.870-1.276	0.593	F	1.842	0.175	45.70	NA
	SNPlex technology	2	2030	2026	0.839	0.677-1.040	0.109	F	0.037	0.847	0.0	NA
	Other methods	2	1452	1198	0.847	0.499-1.437	0.538	R	4.331	0.037	76.91	NA
	**Source of controls**											
	Population-based	2	764	1248	0.818	0.589-1.134	0.228	F	0.268	0.605	0.0	NA
	Hospital-based	4	3552	3608	0.943	0.775-1.149	0.562	R	7.314	0.063	58.98	**0.086**
	**HWE in controls**											
	Equilibrium	6	4316	4856	0.967	0.866-1.080	0.556	F	8.727	0.120	42.70	**0.035**
	**Quality score**											
	High quality	5	4120	4662	0.992	0.886-1.111	0.890	F	5.120	0.275	21.88	0.125
	Low quality	1	196	194	**0.614**	**0.380-0.994**	**0.047**	F	0.0	1.0	0.0	
**Recessive model (AA versus GG+GA)**	**Overall**	6	2158	2428	1.048	0.813-1.352	0.717	F	3.726	0.589	0.0	0.167
	**Geographical region**											
	Asian	2	726	599	0.974	0.632-1.500	0.903	F	0.526	0.468	0.0	NA
	European	1	105	347	0.466	0.024-9.104	0.615	F	0.0	1.0	0.0	NA
	American	2	1015	1013	0.983	0.271-3.566	0.979	F	2.680	0.102	62.69	NA
	Latin American	1	312	469	1.108	0.800-1.536	0.536	F	0.0	1.0	0.0	NA
	**Cancer type**											
	Bladder cancer	1	738	736	1.667	0.397-6.999	0.485	F	0.0	1.0	0.0	NA
	CLL	1	105	347	0.466	0.024-9.104	0.615	F	0.0	1.0	0.0	NA
	CML	1	312	469	1.108	0.800-1.536	0.536	F	0.0	1.0	0.0	NA
	Gastric cancer	1	628	502	1.023	0.651-1.609	0.921	F	0.0	1.0	0.0	NA
	Lung cancer	1	98	97	0.581	0.135-2.501	0.466	F	0.0	1.0	0.0	NA
	Renal cell carcinoma	1	277	277	0.110	0.006-2.044	0.139	F	0.0	1.0	0.0	NA
	**Genotyping method**											
	TaqMan PCR	2	417	816	1.097	0.793-1.517	0.575	F	0.322	0.570	0.0	NA
	SNPlex technology	2	1015	1013	0.983	0.271-3.566	0.979	F	2.680	0.102	62.69	NA
	Other methods	2	726	599	0.974	0.632-1.500	0.903	F	0.526	0.468	0.0	NA
	**Source of controls**											
	Population-based	2	382	624	0.224	0.028-1.798	0.159	F	0.464	0.496	0.0	NA
	Hospital-based	4	1776	1804	1.073	0.830-1.386	0.590	F	1.121	0.772	0.0	0.790
	**HWE in controls**											
	Equilibrium	6	2158	2428	1.048	0.813-1.352	0.717	F	3.726	0.589	0.0	0.167
	**Quality score**											
	High quality	5	2060	2331	1.068	0.825-1.382	0.619	F	3.079	0.545	0.0	0.319
	Low quality	1	98	97	0.581	0.135-2.501	0.466	F	0.0	1.0	0.0	NA
**Dominant model (GA+AA versus GG)**	**Overall**	6	2158	2428	0.941	0.819-1.081	0.392	F	8.968	0.110	44.25	0.224
	**Geographical region**											
	Asian	2	726	599	0.810	0.408-1.607	0.546	R	4.933	0.026	79.73	NA
	European	1	105	347	0.737	0.400-1.357	0.327	F	0.0	1.0	0.0	NA
	American	2	1015	1013	0.830	0.660-1.044	0.111	F	0.401	0.527	0.0	NA
	Latin American	1	312	469	1.172	0.839-1.638	0.353	F	0.0	1.0	0.0	NA
	**Cancer type**											
	Bladder cancer	1	738	736	0.792	0.603-1.039	0.093	F	0.0	1.0	0.0	NA
	CLL	1	105	347	0.737	0.400-1.357	0.327	F	0.0	1.0	0.0	NA
	CML	1	312	469	1.172	0.839-1.638	0.353	F	0.0	1.0	0.0	NA
	Gastric cancer	1	628	502	1.095	0.866-1.386	0.448	F	0.0	1.0	0.0	NA
	Lung cancer	1	98	97	**0.540**	**0.303-0.963**	**0.037**	F	0.0	1.0	0.0	NA
	Renal cell carcinoma	1	277	277	0.932	0.609-1.426	0.745	F	0.0	1.0	0.0	NA
	**Genotyping method**											
	TaqMan PCR	2	417	816	1.053	0.785-1.412	0.731	F	1.707	0.191	41.41	NA

	SNPlex technology	2	1015	1013	0.830	0.660-1.044	0.111	F	0.401	0.527	0.0	NA
	Other methods	2	726	599	0.810	0.408-1.607	0.546	R	4.933	0.026	79.73	NA
	Source of controls											
	Population-based	2	382	624	0.863	0.609-1.223	0.408	F	0.383	0.536	0.0	NA
	Hospital-based	4	1776	1804	0.916	0.699-1.201	0.527	R	8.305	0.040	63.88	0.402
	HWE in controls											
	Equilibrium	6	2158	2428	0.941	0.819-1.081	0.392	F	8.968	0.110	44.25	0.224
	Quality score											
	High quality	5	2060	2331	0.974	0.844-1.124	0.716	F	5.204	0.267	23.14	0.570
	Low quality	1	98	97	**0.540**	**0.303-0.963**	**0.037**	F	0.0	1.0	0.0	NA
**Homozygote model (AA *versus* GG)**	Overall	6	1536	1712	1.090	0.816-1.456	0.561	F	4.571	0.470	0.0	0.111
	Geographical region											
	Asian	2	435	359	0.989	0.635-1.542	0.962	F	1.123	0.289	10.96	NA
	European	1	90	286	0.448	0.023-8.746	0.596	F	0.0	1.0	0.0	NA
	American	2	855	828	0.950	0.262-3.449	0.938	F	2.593	0.107	61.43	NA
	Latin American	1	156	239	1.217	0.812-1.823	0.342	F	0.0	1.0	0.0	NA
	Cancer type											
	Bladder cancer	1	629	601	1.597	0.380-6.713	0.523	F	0.0	1.0	0.0	NA
	CLL	1	90	286	0.448	0.023-8.746	0.596	F	0.0	1.0	0.0	NA
	CML	1	156	239	1.217	0.812-1.823	0.342	F	0.0	1.0	0.0	NA
	Gastric cancer	1	367	304	1.067	0.670-1.699	0.785	F	0.0	1.0	0.0	NA
	Lung cancer	1	68	55	0.462	0.105-2.024	0.305	F	0.0	1.0	0.0	NA
	Renal cell carcinoma	1	226	227	0.110	0.006-2.048	0.139	F	0.0	1.0	0.0	NA
	Genotyping method											
	TaqMan PCR	2	246	525	1.195	0.800-1.783	0.384	F	0.427	0.514	0.0	NA
	SNPlex technology	2	855	828	0.950	0.262-3.449	0.938	F	2.593	0.107	61.43	NA
	Other methods	2	435	359	0.989	0.635-1.542	0.962	F	1.123	0.289	10.96	NA
	Source of controls											
	Population-based	2	316	513	0.219	0.027-1.764	0.154	F	0.437	0.509	0.0	NA
	Hospital-based	4	1220	1199	1.125	0.839-1.507	0.432	F	1.819	0.611	0.0	0.581
	HWE in controls											
	Equilibrium	6	1536	1712	1.090	0.816-1.456	0.561	F	4.571	0.470	0.0	0.111
	Quality score											
	High quality	5	1468	1657	1.128	0.839-1.515	0.425	F	3.222	0.521	0.0	0.247
	Low quality	1	68	55	0.462	0.105-2.024	0.305	F	0.0	1.0	0.0	NA
**Heterozygote model (GA *versus* GG)**	Overall	6	2020	2260	0.940	0.814-1.085	0.397	F	8.321	0.139	39.91	0.341
	Geographical region											
	Asian	2	677	558	0.822	0.420-1.609	0.567	R	4.457	0.035	77.56	NA
	European	1	105	344	0.773	0.419-1.427	0.411	F	0.0	1.0	0.0	NA
	American	2	1010	1006	0.835	0.662-1.054	0.129	F	1.011	0.315	1.094	NA
	Latin American	1	228	352	1.149	0.806-1.639	0.443	F	0.0	1.0	0.0	NA
	Cancer type											
	Bladder cancer	1	733	733	0.774	0.587-1.020	0.069	F	0.0	1.0	0.0	NA
	CLL	1	105	344	0.773	0.419-1.427	0.411	F	0.0	1.0	0.0	NA
	CML	1	228	352	1.149	0.806-1.639	0.443	F	0.0	1.0	0.0	NA
	Gastric cancer	1	582	466	1.101	0.861-1.407	0.445	F	0.0	1.0	0.0	NA
	Lung cancer	1	95	92	**0.549**	**0.303-0.997**	**0.049**	F	0.0	1.0	0.0	NA
	Renal cell carcinoma	1	277	273	1.006	0.654-1.550	0.977	F	0.0	1.0	0.0	NA
	Genotyping method											
	TaqMan PCR	2	333	696	1.040	0.765-1.414	0.801	F	1.203	0.273	16.88	NA
	SNPlex technology	2	1010	1006	0.835	0.662-1.054	0.129	F	1.011	0.315	1.094	NA
	Other methods	2	677	558	0.822	0.420-1.609	0.567	R	4.457	0.035	77.56	NA
	Source of controls											
	Population-based	2	382	617	0.922	0.648-1.312	0.653	F	0.475	0.491	0.0	NA
	Hospital-based	4	1638	1643	0.909	0.692-1.194	0.493	R	7.832	0.050	61.70	0.461
	HWE in controls											
	Equilibrium	6	2020	2260	0.940	0.814-1.085	0.397	F	8.321	0.139	39.91	0.341
	Quality score											
	High quality	5	1925	2168	0.971	0.838-1.126	0.701	F	5.016	0.286	20.25	0.774
	Low quality	1	95	92	**0.549**	**0.303-0.997**	**0.049**	F	0.0	1.0	0.0	NA

OR: odds ratio, CI: confidence interval, F: fixed model, R: random model, NA: not appreciable

Supplementary Table S20. Meta-analysis of the association between AGO2 (C > A; rs4961280) variant and cancer risk.

Comparison	Subgroups	No. of studies	Sample size		Test of association				Test of heterogeneity			Publication bias
			Cancer	Control	OR	95% CI	*P*-value	Model	Q test	*P*-value	I^2 (%)	*P*-value (Egger's)
**Allelic model (A allele *versus* C allele)**	**Overall**	5	3130	3532	0.895	0.787-1.019	0.093	F	3.463	0.483	0.0	0.103
	**Geographical region**											
	Asian	1	198	194	0.774	0.395-1.516	0.455	F	0.0	1.0	0.0	NA
	European	2	922	1334	**0.792**	**0.628-0.998**	**0.048**	F	1.375	0.241	27.25	NA
	American	2	2010	2004	0.957	0.816-1.123	0.593	F	0.142	0.706	0.0	NA
	**Cancer type**											
	Prostate cancer	1	710	636	0.855	0.656-1.113	0.244	F	0.0	1.0	0.0	NA
	CLL	1	212	698	**0.617**	**0.383-0.994**	**0.047**	F	0.0	1.0	0.0	NA
	Lung cancer	1	198	194	0.774	0.395-1.516	0.455	F	0.0	1.0	0.0	NA
	Bladder cancer	1	1458	1450	0.975	0.809-1.176	0.794	F	0.0	1.0	0.0	NA
	Renal cell carcinoma	1	552	554	0.910	0.669-1.238	0.549	F	0.0	1.0	0.0	NA
	**Genotyping method**											
	TaqMan PCR	2	922	1334	**0.792**	**0.628-0.998**	**0.048**	F	1.375	0.241	27.25	NA
	SNPlex technology	2	2010	2004	0.957	0.816-1.123	0.593	F	0.142	0.706	0.0	NA
	Other methods	1	198	194	0.774	0.395-1.516	0.455	F	0.0	1.0	0.0	NA
	**Source of controls**											
	Population-based	3	1474	1888	0.833	0.692-1.002	0.052	F	1.879	0.391	0.0	0.320
	Hospital-based	2	1656	1644	0.959	0.801-1.148	0.651	F	0.423	0.515	0.0	NA
	**HWE in controls**											
	Equilibrium	5	3130	3532	0.895	0.787-1.019	0.093	F	3.463	0.483	0.0	0.103
	**Quality score**											
	High quality	3	2222	2702	0.916	0.787-1.066	0.256	F	3.077	0.215	35.0	0.232
	Low quality	2	908	830	0.843	0.660-1.078	0.175	F	0.073	0.787	0.0	NA
**Recessive model (AA versus CC+CA)**	**Overall**	5	1565	1766	0.926	0.643-1.333	0.680	F	1.352	0.853	0.0	0.243
	**Geographical region**											
	Asian	1	99	97	2.970	0.120-73.79	0.507	F	0.0	1.0	0.0	NA
	European	2	461	667	0.840	0.435-1.619	0.602	F	0.739	0.390	0.0	NA
	American	2	1005	1002	0.947	0.609-1.474	0.810	F	0.011	0.915	0.0	NA
	**Cancer type**											
	Prostate cancer	1	355	318	0.717	0.340-1.516	0.384	F	0.0	1.0	0.0	NA
	CLL	1	106	349	1.423	0.361-5.602	0.614	F	0.0	1.0	0.0	NA
	Lung cancer	1	99	97	2.970	0.120-73.79	0.507	F	0.0	1.0	0.0	NA
	Bladder cancer	1	729	725	0.961	0.575-1.605	0.879	F	0.0	1.0	0.0	NA
	Renal cell carcinoma	1	276	277	0.909	0.380-2.177	0.831	F	0.0	1.0	0.0	NA
	**Genotyping method**											
	TaqMan PCR	2	461	667	0.840	0.435-1.619	0.602	F	0.739	0.390	0.0	NA
	SNPlex technology	2	1005	1002	0.947	0.609-1.474	0.810	F	0.011	0.915	0.0	NA
	Other methods	1	99	97	2.970	0.120-73.79	0.507	F	0.0	1.0	0.0	NA
	**Source of controls**											
	Population-based	3	737	944	0.864	0.511-1.460	0.585	F	0.759	0.684	0.0	0.131
	Hospital-based	2	828	822	0.988	0.595-1.640	0.963	F	0.462	0.497	0.0	NA
	**HWE in controls**											
	Equilibrium	5	1565	1766	0.926	0.643-1.333	0.680	F	1.352	0.853	0.0	0.243
	**Quality score**											
	High quality	3	1111	1351	0.984	0.646-1.499	0.941	F	0.318	0.853	0.0	0.497
	Low quality	2	454	415	0.772	0.372-1.599	0.486	F	0.712	0.399	0.0	NA
**Dominant model (CA+AA versus CC)**	**Overall**	5	1565	1766	0.872	0.751-1.014	0.075	F	4.978	0.290	19.64	**0.083**
	**Geographical region**											
	Asian	1	99	97	0.698	0.339-1.435	0.328	F	0.0	1.0	0.0	NA
	European	2	461	667	**0.750**	**0.572-0.983**	**0.037**	F	2.423	0.120	58.73	NA
	American	2	1005	1002	0.951	0.790-1.146	0.601	F	0.155	0.694	0.0	NA
	**Cancer type**											
	Prostate cancer	1	355	318	0.850	0.622-1.163	0.310	F	0.0	1.0	0.0	NA
	CLL	1	106	349	**0.519**	**0.303-0.888**	**0.017**	F	0.0	1.0	0.0	NA
	Lung cancer	1	99	97	0.698	0.339-1.435	0.328	F	0.0	1.0	0.0	NA
	Bladder cancer	1	729	725	0.973	0.782-1.211	0.810	F	0.0	1.0	0.0	NA
	Renal cell carcinoma	1	276	277	0.895	0.626-1.280	0.543	F	0.0	1.0	0.0	NA
	**Genotyping method**											
	TaqMan PCR	2	461	667	**0.750**	**0.572-0.983**	**0.037**	F	2.423	0.120	58.73	NA
	SNPlex technology	2	1005	1002	0.951	0.790-1.146	0.601	F	0.155	0.694	0.0	NA
	Other methods	1	99	97	0.698	0.339-1.435	0.328	F	0.0	1.0	0.0	NA
	**Source of controls**											
	Population-based	3	737	944	**0.800**	**0.645-0.992**	**0.043**	F	3.019	0.221	33.76	0.243
	Hospital-based	2	828	822	0.947	0.768-1.167	0.607	F	0.751	0.386	0.0	NA
	**HWE in controls**											
	Equilibrium	5	1565	1766	0.872	0.751-1.014	0.075	F	4.978	0.290	19.64	0.083

	Quality score											
	High quality	3	1111	1351	0.891	0.747-1.063	0.201	F	4.525	0.104	55.80	0.258
	Low quality	2	454	415	0.824	0.618-1.098	0.187	F	0.243	0.622	0.0	NA
**Homozygote model (AA *versus* CC)**	**Overall**	5	1137	1244	0.897	0.621-1.295	0.561	F	1.181	0.881	0.0	0.344
	**Geographical region**											
	Asian	1	84	76	2.749	0.110-68.49	0.538	F	0.0	1.0	0.0	NA
	European	2	332	458	0.781	0.402-1.515	0.464	F	0.491	0.483	0.0	NA
	American	2	721	710	0.934	0.598-1.460	0.766	F	0.024	0.878	0.0	NA
	**Cancer type**											
	Prostate cancer	1	243	210	0.685	0.322-1.460	0.328	F	0.0	1.0	0.0	NA
	CLL	1	89	248	1.201	0.304-4.749	0.794	F	0.0	1.0	0.0	NA
	Lung cancer	1	84	76	2.749	0.110-68.49	0.538	F	0.0	1.0	0.0	NA
	Bladder cancer	1	520	514	0.954	0.569-1.600	0.858	F	0.0	1.0	0.0	NA
	Renal cell carcinoma	1	201	196	0.881	0.365-2.123	0.777	F	0.0	1.0	0.0	NA
	**Genotyping method**											
	TaqMan PCR	2	332	458	0.781	0.402-1.515	0.464	F	0.491	0.483	0.0	NA
	SNPlex technology	2	721	710	0.934	0.598-1.460	0.766	F	0.024	0.878	0.0	NA
	Other methods	1	84	76	2.749	0.110-68.49	0.538	F	0.0	1.0	0.0	NA
	**Source of controls**											
	Population-based	3	533	654	0.815	0.480-1.385	0.450	F	0.537	0.765	0.0	0.218
	Hospital-based	2	604	590	0.980	0.588-1.633	0.937	F	0.406	0.524	0.0	NA
	**HWE in controls**											
	Equilibrium	5	1137	1244	0.897	0.621-1.295	0.561	F	1.181	0.881	0.0	0.344
	**Quality score**											
	High quality	3	810	958	0.957	0.626-1.463	0.839	F	0.139	0.933	0.0	0.647
	Low quality	2	327	286	0.737	0.353-1.539	0.417	F	0.679	0.410	0.0	NA
**Heterozygote model (CA *versus* CC)**	**Overall**	5	1508	1701	0.871	0.745-1.019	0.085	F	6.021	0.198	33.56	0.070
	**Geographical region**											
	Asian	1	98	97	0.654	0.315-1.360	0.256	F	0.0	1.0	0.0	NA
	European	2	445	644	0.673	0.370-1.224	0.194	R	3.400	0.065	70.59	NA
	American	2	965	960	0.954	0.785-1.160	0.636	F	0.145	0.704	0.0	NA
	**Cancer type**											
	Prostate cancer	1	342	302	0.875	0.631-1.212	0.421	F	0.0	1.0	0.0	NA
	CLL	1	103	342	**0.472**	**0.267-0.834**	**0.010**	F	0.0	1.0	0.0	NA
	Lung cancer	1	98	97	0.654	0.315-1.360	0.256	F	0.0	1.0	0.0	NA
	Bladder cancer	1	699	694	0.976	0.777-1.227	0.838	F	0.0	1.0	0.0	NA
	Renal cell carcinoma	1	266	266	0.897	0.617-1.303	0.568	F	0.0	1.0	0.0	NA
	**Genotyping method**											
	TaqMan PCR	2	445	644	0.673	0.370-1.224	0.194	R	3.400	0.065	70.59	NA
	SNPlex technology	2	965	960	0.954	0.785-1.160	0.636	F	0.145	0.704	0.0	NA
	Other methods	1	98	97	0.654	0.315-1.360	0.256	F	0.0	1.0	0.0	NA
	**Source of controls**											
	Population-based	3	711	910	0.801	0.639-1.004	0.054	F	3.950	0.139	49.37	0.197
	Hospital-based	2	797	791	0.942	0.757-1.172	0.593	F	1.048	0.306	4.614	NA
	**HWE in controls**											
	Equilibrium	5	1508	1701	0.871	0.745-1.019	0.085	F	6.021	0.198	33.56	0.070
	**Quality score**											
	High quality	3	1068	1302	0.806	0.567-1.146	0.231	R	5.399	0.067	62.96	0.267
	Low quality	2	440	399	0.834	0.619-1.123	0.231	F	0.506	0.477	0.0	NA

OR: odds ratio, CI: confidence interval, F: fixed model, R: random model, NA: not appreciable

Supplementary Table S21. Meta-analysis of the association between GEMIN3 (T > C; rs197412) variant and cancer risk.

Comparison	Subgroups	No. of studies	Sample size		Test of association				Test of heterogeneity			Publication bias
			Cancer	Control	OR	95% CI	*P*-value	Model	Q test	*P*-value	I^2 (%)	*P*-value (Egger's)
**Allelic model (C allele *versus* T allele)**	**Overall**	9	7122	10602	1.008	0.929-1.094	0.848	R	17.89	0.022	55.29	0.351
	**Geographical region**											
	Asian	5	4536	6840	**0.919**	**0.847-0.997**	**0.042**	F	6.520	0.164	38.65	0.937
	European	1	202	688	1.116	0.809-1.538	0.504	F	0.0	1.0	0.0	NA
	American	3	2384	3074	**1.125**	**1.006-1.259**	**0.038**	F	2.573	0.276	22.28	**0.025**
	**Cancer type**											
	Oral cancer	2	1496	1788	0.809	0.698-0.937	0.005	F	0.054	0.817	0.0	NA
	Bladder Cancer	1	1470	1460	1.040	0.898-1.206	0.600	F	0.0	1.0	0.0	NA
	Breast cancer	1	1706	1772	0.909	0.790-1.046	0.182	F	0.0	1.0	0.0	NA
	CLL	1	202	688	1.116	0.809-1.538	0.504	F	0.0	1.0	0.0	NA
	Head/neck carcinoma	1	1150	3100	1.015	0.880-1.171	0.838	F	0.0	1.0	0.0	NA
	Lung cancer	1	184	180	1.217	0.797-1.858	0.362	F	0.0	1.0	0.0	NA
	NHL	1	360	1058	1.255	0.986-1.597	0.064	F	0.0	1.0	0.0	NA
	Renal cell carcinoma	1	554	556	1.248	0.978-1.592	0.075	F	0.0	1.0	0.0	NA
	**Genotyping method**											
	TaqMan PCR	4	3404	4248	**0.881**	**0.799-0.970**	**0.010**	F	3.621	0.305	17.14	0.746
	SNPlex technology	2	2024	2016	1.092	0.963-1.239	0.170	F	1.568	0.210	36.23	NA
	Other methods	3	1694	4338	1.083	0.963-1.219	0.184	F	2.527	0.283	20.86	0.422
	**Source of controls**											
	Population-based	4	3612	6116	1.005	0.920-1.099	0.907	F	5.429	0.143	44.73	0.248
	Hospital-based	5	3510	4486	0.984	0.826-1.173	0.860	R	12.25	0.016	67.34	0.769
	**HWE in controls**											
	Equilibrium	8	5652	9142	1.003	0.889-1.132	0.960	R	17.37	0.015	59.71	0.293
	Disequilibrium	1	1470	1460	1.040	0.898-1.206	0.600	F	0.0	1.0	0.0	NA
	**Quality score**											
	High quality	7	6342	9528	1.024	0.921-1.137	0.663	R	12.93	0.044	53.61	0.259
	Low quality	2	780	1074	0.945	0.625-1.428	0.787	R	3.090	0.079	67.64	NA
**Recessive model (CC versus TT+TC)**	**Overall**	9	3561	5301	1.069	0.912-1.254	0.409	R	15.82	0.045	49.43	0.783
	**Geographical region**											
	Asian	5	2268	3420	0.869	0.731-1.033	0.112	F	7.598	0.107	47.36	0.549
	European	1	101	344	1.034	0.562-1.900	0.916	F	0.0	1.0	0.0	NA
	American	3	1192	1537	**1.273**	**1.027-1.577**	**0.027**	F	0.840	0.657	0.0	0.182
	**Cancer type**											
	Oral cancer	2	748	894	**0.607**	**0.438-0.841**	**0.003**	F	0.004	0.948	0.0	NA
	Bladder Cancer	1	735	730	1.164	0.874-1.551	0.299	F	0.0	1.0	0.0	NA
	Breast cancer	1	853	886	0.892	0.665-1.197	0.447	F	0.0	1.0	0.0	NA
	CLL	1	101	344	1.034	0.562-1.900	0.916	F	0.0	1.0	0.0	NA
	Head/neck carcinoma	1	575	1550	1.114	0.827-1.501	0.476	F	0.0	1.0	0.0	NA
	Lung cancer	1	92	90	1.086	0.437-2.700	0.858	F	0.0	1.0	0.0	NA
	NHL	1	180	529	1.425	0.933-2.175	0.101	F	0.0	1.0	0.0	NA
	Renal cell carcinoma	1	277	278	1.424	0.866-2.341	0.164	F	0.0	1.0	0.0	NA
	**Genotyping method**											
	TaqMan PCR	4	1702	2124	**0.778**	**0.634-0.956**	**0.017**	F	3.904	0.272	23.16	0.730
	SNPlex technology	2	1012	1008	1.224	0.955-1.569	0.110	F	0.473	0.492	0.0	NA
	Other methods	3	847	2169	1.200	0.949-1.519	0.128	F	0.916	0.633	0.0	0.907
	**Source of controls**											
	Population-based	4	1806	3058	1.049	0.873-1.261	0.609	F	2.777	0.427	0.0	0.510
	Hospital-based	5	1755	2243	0.923	0.641-1.329	0.666	R	12.73	0.013	68.58	0.620
	**HWE in controls**											
	Equilibrium	8	2826	4571	0.971	0.773-1.220	0.801	R	14.65	0.041	52.21	0.984
	Disequilibrium	1	735	730	1.164	0.874-1.551	0.299	F	0.0	1.0	0.0	NA
	**Quality score**											
	High quality	7	3171	4764	1.051	0.860-1.284	0.627	R	11.60	0.072	48.27	0.948
	Low quality	2	390	537	0.699	0.453-1.079	0.106	F	1.165	0.280	14.16	NA
**Dominant model (TC+CC versus TT)**	**Overall**	9	3561	5301	0.978	0.895-1.070	0.629	F	11.68	0.166	31.52	**0.062**
	**Geographical region**											
	Asian	5	2268	3420	0.909	0.815-1.015	0.089	F	3.970	0.410	0.0	0.486
	European	1	101	344	1.224	0.772-1.941	0.389	F	0.0	1.0	0.0	NA
	American	3	1192	1537	1.119	0.950-1.318	0.178	F	2.503	0.286	20.10	**0.081**
	**Cancer type**											
	Oral cancer	2	748	894	0.828	0.680-1.008	0.059	F	0.108	0.742	0.0	NA
	Bladder Cancer	1	735	730	0.998	0.804-1.239	0.986	F	0.0	1.0	0.0	NA
	Breast cancer	1	853	886	0.880	0.728-1.064	0.188	F	0.0	1.0	0.0	NA
	CLL	1	101	344	1.224	0.772-1.941	0.389	F	0.0	1.0	0.0	NA
	Head/neck carcinoma	1	575	1550	0.983	0.810-1.192	0.860	F	0.0	1.0	0.0	NA

	Lung cancer	1	92	90	1.455	0.787-2.687	0.232	F	0.0	1.0	0.0	NA
	NHL	1	180	529	1.297	0.900-1.868	0.163	F	0.0	1.0	0.0	NA
	Renal cell carcinoma	1	277	278	1.309	0.929-1.846	0.124	F	0.0	1.0	0.0	NA
	**Genotyping method**											
	TaqMan PCR	4	1702	2124	0.880	0.772-1.003	0.055	F	2.453	0.484	0.0	0.413
	SNPlex technology	2	1012	1008	1.078	0.898-1.295	0.420	F	1.716	0.190	41.73	NA
	Other methods	3	847	2169	1.069	0.907-1.260	0.424	F	2.774	0.250	27.89	0.195
	**Source of controls**											
	Population-based	4	1806	3058	0.989	0.876-1.117	0.856	F	4.837	0.184	37.98	0.145
	Hospital-based	5	1755	2243	0.966	0.847-1.102	0.606	F	6.779	0.148	40.99	0.315
	**HWE in controls**											
	Equilibrium	8	2826	4571	0.974	0.883-1.075	0.602	F	11.64	0.113	39.87	**0.071**
	Disequilibrium	1	735	730	0.998	0.804-1.239	0.986	F	0.0	1.0	0.0	NA
	**Quality score**											
	High quality	7	3171	4764	0.990	0.900-1.088	0.831	F	8.181	0.225	26.66	**0.052**
	Low quality	2	390	537	1.012	0.569-1.799	0.969	R	2.988	0.084	66.53	NA
**Homozygote model (CC *versus* TT)**	**Overall**	9	1900	2820	1.085	0.910-1.294	0.364	R	19.15	0.014	58.23	0.749
	**Geographical region**											
	Asian	5	1249	1857	0.810	0.609-1.077	0.147	R	8.225	0.084	51.37	0.775
	European	1	52	192	1.166	0.597-2.275	0.653	F	0.0	1.0	0.0	NA
	American	3	599	771	**1.319**	**1.039-1.673**	**0.023**	F	1.924	0.382	0.0	0.100
	**Cancer type**											
	Oral cancer	2	416	498	**0.576**	**0.409-0.812**	**0.002**	F	0.002	0.967	0.0	NA
	Bladder Cancer	1	367	350	1.136	0.827-1.561	0.430	F	0.0	1.0	0.0	NA
	Breast cancer	1	474	475	0.841	0.616-1.148	0.275	F	0.0	1.0	0.0	NA
	CLL	1	52	192	1.166	0.597-2.275	0.653	F	0.0	1.0	0.0	NA
	Head/neck carcinoma	1	320	839	1.090	0.795-1.494	0.593	F	0.0	1.0	0.0	NA
	Lung cancer	1	39	45	1.375	0.511-3.701	0.528	F	0.0	1.0	0.0	NA
	NHL	1	93	275	1.587	0.978-2.576	0.062	F	0.0	1.0	0.0	NA
	Renal cell carcinoma	1	139	146	1.606	0.939-2.748	0.084	F	0.0	1.0	0.0	NA
	**Genotyping method**											
	TaqMan PCR	4	942	1165	**0.747**	**0.601-0.929**	**0.009**	F	4.460	0.216	32.74	0.956
	SNPlex technology	2	506	496	1.243	0.946-1.634	0.119	F	1.182	0.277	15.37	NA
	Other methods	3	452	1159	1.229	0.952-1.586	0.114	F	1.678	0.432	0.0	0.601
	**Source of controls**											
	Population-based	4	985	1652	1.042	0.856-1.267	0.684	F	4.507	0.212	33.44	0.339
	Hospital-based	5	915	1168	0.935	0.614-1.422	0.753	R	14.27	0.006	71.96	0.908
	**HWE in controls**											
	Equilibrium	8	1533	2470	0.991	0.753-1.304	0.950	R	18.35	0.010	61.86	0.627
	Disequilibrium	1	367	350	1.136	0.827-1.561	0.430	F	0.0	1.0	0.0	NA
	**Quality score**											
	High quality	7	1692	2526	1.061	0.835-1.347	0.629	R	14.01	0.030	57.17	0.566
	Low quality	2	208	294	0.690	0.436-1.090	0.112	F	2.372	0.123	57.85	NA
**Heterozygote model (TC *versus* TT)**	**Overall**	9	3110	4625	0.979	0.891-1.075	0.655	F	6.875	0.550	0.0	**0.007**
	**Geographical region**											
	Asian	5	2032	3017	0.930	0.829-1.044	0.220	F	2.476	0.649	0.0	0.177
	European	1	85	291	1.245	0.764-2.027	0.379	F	0.0	1.0	0.0	NA
	American	3	993	1317	1.063	0.895-1.263	0.484	F	1.830	0.401	0.0	0.137
	**Cancer type**											
	Oral cancer	2	686	778	0.901	0.733-1.108	0.325	F	0.129	0.720	0.0	NA
	Bladder Cancer	1	617	627	0.961	0.766-1.205	0.729	F	0.0	1.0	0.0	NA
	Breast cancer	1	759	778	0.891	0.729-1.088	0.257	F	0.0	1.0	0.0	NA
	CLL	1	85	291	1.245	0.764-2.027	0.379	F	0.0	1.0	0.0	NA
	Head/neck carcinoma	1	506	1381	0.957	0.781-1.174	0.675	F	0.0	1.0	0.0	NA
	Lung cancer	1	81	80	1.472	0.779-2.782	0.233	F	0.0	1.0	0.0	NA
	NHL	1	141	443	1.199	0.813-1.768	0.360	F	0.0	1.0	0.0	NA
	Renal cell carcinoma	1	235	247	1.239	0.864-1.777	0.243	F	0.0	1.0	0.0	NA
	**Genotyping method**											
	TaqMan PCR	4	1530	1847	0.920	0.801-1.056	0.234	F	1.742	0.628	0.0	0.210
	SNPlex technology	2	852	874	1.033	0.852-1.251	0.742	F	1.375	0.241	27.25	NA
	Other methods	3	728	1904	1.034	0.869-1.230	0.706	F	2.291	0.318	12.71	**0.070**
	**Source of controls**											
	Population-based	4	1585	2697	0.978	0.860-1.111	0.731	F	3.480	0.323	13.80	**0.078**
	Hospital-based	5	1525	1928	0.980	0.853-1.126	0.775	F	3.394	0.494	0.0	0.121
	**HWE in controls**											
	Equilibrium	8	2493	3998	0.983	0.886-1.090	0.739	F	6.843	0.445	0.0	**0.013**
	Disequilibrium	1	617	627	0.961	0.766-1.205	0.729	F	0.0	1.0	0.0	NA
	**Quality score**											

	High quality	7	2756	4156	0.982	0.888-1.085	0.716	F	4.669	0.587	0.0	**0.006**
	Low quality	2	354	469	0.957	0.724-1.264	0.757	F	2.177	0.140	54.07	NANA

OR: odds ratio, CI: confidence interval, F: fixed model, R: random model, NA: not appreciable

Supplementary Table S22. Meta-analysis of the association between GEMIN3 (C > A; rs197414) variant and cancer risk.

Comparison	Subgroups	No. of studies	Sample size		Test of association				Test of heterogeneity			Publication bias
			Cancer	Control	OR	95% CI	*P*-value	Model	Q test	*P*-value	I^2 (%)	*P*-value (Egger's)
**Allelic model (A allele *versus* C allele)**	**Overall**	5	3132	3518	1.090	0.939-1.264	0.257	F	7.453	0.114	46.33	0.886
	**Geographical region**											
	Asian	1	200	198	0.196	0.009-4.109	0.294	F	0.0	1.0	0.0	NA
	European	2	896	1280	1.195	0.613-2.332	0.601	R	6.064	0.014	83.51	NA
	American	2	2036	2040	1.104	0.919-1.326	0.292	F	0.140	0.708	0.0	NA
	**Cancer type**											
	Prostate cancer	1	706	638	0.867	0.639-1.177	0.361	F	0.0	1.0	0.0	NA
	CLL	1	190	642	**1.718**	**1.095-2.694**	**0.018**	F	0.0	1.0	0.0	NA
	Lung cancer	1	200	198	0.196	0.009-4.109	0.294	F	0.0	1.0	0.0	NA
	Bladder cancer	1	1480	1484	1.082	0.876-1.337	0.465	F	0.0	1.0	0.0	NA
	Renal cell carcinoma	1	556	556	1.174	0.810-1.700	0.396	F	0.0	1.0	0.0	NA
	**Genotyping method**											
	SNPlex technology	2	2036	2040	1.104	0.919-1.326	0.292	F	0.140	0.708	0.0	NA
	Other methods	3	1096	1478	1.106	0.573-2.134	0.764	R	7.258	0.027	72.45	0.920
	**Source of controls**											
	Population-based	2	746	1198	**1.369**	**1.028-1.822**	**0.031**	F	1.639	0.200	38.98	NA
	Hospital-based	3	2386	2320	1.002	0.842-1.192	0.984	F	2.469	0.291	19.00	0.361
	**HWE in controls**											
	Equilibrium	4	2932	3320	1.094	0.943-1.269	0.235	F	6.229	0.101	51.84	0.469
	Disequilibrium	1	200	198	0.196	0.009-4.109	0.294	F	0.0	1.0	0.0	NA
	**Quality score**											
	High quality	3	2226	2682	1.176	0.992-1.394	0.062	F	3.319	0.190	39.74	0.337
	Low quality	2	906	836	0.854	0.630-1.158	0.311	F	0.908	0.341	0.0	NA
**Recessive model (AA versus CC+CA)**	**Overall**	5	1566	1759	**1.729**	**1.055-2.832**	**0.030**	F	6.263	0.180	36.13	0.625
	**Geographical region**											
	Asian	1	100	99	0.327	0.013-8.117	0.495	F	0.0	1.0	0.0	NA
	European	2	448	640	1.436	0.415-4.960	0.568	R	2.805	0.094	64.35	NA
	American	2	1018	1020	**2.571**	**1.268-5.214**	**0.009**	F	0.443	0.506	0.0	NA
	**Cancer type**											
	Prostate cancer	1	353	319	0.816	0.342-1.949	0.648	F	0.0	1.0	0.0	NA
	CLL	1	95	321	2.917	0.870-9.778	0.083	F	0.0	1.0	0.0	NA
	Lung cancer	1	100	99	0.327	0.013-8.117	0.495	F	0.0	1.0	0.0	NA
	Bladder cancer	1	740	742	**2.943**	**1.308-6.623**	**0.009**	F	0.0	1.0	0.0	NA
	Renal cell carcinoma	1	278	278	1.679	0.397-7.094	0.481	F	0.0	1.0	0.0	NA
	**Genotyping method**											
	SNPlex technology	2	1018	1020	**2.571**	**1.268-5.214**	**0.009**	F	0.443	0.506	0.0	NA
	Other methods	3	548	739	1.184	0.594-2.360	0.631	F	3.452	0.178	42.07	0.922
	**Source of controls**											
	Population-based	2	373	599	2.321	0.919-5.863	0.075	F	0.331	0.565	0.0	NA
	Hospital-based	3	1193	1160	1.332	0.427-4.156	0.621	R	5.389	0.068	62.89	0.685
	**HWE in controls**											
	Equilibrium	4	1466	1660	**1.800**	**1.092-2.966**	**0.021**	F	5.205	0.157	42.36	0.906
	Disequilibrium	1	100	99	0.327	0.013-8.117	0.495	F	0.0	1.0	0.0	NA
	**Quality score**											
	High quality	3	1113	1341	**2.655**	**1.442-4.888**	**0.002**	F	0.474	0.789	0.0	0.441
	Low quality	2	453	418	0.767	0.331-1.776	0.535	F	0.291	0.590	0.0	NA
**Dominant model (CA+AA versus CC)**	**Overall**	5	1566	1759	1.036	0.877-1.223	0.679	F	5.378	0.251	25.63	0.879
	**Geographical region**											
	Asian	1	100	99	0.327	0.013-8.117	0.495	F	0.0	1.0	0.0	NA
	European	2	448	640	1.167	0.607-2.242	0.644	R	4.411	0.036	77.33	NA
	American	2	1018	1020	1.030	0.840-1.263	0.775	F	0.450	0.502	0.0	NA
	**Cancer type**											
	Prostate cancer	1	353	319	0.859	0.607-1.216	0.392	F	0.0	1.0	0.0	NA
	CLL	1	95	321	1.678	0.999-2.820	0.051	F	0.0	1.0	0.0	NA
	Lung cancer	1	100	99	0.327	0.013-8.117	0.495	F	0.0	1.0	0.0	NA
	Bladder cancer	1	740	742	0.989	0.781-1.253	0.928	F	0.0	1.0	0.0	NA
	Renal cell carcinoma	1	278	278	1.162	0.774-1.743	0.469	F	0.0	1.0	0.0	NA
	**Genotyping method**											
	SNPlex technology	2	1018	1020	1.030	0.840-1.263	0.775	F	0.450	0.502	0.0	NA
	Other methods	3	548	739	1.114	0.612-2.027	0.724	R	4.920	0.085	59.35	0.986

	Source of controls											
	Population-based	2	373	599	1.336	0.970-1.839	0.076	F	1.197	0.274	16.47	NA
	Hospital-based	3	1193	1160	0.942	0.776-1.145	0.551	F	0.850	0.654	0.0	0.325
	HWE in controls											
	Equilibrium	4	1466	1660	1.039	0.880-1.227	0.653	F	4.881	0.181	38.54	0.309
	Disequilibrium	1	100	99	0.327	0.013-8.117	0.495	F	0.0	1.0	0.0	NA
	Quality score											
	High quality	3	1113	1341	1.100	0.909-1.330	0.327	F	3.390	0.184	41.01	0.207
	Low quality	2	453	418	0.850	0.602-1.200	0.355	F	0.344	0.557	0.0	NA
Homozygote model (AA *versus* CC)	Overall	5	1251	1397	**1.727**	**1.052-2.835**	**0.031**	F	6.596	0.159	39.36	0.668
	Geographical region											
	Asian	1	100	99	0.327	0.013-8.117	0.495	F	0.0	1.0	0.0	NA
	European	2	350	507	1.487	0.380-5.811	0.568	R	3.341	0.068	70.07	NA
	American	2	801	791	**2.541**	**1.251-5.160**	**0.010**	F	0.369	0.544	0.0	NA
	Cancer type											
	Prostate cancer	1	278	244	0.790	0.330-1.894	0.598	F	0.0	1.0	0.0	NA
	CLL	1	72	263	3.197	0.947-10.794	0.061	F	0.0	1.0	0.0	NA
	Lung cancer	1	100	99	0.327	0.013-8.117	0.495	F	0.0	1.0	0.0	NA
	Bladder cancer	1	581	566	**2.875**	**1.275-6.482**	**0.011**	F	0.0	1.0	0.0	NA
	Renal cell carcinoma	1	220	225	1.721	0.406-7.290	0.461	F	0.0	1.0	0.0	NA
	Genotyping method											
	SNPlex technology	2	801	791	**2.541**	**1.251-5.160**	**0.010**	F	0.369	0.544	0.0	NA
	Other methods	3	450	606	1.194	0.597-2.388	0.617	F	3.996	0.136	49.95	0.942
	Source of controls											
	Population-based	2	292	488	2.472	0.975-6.267	0.057	F	0.413	0.520	0.0	NA
	Hospital-based	3	959	909	1.299	0.415-4.066	0.653	R	5.388	0.068	62.88	0.688
	HWE in controls											
	Equilibrium	4	1151	1298	**1.799**	**1.090-2.970**	**0.022**	F	5.539	0.136	45.84	0.855
	Disequilibrium	1	100	99	0.327	0.013-8.117	0.495	F	0.0	1.0	0.0	NA
	Quality score											
	High quality	3	873	1054	**2.693**	**1.460-4.967**	**0.002**	F	0.471	0.790	0.0	0.588
	Low quality	2	378	343	0.744	0.320-1.729	0.491	F	0.270	0.603	0.0	NA
Heterozygote model (CA *versus* CC)	Overall	4	1423	1632	0.980	0.824-1.165	0.819	F	3.717	0.294	19.29	0.166
	Geographical region											
	European	2	433	623	1.105	0.642-1.901	0.719	R	2.739	0.098	63.50	NA
	American	2	990	1009	0.957	0.775-1.181	0.680	F	0.823	0.364	0.0	NA
	Cancer type											
	Prostate cancer	1	343	308	0.869	0.604-1.252	0.452	F	0.0	1.0	0.0	NA
	CLL	1	90	315	1.521	0.875-2.644	0.137	F	0.0	1.0	0.0	NA
	Bladder cancer	1	717	734	0.903	0.707-1.154	0.415	F	0.0	1.0	0.0	NA
	Renal cell carcinoma	1	273	275	1.130	0.745-1.715	0.566	F	0.0	1.0	0.0	NA
	Genotyping method											
	SNPlex technology	2	990	1009	0.957	0.775-1.181	0.680	F	0.823	0.364	0.0	NA
	Other methods	2	433	623	1.105	0.642-1.901	0.719	R	2.739	0.098	63.50	NA
	Source of controls											
	Population-based	2	363	590	1.259	0.902-1.756	0.176	F	0.708	0.400	0.0	NA
	Hospital-based	2	1060	1042	0.893	0.729-1.094	0.274	F	0.029	0.864	0.0	NA
	HWE in controls											
	Equilibrium	4	1423	1632	0.980	0.824-1.165	0.819	F	3.717	0.294	19.29	0.166
	Quality score											
	High quality	3	1080	1324	1.015	0.833-1.236	0.885	F	3.184	0.204	37.19	**0.090**
	Low quality	1	343	308	0.869	0.604-1.252	0.452	F	0.0	1.0	0.0	NA

OR: odds ratio, CI: confidence interval, F: fixed model, R: random model, NA: not appreciable

Supplementary Table S23. Meta-analysis of the association between GEMIN4 (G > A; rs7813) variant and cancer risk.

Comparison	Subgroups	No. of studies	Sample size		Test of association				Test of heterogeneity			Publication bias
			Cancer	Control	OR	95% CI	*P*-value	Model	Q test	*P*-value	I^2 (%)	*P*-value (Egger's)
**Allelic model (A allele *versus* G allele)**	**Overall**	14	11294	11236	0.969	0.891-1.053	0.455	R	91.23	< 0.001	85.75	**0.051**
	**Geographical region**											
	Asian	9	7506	6824	1.243	0.980-1.578	0.073	R	80.64	< 0.001	90.08	0.103
	European	1	710	638	1.023	0.823-1.270	0.840	F	0.0	1.0	0.0	NA
	American	4	3078	3774	0.918	0.833-1.013	0.089	F	2.985	0.394	0.0	0.823
	**Cancer type**											
	Prostate cancer	3	2006	1266	1.768	0.829-3.771	0.140	R	41.45	< 0.001	95.17	0.209
	Lung cancer	2	1142	988	**0.794**	**0.663-0.952**	**0.013**	F	2.647	0.104	62.23	NA
	Esophageal cancer	2	1454	1544	0.979	0.844-1.136	0.781	F	0.748	0.387	0.0	NA
	Breast cancer	2	2850	2902	0.974	0.874-1.087	0.643	F	2.376	0.123	57.92	NA
	Renal cell carcinoma	2	754	1006	1.144	0.567-2.311	0.707	R	11.16	0.001	91.04	NA
	Gastric cancer	1	1256	1004	**1.265**	**1.059-1.512**	**0.010**	F	0.0	1.0	0.0	NA
	NHL	1	360	1054	1.088	0.853-1.388	0.498	F	0.0	1.0	0.0	NA
	Bladder cancer	1	1472	1472	0.908	0.785-1.050	0.195	F	0.0	1.0	0.0	NA
	**Genotyping method**											
	TaqMan PCR	5	4706	4780	0.996	0.821-1.210	0.971	R	18.75	0.001	78.66	0.492
	HRM method	3	2552	1632	1.890	0.990-3.607	0.054	R	33.51	< 0.001	94.03	0.395
	SNPlex technology	3	2718	2720	**0.889**	**0.799-0.989**	**0.031**	F	0.772	0.680	0.0	0.540
	Other methods	3	1318	2104	1.065	0.919-1.235	0.402	F	0.074	0.964	0.0	0.594
	**Source of controls**											
	Population-based	6	4654	4406	1.322	0.949-1.841	0.099	R	61.91	< 0.001	91.92	0.144
	Hospital-based	8	6640	6830	0.992	0.864-1.138	0.907	R	23.43	0.001	70.12	0.430
	**HWE in controls**											
	Equilibrium	13	10598	11096	1.014	0.916-1.122	0.794	R	35.84	< 0.001	66.52	0.266
	Disequilibrium	1	696	140	**4.281**	**2.927-6.262**	**< 0.001**	F	0.0	1.0	0.0	NA
	**Quality score**											
	High quality	11	9692	10260	1.012	0.902-1.135	0.846	R	35.59	< 0.001	71.90	0.291
	Low quality	3	1602	976	1.674	0.689-4.063	0.255	R	42.66	< 0.001	95.31	0.603
**Recessive model (AA versus GG+GA)**	**Overall**	14	5647	5618	1.005	0.855-1.182	0.949	R	64.35	< 0.001	79.80	0.022
	**Geographical region**											
	Asian	9	3753	3412	**1.488**	**1.030-2.152**	**0.034**	R	48.54	< 0.001	83.52	0.135
	European	1	355	319	1.121	0.742-1.692	0.588	F	0.0	1.0	0.0	NA
	American	4	1539	1887	0.860	0.723-1.023	0.089	F	3.785	0.286	20.73	0.237
	**Cancer type**											
	Prostate cancer	3	1003	633	2.395	0.803-7.143	0.117	R	33.12	< 0.001	93.96	0.201
	Lung cancer	2	571	494	0.911	0.628-1.322	0.625	F	0.0	0.988	0.0	NA
	Esophageal cancer	2	727	772	0.957	0.710-1.288	0.770	F	0.767	0.381	0.0	NA
	Breast cancer	2	1425	1451	1.059	0.701-1.599	0.785	R	3.274	0.070	69.46	NA
	Renal cell carcinoma	2	377	503	1.382	0.489-3.904	0.542	R	7.051	0.008	85.82	NA
	Gastric cancer	1	628	502	**1.562**	**1.039-2.348**	**0.032**	F	0.0	1.0	0.0	NA
	NHL	1	180	527	1.255	0.812-1.941	0.307	F	0.0	1.0	0.0	NA
	Bladder cancer	1	736	736	**0.760**	**0.588-0.983**	**0.037**	F	0.0	1.0	0.0	NA
	**Genotyping method**											
	TaqMan PCR	5	2353	2390	1.146	0.867-1.515	0.339	R	9.606	0.048	58.36	0.303
	HRM method	3	1276	816	2.667	0.948-7.504	0.063	R	29.87	< 0.001	93.30	**0.052**
	SNPlex technology	3	1359	1360	**0.800**	**0.662-0.968**	**0.021**	F	0.347	0.841	0.0	0.203
	Other methods	3	659	1052	1.159	0.857-1.568	0.338	F	0.418	0.812	0.0	0.233
	**Source of controls**											
	Population-based	6	2327	2203	1.732	0.996-3.012	0.052	R	42.57	< 0.001	88.25	0.111
	Hospital-based	8	3320	3415	1.001	0.831-1.206	0.993	R	12.92	0.074	45.82	0.331
	**HWE in controls**											
	Equilibrium	13	5299	5548	1.071	0.916-1.252	0.390	R	23.44	0.024	48.81	0.128
	Disequilibrium	1	348	70	**11.65**	**5.597-24.25**	**< 0.001**	F	0.0	1.0	0.0	NA
	**Quality score**											
	High quality	11	4846	5130	1.076	0.902-1.284	0.413	R	23.23	0.010	56.95	**0.049**
	Low quality	3	801	488	2.279	0.497-10.46	0.289	R	32.31	< 0.001	93.81	0.699
**Dominant model (GA+AA versus GG)**	**Overall**	14	5647	5618	0.979	0.868-1.104	0.727	R	49.62	< 0.001	73.80	**0.055**
	**Geographical region**											
	Asian	9	3753	3412	1.193	0.915-1.555	0.192	R	45.51	< 0.001	82.42	**0.066**
	European	1	355	319	0.979	0.708-1.355	0.900	F	0.0	1.0	0.0	NA
	American	4	1539	1887	0.920	0.794-1.067	0.271	F	2.998	0.392	0.0	0.484
	**Cancer type**											
	Prostate cancer	3	1003	633	2.328	0.765-7.081	0.137	R	17.22	< 0.001	88.38	0.163
	Lung cancer	2	571	494	0.815	0.416-1.595	0.550	R	4.722	0.030	78.82	NA
	Esophageal cancer	2	727	772	0.980	0.794-1.210	0.853	F	0.358	0.550	0.0	NA
	Breast cancer	2	1425	1451	0.928	0.801-1.075	0.318	F	0.953	0.329	0.0	NA
	Renal cell carcinoma	2	377	503	1.051	0.456-2.425	0.906	R	7.745	0.005	87.09	NA
	Gastric cancer	1	628	502	**1.298**	**1.025-1.644**	**0.030**	F	0.0	1.0	0.0	NA
	NHL	1	180	527	1.032	0.727-1.463	0.862	F	0.0	1.0	0.0	NA

	Bladder cancer	1	736	736	0.981	0.785-1.225	0.865	F	0.0	1.0	0.0	NA
	**Genotyping method**											
	TaqMan PCR	5	2353	2390	0.919	0.719-1.175	0.501	R	15.66	0.004	74.46	0.607
	HRM method	3	1276	816	**2.512**	**1.001-6.308**	**0.050**	R	12.70	0.002	84.25	0.222
	SNPlex technology	3	1359	1360	0.898	0.763-1.057	0.195	F	2.500	0.287	19.99	0.332
	Other methods	3	659	1052	1.055	0.861-1.293	0.603	F	0.247	0.884	0.0	0.254
	**Source of controls**											
	Population-based	6	2327	2203	1.166	0.856-1.589	0.331	R	23.92	< 0.001	79.10	0.143
	Hospital-based	8	3320	3415	0.992	0.803-1.225	0.939	R	25.02	0.001	72.03	0.363
	**HWE in controls**											
	Equilibrium	13	5299	5548	0.989	0.858-1.139	0.873	R	33.10	0.001	63.75	0.282
	Disequilibrium	1	348	70	**6.302**	**2.562-15.50**	**< 0.001**	F	0.0	1.0	0.0	NA
	**Quality score**											
	High quality	11	4846	5130	0.983	0.837-1.153	0.830	R	32.50	< 0.001	69.23	0.374
	Low quality	3	801	488	1.771	0.740-4.235	0.199	F	0.0	1.0	0.0	NA
**Homozygote model (AA *versus* GG)**	**Overall**	14	3142	3005	0.967	0.810-1.155	0.714	R	66.87	< 0.001	80.56	**0.012**
	**Geographical region**											
	Asian	9	2189	1876	**1.646**	**1.043-2.595**	**0.032**	R	54.38	< 0.001	85.29	**0.053***
	European	1	174	150	1.085	0.683-1.723	0.730	F	0.0	1.0	0.0	NA
	American	4	779	979	0.839	0.688-1.023	0.083	F	3.340	0.342	10.18	0.679
	**Cancer type**											
	Prostate cancer	3	606	333	3.940	0.753-20.63	0.105	R	29.04	< 0.001	93.11	0.203
	Lung cancer	2	349	261	0.748	0.505-1.110	0.149	F	0.556	0.456	0.0	NA
	Esophageal cancer	2	374	398	0.951	0.685-1.320	0.764	F	0.883	0.347	0.0	NA
	Breast cancer	2	815	793	1.004	0.639-1.575	0.987	R	3.474	0.062	71.21	NA
	Renal cell carcinoma	2	207	271	1.344	0.334-5.414	0.677	R	10.56	0.001	90.53	NA
	Gastric cancer	1	334	280	**1.728**	**1.128-2.647**	**0.012**	F	0.0	1.0	0.0	NA
	NHL	1	102	285	1.229	0.760-1.989	0.401	F	0.0	1.0	0.0	NA
	Bladder cancer	1	355	384	0.792	0.589-1.065	0.122	F	0.0	1.0	0.0	NA
	**Genotyping method**											
	TaqMan PCR	5	1344	1281	1.089	0.756-1.569	0.647	R	14.24	0.007	71.90	0.389
	HRM method	3	766	463	4.535	1.124-18.30	0.034	R	21.29	< 0.001	90.60	0.320
	SNPlex technology	3	677	694	**0.776**	**0.624-0.965**	**0.022**	F	0.423	0.809	0.0	0.680
	Other methods	3	355	567	1.165	0.842-1.612	0.357	F	0.128	0.938	0.0	0.355
	**Source of controls**											
	Population-based	6	1306	1145	1.786	0.960-3.322	0.067	R	42.42	< 0.001	88.21	**0.091**
	Hospital-based	8	1836	1860	1.023	0.780-1.342	0.867	R	19.28	0.007	63.70	0.173
	**HWE in controls**											
	Equilibrium	13	2912	2985	1.080	0.874-1.333	0.476	R	32.41	0.001	62.97	0.105
	Disequilibrium	1	230	20	**26.89**	**9.084-79.59**	**< 0.001**	F	0.0	1.0	0.0	NA
	**Quality score**											
	High quality	11	2685	2776	1.089	0.856-1.383	0.488	R	32.33	< 0.001	69.07	**0.077**
	Low quality	3	457	229	2.959	0.498-17.57	0.233	R	29.58	< 0.001	93.24	0.503
**Heterozygote model (GA *versus* GG)**	**Overall**	14	4527	4714	0.981	0.867-1.110	0.757	R	31.10	0.003	58.20	0.131
	**Geographical region**											
	Asian	9	2972	2934	1.056	0.847-1.316	0.631	R	27.80	0.001	71.22	0.110
	European	1	295	270	0.949	0.675-1.333	0.762	F	0.0	1.0	0.0	NA
	American	4	1260	1510	0.953	0.814-1.115	0.546	F	3.271	0.352	8.293	0.217
	**Cancer type**											
	Prostate cancer	3	531	431	1.623	0.781-3.373	0.195	R	6.950	0.031	71.22	0.020
	Lung cancer	2	506	433	0.816	0.384-1.736	0.598	R	5.282	0.022	81.07	NA
	Esophageal cancer	2	628	665	0.985	0.790-1.229	0.896	F	0.111	0.739	0.0	NA
	Breast cancer	2	1251	1284	0.904	0.774-1.057	0.206	F	0.202	0.653	0.0	NA
	Renal cell carcinoma	2	305	422	0.955	0.503-1.814	0.889	R	3.970	0.046	74.81	NA
	Gastric cancer	1	555	463	1.223	0.955-1.566	0.110	F	0.0	1.0	0.0	NA
	NHL	1	145	442	0.962	0.660-1.402	0.841	F	0.0	1.0	0.0	NA
	Bladder cancer	1	606	574	1.068	0.844-1.351	0.584	F	0.0	1.0	0.0	NA
	**Genotyping method**											
	TaqMan PCR	5	2045	2104	0.868	0.697-1.082	0.209	R	11.14	0.025	64.08	0.708
	HRM method	3	791	624	**1.346**	**1.071-1.693**	**0.011**	F	4.181	0.124	52.17	**0.006***
	SNPlex technology	3	1115	1068	0.951	0.800-1.130	0.568	F	3.268	0.195	38.80	0.271
	Other methods	3	576	918	1.027	0.829-1.273	0.807	F	0.569	0.753	0.0	0.452
	**Source of controls**											
	Population-based	6	1840	1948	0.963	0.844-1.098	0.570	F	8.971	0.110	44.27	0.202
	Hospital-based	8	2687	2766	0.986	0.799-1.218	0.899	R	22.13	0.002	68.36	0.437
	**HWE in controls**											
	Equilibrium	13	4399	4653	0.964	0.843-1.103	0.596	R	26.56	0.009	54.82	0.339
	Disequilibrium	1	128	61	**2.596**	**1.037-6.501**	**0.042**	F	0.0	1.0	0.0	NA
	**Quality score**											
	High quality	11	4017	4296	0.956	0.823-1.112	0.563	R	25.71	0.004	61.10	0.477

	Low quality	3	510	418	1.111	0.838-1.471	0.464	F	4.279	0.118	53.26	0.132

OR: odds ratio, CI: confidence interval, F: fixed model, R: random model, NA: not appreciable

Supplementary Table S24. Meta-analysis of the association between GEMIN4 (G > C; rs2740348) variant and cancer risk.

Comparison	Subgroups	No. of studies	Sample size		Test of association				Test of heterogeneity			Publication bias
			Cancer	Control	OR	95% CI	*P*-value	Model	Q test	*P*-value	I^2 (%)	*P*-value (Egger's)
**Allelic model (C allele *versus* G allele)**	**Overall**	11	9484	10918	0.919	0.824-1.025	0.128	R	17.336	0.067	42.32	0.982
	**Geographical region**											
	Asian	3	2510	2444	0.907	0.763-1.078	0.268	F	2.715	0.257	26.33	0.635
	European	2	1040	1408	1.162	0.930-1.453	0.186	F	2.025	0.155	50.61	NA
	American	5	5310	6128	**0.873**	**0.791-0.963**	**0.007**	F	7.252	0.123	44.85	0.606
	Mexican	1	624	938	0.932	0.724-1.201	0.586	F	0.0	1.0	0.0	NA
	**Cancer type**											
	Breast cancer	2	2556	2494	1.082	0.925-1.266	0.323	F	2.350	0.125	57.45	NA
	Bladder cancer	1	1476	1478	0.896	0.742-1.082	0.255	F	0.0	1.0	0.0	NA
	CML	1	624	938	0.835	0.503-1.387	0.487	F	0.0	1.0	0.0	NA
	CLL	1	210	690	0.932	0.724-1.201	0.586	F	0.0	1.0	0.0	NA
	Colon cancer	1	2230	2346	0.824	0.706-0.962	0.014	F	0.0	1.0	0.0	NA
	Esophageal cancer	1	692	692	1.000	0.756-1.323	1.0	F	0.0	1.0	0.0	NA
	Lung cancer	1	184	180	0.919	0.465-1.814	0.807	F	0.0	1.0	0.0	NA
	NHL	1	360	1056	1.148	0.838-1.573	0.389	F	0.0	1.0	0.0	NA
	Prostate cancer	1	600	488	0.680	0.462-1.000	0.050	F	0.0	1.0	0.0	NA
	Renal cell carcinoma	1	552	556	**0.676**	**0.501-0.911**	**0.010**	F	0.0	1.0	0.0	NA
	**Genotyping method**											
	TaqMan	4	3390	4122	1.024	0.900-1.165	0.719	F	3.978	0.264	24.59	0.720
	HRM method	1	600	488	0.680	0.462-1.000	0.050	F	0.0	1.0	0.0	NA
	SNPlex technology	3	2720	2726	**0.866**	**0.756-0.995**	**0.042**	F	3.790	0.150	47.23	0.748
	Other methods	3	2774	3582	0.881	0.768-1.009	0.067	F	3.456	0.178	42.13	0.595
	**Source of controls**											
	Population-based	5	5548	6086	0.907	0.744-1.104	0.330	R	12.54	0.014	68.09	0.980
	Hospital-based	6	3936	4832	0.929	0.828-1.042	0.210	F	4.668	0.458	0.0	0.888
	**HWE in controls**											
	Equilibrium	10	8884	10430	0.936	0.841-1.041	0.224	R	14.98	0.092	39.90	0.675
	Disequilibrium	1	600	488	0.680	0.462-1.000	0.050	F	0.0	1.0	0.0	NA
	**Quality score**											
	High quality	8	7870	9532	**0.895**	**0.824-0.972**	**0.008**	F	8.457	0.294	17.23	0.713
	Low quality	3	1614	1386	0.943	0.606-1.465	0.793	R	7.054	0.029	71.65	0.594
**Recessive model (CC versus GG+GC)**	**Overall**	9	4350	5125	0.879	0.688-1.123	0.301	F	5.211	0.735	0.0	0.653
	**Geographical region**											
	Asian	1	863	888	0.890	0.421-1.882	0.761	F	0.0	1.0	0.0	NA
	European	2	520	704	0.845	0.413-1.727	0.644	F	0.125	0.724	0.0	NA
	American	5	2655	3064	0.816	0.605-1.102	0.185	F	3.229	0.520	0.0	0.933
	Mexican	1	312	469	1.422	0.676-2.989	0.353	F	0.0	1.0	0.0	NA
	**Cancer type**											
	Breast cancer	2	1278	1247	0.843	0.488-1.455	0.540	F	0.043	0.835	0.0	NA
	Bladder cancer	1	738	739	0.755	0.412-1.383	0.362	F	0.0	1.0	0.0	NA
	CML	1	312	469	1.422	0.676-2.989	0.353	F	0.0	1.0	0.0	NA
	CLL	1	105	345	1.097	0.218-5.518	0.910	F	0.0	1.0	0.0	NA
	Colon cancer	1	1115	1173	0.832	0.515-1.342	0.450	F	0.0	1.0	0.0	NA
	Esophageal cancer	1	346	346	1.000	0.411-2.434	1.000	F	0.0	1.0	0.0	NA
	NHL	1	180	528	1.318	0.563-3.085	0.525	F	0.0	1.0	0.0	NA
	Renal cell carcinoma	1	276	278	0.458	0.194-1.080	0.075	F	0.0	1.0	0.0	NA
	**Genotyping method**											
	TaqMan	4	1695	2061	1.018	0.666-1.557	0.934	F	1.288	0.732	0.0	0.993
	SNPlex technology	3	1360	1363	0.711	0.461-1.095	0.121	F	1.610	0.447	0.0	0.912
	Other methods	2	1295	1701	0.929	0.612-1.410	0.729	F	0.855	0.355	0.0	NA
	**Source of controls**											
	Population-based	5	2774	3043	0.776	0.560-1.074	0.126	F	1.837	0.766	0.0	0.897

	Hospital-based	4	1576	2082	1.036	0.713-1.504	0.854	F	2.063	0.559	0.0	0.343
	**HWE in controls**											
	Equilibrium	9	4350	5125	0.879	0.688-1.123	0.301	F	5.211	0.735	0.0	0.653
	**Quality score**											
	High quality	8	3935	4766	0.888	0.687-1.149	0.367	F	5.140	0.643	0.0	0.644
	Low quality	1	415	359	0.793	0.357-1.760	0.568	F	0.0	1.0	0.0	NA
**Dominant model (GC+CC versus GG)**	**Overall**	11	4742	5459	0.909	0.798-1.036	0.154	R	19.48	0.035	48.67	0.973
	**Geographical region**											
	Asian	3	1255	1222	0.897	0.742-1.083	0.257	F	3.167	0.205	36.86	0.641
	European	2	520	704	1.118	0.639-1.956	0.695	R	3.242	0.072	69.15	NA
	American	5	2655	3064	**0.859**	**0.767-0.962**	**0.009**	F	6.093	0.192	34.36	0.543
	Mexican	1	312	469	0.858	0.636-1.156	0.314	F	0.0	1.0	0.0	NA
	**Cancer type**											
	Breast cancer	2	1278	1247	1.168	0.817-1.669	0.394	R	3.783	0.052	73.57	NA
	Bladder cancer	1	738	739	0.897	0.721-1.115	0.326	F	0.0	1.0	0.0	NA
	CML	1	312	469	0.858	0.636-1.156	0.314	F	0.0	1.0	0.0	NA
	CLL	1	105	345	0.795	0.455-1.391	0.422	F	0.0	1.0	0.0	NA
	Colon cancer	1	1115	1173	**0.794**	**0.665-0.949**	**0.011**	F	0.0	1.0	0.0	NA
	Esophageal cancer	1	346	346	1.000	0.725-1.379	1.000	F	0.0	1.0	0.0	NA
	Lung cancer	1	92	90	0.909	0.441-1.872	0.792	F	0.0	1.0	0.0	NA
	NHL	1	180	528	1.146	0.795-1.653	0.465	F	0.0	1.0	0.0	NA
	Prostate cancer	1	300	244	**0.644**	**0.427-0.973**	**0.037**	F	0.0	1.0	0.0	NA
	Renal cell carcinoma	1	276	278	**0.668**	**0.470-0.950**	**0.025**	F	0.0	1.0	0.0	NA
	**Genotyping method**											
	TaqMan	4	1695	2061	1.020	0.801-1.297	0.875	R	7.029	0.071	57.32	0.780
	HRM method	1	300	244	**0.644**	**0.427-0.973**	**0.037**	F	0.0	1.0	0.0	NA
	SNPlex technology	3	1360	1363	0.867	0.738-1.017	0.080	F	2.958	0.228	32.38	0.721
	Other methods	3	1387	1791	**0.855**	**0.731-0.999**	**0.049**	F	3.144	0.208	36.39	0.561
	**Source of controls**											
	Population-based	5	2774	3043	0.914	0.716-1.167	0.471	R	14.77	0.005	72.92	0.917
	Hospital-based	6	1968	2416	0.904	0.792-1.032	0.135	F	4.712	0.452	0.0	0.885
	**HWE in controls**											
	Equilibrium	10	4442	5215	0.932	0.818-1.062	0.290	R	16.76	0.053	46.29	0.694
	Disequilibrium	1	300	244	**0.644**	**0.427-0.973**	**0.037**	F	0.0	1.0	0.0	NA
	**Quality score**											
	High quality	8	3935	4766	0.879	0.800-0.966	0.007	F	7.417	0.387	5.616	0.794
	Low quality	3	807	693	0.958	0.546-1.682	0.881	R	9.597	0.008	79.16	0.629
**Homozygote model (CC *versus* GG)**	**Overall**	9	3171	3692	0.858	0.671-1.099	0.225	F	5.771	0.673	0.0	0.570
	**Geographical region**											
	Asian	1	678	697	0.889	0.420-1.882	0.758	F	0.0	1.0	0.0	NA
	European	2	341	527	0.937	0.456-1.926	0.859	F	0.022	0.881	0.0	NA
	American	5	1934	2163	0.783	0.579-1.060	0.113	F	4.035	0.401	0.877	0.878
	Mexican	1	218	305	1.327	0.627-2.809	0.460	F	0.0	1.0	0.0	NA
	**Cancer type**											
	Breast cancer	2	931	948	0.899	0.519-1.557	0.705	F	0.002	0.964	0.0	NA
	Bladder cancer	1	526	515	0.735	0.399-1.351	0.321	F	0.0	1.0	0.0	NA
	CML	1	218	305	1.327	0.627-2.809	0.460	F	0.0	1.0	0.0	NA
	CLL	1	88	276	1.047	0.207-5.281	0.956	F	0.0	1.0	0.0	NA
	Colon cancer	1	829	821	0.779	0.481-1.261	0.309	F	0.0	1.0	0.0	NA
	Esophageal cancer	1	248	248	1.000	0.409-2.447	1.000	F	0.0	1.0	0.0	NA
	NHL	1	131	394	1.359	0.576-3.202	0.484	F	0.0	1.0	0.0	NA
	Renal cell carcinoma	1	200	185	**0.412**	**0.173-0.978**	**0.044**	F	0.0	1.0	0.0	NA
	**Genotyping method**											
	TaqMan	4	1237	1529	1.031	0.673-1.581	0.888	F	0.675	0.879	0.0	0.974
	SNPlex technology	3	974	948	0.683	0.442-1.055	0.085	F	2.066	0.356	3.173	0.897
	Other methods	2	960	1215	0.890	0.585-1.355	0.587	F	1.229	0.268	18.64	NA
	**Source of controls**											
	Population-based	5	2048	2230	0.757	0.546-1.051	0.096	F	2.449	0.654	0.0	0.993
	Hospital-based	4	1123	1462	1.013	0.695-1.474	0.948	F	2.018	0.569	0.0	0.272

	**HWE in controls**											
	Equilibrium	9	3171	3692	0.858	0.671-1.099	0.225	F	5.771	0.673	0.0	0.570
	**Quality score**											
	High quality	8	2918	3441	0.853	0.658-1.106	0.230	F	5.747	0.570	0.0	0.613
	Low quality	1	253	251	0.912	0.408-2.039	0.822	F	0.0	1.0	0.0	NA
**Heterozygote model (GC *versus* GG)**	**Overall**	11	4625	5301	**0.870**	**0.781-0.969**	**0.011**	R	19.101	0.039	47.65	0.988
	**Geographical region**											
	Asian	3	1242	1207	0.898	0.741-1.089	0.274	F	3.241	0.198	38.29	0.646
	European	2	506	685	1.125	0.600-2.111	0.713	R	3.755	0.053	73.37	NA
	American	5	2579	2955	**0.869**	**0.772-0.977**	**0.019**	F	4.470	0.346	10.52	0.460
	Mexican	1	298	454	0.815	0.597-1.111	0.196	F	0.0	1.0	0.0	NA
	**Cancer type**											
	Breast cancer	2	1253	1219	1.198	0.810-1.770	0.366	R	4.270	0.039	76.58	NA
	Bladder cancer	1	719	714	0.915	0.730-1.146	0.438	F	0.0	1.0	0.0	NA
	CML	1	298	454	0.815	0.597-1.111	0.196	F	0.0	1.0	0.0	NA
	CLL	1	103	339	0.774	0.432-1.386	0.388	F	0.0	1.0	0.0	NA
	Colon cancer	1	1084	1134	**0.796**	**0.662-0.958**	**0.016**	F	0.0	1.0	0.0	NA
	Esophageal cancer	1	336	336	1.000	0.717-1.395	1.000	F	0.0	1.0	0.0	NA
	Lung cancer	1	92	90	0.909	0.441-1.872	0.796	F	0.0	1.0	0.0	NA
	NHL	1	172	510	1.118	0.760-1.643	0.571	F	0.0	1.0	0.0	NA
	Prostate cancer	1	300	244	**0.644**	**0.427-0.973**	**0.037**	F	0.0	1.0	0.0	NA
	Renal cell carcinoma	1	268	261	0.715	0.495-1.032	0.073	F	0.0	1.0	0.0	NA
	**Genotyping method**											
	TaqMan	4	1654	2012	1.013	0.766-1.339	0.929	R	8.763	0.033	65.77	0.764
	HRM method	1	300	244	**0.644**	**0.427-0.973**	**0.037**	F	0.0	1.0	0.0	NA
	SNPlex technology	3	1323	1311	0.889	0.753-1.050	0.166	F	1.894	0.388	0.0	0.711
	Other methods	3	1348	1734	0.851	0.724-1.001	0.052	F	2.455	0.293	18.54	0.538
	**Source of controls**											
	Population-based	5	2708	2953	0.934	0.726-1.200	0.592	R	14.48	0.006	72.37	0.889
	Hospital-based	6	1917	2348	0.896	0.782-1.027	0.114	F	4.536	0.475	0.0	0.841
	**HWE in controls**											
	Equilibrium	10	4325	5057	0.938	0.821-1.071	0.344	R	16.27	0.061	44.69	0.742
	Disequilibrium	1	300	244	**0.644**	**0.427-0.973**	**0.037**	F	0.0	1.0	0.0	NA
	**Quality score**											
	High quality	8	3830	4621	0.882	0.800-0.972	0.011	F	6.019	0.538	0.0	0.865
	Low quality	3	795	680	0.971	0.537-1.755	0.922	R	10.45	0.005	80.86	0.641

OR: odds ratio, CI: confidence interval, F: fixed model, R: random model, NA: not appreciable

Supplementary Table S25. Meta-analysis of the association between GEMIN4 (C > T; rs3744741) variant and cancer risk.

Comparison	Subgroups	No. of studies	Sample size		Test of association				Test of heterogeneity			Publication bias
			Cancer	Control	OR	95% CI	*P*-value	Model	Q test	*P*-value	I^2 (%)	*P*-value (Egger's)
**Allelic model (T allele *versus* C allele)**	**Overall**	9	6784	7464	0.957	0.880-1.041	0.307	F	9.710	0.286	17.61	0.883
	**Geographical region**											
	Asian	5	3842	4044	1.012	0.914-1.120	0.825	F	4.024	0.403	0.586	0.727
	European	1	212	694	0.827	0.508-1.346	0.445	F	0.0	1.0	0.0	NA
	American	3	2730	2726	**0.854**	**0.731-0.998**	**0.047**	F	2.136	0.344	6.369	0.667
	**Cancer type**											
	Breast cancer	2	2842	2908	1.010	0.898-1.137	0.863	F	0.690	0.406	0.0	NA
	Renal cell carcinoma	2	754	1004	1.150	0.897-1.473	0.270	F	0.477	0.490	0.0	NA
	Bladder cancer	1	1484	1480	0.822	0.665-1.017	0.071	F	0.0	1.0	0.0	NA
	CLL	1	212	694	0.827	0.508-1.346	0.445	F	0.0	1.0	0.0	NA
	Esophageal cancer	1	692	692	0.771	0.565-1.051	0.099	F	0.0	1.0	0.0	NA
	Prostate cancer	1	600	488	0.846	0.638-1.121	0.245	F	0.0	1.0	0.0	NA
	Lung cancer	1	200	198	1.167	0.734-1.854	0.514	F	0.0	1.0	0.0	NA
	**Genotyping method**											
	TaqMan	4	3254	4052	1.020	0.915-1.138	0.716	F	2.747	0.432	0.0	0.935
	SNPlex technology	3	2730	2726	**0.854**	**0.731-0.998**	**0.047**	F	2.136	0.344	6.369	0.667
	Other methods	2	800	686	0.923	0.725-1.174	0.512	F	1.348	0.246	25.82	NA
	**Source of controls**											
	Population-based	4	2690	3472	1.057	0.935-1.195	0.376	F	1.902	0.593	0.0	0.916
	Hospital-based	5	4094	3992	**0.878**	**0.783-0.985**	**0.026**	F	3.125	0.537	0.0	0.697
	**HWE in controls**											
	Equilibrium	9	6784	7464	0.957	0.880-1.041	0.307	F	9.710	0.286	17.61	0.883
	**Quality score**											
	High quality	8	6584	7266	0.951	0.873-1.035	0.246	F	8.987	0.254	22.11	0.613
	Low quality	1	200	198	1.167	0.734-1.854	0.514	F	0.0	1.0	0.0	NA
**Recessive model (TT *versus* CC+CT)**	**Overall**	9	3392	3732	1.027	0.824-1.281	0.813	F	7.236	0.511	0.0	0.482
	**Geographical region**											
	Asian	5	1921	2022	1.054	0.826-1.346	0.672	F	3.474	0.482	0.0	0.637
	European	1	106	347	3.317	0.462-23.84	0.233	F	0.0	1.0	0.0	NA
	American	3	1365	1363	0.831	0.487-1.420	0.499	F	1.762	0.414	0.0	0.693
	**Cancer type**											
	Breast cancer	2	1421	1454	1.015	0.761-1.355	0.918	F	0.223	0.637	0.0	NA
	Renal cell carcinoma	2	377	502	1.838	0.971-3.480	0.061	F	0.072	0.788	0.0	NA
	Bladder cancer	1	742	740	0.715	0.348-1.471	0.362	F	0.0	1.0	0.0	NA
	CLL	1	106	347	3.317	0.462-23.84	0.233	F	0.0	1.0	0.0	NA
	Esophageal cancer	1	346	346	0.620	0.201-1.913	0.405	F	0.0	1.0	0.0	NA
	Prostate cancer	1	300	244	0.800	0.420-1.524	0.497	F	0.0	1.0	0.0	NA
	Lung cancer	1	100	99	1.200	0.354-4.068	0.770	F	0.0	1.0	0.0	NA
	**Genotyping method**											
	TaqMan	4	1627	2026	1.122	0.859-1.467	0.399	F	3.814	0.282	21.35	0.219
	SNPlex technology	3	1365	1363	0.831	0.487-1.420	0.499	F	1.762	0.414	0.0	0.693
	Other methods	2	400	343	0.874	0.494-1.545	0.643	F	0.331	0.565	0.0	NA
	**Source of controls**											
	Population-based	4	1345	1736	1.237	0.914-1.676	0.169	F	3.217	0.359	6.754	**0.095**
	Hospital-based	5	2047	1996	0.833	0.603-1.149	0.265	F	0.934	0.920	0.0	0.834
	**HWE in controls**											
	Equilibrium	9	3392	3732	1.027	0.824-1.281	0.813	F	7.236	0.511	0.0	0.482
	**Quality score**											
	High quality	8	3292	3633	1.022	0.816-1.279	0.852	F	7.172	0.411	2.394	0.543
	Low quality	1	100	99	1.200	0.354-4.068	0.770	F	0.0	1.0	0.0	NA
**Dominant model (CT+TT versus CC)**	**Overall**	9	3392	3732	0.935	0.845-1.033	0.187	F	7.521	0.482	0.0	0.799
	**Geographical region**											
	Asian	5	1921	2022	1.004	0.885-1.140	0.949	F	2.594	0.628	0.0	0.836
	European	1	106	347	0.738	0.432-1.262	0.267	F	0.0	1.0	0.0	NA

	American	3	1365	1363	**0.837**	**0.704-0.996**	**0.045**	F	1.411	0.494	0.0	0.686
	Cancer type											
	Breast cancer	2	1421	1454	1.012	0.874-1.173	0.870	F	0.638	0.424	0.0	NA
	Renal cell carcinoma	2	377	502	1.072	0.797-1.440	0.647	F	0.173	0.677	0.0	NA
	Bladder cancer	1	742	740	0.813	0.642-1.030	0.086	F	0.0	1.0	0.0	NA
	CLL	1	106	347	0.738	0.432-1.262	0.267	F	0.0	1.0	0.0	NA
	Esophageal cancer	1	346	346	0.758	0.537-1.070	0.115	F	0.0	1.0	0.0	NA
	Prostate cancer	1	300	244	0.829	0.587-1.172	0.289	F	0.0	1.0	0.0	NA
	Lung cancer	1	100	99	1.209	0.688-2.125	0.510	F	0.0	1.0	0.0	NA
	Genotyping method											
	TaqMan	4	1627	2026	1.003	0.876-1.149	0.963	F	2.269	0.519	0.0	0.592
	SNPlex technology	3	1365	1363	**0.837**	**0.704-0.996**	**0.045**	F	1.411	0.494	0.0	0.686
	Other methods	2	400	343	0.919	0.685-1.234	0.575	F	1.245	0.265	19.68	NA
	Source of controls											
	Population-based	4	1345	1736	1.034	0.888-1.203	0.670	F	1.823	0.610	0.0	0.513
	Hospital-based	5	2047	1996	**0.864**	**0.755-0.988**	**0.033**	F	2.690	0.611	0.0	0.547
	HWE in controls											
	Equilibrium	9	3392	3732	0.935	0.845-1.033	0.187	F	7.521	0.482	0.0	0.799
	Quality score											
	High quality	8	3292	3633	0.927	0.837-1.026	0.144	F	6.696	0.461	0.0	0.447
	Low quality	1	100	99	1.209	0.688-2.125	0.510	F	0.0	1.0	0.0	NA
**Homozygote model (TT *versus* CC)**	Overall	9	2362	2550	1.017	0.812-1.274	0.882	F	7.768	0.456	0.0	0.557
	Geographical region											
	Asian	5	1204	1259	1.053	0.820-1.353	0.685	F	3.789	0.435	0.0	0.669
	European	1	87	262	3.059	0.424-22.05	0.267	F	0.0	1.0	0.0	NA
	American	3	1071	1029	0.799	0.467-1.367	0.413	F	1.935	0.380	0.0	0.698
	Cancer type											
	Breast cancer	2	870	893	1.024	0.761-1.376	0.877	F	0.365	0.546	0.0	NA
	Renal cell carcinoma	2	276	349	1.835	0.954-3.530	0.069	F	0.078	0.780	0.0	NA
	Bladder cancer	1	586	561	0.684	0.332-1.410	0.304	F	0.0	1.0	0.0	NA
	CLL	1	87	262	3.059	0.424-22.05	0.267	F	0.0	1.0	0.0	NA
	Esophageal cancer	1	273	258	0.583	0.188-1.806	0.350	F	0.0	1.0	0.0	NA
	Prostate cancer	1	208	162	0.755	0.392-1.457	0.403	F	0.0	1.0	0.0	NA
	Lung cancer	1	62	65	1.286	0.372-4.449	0.692	F	0.0	1.0	0.0	NA
	Genotyping method											
	TaqMan	4	1021	1294	1.129	0.857-1.485	0.389	F	3.583	0.310	16.27	0.260
	SNPlex technology	3	1071	1029	0.799	0.467-1.367	0.413	F	1.935	0.380	0.0	0.698
	Other methods	2	270	227	0.849	0.475-1.517	0.579	F	0.551	0.458	0.0	NA
	Source of controls											
	Population-based	4	872	1144	1.259	0.922-1.720	0.147	F	2.734	0.434	0.0	**0.098**
	Hospital-based	5	1490	1406	0.805	0.581-1.116	0.193	F	1.261	0.868	0.0	0.936
	HWE in controls											
	Equilibrium	9	2362	2550	1.017	0.812-1.274	0.882	F	7.768	0.456	0.0	0.557
	Quality score											
	High quality	8	2300	2485	1.009	0.802-1.269	0.938	F	7.627	0.367	8.216	0.651
	Low quality	1	62	65	1.286	0.372-4.449	0.692	F	0.0	1.0	0.0	NA
**Heterozygote model (CT *versus* CC)**	Overall	9	3226	3558	0.928	0.835-1.030	0.161	F	5.844	0.665	0.0	0.520
	Geographical region											
	Asian	5	1783	1881	0.997	0.872-1.139	0.964	F	1.629	0.804	0.0	0.937
	European	1	104	345	0.684	0.393-1.190	0.179	F	0.0	1.0	0.0	NA
	American	3	1339	1332	0.841	0.703-1.006	0.058	F	0.777	0.678	0.0	0.720
	Cancer type											
	Breast cancer	2	1322	1354	1.012	0.867-1.180	0.883	F	0.475	0.491	0.0	NA
	Renal cell carcinoma	2	356	481	0.988	0.724-1.347	0.938	F	0.011	0.915	0.0	NA
	Bladder cancer	1	729	722	0.826	0.647-1.055	0.125	F	0.0	1.0	0.0	NA
	CLL	1	104	345	0.684	0.393-1.190	0.179	F	0.0	1.0	0.0	NA
	Esophageal cancer	1	341	338	0.774	0.543-1.104	0.157	F	0.0	1.0	0.0	NA
	Prostate cancer	1	280	224	0.847	0.586-1.226	0.379	F	0.0	1.0	0.0	NA

	Lung cancer	1	94	94	1.197	0.665-2.158	0.549	F	0.0	1.0	0.0	NA
	**Genotyping method**											
	TaqMan	4	1513	1908	0.986	0.855-1.137	0.842	F	2.265	0.519	0.0	0.289
	SNPlex technology	3	1339	1332	0.841	0.703-1.006	0.058	F	0.777	0.678	0.0	0.719
	Other methods	2	374	318	0.934	0.683-1.277	0.669	F	0.951	0.329	0.0	NA
	**Source of controls**											
	Population-based	4	1253	1646	1.001	0.854-1.174	0.989	F	2.119	0.548	0.0	0.250
	Hospital-based	5	1973	1912	0.875	0.761-1.006	0.061	F	2.165	0.705	0.0	0.528
	**HWE in controls**											
	Equilibrium	9	3226	3558	0.928	0.835-1.030	0.161	F	5.844	0.665	0.0	0.520
	**Quality score**											
	High quality	8	3132	3464	0.920	0.827-1.024	0.125	F	5.098	0.648	0.0	0.193
	Low quality	1	94	94	1.197	0.665-2.158	0.549	F	0.0	1.0	0.0	NA

OR: odds ratio, CI: confidence interval, F: fixed model, R: random model, NA: not appreciable

Supplementary Table S26. Meta-analysis of the association between PIWIL1 rs1106042 G > A variant and cancer risk.

Comparison	Subgroups	No. of studies	Sample size		Test of association				Test of heterogeneity			Publication bias
			Cancer	Control	OR	95% CI	*P*-value	Model	Q test	*P*-value	I^2 (%)	*P*-value (Egger's)
**Allelic model (A allele *versus* G allele)**	**Overall**	5	3494	5834	0.878	0.745-1.034	0.119	F	7.492	0.112	46.61	0.678
	**Geographical region**											
	Asian	2	1344	3296	0.889	0.541-1.462	0.644	R	2.753	0.097	63.67	NA
	European	1	208	686	0.774	0.382-1.572	0.479	F	0.0	1.0	0.0	NA
	American	2	1942	1852	1.037	0.797-1.350	0.786	F	2.205	0.138	54.66	NA
	**Cancer type**											
	Head/Neck carcinoma	1	1150	3102	**0.732**	**0.576-0.928**	**0.010**	F	0.0	1.0	0.0	NA
	CLL	1	208	686	0.774	0.382-1.572	0.479	F	0.0	1.0	0.0	NA
	Lung cancer	1	194	194	1.237	0.698-2.192	0.467	F	0.0	1.0	0.0	NA
	Bladder cancer	1	1416	1382	1.178	0.862-1.609	0.305	F	0.0	1.0	0.0	NA
	Renal cell carcinoma	1	526	470	0.758	0.464-1.238	0.268	F	0.0	1.0	0.0	NA
	**Genotyping method**											
	TaqMan	1	208	686	0.774	0.382-1.572	0.479	F	0.0	1.0	0.0	NA
	SNPlex technology	2	1942	1852	1.037	0.797-1.350	0.786	F	2.205	0.138	54.66	NA
	Other methods	2	1344	3296	0.889	0.541-1.462	0.644	R	2.753	0.097	63.67	NA
	**Source of controls**											
	Population-based	3	1884	4258	**0.740**	**0.602-0.908**	**0.004**	F	0.034	0.983	0.0	**0.054**
	Hospital-based	2	1610	1576	1.191	0.905-1.567	0.212	F	0.022	0.883	0.0	NA
	**HWE in controls**											
	Equilibrium	5	3494	5834	0.878	0.745-1.034	0.119	F	7.492	0.112	46.61	0.678
	**Quality score**											
	High quality	4	3300	5640	0.851	0.717-1.010	0.065	F	5.990	0.112	49.91	0.959
	Low quality	1	194	194	1.237	0.698-2.192	0.467	F	0.0	1.0	0.0	NA
**Recessive model (AA versus GG+GA)**	**Overall**	5	1747	2917	0.899	0.414-1.953	0.789	F	3.713	0.446	0.0	0.700
	**Geographical region**											
	Asian	2	672	1648	0.895	0.332-2.410	0.826	F	1.379	0.240	27.47	NA
	European	1	104	343	2.222	0.366-13.48	0.385	F	0.0	1.0	0.0	NA
	American	2	971	926	0.401	0.072-2.241	0.298	F	0.520	0.471	0.0	NA
	**Cancer type**											
	Head/Neck carcinoma	1	575	1551	0.672	0.224-2.019	0.479	F	0.0	1.0	0.0	NA
	CLL	1	104	343	2.222	0.366-13.48	0.385	F	0.0	1.0	0.0	NA
	Lung cancer	1	97	97	3.064	0.313-29.98	0.336	F	0.0	1.0	0.0	NA
	Bladder cancer	1	708	691	0.243	0.027-2.179	0.206	F	0.0	1.0	0.0	NA
	Renal cell carcinoma	1	263	235	0.893	0.056-14.36	0.936	F	0.0	1.0	0.0	NA
	**Genotyping method**											
	TaqMan	1	104	343	2.222	0.366-13.48	0.385	F	0.0	1.0	0.0	NA
	SNPlex technology	2	971	926	0.401	0.072-2.241	0.298	F	0.520	0.471	0.0	NA
	Other methods	2	672	1648	0.895	0.332-2.410	0.826	F	1.379	0.240	27.47	NA
	**Source of controls**											
	Population-based	3	942	2129	0.926	0.380-2.253	0.865	F	1.233	0.540	0.0	0.645
	Hospital-based	2	805	788	0.821	0.169-3.992	0.807	F	2.464	0.116	59.42	NA
	**HWE in controls**											
	Equilibrium	5	1747	2917	0.899	0.414-1.953	0.789	F	3.713	0.446	0.0	0.700
	**Quality score**											
	High quality	4	1650	2820	0.766	0.336-1.748	0.527	F	2.460	0.483	0.0	0.996
	Low quality	1	97	97	3.064	0.313-29.98	0.336	F	0.0	1.0	0.0	NA
**Dominant model (GA+AA versus GG)**	**Overall**	5	1747	2917	0.885	0.658-1.190	0.419	R	8.785	0.067	54.47	0.884
	**Geographical region**											
	Asian	2	672	1648	**0.762**	**0.602-0.966**	**0.025**	F	2.017	0.156	50.42	NA
	European	1	104	343	0.650	0.293-1.438	0.287	F	0.0	1.0	0.0	NA
	American	2	971	926	0.999	0.601-1.661	0.997	R	2.833	0.092	64.70	NA
	**Cancer type**											
	Head/Neck carcinoma	1	575	1551	**0.712**	**0.552-0.919**	**0.009**	F	0.0	1.0	0.0	NA
	CLL	1	104	343	0.650	0.293-1.438	0.287	F	0.0	1.0	0.0	NA
	Lung cancer	1	97	97	1.173	0.619-2.225	0.625	F	0.0	1.0	0.0	NA
	Bladder cancer	1	708	691	1.249	0.900-1.732	0.184	F	0.0	1.0	0.0	NA
	Renal cell carcinoma	1	263	235	0.739	0.441-1.238	0.250	F	0.0	1.0	0.0	NA
	**Genotyping method**											
	TaqMan	1	104	343	0.650	0.293-1.438	0.287	F	0.0	1.0	0.0	NA

	SNPlex technology	2	971	926	0.999	0.601-1.661	0.997	R	2.833	0.092	64.70	NA
	Other methods	2	672	1648	**0.762**	**0.602-0.966**	**0.025**	F	2.017	0.156	50.42	NA
	**Source of controls**											
	Population-based	3	942	2129	**0.712**	**0.571-0.887**	**0.002**	F	0.071	0.965	0.0	0.728
	Hospital-based	2	805	788	1.233	0.921-1.650	0.160	F	0.029	0.865	0.0	NA
	**HWE in controls**											
	Equilibrium	5	1747	2917	0.885	0.658-1.190	0.419	R	8.785	0.067	54.47	0.884
	**Quality score**											
	High quality	4	1650	2820	0.844	0.602-1.181	0.322	R	7.869	0.049	61.87	0.905
	Low quality	1	97	97	1.173	0.619-2.225	0.625	F	0.0	1.0	0.0	NA
**Homozygote model (AA *versus* GG)**	**Overall**	5	1510	2446	0.867	0.399-1.885	0.719	F	3.687	0.450	0.0	0.659
	**Geographical region**											
	Asian	2	561	1317	0.856	0.317-2.307	0.758	F	1.520	0.218	34.23	NA
	European	1	98	307	2.111	0.348-12.82	0.417	F	0.0	1.0	0.0	NA
	American	2	851	822	0.402	0.072-2.248	0.299	F	0.465	0.495	0.0	NA
	**Cancer type**											
	Head/Neck carcinoma	1	488	1243	0.634	0.211-1.905	0.417	F	0.0	1.0	0.0	NA
	CLL	1	98	307	2.111	0.348-12.82	0.417	F	0.0	1.0	0.0	NA
	Lung cancer	1	73	74	3.129	0.318-30.80	0.328	F	0.0	1.0	0.0	NA
	Bladder cancer	1	618	622	0.250	0.028-2.247	0.216	F	0.0	1.0	0.0	NA
	Renal cell carcinoma	1	233	200	0.858	0.053-13.80	0.914	F	0.0	1.0	0.0	NA
	**Genotyping method**											
	TaqMan	1	98	307	2.111	0.348-12.82	0.417	F	0.0	1.0	0.0	NA
	SNPlex technology	2	851	822	0.402	0.072-2.248	0.299	F	0.465	0.495	0.0	NA
	Other methods	2	561	1317	0.856	0.317-2.307	0.758	F	1.520	0.218	34.23	NA
	**Source of controls**											
	Population-based	3	819	1750	0.876	0.360-2.134	0.771	F	1.246	0.536	0.0	0.639
	Hospital-based	2	691	696	0.840	0.172-4.092	0.829	F	2.439	0.118	59.01	NA
	**HWE in controls**											
	Equilibrium	5	1510	2446	0.867	0.399-1.885	0.719	F	3.687	0.450	0.0	0.659
	**Quality score**											
	High quality	4	1437	2372	0.734	0.322-1.675	0.463	F	2.321	0.509	0.0	0.966
	Low quality	1	73	74	3.129	0.318-30.80	0.328	F	0.0	1.0	0.0	NA
**Heterozygote model (GA *versus* GG)**	**Overall**	5	1736	2892	0.872	0.630-1.208	0.410	R	9.922	0.042	59.69	0.918
	**Geographical region**											
	Asian	2	665	1631	**0.758**	**0.595-0.965**	**0.025**	F	1.339	0.247	25.34	NA
	European	1	102	340	0.528	0.216-1.291	0.161	F	0.0	1.0	0.0	NA
	American	2	969	921	1.017	0.582-1.778	0.953	R	3.302	0.069	69.72	NA
	**Cancer type**											
	Head/Neck carcinoma	1	571	1535	**0.716**	**0.552-0.929**	**0.012**	F	0.0	1.0	0.0	NA
	CLL	1	102	340	0.528	0.216-1.291	0.161	F	0.0	1.0	0.0	NA
	Lung cancer	1	94	96	1.088	0.563-2.104	0.802	F	0.0	1.0	0.0	NA
	Bladder cancer	1	707	687	1.306	0.937-1.822	0.115	F	0.0	1.0	0.0	NA
	Renal cell carcinoma	1	262	234	0.735	0.436-1.241	0.249	F	0.0	1.0	0.0	NA
	**Genotyping method**											
	TaqMan	1	102	340	0.528	0.216-1.291	0.161	F	0.0	1.0	0.0	NA
	SNPlex technology	2	969	921	1.017	0.582-1.778	0.953	R	3.302	0.069	69.72	NA
	Other methods	2	665	1631	**0.758**	**0.595-0.965**	**0.025**	F	1.339	0.247	25.34	NA
	**Source of controls**											
	Population-based	3	935	2109	0.706	0.563-0.884	0.002	F	0.441	0.802	0.0	0.480
	Hospital-based	2	801	783	1.259	0.935-1.694	0.129	F	0.235	0.628	0.0	NA
	**HWE in controls**											
	Equilibrium	5	1736	2892	0.872	0.630-1.208	0.410	R	9.922	0.042	59.69	0.918
	**Quality score**											
	High quality	4	1642	2796	0.834	0.569-1.222	0.352	R	9.454	0.024	68.27	0.791
	Low quality	1	94	96	1.088	0.563-2.104	0.802	F	0.0	1.0	0.0	NA

OR: odds ratio, CI: confidence interval, F: fixed model, R: random model, NA: not appreciable

Supplementary Table S27. Meta-analysis of the association between PIWIL1 rs10773771 C > T variant and cancer risk.

Comparison	Subgroups	No. of studies	Sample size		Test of association				Test of heterogeneity			Publication bias
			Cancer	Control	OR	95% CI	*P*-value	Model	Q test	*P*-value	I^2 (%)	*P*-value (Egger's)
**Allelic model (T allele *versus* C allele)**	**Overall**	7	10580	13696	0.971	0.921-1.023	0.267	F	5.035	0.539	0.0	0.676
	**Geographical region**											
	Asian	6	9774	12996	0.962	0.911-1.015	0.158	F	3.267	0.659	0.0	0.744
	European	1	806	700	1.111	0.904-1.366	0.316	F	0.0	1.0	0.0	NA
	**Cancer type**											
	Breast cancer	2	2514	2452	1.082	0.966-1.212	0.174	F	0.095	0.758	0.0	NA
	HCC	2	3194	3308	0.952	0.862-1.051	0.332	F	0.055	0.815	0.0	NA
	Head/Neck carcinoma	2	1934	4884	0.926	0.831-1.031	0.158	F	0.252	0.616	0.0	NA
	Cervical carcinoma	1	2938	3052	0.947	0.854-1.049	0.298	F	0.0	1.0	0.0	NA
	**Genotyping method**											
	TaqMan	6	9430	10596	0.975	0.921-1.032	0.380	F	4.884	0.430	0.0	0.697
	Other methods	1	1150	3100	0.946	0.824-1.087	0.435	F	0.0	1.0	0.0	NA
	**Source of controls**											
	Population-based	6	9956	13056	0.973	0.922-1.027	0.324	F	4.883	0.430	0.0	0.460
	Hospital-based	1	624	640	0.930	0.744-1.162	0.522	F	0.0	1.0	0.0	NA
	**HWE in controls**											
	Equilibrium	6	9796	11912	0.979	0.926-1.035	0.452	F	4.067	0.540	0.0	0.435
	Disequilibrium	1	784	1784	0.895	0.754-1.061	0.201	F	0.0	1.0	0.0	NA
	**Quality score**											
	High quality	6	9774	12996	0.962	0.911-1.015	0.158	F	3.267	0.659	0.0	0.744
	Low quality	1	806	700	1.111	0.904-1.366	0.316	F	0.0	1.0	0.0	NA
**Recessive model (TT versus CC+CT)**	**Overall**	7	5290	6848	1.004	0.872-1.157	0.953	R	11.81	0.066	49.21	0.476
	**Geographical region**											
	Asian	6	4887	6498	0.956	0.864-1.056	0.373	F	4.016	0.547	0.0	0.701
	European	1	403	350	**1.658**	**1.141-2.408**	**0.008**	F	0.0	1.0	0.0	NA
	**Cancer type**											
	Breast cancer	2	1257	1226	1.310	0.866-1.981	0.201	R	3.398	0.065	70.57	NA
	HCC	2	1597	1654	1.034	0.863-1.239	0.715	F	0.218	0.641	0.0	NA
	Head/Neck carcinoma	2	967	2442	0.868	0.712-1.059	0.163	F	0.695	0.405	0.0	NA
	Cervical carcinoma	1	1469	1526	0.892	0.739-1.076	0.232	F	0.0	1.0	0.0	NA
	**Genotyping method**											
	TaqMan	6	4715	5298	1.021	0.863-1.207	0.811	R	11.55	0.041	56.71	0.510
	Other methods	1	575	1550	0.931	0.719-1.206	0.589	F	0.0	1.0	0.0	NA
	**Source of controls**											
	Population-based	6	4978	6528	1.012	0.864-1.186	0.883	R	11.77	0.038	57.51	0.379
	Hospital-based	1	312	320	0.951	0.641-1.412	0.805	F	0.0	1.0	0.0	NA
	**HWE in controls**											
	Equilibrium	6	4898	5956	1.038	0.897-1.201	0.616	R	9.390	0.094	46.75	0.251
	Disequilibrium	1	392	892	0.784	0.575-1.070	0.125	F	0.0	1.0	0.0	NA
	**Quality score**											
	High quality	6	4887	6498	0.956	0.864-1.056	0.373	F	4.016	0.547	0.0	0.701
	Low quality	1	403	350	**1.658**	**1.141-2.408**	**0.008**	F	0.0	1.0	0.0	NA
**Dominant model (CT+TT versus CC)**	**Overall**	7	5290	6848	0.944	0.874-1.019	0.138	F	3.261	0.775	0.0	0.782
	**Geographical region**											
	Asian	6	4887	6498	0.947	0.875-1.025	0.181	F	3.132	0.680	0.0	0.952
	European	1	403	350	0.896	0.667-1.203	0.465	F	0.0	1.0	0.0	NA
	**Cancer type**											
	Breast cancer	2	1257	1226	1.031	0.874-1.215	0.719	F	1.259	0.262	20.54	NA
	HCC	2	1597	1654	0.883	0.765-1.019	0.089	F	0.0	0.990	0.0	NA
	Head/Neck carcinoma	2	967	2442	0.929	0.796-1.085	0.353	F	0.0	0.985	0.0	NA
	Cervical carcinoma	1	1469	1526	0.957	0.824-1.113	0.571	F	0.0	1.0	0.0	NA
	**Genotyping method**											

	TaqMan	6	4715	5298	0.946	0.871-1.028	0.191	F	3.237	0.664	0.0	0.810
	Other methods	1	575	1550	0.930	0.762-1.135	0.477	F	0.0	1.0	0.0	NA
	**Source of controls**											
	Population-based	6	4978	6528	0.947	0.876-1.025	0.178	F	3.102	0.684	0.0	0.989
	Hospital-based	1	312	320	0.885	0.638-1.227	0.463	F	0.0	1.0	0.0	NA
	**HWE in controls**											
	Equilibrium	6	4898	5956	0.946	0.873-1.025	0.172	F	3.240	0.663	0.0	0.837
	Disequilibrium	1	392	892	0.927	0.724-1.188	0.551	F	0.0	1.0	0.0	NA
	**Quality score**											
	High quality	6	4887	6498	0.947	0.875-1.025	0.181	F	3.132	0.680	0.0	0.952
	Low quality	1	403	350	0.896	0.667-1.203	0.465	F	0.0	1.0	0.0	NA
**Homozygote model (TT *versus* CC)**	**Overall**	7	2841	3597	0.957	0.860-1.066	0.424	F	7.017	0.319	14.50	0.560
	**Geographical region**											
	Asian	6	2590	3415	0.930	0.833-1.040	0.204	F	3.362	0.644	0.0	0.741
	European	1	251	182	1.408	0.935-2.122	0.102	F	0.0	1.0	0.0	NA
	**Cancer type**											
	Breast cancer	2	686	637	1.217	0.965-1.535	0.097	F	0.713	0.398	0.0	NA
	HCC	2	878	854	0.950	0.777-1.161	0.616	F	0.117	0.732	0.0	NA
	Head/Neck carcinoma	2	513	1302	0.850	0.682-1.058	0.146	F	0.380	0.538	0.0	NA
	Cervical carcinoma	1	764	804	0.884	0.717-1.091	0.251	F	0.0	1.0	0.0	NA
	**Genotyping method**											
	TaqMan	6	2537	2788	0.967	0.861-1.086	0.571	F	6.805	0.236	26.53	0.586
	Other methods	1	304	809	0.900	0.677-1.196	0.467	F	0.0	1.0	0.0	NA
	**Source of controls**											
	Population-based	6	2669	3427	0.962	0.861-1.074	0.490	F	6.896	0.229	27.49	0.392
	Hospital-based	1	172	170	0.887	0.570-1.381	0.595	F	0.0	1.0	0.0	NA
	**HWE in controls**											
	Equilibrium	6	2632	3104	0.978	0.874-1.095	0.703	F	5.546	0.353	9.841	0.320
	Disequilibrium	1	209	493	0.782	0.554-1.103	0.161	F	0.0	1.0	0.0	NA
	**Quality score**											
	High quality	6	2590	3415	0.930	0.833-1.040	0.204	F	3.362	0.644	0.0	0.741
	Low quality	1	251	182	1.408	0.935-2.122	0.102	F	0.0	1.0	0.0	NA
**Heterozygote model (CT *versus* CC)**	**Overall**	7	4362	5638	0.938	0.865-1.018	0.124	F	5.967	0.427	0.0	0.517
	**Geographical region**											
	Asian	6	4051	5341	0.954	0.877-1.038	0.275	F	3.544	0.617	0.0	0.914
	European	1	311	297	0.734	0.533-1.010	0.058	F	0.0	1.0	0.0	NA
	**Cancer type**											
	Breast cancer	2	1019	1033	0.910	0.622-1.332	0.627	R	4.017	0.045	75.11	NA
	HCC	2	1313	1368	0.859	0.738-1.001	0.052	F	0.030	0.862	0.0	NA
	Head/Neck carcinoma	2	808	1993	0.962	0.815-1.134	0.641	F	0.101	0.751	0.0	NA
	Cervical carcinoma	1	1222	1244	0.986	0.840-1.157	0.862	F	0.0	1.0	0.0	NA
	**Genotyping method**											
	TaqMan	6	3880	4354	0.938	0.859-1.024	0.152	F	5.966	0.310	16.19	0.566
	Other methods	1	482	1284	0.941	0.762-1.163	0.574	F	0.0	1.0	0.0	NA
	**Source of controls**											
	Population-based	6	4109	5381	0.941	0.866-1.023	0.157	F	5.849	0.321	14.52	0.605
	Hospital-based	1	253	257	0.884	0.622-1.255	0.490	F	0.0	1.0	0.0	NA
	**HWE in controls**											
	Equilibrium	6	4036	4929	0.933	0.856-1.016	0.109	F	5.763	0.330	13.23	0.451
	Disequilibrium	1	326	709	0.994	0.763-1.295	0.966	F	0.0	1.0	0.0	NA
	**Quality score**											
	High quality	6	4051	5341	0.954	0.877-1.038	0.275	F	3.544	0.617	0.0	0.914
	Low quality	1	311	297	0.734	0.533-1.010	0.058	F	0.0	1.0	0.0	NA

OR: odds ratio, CI: confidence interval, F: fixed model, R: random model, NA: not appreciable.

Supplementary Table S28. Overall survival analysis.

Gene name	Mutation state	Kaplan Meier curve								Cox regression				
		No of samples	Number at risk	Number of event	Number of censor	5-year survival probability			Log test	HR	HR lower	HR upper	Wald test	Likelihood test
						Mean	Lower mean	Upper mean						
DDX20	WT	7713	1359	2079	4275	0.60	0.58	0.61						
	MUT	49	9	14	26	0.63	0.45	0.76	0.441	0.82	0.49	1.36	0.442	0.427
**DGCR8**	**WT**	7683	1359	2064	4260	0.60	0.58	0.61						
	**MUT**	79	9	29	41	0.48	0.32	0.63	**0.026**	1.51	1.05	2.18	**0.027**	**0.038**
GEMIN4	WT	7711	1360	2079	4272	0.60	0.58	0.61						
	MUT	51	8	14	29	0.57	0.36	0.73	0.576	1.14	0.72	1.81	0.577	0.585
DICER1	WT	7615	1341	2048	4226	0.60	0.58	0.61						
	MUT	147	27	45	75	0.58	0.48	0.67	0.416	1.12	0.85	1.49	0.417	0.425
**PIWIL1**	**WT**	7639	1340	2068	4231	0.59	0.58	0.61						
	**MUT**	123	28	25	70	0.69	0.56	0.78	**0.033**	0.68	0.47	0.97	**0.034**	**0.024**
AGO1	WT	7679	1361	2069	4249	0.60	0.58	0.61						
	MUT	83	7	24	52	0.53	0.37	0.67	0.222	1.27	0.86	1.87	0.223	0.241
TARBP2	WT	7722	1360	2086	4276	0.60	0.58	0.61						
	MUT	40	8	7	25	0.70	0.44	0.86	0.109	0.55	0.26	1.15	0.114	0.079
AGO2	WT	7666	1352	2066	4248	0.60	0.58	0.61						
	MUT	96	16	27	53	0.60	0.47	0.72	0.902	0.98	0.67	1.42	0.902	0.902
XPO5	WT	7720	1359	2082	4279	0.60	0.58	0.61						
	MUT	42	9	11	22	0.67	0.47	0.81	0.813	1.06	0.64	1.77	0.813	0.815
RAN	WT	7743	1363	2087	4293	0.60	0.58	0.61						
	MUT	19	5	6	8	0.61	0.32	0.81	0.622	1.19	0.59	2.38	0.622	0.632
DROSHA	WT	7655	1349	2061	4245	0.60	0.58	0.61						
	MUT	107	19	32	56	0.56	0.43	0.68	0.983	1.00	0.71	1.39	0.983	0.983

Supplementary Table S29. Disease-specific survival analysis.

Gene name	Mutation state	Kaplan Meier curve								Cox regression				
		No of samples	Number at risk	Number of event	Number of censor	5-year survival probability			Log test	HR	HR lower	HR upper	Wald test	Likelihood test
						Mean	Lower mean	Upper mean						
DDX20	WT	7640	1359	1509	4772	0.68	0.67	0.70						
	MUT	49	9	13	27	0.66	0.48	0.79	0.784	1.08	0.63	1.83	0.784	0.786
DGCR8	WT	7611	1359	1503	4749	0.68	0.67	0.70						
	MUT	78	9	19	50	0.58	0.39	0.73	0.195	1.35	0.86	2.12	0.196	0.218
GEMIN4	WT	7640	1360	1511	4769	0.68	0.67	0.70						
	MUT	49	8	11	30	0.66	0.45	0.80	0.469	1.22	0.71	2.11	0.470	0.484
DICER1	WT	7546	1340	1492	4714	0.68	0.67	0.70						
	MUT	143	28	30	85	0.69	0.59	0.78	0.974	0.99	0.70	1.42	0.974	0.974
**PIWIL1**	**WT**	7569	1341	1507	4721	0.68	0.67	0.70						
	**MUT**	120	27	15	78	0.79	0.67	0.87	**0.007**	0.51	0.31	0.84	**0.008**	**0.003**
AGO1	WT	7610	1360	1506	4744	0.68	0.67	0.70						
	MUT	79	8	16	55	0.65	0.47	0.78	0.601	1.14	0.70	1.83	0.601	0.609
TARBP2	WT	7650	1360	1516	4774	0.68	0.67	0.70						
	MUT	39	8	6	25	0.72	0.45	0.88	0.312	0.66	0.30	1.48	0.315	0.281
AGO2	WT	7596	1353	1503	4740	0.68	0.67	0.70						
	MUT	93	15	19	59	0.70	0.56	0.80	0.878	0.97	0.61	1.52	0.878	0.877
XPO5	WT	7648	1359	1517	4772	0.68	0.67	0.70						
	MUT	41	9	5	27	0.81	0.59	0.92	0.539	0.80	0.40	1.61	0.539	0.524
RAN	WT	7670	1363	1518	4789	0.68	0.67	0.70						
	MUT	19	5	4	10	0.69	0.35	0.88	0.564	1.27	0.57	2.82	0.565	0.580
DROSHA	WT	7585	1349	1497	4739	0.68	0.67	0.70						
	MUT	104	19	25	60	0.63	0.48	0.74	0.948	1.01	0.69	1.49	0.948	0.948
